
JOURNAL INFORMATION
==============================
NLM Title Abbreviation: Eur Psychiatry
Journal ID: Eur Psychiatry
NLM Journal ID: 9111820
Journal ID: EPA

European Psychiatry

ISSN: 0924-9338
EISSN: 1778-3585
Publisher: Cambridge University Press

ARTICLE INFORMATION
==============================
PMCID: PMC13070663
DOI: 10.1192/j.eurpsy.2020.6
Article ID: S0924933820000061
Article version: 1
Subjects: Abstracts

E-Poster Viewing

Publication date: 2020 Jul
Electronic publication date: 2020 Sep 4
Volume: 63
Issue: Suppl 1
Issue title: Abstracts of the 28th European Congress of Psychiatry
First page: S283
Last page: S589
Copyright: © The Author(s) 2020
Copyright year: 2020
Copyright holder: The Author(s)
License: This is an Open Access article, distributed under the terms of the Creative Commons Attribution licence (http://creativecommons.org/licenses/by/4.0/), which permits unrestricted re-use, distribution, and reproduction in any medium, provided the original work is properly cited.
License URL: https://creativecommons.org/licenses/by/4.0/

Figure count: 78
Table count: 10
Page count: 307
displayText: Special Issue
tdm-reservation: 1
tdm-policy: https://www.cambridge.org/tdm/tdmrep-policy.json

==============================

Article ID: EPV0001
Subjects: Abstract, Addictive Disorders

RANZCP position: e-cigarettes and vaporisers

Allan J.
Royal Australian and New Zealand College of Psychiatrists , President, Melbourne , Australia
Keywords: e-cigarettes, vaporisers

Conflict of interest:

No

Article ID: EPV0002

The relationship between alexithymia and suicide among moroccan patients with substance use disorders

Karjouh K. 1
Azzaoui F.-Z. 2 *
Ahami A.O.T. 3
Hami H. 4
Boulbaroud S. 5
1 Unit of Cognitive and Behavioral Neuroscience and Applied Nutrition, Faculty of Science, Department of Biology, KENITRA , Morocco
2 Unit of Cognitive and Behavioral Neuroscience and Applied Nutrition, Faculty of Science, Department of Biology, kenitra , Morocco
3 Unit of Cognitive and Behavioral Neuroscience and Applied Nutrition, Faculty of Science, Department of Biology, Kenitra , Morocco
4 Laboratory of Genetics and Biometry, Faculty of Science, Ibn Tofail University , Department of Biology, Kenitra , Morocco
5 Polydisciplinary Faculty- Sultan Moulay Slimane University , Polydisciplinary Faculty, Benimellal , Morocco
* Corresponding author.
Keywords: Suicide, Addiction, Morocco, Alexithymia

Conflict of interest:

No

Article ID: EPV0003

Suicidality in a sample of patients with gambling disorder

Carmassi C. 1
Barberi F.M. 1 *
Dell'Oste V. 1
Bertelloni C.A. 1
Cremone I.M. 1
Pedrinelli V. 1
Cordone A. 1
Mannari P. 2
Pioli E. 2
Maglio A. 1
Dell'Osso L. 1
1 University of Pisa , Department of Clinical and Experimental Medicine, Pisa , Italy
2 Asl Toscana Nord Ovest , Serd, Lucca , Italy
* Corresponding author.
Keywords: Suicide, Gambling, behavioral addictions, Suicidality

Conflict of interest:

No

Article ID: EPV0004

Problematic use of the internet (PUI) and post-traumatic stress spectrum symptoms in patients with bipolar disorder

Carmassi C.
Bertelloni C.A.
Barberi F.M. *
Dell'Oste V.
Maglio A.
Cordone A.
Pedrinelli V.
Cremone I.M.
Dell’Osso L.
University of Pisa , Department of Clinical and Experimental Medicine, Pisa , Italy
* Corresponding author.
Keywords: PTSD, Bipolar disorder, internet addiction

Conflict of interest:

No

Article ID: EPV0005

The effect of psychotherapeutic treatment in co-dependent relatives of drug-dependent patients on the efficiency of the treatment-rehabilitation process.

Bereza Z. 1 *
Isaeva E. 2
1 Bechterev Medical Centre , Psychiatry, Saint- Petersburg , Russian Federation
2 Medical State University n.a Pavlov , Psychology, Saint-Peterburg , Russian Federation
* Corresponding author.
Keywords: in-patient rehabilitation, co-dependent psychotherapy, co-dependence, complex medical rehabilitation of drug addicts

Conflict of interest:

No

Article ID: EPV0006

Age, gender and personality: factors linked with binge-watching

Jolly C.
Bristuile C. *
Romo L.
Université Paris Nanterre , Psychology, Nanterre , France
* Corresponding author.
Keywords: Binge-Watching, Personality, Series, BFI-10

Conflict of interest:

No

Article ID: EPV0008

Cannabinoid hyperemesis syndrome: two cases managed for 10 years in ramón y cajal hospital´s psychiatry department

Caballero I. 1 *
Correa A. 2
Guillen S. 1
Ochoa Mangado E. 1
1 University Hospital Ramon y Cajal , Psychiatry Department, Madrid , Spain
2 Ramon y Cajal Hospital , Psychiatry, Madrid , Spain
* Corresponding author.
Keywords: chronic users, cannabis, cyclical hyperemesis, vomiting

Conflict of interest:

No

Article ID: EPV0009

Could the cloninger’s personality characteristics distinguish addiction problems in korea?

Lee S.J. 1
Chae H. 2 *
1 Kyungsung University , Department of Psychology, Busan , Korea , Republic of
2 Pusan National University , School of Korean Medicine, Busan , Korea , Republic of
* Corresponding author.
Keywords: Alcohol problem, temperament, gambling problem, Character

Conflict of interest:

No

Article ID: EPV0012

A case series of inpatient treatment of GHB/GBL addiction

Delic M.
University Psychiatric Clinic Ljubljana , Center for Treatment Of Drug Addiction, Ljubljana , Slovenia
Keywords: treatment, GHB/GBL, inpatient, withdrawal

Conflict of interest:

No

Article ID: EPV0013

Polysomnographic features of opiate use disorder

Elsheshai A. 1 *
Mekky J. 2
Rady A. 2
Elkholy O. 2
Molokhia T. 2
1 Maamoura Psychiatric Hospital , Psychiatry, Alexandria , Egypt
2 Alexandria Faculty of Medicine, Neuropsychiatry , Alexandria , Egypt
* Corresponding author.
Keywords: Addiction, polysomnography, sleep architecture, substance use

Conflict of interest:

No

Article ID: EPV0014

Protracted abstinence syndrome in recovering opiate addicts

Elsheshai A. 1 *
Mekky J. 2
Rady A. 2
Elkholy O. 2
Molokhia T. 2
1 Maamoura Psychiatric Hospital , Psychiatry, Alexandria , Egypt
2 Alexandria Faculty of Medicine, Neuropsychiatry , Alexandria , Egypt
* Corresponding author.
Keywords: substance use, opiate, Relapse prevention, Addiction

Conflict of interest:

No

Article ID: EPV0017

Addictive behavior and free will: from philosophy to neurobiology

Froes V. *
Afonso H.
Esteves S.
Carneiro M.
Torres S.
Vilas Boas J.
Gameiro Z.
Centro Hospitalar Barreiro Montijo, Psiquiatria E Saúde Mental , Barreiro , Portugal
* Corresponding author.
Keywords: free will, reward system, Addiction

Conflict of interest:

No

Article ID: EPV0024

The feeling of guilt experienced by adults whose parents were alcohol addicts (case-study research)

Lutsenko A. 1 *
Spivakovskaya A. 2
1 Moscow State University , Psychology, Moscow , Russian Federation
2 Lomonosov Moscow State University , Psychology, Moscow , Russian Federation
* Corresponding author.
Keywords: guilt, 12-step recovery program, Alcohol addiction, dysfunctional family

Conflict of interest:

No

Article ID: EPV0025

The negative emotions experienced by adults whose parents were alcohol addicts

Lutsenko A. 1 *
Spivakovskaya A. 2
1 Moscow State University , Psychology, Moscow , Russian Federation
2 Lomonosov Moscow State University , Psychology, Moscow , Russian Federation
* Corresponding author.
Keywords: guilt, Alcohol addiction, shame, negative emotions

Conflict of interest:

No

Article ID: EPV0027

The bridge between classical and ‘synthetic’/chemical psychoses: towards a clinical, psychopathological and therapeutic perspective

Orsolini L.
University of Hertfordshire , Psychopharmacology Drug Misuse and Novel Psychoactive Substances Research Unit, Herts , Italy
Keywords: Synthetic psychosis, psychosis, Novel Psychoactive Substances

Conflict of interest:

No

Article ID: EPV0028

The role of day hospital in the treatment of dual pathology

Palma Conesa Á.J. *
Represa Martinez Á.
Moreno Bustos J.
Merino Del Villar C.
Àrea de Salut Eivissai Formentera , Psychiatry, Ibiza , Spain
* Corresponding author.
Keywords: dual pathology, partial hospitalization, hospital day clinic

Conflict of interest:

No

Article ID: EPV0029

Life-meaningful orientations and coping styles in women from dysfunctional families

Pervichko E. *
Kshniankina S.
Koniukhovskaia J.
Lomonosov Moscow State University , Faculty of Psychology, Moscow , Russian Federation
* Corresponding author.
Keywords: adult children of alcoholics, Twelve-step program, coping behaviour, life-meaningful orientations

Conflict of interest:

No

Article ID: EPV0030

Inhibitory control in patients with alcohol-use disorder: an EEG study

Peshkovskaya A. *
Galkin S.
Tomsk National Research Medical Center, Russian Academy of Sciences, Mental Health Research Institute , Tomsk , Russian Federation
* Corresponding author.
Keywords: alcohol-use disorder, EEG, inhibitory control, Cognition

Article ID: EPV0032

Internet gaming disorder: case report

Ponizovskiy P. *
Khanykov V.
Gofman A.
Moscow Research Institute of Psychiatry – branch of V.Serbsky National Medical Research Center for Psychiatry and Addiction, Department For Treatment Of Mental Disorders, Complicated By Addictions, Moscow, Russian Federation
* Corresponding author.
Keywords: internet addiction, internet gaming disorder, case report, Problematic Internet Use

Conflict of interest:

No

Article ID: EPV0036

Predictive ability of addictive beliefs

Suarez Pérez C. *
Ballesteros López J.
Álvarez González V.
Hospital Universitario Infanta Cristina , Psychiatry, Madrid , Spain
* Corresponding author.
Keywords: dual disorders, nuclear beliefs, substances, craving

Conflict of interest:

No

Article ID: EPV0039

Addiction treatment services in belarus.

Yarmalinskaya A.
health care facilicy city clinical drug dispensary , Narcology , Misk , Belarus
Keywords: Addiction, treatment service, health care, alcohol, Addiction, treatment service, health care

Conflict of interest:

No

Article ID: EPV0040

Childhood trauma, emotion regulation and pain in alcohol-dependent individuals

Zaorska J. 1 *
Kopera M. 1
Nowakowska M. 1
Kobyliński P. 2
Wojnar M. 3
Jakubczyk A. 1
1 Medical University of Warsaw , Psychiatry, Warsaw , Poland
2 National Information Processing Institute , Laboratory of Interactive Technologies, Warsaw , Poland
3 Medical University of Warsaw, Psychiatry Clinic , Warsaw , Poland
* Corresponding author.
Keywords: childhood trauma, pain, emotion regulation

Conflict of interest:

No

Article ID: EPV0041

Cannabis use and risk perception: a review.

Arribas Pinero E. *
León-Quismondo L.
RAMON Y CAJAL UNIVERSITY HOSPITAL , Psychiatry, MADRID , Spain
* Corresponding author.
Keywords: cannabis, risk perception, risk factor, false believes

Conflict of interest:

No

Article ID: EPV0046

Peculiarities of alcohol dependence formation in combatants as a basis for their rehabilitation

Kozhyna H. *
Lytvynenko V.
Zelenska K.
Koshchii V.
Radchenko T.
KHARKIV NATIONAL MEDICAL UNIVERSITY , Psychiatry, Narcology And Medical Psychology, Kharkiv, Ukraine
* Corresponding author.
Keywords: Alcohol dependence, combatants, rehabilitation, psychocorrection

Conflict of interest:

No

Article ID: EPV0047

Specificity of psychopathological symptoms, associated with disorders related to alcohol consumption, in combatants, forcibly displaced persons and ordinary residents

Markov A. 1 *
Gaponov K. 2
Markova M. 3
Koshchii V. 4
Chernyaev M. 1
1 Kharkiv Medical Academy of Postgraduate Education , Sexology, Medical Psychology Medical And Psychological Rehabilitation, Kharkiv, Ukraine
2 Kharkiv Medical Academy of Postgraduate Education , Narcology, Kharkiv , Ukraine
3 Kharkiv Medical Academy of Postgraduate Education , Sexology, Medical Psychology, Medical Abd Psychological Rehabilitation, Kharkiv, Ukraine
4 Kharkiv National Medical University , Psychiatry,narcology And Medical Psychology, Kharkiv, Ukraine
* Corresponding author.
Keywords: Alcohol addiction, Psychosocial Stress, combatants, forcibly displaced persons

Conflict of interest:

No

Article ID: EPV0048

Characteristics of addictive status in patients with alcohol dependence and different levels of macrosocial stress

Markova M. 1 *
Gaponov K. 2
Markov A. 3
Kozhyna H. 4
Koshchii V. 5
1 Kharkiv Medical Academy of Postgraduate Education , Sexology, Medical Psychology, Medical Abd Psychological Rehabilitation, Kharkiv, Ukraine
2 Kharkiv Medical Academy of Postgraduate Education , Narcology, Kharkiv , Ukraine
3 Kharkiv Medical Academy of Postgraduate Education , Sexology, Medical Psychology Medical And Psychological Rehabilitation, Kharkiv, Ukraine
4 KHARKIV NATIONAL MEDICAL UNIVERSITY , Psychiatry, Narcology And Medical Psychology, Kharkiv, Ukraine
5 Kharkiv National Medical University , Psychiatry,narcology And Medical Psychology, Kharkiv, Ukraine
* Corresponding author.
Keywords: ADDICTIVE STATUS, MACROSOCIAL STRESS, ALCOHOL DEPENDENCE

Conflict of interest:

No

Article ID: EPV0055

Novel link between poor impulse control and high caffeine intake and expectancies

Cebrián J. 1 *
Gonzalez-Cuevas G. 1 2
1 European University of Madrid , Department of Psychology, Madrid , Spain
2 Idaho State University , Department of Biomedical And Pharmaceutical Sciences, Meridian , United States of America
* Corresponding author.
Keywords: Caffeine, impulsivity, intake, expectancies

Conflict of interest:

No

Article ID: EPV0056

Alcohol related dementia: a distinct or multifactorial nosological entity?

Costa P. 1 *
Melo M. 1
Branco P. 1
Teixeira J. 2
1 Centro Hospitalar Psiquiatrico de Lisboa - CHPL , Psicogeriatria, Lisboa , Portugal
2 Centro Hospitalar Psiquiátrico de Lisboa , Serviço Alcoologia E Novas Dependências, Lisboa , Portugal
* Corresponding author.
Keywords: alcohol related dementia, Wernicke-Korsakoff syndrome, thiamine

Conflict of interest:

No

Article ID: EPV0057

Psychosocial factors involved in substance use behavior in young medical undergraduate

Mihailescu A. 1 2
Ciobanu A. 3 4
Iorga B. 2
Damian A. 3 *
Mihailescu C. 5
Popa-Velea O. 2
1 Clinical Hospital of Psychiatry „Prof dr Al. Obregia”, Psychiatry 1, Bucharest , Romania
2 University of Medicine and Pharmacy „Carol Davila” , Medical Psychology, Bucharest , Romania
3 ”Prof. Alexandru Obregia” Clinical Hospital of Psychiatry, Psychiatry 1, Bucharest , Romania
4 ”Prof. Alexandru Obregia” Clinical Hospital of Psychiatry, 1st, Bucharest , Romania
5 CMI MIhailescu S. Cristian, General Practice , Bucharest , Romania
* Corresponding author.
Keywords: cannabis, mental health, medical undergraduates, substance use

Conflict of interest:

No

Article ID: EPV0058

Regular alcohol use in young adults is associated with reduced motivation to work for financial reward

David C. 1 2 *
Ersche K. 3
Lim T.V. 3
1 Brunel University London , Psychology, London , United Kingdom
2 Cambridge University , Psychiatry, Cambridge , United Kingdom
3 University of Cambridge , Department of Psychiatry, Cambridge , United Kingdom
* Corresponding author.
Keywords: Motivation, monetary, reinforcement sensitivity, Alcohol

Conflict of interest:

No

Article ID: EPV0060

Pramipexole differentially modulates fronto-striatal circuits in addictive and compulsive disorders

Dionelis K. 1 *
Meng C. 1
Craig K. 1
Shabbir S. 2
Fineberg N. 3
Sahakian B. 4
Suckling J. 1
Bullmore E. 4
Robbins T. 4
Ersche K. 4
1 University of Cambridge , Department of Psychiatry, Cambridge , United Kingdom
2 GlaxoSmithKline, Clinical Unit Cambridge , Cambridge , United Kingdom
3 Hertfordshire Partnership University NHS Foundation Trust , Psychiatry, Welwyn Garden City , United Kingdom
4 Cambridge university , Department of Psychiatry And Behavioural And Clinical Neuroscience Institute, Cambridge , United Kingdom
* Corresponding author.
Keywords: compulsivity addiction OCD dopamine

Article ID: EPV0066

“Correlation of urine ethyl glucuronide (ETG) and ethyl sulfate (ETS) with other markers of recent alcohol use and factors affecting its levels”.

Huddar S. *
Murthy P.
Sharma P.
Chand P.
Shukla L.
National Institute of Mental Health and Neurosciences(NIMHANS) , Psychiatry, Bengaluru , India
* Corresponding author.
Keywords: Ethyl Glucuronide, Ethyl Sulfate, Alcohol, biomarker

Conflict of interest:

No

Article ID: EPV0070

The relationship between family history and early onset alcoholism: more rapid transition to dependence or denial of their addiction?

Patrascu M.R. *
Ionescu T.C.
Dumitrescu M.R.
Ladea M.
Alexandru Obregia Clinical Hospital , Department 3, Bucharest , Romania
* Corresponding author.
Keywords: family history, alcoholism, denial, Early Onset

Conflict of interest:

No

Article ID: EPV0071

The relationship between family history and early onset alcoholism: more rapid transition to dependence or denial of their addiction?

Patrascu M.R. 1 *
Ionescu T.C. 1
Dumitrescu M.R. 1
Ladea M. 2
1 Alexandru Obregia Clinical Hospital , Department 3, Bucharest , Romania
2 University of Medicine and Pharmacy “Carol Davila” , Psychiatry, Bucharest , Romania
* Corresponding author.
Keywords: Early Onset, denial, Addiction, family history

Conflict of interest:

No

Article ID: EPV0079

Psychometric properties of the transaddiction craving triggers questionnaire in alcohol use disorder

Von Hammerstein C. 1 2 *
Cornil A. 3
Rothen S. 4
Romo L. 5 6
Khazaal Y. 7
Benyamina A. 2
Billieux J. 8 9
Luquiens A. 2 10
1 Université parisnanterre , Ea 4430 Clipsyd, nanterre, France
2 Hôpital Paul brousse APHP , Department of Psychiatry And Addictologyuniversity Paris-sud , Uvsq, Cesp , Inserm U 1178, villejuif, France
3 Université Catholique de Louvain , Laboratory For Experimental Psychopathology (lep), Psychological Science Research Institute ,, Louvain- la-neuve , Belgium
4 University of geneva , Research Center for Statistics, Geneva School of Economics And Management,, Geneva , Switzerland
5 Saint Anne Hospital , Inserm, U894 , Center for Psychiatry And Neuroscience,, Paris, France
6 Université Paris Nanterre , Clipsyd Ea4430, Nanterre , France
7 University hospital Lausanne , Addiction Medecine, Lausanne , Switzerland
8 University of Lausanne , Institut Of Psychology, Lausanne , Switzerland
9 University of luxembourg, Addictive And Compulsive Behaviorslab. Institute For Health And Behaviour ,, luxembourg , Luxembourg
10 CHU Nîmes - Inserm , Addiction, Nîmes , France
* Corresponding author.
Keywords: psychometrics, triggers, alcohol use disorder, craving

Article ID: EPV0080

Two faces of exercise addiction - self-esteem, narcissism and sport addiction in women

Wojtyna E. 1 *
Król T. 2
Hyla M. 1
1 University of Silesia in Katowice, Institute of Psychology, Katowice, Poland
2 Medical University of Silesia , School of Public Health, Katowice , Poland
* Corresponding author.
Keywords: exercise addiction, narcissism, self-esteem

Conflict of interest:

No

Article ID: EPV0083

The role of different game-genres in predicting internet gaming disorder (IGD)

Ferraro L. 1 *
Avanzato C. 1
Maniaci G. 1
Sartorio C. 1
Seminerio F. 1
Tripoli G. 2
Quattrone D. 3
Daino M. 1
La Barbera D. 1
La Cascia C. 1
1 University of Palermo , Psychiatry section, Biomedicine , Neuroscience And Advanced Diagnostic (bind) , Palermo , Italy
2 King’s College London , Psychosis Studies, London , United Kingdom
3 King’s College London , Social , Genetic , And Developmental Psychiatry Centre Institute of Psychiatry , Psychology, And Neuroscience, London, United Kingdom
* Corresponding author.
Keywords: MMORPG, MINECRAFT, playing time, Alexithymia

Conflict of interest:

No

Article ID: EPV0084

Differences between female and male gamers and gender-specific risk-factors for internet gaming disorder (IGD)

Ferraro L. 1 *
Avanzato C. 1
Maniaci G. 1
Sartorio C. 1
Seminerio F. 1
Tripoli G. 1 2
Quattrone D. 3
Daino M. 1
La Barbera D. 1
La Cascia C. 1
1 University of Palermo , Psychiatry section, Biomedicine , Neuroscience And Advanced Diagnostic (bind) , Palermo , Italy
2 King’s College London , Psychosis Studies, London , United Kingdom
3 King’s College London , Social , Genetic , And Developmental Psychiatry Centre Institute of Psychiatry , Psychology, And Neuroscience, London, United Kingdom
* Corresponding author.
Keywords: gender psychiatry, DSM 5, videogames, time spent playing

Conflict of interest:

No

Article ID: EPV0085

Service evaluation of child safeguarding referral process from the drug and alcohol service in SWLSTG mental health trust

Nagasinghe P.
SOUTHWEST LONDON & ST GEORGE’S MENTAL HEALTH NHS TRUST, Neurodevelopment Services, TOOTING , United Kingdom
Keywords: Child safeguarding, Drugs and Alcohol Service, Social Service, Parental responsibility

Conflict of interest:

No

Article ID: EPV0092
Subjects: Abstract, Anxiety Disorders and Somatoform Disorders

Treatment of social anxiety disorder in a public context. About a case.

Carracedo Sanchidrián D.
Hospital Universitario la Paz, Mental Health , Madrid , Spain
Keywords: Social Anxiety Disorder, cognitive behavioral therapy, public health

Conflict of interest:

No

Article ID: EPV0094

Comparative efficacy and tolerability of 14 benzodiazepines in the treatment of patients with anxiety: a network meta-analysis

Fernandes H. 1 *
Novais C. 2
Moreira R. 3
1 BIAL - Portela & C.ª, S.A ., Department of Research And Development, Coronado (S. Romão e S. Mamede) , Portugal
2 BIAL - Portela & C.ª, S.A , Department of Research And Development, Coronado (S. Romão e S. Mamede) , Portugal
3 São João University Hospital Centre, Psychiatry And Mental Health Clinic , Porto , Portugal
* Corresponding author.
Keywords: benzodiazepines, Anxiety, network meta-analysis, efficacy and tolerability

Article ID: EPV0095

Adherence to treatment among patients with chronic rhinosinusitis and anxiety disorders

Pervichko E. 1
Kim I. 2
Vinogradov V. 2
Grishanina A. 1 *
1 Lomonosov Moscow State University , Faculty of Psychology, Moscow , Russian Federation
2 Federal State Budgetary Institution " Scientific and Clinical Center of Otorhinolaryngology of the Federal Medico-Biological Agency of the Russian Federation , Head And Neck Cancer, Moscow , Russian Federation
* Corresponding author.
Keywords: Anxiety, adherence to treatment, emotion regulation, Reactions to frustration

Conflict of interest:

No

Article ID: EPV0098

Efficacy of acceptance and commitment therapy in the treatment of anxiety disorders

León-Quismondo L. 1 *
López Ríos F. 2
Fernández Liria A. 3
Lahera G. 4
1 Ramon y Cajal University Hospital , Psychiatry, Madrid , Spain
2 University of Almería , Psychology, Almería , Spain
3 Hospital Universitario Príncipe de Asturias , Psychiatry, Alcalá de Henares , Spain
4 University of Alcala , School of Medicine, Alcalá de Henares , Spain
* Corresponding author.
Keywords: anxiety disorders, Acceptance and Commitment Therapy, Efficacy

Conflict of interest:

No

Article ID: EPV0099

Predictors of efficacy of cognitive behavioral therapy in patients with panic disorder

León-Quismondo L. 1 *
Rodríguez Pedraza E. 2
Fernández Liria A. 3
Lahera G. 4
1 Ramon y Cajal University Hospital , Psychiatry, Madrid , Spain
2 Príncipe de Asturias University Hospital , Psychiatry, Alcalá de Henares, Madrid , Spain
3 Hospital Universitario Príncipe de Asturias , Psychiatry, Alcalá de Henares , Spain
4 University of Alcala , School of Medicine, Alcalá de Henares , Spain
* Corresponding author.
Keywords: cognitive behavioral therapy, panic disorder, predictors

Conflict of interest:

No

Article ID: EPV0100

Supporting anxiety management through mHealth: methods and first contributions considering the academic campus

Ferreira D. 1
Melo D. 2
Silva P. 2
Santo A. 2
Madeira N. 3 *
Soares S. 4
Silva S. 1
1 University of Aveiro , Department of Electronics, Telecommunication And Informatics (deti)/Institute of Electronics And Informatics Engineering (ieeta), Aveiro , Portugal
2 University of Aveiro , Department of Education And Psychology, Aveiro , Portugal
3 University of Coimbra , Faculty of Medicine, Department of Psychological Medicine, Coimbra , Portugal
4 William James Center for Research, University of Aveiro , Department of Education And Psychology, Aveiro , Portugal
* Corresponding author.
Keywords: higher education, mHealth, Anxiety, mobile health

Conflict of interest:

No

Article ID: EPV0102

Anxiety during pregnancy, about 62 cases

Mnif D. *
Sellami R.
HEDI CHAKER hospital , Psychiatric Service, SFAX , Tunisia
* Corresponding author.
Keywords: Anxiety, anxiety trait, anxiety state, Pregnancy

Conflict of interest:

No

Article ID: EPV0103

Phantom phone signals in undergraduate university students of different faculties

Nikolaev E.
Ulianov Chuvash State University , Department of Social And Clinical Pscyhology, Cheboksary , Russian Federation
Keywords: Anxiety, phantom phone signals, University students, smartphone use

Conflict of interest:

No

Article ID: EPV0104

Mechanisms of anxiety – a reappraisal on GABAA receptor subunits and role of benzodiazepines

Novais C. 1 *
Fernandes H. 2
Vieira-Coelho M. 3
1 BIAL - Portela & C.ª, S.A , Department of Research And Development, Coronado (S. Romão e S. Mamede) , Portugal
2 BIAL - Portela & C.ª, S.A ., Department of Research And Development, Coronado (S. Romão e S. Mamede) , Portugal
3 Centro Hospitalar Universitário de São João , Psychiatry, Porto , Portugal
* Corresponding author.
Keywords: benzodiazepine, GABAA receptor, Anxiety

Article ID: EPV0105

Role of hope, personality, dissociation, and self-stigma in the treatment of the resistant anxiety disorders

Ociskova M. 1
Prasko J. 1 *
Slepecky M. 2
Kotianova A. 2
1 Faculty of Medicine and Dentistry, University Palacky Olomouc , Department of Psychiatry, Olomouc , Czech Republic
2 Faculty of Social Science and Health Care - Constantine the Philosopher University in Nitra- Slovak Republic ,, Department of Psychology Sciences, Nitra , Slovak Republic
* Corresponding author.
Keywords: anxiety disorders, dissociation, self-stigma, hope

Conflict of interest:

No

Article ID: EPV0106

Relationship between self-stigma and personality in mixed neurotic spectrum and depressive disorders

Ociskova M.
Prasko J. *
Faculty of Medicine and Dentistry, University Palacky Olomouc , Department of Psychiatry, Olomouc , Czech Republic
* Corresponding author.
Keywords: self-stigma, / hope /, anxiety disorders, Dépression

Conflict of interest:

No

Article ID: EPV0107

Dissociation and therapy of depressive and anxiety disorders with or without personality disorders

Prasko J. 1 *
Ociskova M. 1
Slepecky M. 2
Kotianova A. 2
1 Faculty of Medicine and Dentistry, University Palacky Olomouc , Department of Psychiatry, Olomouc , Czech Republic
2 Faculty of Social Science and Health Care - Constantine the Philosopher University in Nitra- Slovak Republic ,, Department of Psychology Sciences, Nitra , Slovak Republic
* Corresponding author.
Keywords: Dépression, dissociation, cognitive behavioral therapy, anxiety disorders

Conflict of interest:

No

Article ID: EPV0108

I know i will die, but when?

Puljic K. 1 *
Herceg M. 2
Herceg D. 3
1 University Psychiatric Hospital Vrapče , Department For Psychotic Disorders, Zagreb , Croatia
2 1School of Medicine, University of Zagreb , Croatia 2 University Psychiatric Hospital Vrapče , Zagreb, Croatia, Department For Psychotic Disorders, Zagreb, Croatia
3 School of Medicine, University of Zagreb , School Pf Medicine, Zagreb , Croatia
* Corresponding author.
Keywords: Brugada syndrome, Psychiatry, Anxiety, womwn

Conflict of interest:

No

Article ID: EPV0109

Psychosomatic diseases as a result of stress caused by robberies

Sabo T. 1 *
Vranko M. 2
Bosnjak Kuharic D. 3
Cervenjak T. 4
Brečić P. 5
Jendričko T. 6
1 University Psychiatric Hospital Vrapce , Department of Affective Disorders, Zagreb , Croatia
2 University Psychiatric Hospital Vrapče , Department of Psychotherapy, Department of Social Pedagogy, Zagreb , Croatia
3 University Psychiatric Hospital Vrapce , Department For Diagnostics And Intesive Care, Zagreb , Croatia
4 General Hospital Vinkovci , Department For General Psychiatry, Vinkovci , Croatia
5 University Psychiatric Hospital Vrapče , Department of Affective Disorders, Zagreb , Croatia
6 University Psychiatric Hospital Vrapče , Department of Psychotherapy, Zagreb , Croatia
* Corresponding author.
Keywords: Psychosomatic disease; Traumatic event; psychotherapy,Posttraumatic Stress Disorder

Conflict of interest:

No

Article ID: EPV0110

The rise of severe anxiety when exposed to food – a specific phobia case report

Santos N. *
Alho A.
Gasparinho R.
Ferreira L.
Martins M.
Fernandes N.
Sêco E.
Hospital Distrital de Santarém, Psychiatry And Mental Health , Santarém , Portugal
* Corresponding author.
Keywords: Anxiety, Specific phobias, phagophobia

Conflict of interest:

No

Article ID: EPV0111

Life-threatening somatic symptom disorder: a case report

Santos N. *
Alho A.
Martins M.
Fernandes N.
Ferreira L.
Gasparinho R.
Sêco E.
Hospital Distrital de Santarém, Psychiatry And Mental Health , Santarém , Portugal
* Corresponding author.
Keywords: somatic symptom disorder, Hypochondriasis

Conflict of interest:

No

Article ID: EPV0113

Therapeutic management of anxiety disorders due to multiple sclerosis

Vasiliu O.
University Emergency Central Military Hospital Dr. Carol Davila , Psychiatry, Bucharest , Romania
Keywords: generalized anxiety disorder, multiple sclerosis, anxiety disorders, panic disorder

Article ID: EPV0115

Virtual/augmented reality and neurofeedback in the treatment of agoraphobia and social phobia - a comparative and randomized study

Canais F. 1 *
Maçorano R. 1
Ferreira H.A. 1
Charraz A. 2
Martins Y. 2
Matos Pires A. 2
Suárez-Gómez M. 2
1 Faculty of Sciences of Lisbon University , Biophysics And Biomedical Engineering Institute, Lisbon , Portugal
2 Unidade Local de Saúde do Baixo Alentejo , Psychiatry, Beja , Portugal
* Corresponding author.
Keywords: Virtual/Augmented Reality, Digital therapeutics, neurofeedback, digital health

Conflict of interest:

No

Article ID: EPV0118

Specifics of emotional regulation and cognitive processing in acute myocardial infarction

Nikolaeva O. 1
Nikolaev E. 2 *
Bogdanov A. 2
Zakharova A. 3
Maksimova N. 3
1 Chuvash Republic Cardiology Clinic, Cardiosurgery Unit , Cheboksary , Russian Federation
2 Ulianov Chuvash State University , Faculty of Medicine, Cheboksary , Russian Federation
3 Ulianov Chuvash State University , Social And Clinical Psychology Department, Cheboksary , Russian Federation
* Corresponding author.
Keywords: emotional regulation, cognitive processing, Alexithymia, acute myocardial infarction

Conflict of interest:

No

Article ID: EPV0122
Subjects: Abstract, Bipolar Disorders

Bipolar disorder misdiagnose: a case report of cushing’s disease psychiatric manifestations.

De Hita Santillana R. 1 *
Cerame Del Campo Á. 2
Costa Ferrera Da Silva M.L. 3
1 Instituto psiquiatrico Jose Germain, Psyhciatric Trainee , leganes , Spain
2 Instituto Psiquiatrico José Germain , Psiquiatría, Leganés , Spain
3 Instituto psiquiatrico Jose Germain , Psyhciatrist, leganes , Spain
* Corresponding author.
Keywords: Bipolar, cushing, ORGANIC

Conflict of interest:

No

Article ID: EPV0123

When there is no other option: ECT in bipolar disorder

De Hita Santillana R. 1 *
Aguilar Romero C. 2
Suárez Velázquez A. 3
Cerame Del Campo Á. 4
1 Instituto psiquiatrico Jose Germain, Psyhciatric Trainee , leganes , Spain
2 Hospital Universitario Severo Ochoa , Uhb, Leganés , Spain
3 Instituto Psiquiátrico Montreal , Hospital De Día-cet, Madrid , Spain
4 Instituto Psiquiatrico José Germain , Psiquiatría, Leganés , Spain
* Corresponding author.
Keywords: electroconvulsive, comorbidities, Bipolar

Conflict of interest:

No

Article ID: EPV0127

Residual symptoms and specific functional impairments in euthymic patients with bipolar disorder

Felhi R. 1 *
Chebli S. 1
Bouallagui A. 2
Karoui M. 3
Ellouze F. 3
1 Razi hospital , Forensic Psychiatry, Manouba , Tunisia
2 Razi hospital , Psychiatry Ward B, Manouba , Tunisia
3 Razi hospital , Psychiatry Ward G, Manouba , Tunisia
* Corresponding author.
Keywords: Bipolar disorder, residual symptoms, functioning

Conflict of interest:

No

Article ID: EPV0129

Hetero-aggressive behavior in bipolar disorders: along the thread of affective temperaments and predominant polarity

Fico G. 1 *
Verdolini N. 1
Anmella G. 1
Pacchiarotti I. 2
Murru A. 2
1 Bipolar and Depressive Disorders Unit, IDIBAPS , CIBERSAM ,, Institute of Neuroscience, Hospital Clinic, University Of Barcelona ,, Barcelona , Spain
2 Bipolar and Depressive Disorders Unit, Hospital Clínic de Barcelona , Psychiatry, Barcelona , Spain
* Corresponding author.
Keywords: aggressive behaviour, predominant polarity, affective temperaments, Bipolar Disorders

Conflict of interest:

No

Article ID: EPV0130

Gender differences in hospitalized patients with bipolar disorder in spain.

García A. 1 *
Hernández-Calle D. 2
Kollias G. 3
Suárez Lorenzo A. 3
Curto Ramos J. 3
Louzao Í.I. 1
Bravo-Ortiz M.F. 3
1 Hospital Universitario La Paz , Psychiatry, Clinical Psychology And Mental Health , Madrid , Spain
2 Hospital Universitario La Paz , Psychiatry, Madrid , Spain
3 La Paz University Hospital , Psychiatry, Clinical Psychology And Mental Health , Madrid , Spain
* Corresponding author.
Keywords: Bipolar disorder, Gender, inpatient

Conflict of interest:

No

Article ID: EPV0131

Comorbidities and gender differences in hospitalized patients with bipolar disorder

García A. 1 *
Hernández-Calle D. 1
Kollias G. 1
Suárez Lorenzo A. 2
Curto Ramos J. 2
Louzao Í.I. 1
Bravo-Ortiz M.F. 3
1 Hospital Universitario La Paz , Psychiatry, Clinical Psychology And Mental Health , Madrid , Spain
2 La Paz University Hospital , Psychiatry, Clinical Psychology And Mental Health , Madrid , Spain
3 La Paz University Hospital Research Health Institute (IdiPAZ) , Psychiatry, Clinical Psychology And Mental Health , Madrid , Spain
* Corresponding author.
Keywords: Gender, inpatient, Bipolar disorder, comorbidities

Conflict of interest:

No

Article ID: EPV0137

From personality disorder to affective one: case report.

Martín Villarroel C. *
Carpio Garcia L.
Dominguez Cutanda J.
Belmonte Garcia G.
Sánchez Revuelta M.
Matsuura J.
Garcia E.
HOSPITAL PROVINCIAL DE LA MISERICORDIA , PsiquiatrÍa (consultas Externas), TOLEDO , Spain
* Corresponding author.
Keywords: mood symptoms, affective disorders, borderline disorder, differential diagnosis

Conflict of interest:

No

Article ID: EPV0138

The combination of pharmacotherapy and a software system at the remission stage for patients with affective disorders

Moroz S. 1 *
Semenikhina V. 2
Makarova I. 2
Khaitov R. 2
Turishcheva N. 2
1 Dneproptrosk Regional Clinic Hospital named after I.I.Mechnikov , Psychosomatic Center, Dnepr , Ukraine
2 Dnipropetrovsk Regional Clinic Hospital named after I.I. Mechnikov , Psychosomatic Center, Dnepr , Ukraine
* Corresponding author.

Conflict of interest:

No

Article ID: EPV0140

Impulsivity, decision-making and risk behavior in bipolar disorder and major depression in bipolar multiplex families.

Ramírez-Martín A. 1 *
Guzmán-Parra J. 1
Streit F. 2
Witt S. 2
Frank J. 2
Sirignano L. 2
Andlauer T. 3
Nöthen M. 4
Forstner A. 5
De Diego-Otero Y. 1
Mayoral F. 6
Rietschell M. 2
Moreno-Küstner B. 7
1 University Regional Hospital of Málaga , Department of Mental Health, Málaga , Spain
2 Central Institute of Mental Health , Department of Genetic Epidemiology In Psychiatry, Mannheim , Germany
3 University Hospital of the Technical University of Munich , Department of Neurology, Munich , Germany
4 University of Bonn, Institute of Human Genetics , Bonn , Germany
5 University of Bonn School of Medicine & University Hospital Boon , Department of Genomics, Bonn , Germany
6 University Regional Hospital of Málaga , Psychiatry Section, Málaga , Spain
7 University of Málaga , Department of Personality, Assessment And Psychological Treatment, Málaga , Spain
* Corresponding author.
Keywords: major depression, Bipolar disorder, Impulsivity, decision-making and risk behavior, bipolar multiplex families

Conflict of interest:

No

Article ID: EPV0141

Influence of the combined consumption of tobacco and cannabis on the clinical/funtional and cognitive evolution of patients with first manic episodes

Rementeria L. *
García S.
Fernandez J.
González-Ortega I.
Alberich S.
López P.
Zorrilla I.
González-Pinto A.
University Hospital of Alava-Santiago , Department of Psychiatry, Vitoria-Gasteiz , Spain
* Corresponding author.
Keywords: First manic episode, Bipolar disorder, Tobacco, cannabis

Conflict of interest:

No

Article ID: EPV0142

Treatment and response in a spanish sample of children and adolescents with bipolar disorder

Ribeiro-Fernández M. 1 *
Diez-Suarez A. 2 3
1 Complejo Hospitalario de Navarra , Psiquiatría, Pamplona , Spain
2 Clínica Universidad de Navarra , Psychiatry, Pamplons , Spain
3 The University of Texas , Psychiatry And Behavioral Sciences, Houston , United States of America
* Corresponding author.
Keywords: Bipolar disorder, Children, adolescent, treatment

Conflict of interest:

No

Article ID: EPV0143

Treatment and response in a spanish sample of children and adolescents with bipolar disorder

Ribeiro-Fernández M. 1 *
Diez-Suarez A. 2 3
1 Complejo Hospitalario de Navarra , Psiquiatría, Pamplona , Spain
2 Clínica Universidad de Navarra , Psychiatry, Pamplons , Spain
3 The University of Texas , Psychiatry And Behavioral Sciences, Houston , United States of America
* Corresponding author.
Keywords: treatment, Children, adolescent, Bipolar

Conflict of interest:

No

Article ID: EPV0148

Switching from carbolithium to lithium sulphate prolonged-release: differences in subjective experience of psychosocial functioning and quality of life

Tortorelli M. *
Tatini L.
D’Anna G.
Santella M.
Piumelli F.
Cassioli E.
Ballerini A.
University of Florence , Psychiatry, Florence , Italy
* Corresponding author.
Keywords: Bipolar disorder, carbolithium, Lithium Solphate, subjective experience

Conflict of interest:

No

Article ID: EPV0149

Efficacy of functional remediation on cognitive and psychosocial functioning in patients with bipolar disorder: study protocol for a randomized controlled study

Accardo V. 1 2 *
Barlati S. 2 3
Vita A. 2 3
1 University of Brescia , Viale Europa 11, 25123 , Brescia, Italy, Department of Molecular And Translational Medicine, Brescia, Italy
2 University of Brescia School of Medicine , Department of Mental Health And Addiction Services, Asst Spedali Civili , Brescia, Italy., Brescia, Italy
3 University of Brescia, Brescia , Italy ., Department of Clinical and Experimental Sciences, University Of Brescia , Brescia, Italy, Brescia, Italy
* Corresponding author.
Keywords: Bipolar disorder, Cognition, biomarkers, Functional remediation

Conflict of interest:

No

Article ID: EPV0150

Association between borderline personality symptoms and psychiatric pregnancy outcomes in women with bipolar disorder

Casanova Dias M. 1 *
Di Florio A. 1
Kelson M. 2
Jones L. 3
Jones I. 1
1 National Centre for Mental Health School of Medicine Cardiff University , Psychological Medicine and Clinical Neurosciences, Cardiff , United Kingdom
2 Exeter University , College Of Engineering, Mathematics And Physical Sciences, Cardiff , United Kingdom
3 Worcester University , Institute of Health & Society, Worcester , United Kingdom
* Corresponding author.
Keywords: Bipolar disorder, Pregnancy, Borderline Personality Disorder

Conflict of interest:

No

Article ID: EPV0151

Association between borderline personality traits and becoming pregnant in women with bipolar disorder

Casanova Dias M. 1 *
Di Florio A. 2
Kelson M. 3
Jones L. 4
Jones I. 2
1 National Centre for Mental Health School of Medicine Cardiff University , Psychological Medicine, Cardiff , United Kingdom
2 National Centre for Mental Health School of Medicine Cardiff University , Psychological Medicine and Clinical Neurosciences, Cardiff , United Kingdom
3 Exeter University , College Of Engineering, Mathematics And Physical Sciences, Cardiff , United Kingdom
4 Worcester University , Institute of Health & Society, Worcester , United Kingdom
* Corresponding author.
Keywords: Bipolar disorder, Pregnancy

Conflict of interest:

No

Article ID: EPV0153

Seasonality in bipolar disorder: a specifier that needs being specified?

De Toffol M. 1 *
Fico G. 2
Sagué M. 3
Verdolini N. 2
Pacchiarotti I. 4
Solmi M. 5
Vieta E. 6
Murru A. 4
1 University of Padua , Department of Neuroscience, Padova , Italy
2 Bipolar and Depressive Disorders Unit, IDIBAPS , CIBERSAM , Institute of Neuroscience, Hospital Clinic, University Of Barcelona , Barcelona , Spain
3 Hospital Clínic de Barcelona , Addictions Unit. Psychiatry And Psychology Service, Barcelona , Spain
4 Bipolar and Depressive Disorders Unit, Hospital Clínic de Barcelona , Psychiatry, Barcelona , Spain
5 Padova Neuroscience Center, University of Padova , Department of Neurosciences, Padova , Italy
6 University of Barcelona , Department of Psychiatry And Psychology, Barcelona , Spain
* Corresponding author.
Keywords: seasonality, Bipolar disorder

Conflict of interest:

No

Article ID: EPV0156

Characteristics of the early onset bipolar disorder

Guermazi A. *
Omri S.
Smaoui N.
Feki R.
Zouari L.
Charfi N.
Ben Thabet J.
Maalej Bouali M.
Maalej M.
Hedi Chaker University Hospital , Psychiatry C Department, Sfax , Tunisia
* Corresponding author.
Keywords: early onset BD, Bipolar disorder, age at onset

Conflict of interest:

No

Article ID: EPV0157

Impact of antipsychotics on the sexuality of women with bipolar disorder type I

Boudali Z. 1
Zgueb Y. 2
Ouali U. 2
Harrabi Y. 3 *
Jomli R. 3
Nacef F. 3
1 Razi Hospital , Departement "a", Ariana , Tunisia
2 razi hospital, Psychiatry A, manouba, Tunisia
3 Razi Hospital, A "avicenna" Department , Tunis , Tunisia
* Corresponding author.
Keywords: antipsychotics, sexuality, women, bipolar disorder

Conflict of interest:

No

Article ID: EPV0158

Arab-muslim society’s relationship with female sexuality

Boudali Z. 1
Zgueb Y. 1
Ouali U. 1
Harrabi Y. 2 *
Jomli R. 2
Nacef F. 2
1 razi hospital, Psychiatry A, manouba, Tunisia
2 Razi Hospital, A "avicenna" Department , Tunis , Tunisia
* Corresponding author.
Keywords: society, Women, sexuality, arab-muslil countries

Conflict of interest:

No

Article ID: EPV0159

Effects of benzodiazepines on sexuality of women with bipolar disorder type I

Boudali Z. 1
Zgueb Y. 1
Ouali U. 1
Harrabi Y. 2 *
Jomli R. 2
Nacef F. 2
1 razi hospital, Psychiatry A, manouba, Tunisia
2 Razi Hospital, A "avicenna" Department , Tunis , Tunisia
* Corresponding author.
Keywords: benzodiazepines, sexuality, women, bipolar disorder

Conflict of interest:

No

Article ID: EPV0160

Evaluation of the sexual function of women with bipolar disorder type I on the psychotropic-related sexual dysfunction questionnaire scale

Boudali Z. 1
Zgueb Y. 1
Ouali U. 1
Harrabi Y. 2 *
Jomli R. 2
Nacef F. 2
1 razi hospital, Psychiatry A, manouba, Tunisia
2 Razi Hospital, A "avicenna" Department , Tunis , Tunisia
* Corresponding author.
Keywords: Sexual Function, Women, Bipolar disorder;

Conflict of interest:

No

Article ID: EPV0161

Affective temperament and suicide in bipolar disorder: a comparative study

Zgueb Y.
Chakroun M.
Ben Khaled D.
Hechmi S. *
Ouali U.
Nacef F.
Razi University Hospital , Department of Psychiatry A, Mannouba , Tunisia
* Corresponding author.
Keywords: temperament, Bipolar disorder, Suicide, mood

Conflict of interest:

No

Article ID: EPV0162

Suicidal behavior in bipolar disorder type 1

Zgueb Y.
Chakroun M.
Ben Khaled D.
Hechmi S. *
Ouali U.
Nacef F.
Razi University Hospital , Department of Psychiatry A, Mannouba , Tunisia
* Corresponding author.
Keywords: Suicide, Bipolar disorder, suicidal behavior

Conflict of interest:

No

Article ID: EPV0163

The cannabis use as a predictor of suicide attempts in bipolar disorder

Zgueb Y.
Chakroun M.
Ben Khaled D.
Hechmi S. *
Ouali U.
Nacef F.
Razi University Hospital , Department of Psychiatry A, Mannouba , Tunisia
* Corresponding author.
Keywords: cannabis, Bipolar disorder, Suicide

Conflict of interest:

No

Article ID: EPV0164

The clinical aspect of bipolar disorder type 1: about a mediterranean population

Zgueb Y.
Chakroun M.
Ben Khaled D.
Hechmi S. *
Ouali U.
Nacef F.
Razi University Hospital , Department of Psychiatry A, Mannouba , Tunisia
* Corresponding author.
Keywords: Bipolar disorder, Mediterranean, Type 1, Clinical aspect

Conflict of interest:

No

Article ID: EPV0165

Thymoregulators and suicide prevention in bipolar disorder

Zgueb Y.
Chakroun M.
Ben Khaled D.
Hechmi S. *
Ouali U.
Nacef F.
Razi University Hospital , Department of Psychiatry A, Mannouba , Tunisia
* Corresponding author.
Keywords: Bipolar disorder, Suicide prevention, Thymoregulators

Conflict of interest:

No

Article ID: EPV0166

Hyperthyroidism after levothyroxine overdose may induce delayed mixed states in bipolar disorder: a case report and review of recent work

Izquierdo Vendrell E. 1 *
González-Rodríguez A. 2
Sanz N. 2
Cobo J. 2
Salvat N. 3
Feijoo C. 4
Guàrdia Delgado A. 5
Álvarez Pedrero A. 2
Monreal J.A. 6
Palao Vidal D. 7
Labad J. 7
1 Corporació Sanitària Parc Taulí, Mental Health , Sabadell , Spain
2 Parc Taulí University Hospital . Autonomous University of Barcelona (UAB) . I3PT, Mental Health , Sabadell , Spain
3 Corporació Sanitària Parc Taulí. Hospital Universitari de Bellvitge. IDIBELL, Mental Health , Sabadell , Spain
4 Corporació Sanitària Parc Taulí , Internal Medicine, Sabadell , Spain
5 Parc Taulí University Hospital, Mental Health , Sabadell , Spain
6 Parc Taulí University Hospital . Autonomous University of Barcelona (UAB) . I3PT , CIBERSAM, Mental Health , Sabadell , Spain
7 Parc Taulí University Hospital . Autonomous University of Barcelona (UAB) . I3PT. CIBERSAM, Mental Health , Sabadell , Spain
* Corresponding author.
Keywords: Bipolar disorder, Hyperthyroidism, levothyroxine, mixed states

Conflict of interest:

No

Article ID: EPV0169

Family illness perception of bipolar disorders: why we have to take into account family in mental health?

M’Bailara K. 1 2 *
Munuera C. 3
Minois I. 2
Zanouy L. 2
Sportich J. 2
Josse F. 2
Gard S. 2
1 University of Bordeaux , Ea4139, Laboratory Ofpsychology, Bordeaux , France
2 Hospital Charles Perrens , Bipolar Expert Center, Bordeaux , France
3 University of Bordeaux , Laboratory of Psychology, Bordeaux , France
* Corresponding author.
Keywords: illness perception, Bipolar disorder, family

Conflict of interest:

No

Article ID: EPV0171

Bipolar disorder, implications of dimensional perspective

Muntean L. 1 *
Nirestean A. 1 2
Lukacs E. 2
Comaniciu A. 1
1 Mures County Hospital , Psychiatric Clinic No. 2, Targu Mures , Romania
2 George Emil Palade University of Medicine , Pharmacy, Science and Technology of Targu Mures, Psychiatry, Targu Mures , Romania
* Corresponding author.
Keywords: Bipolar disorder, dimensional perspective, personality disorder, insight

Conflict of interest:

No

Article ID: EPV0172

The efficacy of the interpersonal and social rhythm therapy (IPSRT) for patients with bipolar disorder

Palummo C. *
Caivano V.
Zinno F.
Vece A.
Marone L.
Giallonardo V.
Di Cerbo A.
Sampogna G.
Luciano M.
Fiorillo A.
University of Campania "Luigi Vanvitelli" , Department of Psychiatry, Naples , Italy
* Corresponding author.
Keywords: Bipolar disorder, outcome, Interpersonal and Social Rhythm Therapy, group interventions

Conflict of interest:

No

Article ID: EPV0176

Aggressive behaviours and clinical course of bipolar disorder

Caivano V.
Zinno F.
Tarantino G. *
Carfagno M.
Palummo C.
Raia M.
Cristilli G.
Di Cerbo A.
Sampogna G.
Luciano M.
Fiorillo A.
University of Campania "Luigi Vanvitelli" , Department of Psychiatry, Naples , Italy
* Corresponding author.
Keywords: Aggressive behaviours, Bipolar disorder, alcohol abuse, affective temperaments

Conflict of interest:

No

Article ID: EPV0177

Minor physical anomalies in bipolar disorders: a meta-analysis and systematic review

Varga E. *
Hajnal A.
Tenyi T.
Herold R.
University of Pécs , Department of Psychiatry, Pécs , Hungary
* Corresponding author.
Keywords: Méhes scale, Waldrop scale, Bipolar disorder, minor phisical anomalies

Conflict of interest:

No

Article ID: EPV0179

Affective temperaments in patients with bipolar and cyclothymic disorder: a clinical characterization

Zinno F. 1 *
Sampogna G. 1
Del Vecchio V. 1
Giallonardo V. 1
Luciano M. 1
Steardo L. 2
Perugi G. 3
Pompili M. 4
Tortorella A. 5
Volpe U. 6
Fiorillo A. 1
1 University of Campania "L. Vanvitelli" , Department of Psychiatry, Naples , Italy
2 University "Magna Graecia" of Catanzaro , Psychiatry Unit, Catanzaro , Italy
3 University of Pisa, Azienda Ospedaliera Universitaria Pisana (AOUP) , Department of Clinical and Experimental Medicine, Psychiatric Section, Pisa , Italy
4 S. Andrea Hospital, Sapienza University , Department of Neurosciences, Mental Health And Sensory Organs , Suicide Prevention Center, Rome , Italy
5 University of Perugia , Department of Medicine, Division Of Psychiatry, Perugia , Italy
6 School of Medicine Università Politecnica delle Marche , Department of Neurosciences/dimsc, Ancona , Italy
* Corresponding author.
Keywords: affective temperaments, Bipolar disorder, cyclothymic disorder

Conflict of interest:

No

Article ID: EPV0182
Subjects: Abstract, Child and Adolescent Psychiatry

Awareness of attention deficit hyperactivity disorder among special education students in riyadh and qassim regions of saudi arabia 2019-2020

Alotaibi S. 1
Alsoqih N. 2
Aljhani S. 3 *
Alshubrmi A. 1
Alshawi A. 1
Abdullah H. 1
Almushaity R. 1
Alkuraidess S. 1
1 Qassim university, Medical College , Qassim , Saudi Arabia
2 Qassim university , Pediatric Neurology, Qassim , Saudi Arabia
3 Qassim university , Psychiatry, Qassim , Saudi Arabia
* Corresponding author.
Keywords: Autism, awarness, Neurodevelopmental

Conflict of interest:

No

Article ID: EPV0184

Separation anxiety - how to help to child and to a parent case report

Barac-Otasevic Z. *
Ljutica I.
Medical Clinical Centre Montenegro , Podgorica , Psychiatry Clinic , Podgorica , Montenegro
* Corresponding author.
Keywords: Anxiety, child, separation, parents

Conflict of interest:

No

Article ID: EPV0186

Construction and analysis of psychological anamnesis as basis for diagnostics of children and adolescents in psychiatric clinic

Burlakova N. 1 *
Oleshkevich V. 2
1 Lomonosov Moscow State University , Faculty of Psychology, Department of Neuro- And Pathopsychology, Moscow , Russian Federation
2 Scientific Practical Children’s Mental Health Centre n. a. G. Sukhareva of Moscow City Department of Healthcare, -, Moscow , Russian Federation
* Corresponding author.
Keywords: psychological diagnostics, psychological anamnesis

Conflict of interest:

No

Article ID: EPV0192

Adhd and emotional dysregulation

Chinchurreta N. 1 *
De Frutos Guijarro J.J. 2
Martín Aragón R. 3
Moreno Menguiano C. 2
1 HOSPITAL PUERTA DE HIERRO , Psychiatry, MADRID , Spain
2 Centro de Salud Doctor Luengo Rodriguez , Servicio De Psiquiatría, Mostoles , Spain
3 Complejo Hospitalario Mancha Centro ., Unidad De Salud Mental Infanto-juvenil ., Alcázar de San Juan ., Spain
* Corresponding author.
Keywords: ADHD, emotional regulation

Conflict of interest:

No

Article ID: EPV0193

Function of self-harm in adolescents. about a case

Chinchurreta N. 1 *
De Frutos Guijarro J.J. 2
Martín Aragón R. 3
Moreno Menguiano C. 2
De Cós Milas A. 4
1 HOSPITAL PUERTA DE HIERRO , Psychiatry, MADRID , Spain
2 Centro de Salud Doctor Luengo Rodriguez , Servicio De Psiquiatría, Mostoles , Spain
3 Complejo Hospitalario Mancha Centro ., Unidad De Salud Mental Infanto-juvenil ., Alcázar de San Juan., Spain
4 HOSPITAL UNIVERSITARIO DE MÓSTOLES , Psychiatry, MADRID , Spain
* Corresponding author.

Conflict of interest:

No

Article ID: EPV0194

Mood disorder and hypothyroidism in adolescents. a case report

Chinchurreta N. 1 *
Martín Aragón R. 2
De Frutos Guijarro J.J. 3
Martin Carballeda J. 4
De Cós Milas A. 5
1 HOSPITAL PUERTA DE HIERRO , Psychiatry, MADRID , Spain
2 Complejo Hospitalario Mancha Centro., Unidad De Salud Mental Infanto-juvenil., Alcázar de San Juan., Spain
3 Centro de Salud Doctor Luengo Rodriguez, Servicio De Psiquiatría, Mostoles , Spain
4 Centro salud mental Alcorcon, Unidad De Salud Mental Infanto-juvenil., Alcorcon , Spain
5 HOSPITAL UNIVERSITARIO DE MÓSTOLES , Psychiatry, MADRID , Spain
* Corresponding author.
Keywords: hypothyroidism, mood, disorder

Conflict of interest:

No

Article ID: EPV0197

A case of 16p11.2 duplications and deletions as a risk factor to autism

De La Mata Hidalgo M. 1 *
De La Mata R. 2
Tenorio R. 1
Pacheco M. 1
Mota M. 1
Anmella G. 3
Suárez M. 1
1 Hospital Juan Ramón Jiménez , Psiquiatría, Huelva , Spain
2 Centro Salud Mental Infanto Juvenil, Psychiatry, Barcelona , Spain
3 Hospital Clinic, University of Barcelona, IDIBAPS, CIBERSAM, Schizophrenia Unit, Institute of Neuroscience , Barcelona , Spain
* Corresponding author.
Keywords: 16p11.2, microdelection, arrayCGH, Autism

Conflict of interest:

No

Article ID: EPV0200

Anxiety/depression has a strong impact on attention to eye region in adolescents with autism spectrum disorder

Fujioka T. 1 *
Tsuchiya K. 2
Katayama T. 3
Kosaka H. 4
1 University of Fukui , Department of Science Of Human Development, Humanities And Social Science, Faculty of Education,, fukui, Japan
2 Hamamatsu University School of Medicine , Research Center for Child Mental Development, Hamamatsu , Japan
3 Osaka Univrersity , Department of Child Development, Osaka , Japan
4 University of Fukui , Department of Neuropsychiatry, Fukui , Japan
* Corresponding author.
Keywords: Autism Spectrum Disorder, eye-tracking, Dépression, Anxiety

Conflict of interest:

No

Article ID: EPV0201

"My mother was very sad when i was born." psychopathology in sons of patients with severe mental disorder. Case report

Del Sol Calderón P. *
Izquierdo De La Puente Á.
Garcia Moreno M.
HOSPITAL UNIVERSITARIO PUERTA DE HIERRO MAJADAHONDA , Psychiatry, MADRID , Spain
* Corresponding author.
Keywords: ADHD, attachment disorder, child and adolescent, parent´s psychopathology

Conflict of interest:

No

Article ID: EPV0203

Group therapy experiences in adolescents with autism spectrum disorder

Garcia Murillo L. *
Forti Buratti A.
Díaz De Neira M.
Palanca Maresca I.
Hospital Universitario Puerta de Hierro , Child And Adolescent Psychiatry, Majadahonda, Madrid , Spain
* Corresponding author.
Keywords: group therapy, adolescents, Autism, social skills

Conflict of interest:

No

Article ID: EPV0204

Subjective experiences and quality of life in siblings of children with intellectual disabilities: a review of literature

Gnanavel S.
Health Education North East England , Child And Adolescent Psychiatry, Newcastle upon Tyne, United Kingdom
Keywords: siblings, QOL, learning disability

Conflict of interest:

No

Article ID: EPV0205

Spatial representation in children with mild mental retardation

Goryacheva T. *
Makarova O.
Pirogov Russian National Research Medical University , Psychological-social Faculty, Moscow , Russian Federation
* Corresponding author.
Keywords: mental retardation, spatial representations

Conflict of interest:

No

Article ID: EPV0206

The characteristics of visual attention of the students of the elementary school with ADHD

Goryacheva T. *
Belousov A.
Pirogov Russian National Research Medical University , Psychological-social Faculty, Moscow , Russian Federation
* Corresponding author.
Keywords: visual attention, ADHD

Conflict of interest:

No

Article ID: EPV0207

Agression and impulsivity in play therapy: therapeutic challenges and limits

Gungor M. 1 *
Calli S. 2
Arslandogdu S. 2
Yavuz M. 3
1 ISTANBUL AYDIN UNİVERSİTY, FRENCH LAPE HOSPİTAL , Psychology, Child And Adolescent Psychiatry, Küçükçekmece , Turkey
2 Istanbul Aydin University , Psychology Department, Küçükçekmece , Turkey
3 Istanbul Aydin University, French Lape Hospital, Psychology, Child And Adolescent Psychiatry , Küçükçekmece , Turkey
* Corresponding author.
Keywords: Play therapy, agression, limits, Children

Conflict of interest:

No

Article ID: EPV0212

The zone of proximal development in children with specific developmental disorders

Ivanov M.
Moscow Institute of Psychoanalysis , Department of Child And Adolescent Clinical Psychology, Moscow , Russian Federation
Keywords: Children, specific developmental disorders, zone of proximal development, Lev S. Vygotsky

Conflict of interest:

No

Article ID: EPV0213

Deprivation factors and mental health in early childhood

Ivanov M. *
Margolina I.
Platonova N.
Federal State Budgetary Scientific Institution "Mental Health Research Centre" , Department of Child Psychiatry, Moscow , Russian Federation
* Corresponding author.
Keywords: orphans, deprivation, physical abuse, sexual abuse

Conflict of interest:

No

Article ID: EPV0214

Suicide attempts in adolescents: a case report

Izquierdo De La Puente Á. 1 *
Del Sol Calderón P. 1
Garcia Moreno M. 1
Vizcaíno Da Silva M. 1
Fernández Fernández R. 2
Mendez Gonzalez O. 1
Rodríguez Rodríguez A. 1
Blanco R. 1
Martín García M. 3
González-Villalobos Rincón I. 1
1 HOSPITAL UNIVERSITARIO PUERTA DE HIERRO MAJADAHONDA , Psychiatry, MADRID , Spain
2 Hospital Universitario HM Puerta del Sur , Psychiatry, Mostoles , Spain
3 Hospital Universitario Puerta de Hierro , Majadahonda, Madrid , Spain
* Corresponding author.

Conflict of interest:

No

Article ID: EPV0215

Kleine lein syndrome: a case report

Izquierdo De La Puente Á. *
Perteguer R.
Del Sol Calderón P.
Garcia Moreno M.
HOSPITAL UNIVERSITARIO PUERTA DE HIERRO MAJADAHONDA , Psychiatry, MADRID , Spain
* Corresponding author.
Keywords: teenagers, Kleine Lein Syndrome

Conflict of interest:

No

Article ID: EPV0218

PTSD symptoms in adolescents from low- and middle-income countries: international child mental health - study group

Stupar D. 1
Stevanovic D. 1
Doric A. 2
Knez R. 3 *
1 Clinic for Neurology and Psychiatry for Children and Youth, Department of Child Psychiatry, Belgrade , Serbia
2 Faculty of Humanities and Social Sciences, Department of Psychology, Rijeka , Croatia
3 University of Gothenburg , Department of Psychiatry And Neurochemistry, Gothenburg , Sweden
* Corresponding author.
Keywords: UCLA PTSD index, Culture, traumatic events, prevalence

Conflict of interest:

No

Article ID: EPV0219

Disorders of adaptation in children from forcibly displaced families

Kozhyna H. *
Samoylova O.
Zelenska K.
Radchenko T.
Koshchii V.
KHARKIV NATIONAL MEDICAL UNIVERSITY , Psychiatry, Narcology And Medical Psychology, Kharkiv , Ukraine
* Corresponding author.
Keywords: children from forcibly displaced families, adaptation disorders, psycho-traumatic factor, post-traumatic stress disorder

Conflict of interest:

No

Article ID: EPV0220

A brief paradigm to evaluate mentalization and theory of mind, in preschool children

Lascarez Martínez S. *
Flores Lázaro J.
Universidad Nacional Autónoma de México , Neurociencias, México , Mexico
* Corresponding author.
Keywords: preschool children, paradigm, mentalization, theory of mind

Conflict of interest:

No

Article ID: EPV0225

Child sexual abuse and revictimization at law courts: neuroendocrine effects.

Martorella A.M.
Hospital Materno Mar del Plata, Mental Health, Mar del Plata, Argentina
Keywords: child sexual abuse, revictimization, post traumatic disorder, neuroendocrine effects

Conflict of interest:

No

Article ID: EPV0229

Psychopathology in fragile X syndrome. Experience in a genetic disorders unit from a children and adolescent mental health center.

Oscoz Irurozqui M. 1 *
Urraca Camps L. 2
Pujol Serra S. 2
Colomina Llobell C. 2
Pàmias Massana M. 2
Palao Vidal D. 2
1 Benito Menni CASM , Psychiatry, Sant Boi de Llobregat, Spain
2 Parc Tauli-University Hospital, Mental Health , Sabadell ( Barcelona ), Spain
* Corresponding author.
Keywords: fragile x syndrome, psycopathology, GENETIC DISORDERS, Children

Conflict of interest:

No

Article ID: EPV0231

“How long does it take for antipsychotic to get out of my child system?”- Discontinuation of antipsychotic in children.

Patel R.
County of San bernadino, Mental Health , Corona , United States of America
Keywords: Antipsychotic medication, Discontinuation of Antipsychotic medication, Withdrawal dyskinesia, switching of medication

Conflict of interest:

No

Article ID: EPV0232

Parental practices implemented in adolescents in the city of santa marta, colombia

Miranda L.F. 1
Correa K. Pérez 2 *
Álvarez L. Pedraza 3
Salas Picón W. 2
1 Universidad del Magdalena , Facultad De Educación, Santa Marta, Colombia
2 Universidad Cooperativa de Colombia , Facultad De Psicología, Santa Marta, Colombia
3 Universidad del Magdalena , Facultad De Ciencias De La Salud, Santa Marta, Colombia
* Corresponding author.
Keywords: Parental practices, prosocial behavior, aggressive behavior

Conflict of interest:

No

Article ID: EPV0233

Prosocial and aggressive behaviors in adolescents in the city of santa marta, colombia

Miranda L.F. 1
Pérez Correa K. 2 *
Viloria Escobar J. 3
Rodríguez Pacheco F. 3
1 Universidad del Magdalena , Facultad De Educación, Santa Marta, Colombia
2 Universidad Cooperativa de Colombia , Facultad De Psicología, Santa Marta, Colombia
3 Universidad del Magdalena , Facultad De Ciencias De La Educación, Santa Marta, Colombia
* Corresponding author.
Keywords: Parental practices, prosocial behavior, aggressive behavior

Conflict of interest:

No

Article ID: EPV0234

Approach through stories of emotional regulation to anxiety in a sample of primary school students.

Pérez Sánchez S. 1 *
Martín Herrero I. 1
Crespo Portero A. 2
Güimil Raya D. 1
1 Morales Meseguer Public University Hospital , Psychiatry, Murcia , Spain
2 Lorca Mental health Center , Psychiatry, Lorca , Spain
* Corresponding author.
Keywords: stories, emotional regulation, anxiety, primary school students.

Conflict of interest:

No

Article ID: EPV0235

“Why do i have this pain? What if it is a serious disease?” – A reflection about somatic symptoms in children and adolescents

Pinto M. *
Lobato De Sousa M.J.
Almeida M.G.
Maia C.
Centro Hospitalar do Tâmega e Sousa, Child And Adolescent Psychiatry, Guilhufe , Portugal
* Corresponding author.
Keywords: somatization, somatic symptom disorder, Anxiety, Children and adolescents

Conflict of interest:

No

Article ID: EPV0236

Antipsychotic and mood stabilizer treatments complete slow removal in a severe mental illness young adult diagnosed in adolescence.

Ponte-López T. 1 *
Herranz-Herrer J. 1
Caballero Martínez L. 2
Gil-Benito E. 1
Estevez-Peña B. 1
Pérez-Balaguer A. 3
Lobato M.J. 1
Martín García M. 1
1 Hospital Universitario Puerta de Hierro , Majadahonda, Madrid , Spain
2 Hospital Puerta de Hierro , Psychiatry, Madrid , Spain
3 Hospital Puerta de Hierro de Majadahonda , Majadahonda, Madrid , Spain
* Corresponding author.
Keywords: Bipolar disorder, borderline personality, psychosis, dissociative

Conflict of interest:

No

Article ID: EPV0239

Current situation of chilean education and a way to prevent desertion and delinquency based on mental health

Rojas J.
Hospital de Niños Dr. Luis Calvo Mackenna , Psychiatry, Providencia , Chile
Keywords: child, education, Motivation, delinquency

Conflict of interest:

No

Article ID: EPV0240

Dietary and psychological characteristics of obese japanese children.

Saito M. 1 *
Lefor A. 2
Saito S. 2
Kikuchi Y. 1
1 Haga Red Cross Hospital , Pediatrics , Mouka , Japan
2 Jichi Medical University , Surgery, Shimotsuke , Japan
* Corresponding author.
Keywords: obesity, Japanese, self-esteem, Children

Conflict of interest:

No

Article ID: EPV0244

Pharmacogenetics of short-term antipsychotics safety in adolescents with acute psychotic episode: CYP2D6 and ABCB1 polymorphisms do matter

Tazagulova M. 1 *
Ivashchenko D. 2
Buromskaya N. 3
Shimanov P. 4
Deitch R. 5
Akmalova K. 6
Grishina E. 6
Shevchenko Y. 2
Sychev D. 7
1 Russian Medical Academy of Continuous Professional Education , Moscow, Russia, Child And Adolescent Psychiatry And Psychotherapy, Moscow , Russian Federation
2 Russian Medical Academy of Continuous Professional Education , Child Psychiatry And Psychotherapy, Moscow , Russian Federation
3 G.E. Sukhareva Research Practical Centre of Children and Adolescents Mental Health, No 1, Moscow , Russian Federation
4 G.E. Sukhareva Research Practical Centre of Children and Adolescents Mental Health, No 7, Moscow , Russian Federation
5 G.E. Sukhareva Research Practical Centre of Children and Adolescents Mental Health, No 3, Moscow , Russian Federation
6 Russian Medical Academy of Continuous Professional Education , Molecular Medicine, Moscow , Russian Federation
7 Russian Medical Academy of Continuous Professional Education , Clinical Pharmacology And Therapeutics, Moscow , Russian Federation
* Corresponding author.
Keywords: antipsychotics, adolescents, acute psychotic episode, pharmacogenetics

Article ID: EPV0245

Prevalence of attention deficit and hyperactivity disorder in adolescents attending school

Morel S.
Tejada N. *
Martinez N.
Valdez A.
Pontificia Universidad Catolica Madre y Maestra, Escuela De Medicina , Santiago , Dominican Republic
* Corresponding author.
Keywords: adolescent, attention-deficit hyperactivity disorder, prevalence

Conflict of interest:

No

Article ID: EPV0247

Treatment-resistant schizophrenia in adolescence: a case report.

Torres Varona F.J. 1 *
Rodríguez Criado N. 2
1 Hospital Central de la Defensa Gómez Ulla, Psychiatry And Mental Health Department , Madrid , Spain
2 Hospital Infantil Universitario Niño Jesús , Child And Adolescent Psychiatry And Psychology Department, Madrid , Spain
* Corresponding author.
Keywords: early-onset schizophrenia, treatment-resistant schizophrenia, clozapine, adolescent

Conflict of interest:

No

Article ID: EPV0249

Comparison of attitudinal aspects of body image in adolescents with self-reported nssi and body modifications

Ryzhov A. 1 *
Mamedova K. 2
Sokolova E. 1
Pechnikova L. 1
Zhuykova E. 3
1 Lomonosov MSU , Faculty of Psychology, Moscow , Russian Federation
2 Lomonosov MSU , Baku Branch, Baku , Azerbaijan
3 Psychological Institute of the Russian Academy of Education , Adolescent Psychology Laboratory, Moscow , Russian Federation
* Corresponding author.
Keywords: hopelessness, NSSI, body modifications, body image

Conflict of interest:

No

Article ID: EPV0250

Variability in the final subtype of bipolar disorder in children and adolescents in a spanish sample

Ribeiro-Fernández M. 1 *
Diez-Suarez A. 2 3
1 Complejo Hospitalario de Navarra , Psiquiatría, Pamplona , Spain
2 Clínica Universidad de Navarra , Psychiatry, Pamplona , Spain
3 The University of Texas , Psychiatry And Behavioral Sciences, Houston , United States of America
* Corresponding author.
Keywords: Subtype, Bipolar disorder, Children, adolescent

Conflict of interest:

No

Article ID: EPV0257

Depressive disorder in children and adolescents: comorbidities and therapeutic management: about 40 cases

Bamaarouf W.
Hospital ARAZZI SALE , Psychiatry, SALE , Morocco
Keywords: médication, Dépression, Children, adolescent

Conflict of interest:

No

Article ID: EPV0258

Parent screening during child assessment: exploring child and parent psychopathology in child/adolescent clinic.

Bani M. 1 *
Allkoja B. 2
Petrela E. 3
1 University Hospital Center “Mother Teresa” , Tirana, Albania ., Department of Neuroscience,, tirana , Albania
2 HEALTH COMMUNITY CENTER NR.1 , Child/adolescent Psychiatry, TIRANA , Albania
3 University Hospital Center “Mother Teresa” , Tirana, Albania ., Department of Statistics, tirana , Albania
* Corresponding author.
Keywords: child psychiatric evaluation, parent screening

Conflict of interest:

No

Article ID: EPV0260

Outcome predictors in a low-intensity early start denver model implemented in the taiwanese public health system

Chiang C.-H. *
Lin T.L.
National Chengchi University , Psychology, Taipei , Taiwan
* Corresponding author.
Keywords: Autism, Early Start Denver Model, Outcome Predictors, Comprehensive Early Intervention

Conflict of interest:

No

Article ID: EPV0261

Dating violence in adolescents

Cohen D.S. 1 *
Muñoz Domenjó A. 2
Muñoz-Calero P. 3
Gadea Del Castillo S. 1
Herrero Escudero D. 1
Rybak Koite E.M. 1
Rodríguez Criado N. 4
Mesián Pérez I. 4
1 Complejo Asistencial de Segovia , Servicio De Psiquiatría, Segovia , Spain
2 Hospital Universitario de Móstoles , Psiquiatría, Móstoles, Madrid , Spain
3 Hospital Universitario de Móstoles , Psychiatry, MADRID , Spain
4 Hospital Infantil Universitario Niño Jesús , Servicio De Psiquiatría Y Psicología Del Niño Y El Adolescente, Madrid , Spain
* Corresponding author.
Keywords: dating violence, Intimate partner violence, adolescent

Conflict of interest:

No

Article ID: EPV0264

Updating ASD and comorbid eating disorders: strategies for treatment and management.

Dominguez Ballesteros E. 1 *
Santamaría Rubio E. 1
Sanchez Vicente M. 2
1 Clinica Lopez Ibor , Child Psychiatry, Madrid , Spain
2 Quirón Salud , Accident And Emergency Department, Madrid , Spain
* Corresponding author.
Keywords: ASD, eating disorders, comorbidity, Child Psychiatry

Conflict of interest:

No

Article ID: EPV0267

Improving mental health care service in children and adolescents with e-poster viewing: intellectual disability

Insa Pineda I. *
Chamorro Fernández M.
Huguet Miguel A.
Gómez González C.L.
Ventura Mallofré E.
Hospital Sant Joan de Déu of Barcelona, Child And Adolescent Psychiatry And Psychology Department, Esplugues de Llobregat, Barcelona , Spain
* Corresponding author.
Keywords: special education school, Intellectual disability, Caregivers, Burnout

Conflict of interest:

No

Article ID: EPV0269

Effects of dance movement therapy on social engagement in young children with autism spectrum disorder

Lee T.-C. 1 *
Su Y.-C. 2
Chiang C.-H. 2
1 National Chengchi University , Education, Taipei , Taiwan
2 National Chengchi University , Psychology, Taipei , Taiwan
* Corresponding author.
Keywords: dance movement therapy, Autism, joint engagement, affect attunement

Conflict of interest:

No

Article ID: EPV0270

Improving policy and practice in the interface between child and adolescence psychiatry and adult psychiatry services: a portuguese articulation project

Maia A. 1 2 3 *
Couto D. 3
Gago J. 1 3
Maia G. 1 3
Sardinha L. 1 3
1 Universidade Nova de Lisboa , Portugal, Nova Medical School | Faculdade De Ciências Médicas, Lisbon , Portugal
2 Champalimaud Centre for the Unknown, Champalimaud Clinical Centre, Lisbon , Portugal
3 Centro Hospitalar de Lisboa Ocidental, Psychiatry And Mental Health, Lisbon , Portugal
* Corresponding author.
Keywords: Transition into adulthood, COPMI, Articulation project, quality of care

Conflict of interest:

No

Article ID: EPV0272

Sleep disturbances in unmedicated children recently diagnosed with attention-deficit/hyperactivity disorder assessed by actigraphy and parent-report questionnaires

Marta C.F. *
Imma I.P.
Cristina G.G.
Ja A.D.
Sant Joan de Deu Hospital, Child/youth Mental Health Centre, Barcelona , Spain
* Corresponding author.
Keywords: Newly diagnosed ADHD, comorbidity, Children, sleep disorders

Conflict of interest:

No

Article ID: EPV0273

Clinical phenotype of autism spectrum disorders in children of preschool and school age, burdened epileptic seizures.

Martsenkovsky I. *
Skrypnyk T.
Research Institute of Psychiatry of the Ministry of Health of Ukraine , Department of Mental Disorders Of Children And Adolescents, Kyiv , Ukraine
* Corresponding author.
Keywords: autism, epileptic seizures, motor and vocal tics

Conflict of interest:

No

Article ID: EPV0274

Efficacy and safety of levetiracetam and risperidone for aggression irritability, and hyperactivity in adolescent with autism spectrum disorder

Martsenkovsky I. *
Makarenko G.
Martsenkovska I.
Research Institute of Psychiatry of the Ministry of Health of Ukraine , Department of Mental Disorders Of Children And Adolescents, Kyiv , Ukraine
* Corresponding author.
Keywords: Efficacy, safety, levetiracetam, risperidone

Conflict of interest:

No

Article ID: EPV0275

Quality improvement project in ADHD treatment pathway under merton child and adolescent mental health service (CAMHS)

Nagasinghe P.
SOUTHWEST LONDON & ST GEORGE'S MENTAL HEALTH NHS TRUST, Neurodevelopment Services, TOOTING , United Kingdom
Keywords: ADHD Medication, Treatment pathway

Conflict of interest:

No

Article ID: EPV0276

Functional NIRS evaluation of prefrontal cortex activity during emotional processing in children with ADHD

Maddalena M. 1
Grazioli S. 2
Crippa A. 2
Bacchetta A. 2
Maggioni E. 3
Brambilla P. 4
Diwadkar V. 5
Gatti E. 2
Bertella S. 2
Molteni M. 2
Nobile M. 2 *
1 University of Milano-Bicocca , Phd Program In Neuroscience, School of Medicine and Surgery, Milan , Italy
2 Scientific Institute 'Medea' , Child Psychopathology Unit, Bosisio Parini, Italy
3 Foundation IRCCS Ca’ Granda Ospedale Maggiore Policlinico , Department of Neurosciences And Mental Health, Milan , Italy
4 University of Milan , Department of Pathophysiology And Transplantation, Milan , Italy
5 Wayne State University School of Medicine , Departments Of Psychiatry Andbehavioral Neurosciences,, DETROIT, United States of America
* Corresponding author.
Keywords: emotional regulation, ADHD, Prefrontal Cortex, fNIRS

Conflict of interest:

No

Article ID: EPV0277

Features of the interrelations of existential characteristics and depressive symptoms of adolescents with autoaggressive behavior

Grigorieva A. 1
Osipova B. 2 *
Nedelko D. 2
Usova L. 3
Gavrichenkova A. 3
1 National Medical Research Center of Psychiatry and Addiction, Ministry of Health Care , Russia, Department of Organization Of The Prevention Aid In Addiction, Moscow , Russian Federation
2 Moscow state University of Psychology and Education , Legal And Forensic Psychology, Moscow , Russian Federation
3 National Medical Research Center of Psychiatry and Addiction, Department of Organization Of The Prevention Aid In Addiction, Moscow , Russian Federation
* Corresponding author.
Keywords: Self-harm, adolescence, Dépression, autoagressive

Conflict of interest:

No

Article ID: EPV0278

Support program for the carieer of attention deficit hyperactivity disorder: a university extension project

Paiva Wagner C.J. *
Kurmann A.C.
Viapiana V.
Santos De Morais M.
Rebechi C.
Glimm D.
Universidade de Passo Fundo , Faculdade De Medicina, Passo Fundo, Brazil
* Corresponding author.
Keywords: child and adolescent psychiatry, Attention Deficit Hyperactivity Disorder, University extension project

Conflict of interest:

No

Article ID: EPV0280

Nutrition in neurodevelopmental disorders: to supplement or not to supplement, that is the question!

Sa-Carneiro F. 1 *
Coelho R. 1
Calhau C. 2
Figueiredo-Braga M. 3
1 Centro Hospitalar Universitário de São João , Psychiatry, Porto , Portugal
2 Faculty of Medicine University of Porto, Center for Health Technology And Services Research (cintesis), Porto , Portugal
3 Faculty of Medicine University of Porto, Department of Neurosciences And Mental Health, Porto , Portugal
* Corresponding author.
Keywords: Autism Spectrum Disorder, Attention Deficit Hyperactivity Disorder, nutritional deficits, supplementation

Conflict of interest:

No

Article ID: EPV0283
Subjects: Abstract, Classification of Mental Disorders

Perspectives of latin version of ICD-11. Tradition and modern trends in the newest diagnostic categories of mental disorders

Kosmowski W. 1 *
Jóskowska K. 2
1 Nicolaus Copernicus University , Department of Psychiatry, Bydgoszcz , Poland
2 Nicolaus Copernicus University , Collegium Medicum, Department of Applied Linguistics, Bydgoszcz , Poland
* Corresponding author.
Keywords: Latin, ICD-11, classification

Conflict of interest:

No

Article ID: EPV0286

Misophonia: considering a new mental disorder.

Ezquerra B. *
Esteban M.
Carpintero A.
Lopez C.
Sanchez M.
Del Hoyo Mitjans R.
Hospital Universitario Guadalajara , Psychiatry, Guadalajara , Spain
* Corresponding author.
Keywords: Misophonia

Conflict of interest:

No

Article ID: EPV0288

Diagnostic conversion of schizoaffective disorder: a retrospective study

Ferreira T. *
Dehanov S.
Figueiredo I.
Dias M.
Borja Santos N.
Maia T.
Hospital Prof. Doutor Fernando Fonseca, Psychiatry Department, Amadora , Portugal
* Corresponding author.
Keywords: Schizoaffective disorder, psychosis, Mood disorders, Diagnostic Conversion

Conflict of interest:

No

Article ID: EPV0290
Subjects: Abstract, Comorbidity / Dual Pathologies

Relationship between cannabis use and affective disorders: a review of the evidence.

Arribas Pinero E. *
León-Quismondo L.
RAMON Y CAJAL UNIVERSITY HOSPITAL, Psychiatry, MADRID , Spain
* Corresponding author.
Keywords: cannabis, Depressive disorder, risk factor, affective disorders

Conflict of interest:

No

Article ID: EPV0291

Cannabis use as a risk factor for psychosis: a review.

Arribas Pinero E. *
León-Quismondo L.
RAMON Y CAJAL UNIVERSITY HOSPITAL, Psychiatry, MADRID , Spain
* Corresponding author.
Keywords: schizophrenia, cannabis, risk factor, psychosis

Conflict of interest:

No

Article ID: EPV0292

Psychosomatic aspects of gastric ulcer disease and hypothyroidism combination

Artemieva M. *
Kuznetsov V.
Basova E.
Manyakin I.
Danilin I.
Peoples' Friendship University of Russia (RUDN University), Department of Psychiatry And Medical Psychology, Moscow , Russian Federation
* Corresponding author.
Keywords: Gastric Ulcer Disease, psychosomatics

Conflict of interest:

No

Article ID: EPV0293

The quality of life of patients with gastroesophageal reflux disease

Artemieva M. *
Kuznetsov V.
Manyakin I.
Basova E.
Lazukova A.
Peoples' Friendship University of Russia (RUDN University), Department of Psychiatry And Medical Psychology, Moscow , Russian Federation
* Corresponding author.
Keywords: eating disorders, GERD

Conflict of interest:

No

Article ID: EPV0294

Multiple sclerosis and the impact of the association of psychiatric pathology

Darie C. 1 *
Mutica M. 2
Peterson I. 3
Ciubara A. 4
1 Resident Psychiatrist at Psychiatric Hospital ''Elisabeta Doamna'', Psychiatry, GALATI , Romania
2 PhD "Dunarea de Jos" University of Galați, Psychiatry, Galati , Romania
3 PhD Student, Psychiatric Clinic Ryhov, Psychiatry, Ryhov , Sweden
4 Professor at "Dunarea de Jos" University, Senior Psychiatrist at ''Elena Doamna'' Psychiatric Hospital, Psychiatry, Galati , Romania
* Corresponding author.
Keywords: multiple sclerosis, Psychiatry, Dépression, Anxiety

Conflict of interest:

No

Article ID: EPV0295

Impact of psoriasis, depression and alcohol related disorder on quality of life

Darie C. 1 *
Stoleriu G. 2
Terpan M. 3
Oltenacu R.-C. 3
Ciubara A. 4
1 Resident Psychiatrist at Psychiatric Hospital ''Elisabeta Doamna'', Psychiatry, GALATI , Romania
2 PhD, Assos. Professor "Dunarea de Jos" University, Psychiatry, Galati , Romania
3 PhD Student "Dunarea de Jos" University, Psychiatry, Galati , Romania
4 Professor at "Dunarea de Jos" University, Senior Psychiatrist at ''Elena Doamna'' Psychiatric Hospital, Psychiatry, Galati , Romania
* Corresponding author.
Keywords: Psychiatry, dermatology, Dépression, psoriasis

Conflict of interest:

No

Article ID: EPV0296

Eating disorders and disorders due to substance use comorbidity etiopathogenesis.

De Frutos Guijarro J.J. 1 *
Martín Aragón R. 2
Chinchurreta N. 3
Saez Roche M.E. 1
Calleja Alonso P. 4
Garcia-Consuegra Colado D. 4
Moreno Menguiano C. 1
1 Centro de Salud Doctor Luengo Rodriguez, Servicio De Psiquiatría, Mostoles , Spain
2 Complejo Hospitalario Mancha Centro ., Unidad De Salud Mental Infanto-juvenil., Alcázar de San Juan., Spain
3 HOSPITAL PUERTA DE HIERRO , Psychiatry, MADRID , Spain
4 Centro de Salud Coronel de Palma, Unidad De Trastornos De Conducta Alimentaria , Mostoles , Spain
* Corresponding author.
Keywords: eating disorders, substance use, comorbidity, etiopathogenesis

Conflict of interest:

No

Article ID: EPV0297

Myristica fragrans abuse and mania, a case report

Fernandez Gomez N. 1 *
Bravo-Ortiz M.F. 2
Bardón Rivera B. 2
Marín Lozano J. 2
Román Mazuecos E.M. 2
1 Hospital Universitario La Paz , Psiquiatría Y Salud Mental, Madrid , Spain
2 La Paz University Hospital Research Health Institute (IdiPAZ) , Psychiatry, Clinical Psychology And Mental Health, Madrid , Spain
* Corresponding author.
Keywords: mania, myristicafragans, substanceabuse, stimulant

Conflict of interest:

No

Article ID: EPV0300

The importance of screening for bipolar disorder in patients treated for substance use disorders

Jonovska S. *
Sendula Jengic V.
Psychiatric Hospital Rab , Department of Addictions, Rab , Croatia
* Corresponding author.
Keywords: Screening, Addiction, treatment, Bipolar disorder

Conflict of interest:

No

Article ID: EPV0302

Dysfunctional breathing as interdisciplinary phenomenon

Koniukhovskaia J. *
Pervichko E.
Lomonosov Moscow State University , Faculty of Psychology, Moscow , Russian Federation
* Corresponding author.
Keywords: dysfunctional breathing, panic disorder, PTSD, anxiety disorder

Conflict of interest:

No

Article ID: EPV0309

Subjective anxiety according to comorbidity in dual pathology

Pino Calderon F.J. 1 *
Lozano Gomez V. 2
Zoido Ramos J. 3
Gutierrez Casares J.R. 4
1 Servicio Extremeño de Salud, Equipo De Salud Mental De Montijo, Montijo , Spain
2 Servicio Extremeño de Salud, Psychiatry Unit - Hospital Universitario De Badajoz, Badajoz , Spain
3 Servico Extremeño de Salud, Cedex Los Pinos, Badajoz , Spain
4 Servicio Extremeño de Salud, Esm Psiquiatría Infantil - Hospital Perpetuo Socorro, Badajoz , Spain
* Corresponding author.
Keywords: Anxiety, Dépression, dual pathology, ADHD

Conflict of interest:

No

Article ID: EPV0310

Confusion in children after moderate-severe and severe traumatic brain injury

Tyutyukina A. 1
Sidneva Y. 2 *
Bykova V. 3
1 Clinical and Research Institute of Emergency Pediatric Surgery and Trauma, Neurosurgery; Rehabilitation , Moscow , Russian Federation
2 Clinical and Research Institute of Emergency Pediatric Surgery and Trauma (CRIEPST), Department of Recovery Treatment And Rehabilitation, Moscow , Russian Federation
3 Clinical and Research Institute of Emergency Pediatric Surgery and Trauma (CRIEPST), Neurosurgery; Rehabilitation , Moscow , Russian Federation
* Corresponding author.
Keywords: Post-traumatic confusion, Children, traumatic brain injury

Conflict of interest:

No

Article ID: EPV0312

Start-up and first results of the dual pathology outpatient program in salamanca.

Vicente Hernandez B. 1 *
Gutierrez Álvarez P.R. 1
Martinez R. 1
Herranz Rodríguez S. 1
Recio Martín L. 1
García Sanchez R. 1
Roncero C. 2
Álvarez A. 1
1 University of Salamanca Health Care Complex , Psychiatry, Salamanca , Spain
2 University of Salamanca Health Care Complex ,, Psychiatry, Salamanca , Spain
* Corresponding author.
Keywords: dual pathology, dual disorders, outpatient program

Conflict of interest:

No

Article ID: EPV0315

Personality features, dissociation, self-stigma, hope, and the complex treatment of depressive disorder

Prasko J. 1 *
Ociskova M. 1
Slepecky M. 2
Kotianova A. 2
1 Faculty of Medicine and Dentistry, University Palacky Olomouc , Department of Psychiatry, Olomouc , Czech Republic
2 Faculty of Social Science and Health Care - Constantine the Philosopher University in Nitra- Slovak Republic ,, Department of Psychology Sciences, Nitra , Slovak Republic
* Corresponding author.
Keywords: dissociation, Depressive disorder, personality features, Psychotherapy

Conflict of interest:

No

Article ID: EPV0317

Psychiatric comorbidity and use of psychopharmacology treatments in adults with autism spectrum disorder (ASD).

Álvarez Pedrero A. *
Palacin A.
Parra I.
Muñoz R.
Membrives S.
Llorens M.
Santos L.
Monreal J.A.
Palao Vidal D.
Parc Taulí University Hospital. Autonomous University of Barcelona (UAB). I3PT, Mental Health, Sabadell , Spain
* Corresponding author.
Keywords: psychopharmacology, Autism, comorbidity, ASD

Conflict of interest:

No

Article ID: EPV0321

Fahr's syndrome revealed by a psychotic disorder: a case report

Ben Hamadi A. 1 *
Ben Ammar H. 1
Hamdoun J. 1
Khelifa E. 2
Aissa A. 3
Zouhaier E.H. 3
1 Razi hospital, Psychiatry F, Manouba , Tunisia
2 University of Tunis El Manar- Faculty of medecine of Tunis, Psychiatry - Razi Hospital, Tunis , Tunisia
3 Razi Hospital, F Department, Tunis , Tunisia
* Corresponding author.
Keywords: comorbidity, dementia, Fahr syndrome

Conflict of interest:

No

Article ID: EPV0322

Prevalence and characteristics of substance use disorders among moroccan patients with a first psychotic episode

Boualame A. 1 2 *
Elfahiri F.Z. 1
Adali I. 3 4
Manoudi F. 3 4
Asri F. 1 4
1 Mohammed VI University Hospital, Psychiatry, Marrakech , Morocco
2 Vichy Hospital, Psychiatry, Vichy , France
3 CHU Mohamed VI, Psychiatry, marrakesh , Morocco
4 Equipe de recherche pour la santé mentale, service de psychiatrie, Hôpital Ibn Nafis, CHU MARRAKECH, Psychiatry, Marrakech , Morocco
* Corresponding author.
Keywords: substance use disorder, cannabis, brief psychotic episode

Conflict of interest:

No

Article ID: EPV0323

Depression severity is associated with sarcopenia in non-elderly chinese inpatients with major depressive disorder

Fan X. 1 *
Yang J. 1
Yuan J. 1
Wei Y. 1
Jin X. 2
1 The Second Affiliated Hospital of Kunming Medical University , Psychiatry, Kunming , China
2 The Sixth Affiliated Hospital of Kunming Medical University (the People's Hospital of Yuxi), Geriatrics, Kunming , China
* Corresponding author.
Keywords: sarcopenia, severity, major depressive disorder

Conflict of interest:

No

Article ID: EPV0326

Comorbid alcohol dependence and generalized anxiety disorder

Grahovac Juretić T. 1 2 *
Rebić J. 1 2
Stevanović A. 1 2 3
Letica Crepulja M. 1 2
Došen A. 2
Rončević Gržeta I. 1 2
1 Clinical Hospital Rijeka, Psychiatric Clinic , Rijeka , Croatia
2 Faculty of Medicine University of Rijeka, Department of Psychiatry And Psychological Medicine, Rijeka , Croatia
3 Faculty of health studies University of Rijeka, Department of Basic Medical Sciences, Rijeka , Croatia
* Corresponding author.
Keywords: Alcohol dependence, Group treatment, social skills training, generalized anxiety disorder

Conflict of interest:

No

Article ID: EPV0327

Evaluation of disease control in greek patients with major depressive disorder with/without generalized anxiety disorder and comorbidities–pronoi study

Nimatoudis I. 1
Petrikis P. 2
Konstantinou A. 3
Bargiota S.I. 4
Ginis A. 5
Karagiovanaki S. 6 *
1 AHEPA University General Hospital of Thessaloniki , 3rd Department of Psychiatry, Thessaloniki , Greece
2 University Hospital of Ioannina , Department of Psychiatry, IOANNINA , Greece
3 Private Site, Psychiatry, Larissa , Greece
4 Private Site, Psychiatry, Thessaloniki , Greece
5 Elpen Pharmaceutical Co. Inc ., Medical , Pikermi , Greece
6 Elpen Pharmaceutical Co. Inc., Clinical Research , Pikermi , Greece
* Corresponding author.
Keywords: major depressive disorder, comorbidities, generalized anxiety disorder, disease control

Conflict of interest:

No

Article ID: EPV0328

Evaluation of quality of life in greek patients with major depressive disorder with/without generalized anxiety disorder and comorbidities-pronoi study

Gourzis P. 1
Konsta A. 2
Koutsodonti D. 3
Kampouri E. 4
Ginis A. 5
Karagiovanaki S. 6 *
1 School of Medicine, University of Patras, Greece , Psychiatry , Patras, Greece
2 Aristotle University of Thessaloniki, Medical School, Thessaloniki , Greece
3 Private Site, Psychiatry, Ilion , Greece
4 Private Site, Psychiatry, Perama , Greece
5 Elpen Pharmaceutical Co. Inc ., Medical , Pikermi , Greece
6 Elpen Pharmaceutical Co. Inc., Clinical Research , Pikermi , Greece
* Corresponding author.
Keywords: major depression disorder, comorbidities, anxiety disorder, quality of life

Conflict of interest:

No

Article ID: EPV0330

Depressive disorder in cardiological inpatients

Kornetov N. 1 *
Molodykh O. 2
Zvereva N. 3
Arzhanik A. 2
1 Siberian State Medical University , Department of Psychiatry, Drug Addiction, Psychotherapy, Tomsk , Russian Federation
2 Siberian States Medical Unifersity , 4th Year Student Of The Medical Faculty, Tomsk , Russian Federation
3 Federal state BUDGETARY INSTITUTION "SIBERIAN FEDERAL SCIENTIFIC and CLINICAL CENTER of the FEDERAL MEDICAL and BIOLOGICAL AGENCY" of Russia, Department of Psychiatry, Drug Addiction, Psychotherapy, Seversk , Russian Federation
* Corresponding author.
Keywords: Cardiologic inpathients, comorbidity, Educational Program, Depressive disorder

Conflict of interest:

No

Article ID: EPV0331

Patient with bipolar disorder and prolactinoma treated with aripiprazole: a case report

Kampouras I. 1
Louki F. 2 *
1 PSYCHIATRIC HOSPITAL OF ATTICA, Medical Health Centre Of Athens, ATHENS , Greece
2 PSYCHIATRIC HOSPITAL OF ATHENS, Mental Health Centre Agii Anargiri, ATHENS , Greece
* Corresponding author.
Keywords: bipolar disorder and prolactinoma and aripiprazole

Conflict of interest:

No

Article ID: EPV0334

Comorbidities in dementia: state of knowledge.

Pawłowski M. 1 *
Łuc M. 1
Lenart M. 1
Szcześniak D. 1
Seifert I. 2
Wiegelman H. 2
Wolf-Ostermann K. 2
Gerhardus A. 2
Rouwette E. 3
Vernooij-Dassen M. 4
Ikram M.A. 5
Melis R.J.F. 6
Welmer A.-K. 7
Brodaty H. 8
Thyrian J.R. 9
Davis D. 10
Chattat R. 11
Jeon Y.-H. 12
Perry M. 6
Samtani S. 8
Wang H.X. 13 14
Rymaszewska J. 1
1 Wroclaw Medical Univeristy , Department of Psychiatry, Wroclaw , Poland
2 University of Bremen , Institute of Public Health And Nursing Research, Bremen , Germany
3 Radboud University , Department of Business Administration, Nijmegen , Netherlands
4 Radboud University , Radboudumc Iq Healthcare, Nijmegen , Netherlands
5 Erasmus MC University Medical Center , Department of Epidemiology, Rotterdam , Netherlands
6 Radboud University Medical Center ,, Department of Geriatrics, Nijmegen , Netherlands
7 Karolinska Institutet , Department of Neurobiology, Care Sciences And Society, Solna , Sweden
8 University of New South Wales , Centre For Healthy Brain Ageing, Sydney , Australia
9 Deutsches Zentrum für Neurodegenerative Erkrankungen, Site Rostock/ Greifswald, Bonn , Germany
10 University College London , Mrc Unit For Lifelong Health And Ageing, London , United Kingdom
11 University of Bologna, Department of Psychology Psi, Bologna , Italy
12 The University of Sydney , Faculty of Nursing And Midwifery, Sydney , Australia
13 Stockholm University, Stress Research Institute , Stockholm , Sweden
14 Karolinska Institutet , Department of Neurobiology, Care Sciences And Society, Stockholm , Sweden
* Corresponding author.
Keywords: cognitive decline, dementia, comorbidity

Conflict of interest:

No

Article ID: EPV0336

Does anxiety moderate the efficacy of mirtazapine in patients with treatment resistant depression? A secondary analysis of the mir trial

Rifkin-Zybutz R. 1 *
Macneill S. 1
Dickens C. 2
Campbell J. 3
Chew-Graham C. 4
Peters T. 1
Wiles N. 1
Kessler D. 1
1 University of Bristol , Centre Of Academic Mental Health, Bristol , United Kingdom
2 University of Exeter , Mental Health Research Group, Exeter , United Kingdom
3 University of Exeter , Academic Primary Care, Exeter , United Kingdom
4 University Of Keele , Academic Primary Care, Keele , United Kingdom
* Corresponding author.
Keywords: Treatment resistance, Mirtazapine, Anxiety

Conflict of interest:

No

Article ID: EPV0337

The chemsex phenomenon and its associated risk of psychosis

Almeida C. *
Miranda J.
Barbosa M.
Santos Silva L.
Araújo R.
Silva M.
Centro Hospitalar de Leiria, Psychiatry And Mental Health, Leiria , Portugal
* Corresponding author.
Keywords: slamsex, chemsex, psychosis

Conflict of interest:

No

Article ID: EPV0340
Subjects: Abstract, Consultation Liaison Psychiatry and Psychosomatics

Psychiatric symptoms associated with septo-optic dysplasia.

Becerra Darriba H.
Osasunbidea - Servicio Navarro de Salud, Centro De Salud Mental De Tudela, Tudela , Spain
Keywords: Organic Mental Disorder, Septo-optic dysplasia, Morsier's syndrome

Conflict of interest:

No

Article ID: EPV0344

The role of psychosocial factors in development and urticaria treatment

Blazevic Zelic S. 1 *
Pavešić Radonja A. 2
Skarpa I. 1
1 Clinical hospital Rijeka, Psychiatry Department, Rijeka , Croatia
2 University Hospital Rijeka , Psychiatry, Rijeka , Croatia
* Corresponding author.
Keywords: Coping skills, satisfaction with life, Personality traits, Urticaria

Conflict of interest:

No

Article ID: EPV0345

Brief psychotic episode in a patient living with HIV and treated with dolutegravir. Much more common tan we thought? A case report and review.

Curto Ramos J. 1 *
Kollias G. 1
Tsoukalis G. 2
1 La Paz University Hospital , Psychiatry, Clinical Psychology And Mental Health, Madrid , Spain
2 HOSPITAL RODRIGUEZ LAFORA , Psychiatry, Clinical Psychology And Mental Health, MADRID, Spain
* Corresponding author.
Keywords: ADVERSE EFFECTS ARV, HIV

Conflict of interest:

No

Article ID: EPV0346

Eye movement desensitisation and reprocessing therapy for medically unexplained symptoms: a qualitative study of patients’ experiences

Hussain A. 1
Proudlock S. 2
Peris J. 2
Hopkins C. 3 *
1 Berkshire Healthcare NHS Foundation Trust, Community Psychological Medicine Service, Bracknell , United Kingdom
2 Berkshire Healthcare NHS Foundation Trust, Prospect Park Hospital, Reading , United Kingdom
3 Berkshire Healthcare NHS Foundation Trust, Psychological Medicine Service, Royal Berkshire Hospital , Reading , United Kingdom
* Corresponding author.
Keywords: Eye movement desensitisation & reprocessing therapy, Medically unexplained symptoms

Conflict of interest:

No

Article ID: EPV0347

Eye movement desensitisation and reprocessing therapy for the treatment of functional neurological disorder: a case series

Hussain A. 1
Peris J. 2
Proudlock S. 2
Hopkins C. 3 *
1 Berkshire Healthcare NHS Foundation Trust, Community Psychological Medicine Service, Reading , United Kingdom
2 Berkshire Healthcare NHS Foundation Trust, Prospect Park Hospital, Reading , United Kingdom
3 Berkshire Healthcare NHS Foundation Trust, Psychological Medicine Service, Royal Berkshire Hospital, Reading , United Kingdom
* Corresponding author.
Keywords: Eye movement desensitisation & reprocessing therapy, Medically unexplained symptoms, functional neurological disorder

Conflict of interest:

No

Article ID: EPV0350

How to manage the risk for seretonin syndrome (SS) in patients with current antidepressant treatment requiring linezolid for a new resistant nosocomial infection?

Kollias G. 1 *
Tsoukalis G. 2
Garcia Martínez D. 1
Kollia A. 3
Suárez Lorenzo A. 1
Curto Ramos J. 1
Cebolla S. 1
Palao Á. 1
Rodríguez-Vega B. 1
Bravo-Ortiz M.F. 1
1 La Paz University Hospital , Psychiatry, Clinical Psychology And Mental Health, Madrid , Spain
2 Hospital Dr. Rodríguez Lafora, Psychiatry, Clinical Psychology And Mental Health, MADRID, Spain
3 Evangelismos General Hospital , Psychiatry, Athens , Greece
* Corresponding author.
Keywords: seretonin syndrom, antidepressant, linezolid

Conflict of interest:

No

Article ID: EPV0351

Psychosomatic correlates and psycho-emotional disorders in patients with dermatologic disorders and chronic itch

Markova M. 1 *
Yaremkevych R. 1
Kozhyna H. 2
Yudin M. 3
Mukharovska I. 4
1 Kharkiv Medical Academy of Postgraduate Education, Sexology, Medical Psychology, Medical Abd Psychological Rehabilitation, Kharkiv , Ukraine
2 KHARKIV NATIONAL MEDICAL UNIVERSITY, Psychiatry, Narcology And Medical Psychology, Kharkiv , Ukraine
3 2Kharkiv National Medical University, Psychiatry, Narcology And Medical Psychology, Kharkiv , Ukraine
4 Bogomolets National Medical University, Medical Psychology, Psychosomatic Medicine and Psychotherapy, Kharkiv , Ukraine
* Corresponding author.
Keywords: chronic itch, psycho-emotional disorders

Conflict of interest:

No

Article ID: EPV0353

Psychosocial assessment in heart transplant. A literature review and a case report

López Álvarez I. 1
Palao Á. 2 *
Torrijos M. 3
Curto Ramos J. 4
Svintitckaia A. 1
1 HOSPITAL UNIVERSITARIO LA PAZ, Psychiatry, Clinical Psychology And Mental Health, Madrid , Spain
2 HOSPITAL UNIVERSITARIO LA PAZ, Psiquiatria , MADRID , Spain
3 Psychiatry and Mental Health Department La Paz Hospital., Psychiatry, MADRID , Spain
4 La Paz University Hospital, Psychiatry, Clinical Psychology And Mental Health, Madrid , Spain
* Corresponding author.
Keywords: psychosocial assessment, heart transplant

Conflict of interest:

No

Article ID: EPV0357

Delirium vs dementia: an approach since liaison psychiatry

Ruiz Martínez G.M. 1 *
Soldado Rodriguez L. 2
Solis M. 3
1 Neurotraumatological Hospital , Mental Health Hospitalization Unit, Jaen , Spain
2 Neurotraumatological Hospital, Mir , Mental Health Unit, Jaen , Spain
3 COMPLEJO HOSPITALARIO DE JAEN, Psiquiatria , JAEN , Spain
* Corresponding author.
Keywords: delirium, dementia, Liaison Psychiatry, differential diagnosis

Conflict of interest:

No

Article ID: EPV0358

Diagnostic and management challenges in an elderly male with neuropsychiatric manifestations of HIV and neurosyphilis

Selvaraj A. 1 *
Tan H.T. 2
1 Institute of Mental Health, Forensic Psychiatry , Singapore , Singapore
2 Institute of Mental Health, Developmental Psychiatry , Singapore , Singapore
* Corresponding author.
Keywords: HIV, consultation-liaison psychiatry, Neurosyphilis, Neuropsychiatric symptoms

Conflict of interest:

No

Article ID: EPV0362

When should an anti-NMDAR encephalitis be suspected in a pacient with symptoms of mood disorder

Tsoukalis G. 1 *
Kollias G. 2
Cebolla S. 2
Medina Lopez A. 2
Garcia Martínez D. 2
Curto Ramos J. 2
Cáceres Quintanilla E. 1
Rodríguez-Vega B. 3
Bravo-Ortiz M.F. 3
Palao Á. 4
López Álvarez I. 5
1 Hospital Dr. Rodríguez Lafora , Psychiatry, Clinical Psychology And Mental Health, MADRID, Spain
2 La Paz University Hospital, Psychiatry, Clinical Psychology And Mental Health, Madrid , Spain
3 La Paz University Hospital Research Health Institute (IdiPAZ), Psychiatry, Clinical Psychology And Mental Health, Madrid , Spain
4 HOSPITAL UNIVERSITARIO LA PAZ , Psiquiatria , MADRID , Spain
5 HOSPITAL UNIVERSITARIO LA PAZ, Psychiatry, Clinical Psychology And Mental Health, Madrid , Spain
* Corresponding author.
Keywords: mood disorder, ovarian teratoma, maniac episode, anti-NMDAR encephalitis

Conflict of interest:

No

Article ID: EPV0366

Intramuscular aripiprazole as a safe drug in QT index prolongation in patients with acute confusion syndrome. Case report

Del Sol Calderón P. *
Izquierdo De La Puente Á.
Garcia Moreno M.
HOSPITAL UNIVERSITARIO PUERTA DE HIERRO MAJADAHONDA, Psychiatry, MADRID , Spain
* Corresponding author.
Keywords: aripriprazole, QT index, adverse effect, delirium

Conflict of interest:

No

Article ID: EPV0373

Lithium toxicity following sleeve gastrectomy

Trenton A.
Anam A.
Aziz R.
Chaguturu V.
Coluccio M.
Kaufman K. *
Tobia A.
Rutgers Robert Wood Johnson Medical School, Psychiatry, New Brunswick , United States of America
* Corresponding author.
Keywords: bariatric surgery, lithium toxicity

Conflict of interest:

No

Article ID: EPV0374

Psychotic relapse after hepatitis C treatment with sofosbuvir and velpatasvir: a case report and literature review

Llach C. 1 *
Ilzarbe L. 1
Arbelo N. 1
Aranda N. 2
Barrio P. 3
Marfà M. 4
Guzman P. 3
1 Institute of Neuroscience/Hospital Clinic de Barcelona, Psychiatry And Psychology, Barcelona , Spain
2 Hospital Regional de Chepo, Psiquiatría, Chepo , Panama
3 Addictive Behaviors Unit/Hospital Clinic de Barcelona, Psychiatry And Psychology, Barcelona , Spain
4 Hospital Clinic de Barcelona, Clinical Pharmacology, Barcelona , Spain
* Corresponding author.
Keywords: schizophrenia, hepatitis C, Direct Antiviral Agent, sofosbuvir/velpatasvir

Conflict of interest:

No

Article ID: EPV0376

Prevalence and associated factors of depressive disorder in lung cancer patients received chemotherapy at the chemotherapy unit: a cross-sectional study

Maneeton B. 1 *
Maneeton N. 1
Kitiruangsaeng U. 1
Eurviriyanukul K. 1
Reungyos J. 1
Srisurapanont M. 1
Chewaskulyong B. 2
Woottiluk P. 3
Wanaratwichit W. 4
Varnado P. 1
Traisathit P. 5
Homnan N. 5
Bunyatisai W. 5
Kawilapat S. 1
1 Chiang Mai University , Department of Psychiatry, Faculty of Medicine, Chiang Mai, Thailand
2 Chiang Mai University , Department of Medicine, Faculty of Medicine, Chiang Mai, Thailand
3 Chiang Mai University , Department of Psychiatric Nursing, Faculty of Nursing, Chiang Mai, Thailand
4 Chiang Mai University , Chemotherapy Unit, Maharaj Nakorn Chiang Mai Hospital, Faculty of Medicine, Chiang Mai, Thailand
5 Chiang Mai University , Department of Statistics, Faculty of Science, Chiang Mai, Thailand
* Corresponding author.
Keywords: Depressive disorder, lung cancer, prevalence

Article ID: EPV0377

Coping with breast cancer - the experience of a specialized liaison psychiatric consultation

Mota D. 1 *
Oliveira L. 2
Ribeiro L. 2
1 Centro Hospitalar Vila Nova de Gaia/ Espinho, Psychiatry And Mental Health, Vila Nova de Gaia, Portugal
2 Centro Hospitalar Vila Nova de Gaia/Espinho, Serviço De Psiquiatria, Vila Nova de Gaia, Portugal
* Corresponding author.
Keywords: consultation liaison psychiatry, breast cancer, Coping, psychooncology

Conflict of interest:

No

Article ID: EPV0379

Neurotoxicity and depression symptomatology changes in patients with chronic hepatitis C after viral cure with direct-acting antivirals

Navines R. 1 *
Cañizares S. 1
Bartres C. 2
Nácar L. 1
Lens S. 2
Rodriguez-Tajes S. 2
Pariente J.C. 3
Muñoz-Moreno E. 3
Cavero M. 1
Bargalló N. 3
Forns X. 2
Mariño Z. 2
Capuron L. 4
Martin-Santos R. 1
1 Hospital Clinic, UB, IDIBAPS, CIBERSAM, Psychiatry And Psychology, Barcelona , Spain
2 Hospital Clinic, UB, IDIBAPS, CIBERehd , Liver Unit, Barcelona , Spain
3 Hospital Clinic, IDIBAPS, Neuroradiología, Servicio Radiología. Magnetic Resonance Image Core Facility., Barcelona , Spain
4 University of Bordeaux , Inra, Nutrition And Integrative Neurobiology Laboratory (nutrineuro), Bordeaux , France
* Corresponding author.
Keywords: neurotoxiciy, Dépression, direct-acting antivirals, chronic hepatitis c

Article ID: EPV0381

Gender differences in adherence to a psychoeducational group in a cardiac rehabilitation patients sample

Martin-Muñoz V.
Aguilar Sanchez L.
Muela De Blas P.
De Alarcón Gómez R.
Del Campo Bujedo F.
Velasco Cañedo M.J.
Oreja Sánchez C.
Roncero Alonso C. *
Hospital Clínico Universitario de Salamanca , Servicio De Psiquiatría, Salamanca , Spain
* Corresponding author.
Keywords: CARDIAC, rehabilitation, Intervention, PSYCHOLOGICAL

Conflict of interest:

No

Article ID: EPV0382

Diagnosis differences between departments which request psychiatry liaison service consultation.

Trabsa A. 1 *
Casanovas F. 2
Arroyo À. 2
Pérez Oms A. 3
Salgado P. 2
Oller Canet S. 3
1 Autonomous University of Barcelona , Department of Psychiatry And Legal Medicine, Barcelona , Spain
2 Parc de Salut Mar, Institut De Neuropsiquiatria I Adiccions, Barcelona , Spain
3 Institut de Neuropsiquiatria i Addicions (INAD)- Parc Salut Mar. Barcelona , Spain, Psychiatry, Barcelona , Spain
* Corresponding author.
Keywords: LiaisonPsychiatry, psychosomatics, Consultation, generalhospital

Conflict of interest:

No

Article ID: EPV0383

Interruption of psychopharmacologic treatment in general hospital comparing surgery and internal medicine hospitalized patients.

Trabsa A. 1 *
Casanovas F. 2
Arroyo À. 2
Pérez Oms A. 3
Salgado P. 2
Oller Canet S. 3
1 Autonomous University of Barcelona , Department of Psychiatry And Legal Medicine, Barcelona , Spain
2 Parc de Salut Mar, Institut De Neuropsiquiatria I Adiccions, Barcelona , Spain
3 Institut de Neuropsiquiatria i Addicions (INAD)- Parc Salut Mar. Barcelona, Spain, Psychiatry , Barcelona , Spain
* Corresponding author.
Keywords: treatment, Antidepressants, psychosomatics, LiaisonPsychiatry

Conflict of interest:

No

Article ID: EPV0384

Pernicious anemia revealed by a young onset dementia

Boualame A. 1 2 *
Elfahiri F.Z. 2
Adali I. 2 3
Manoudi F. 2
Asri F. 2 3
1 Vichy Hospital , Psychiatry, Vichy , France
2 Mohammed VI University Hospital , Psychiatry, Marrakech , Morocco
3 Equipe de recherche pour la santé mentale, service de psychiatrie, Hôpital Ibn Nafis, CHU MARRAKECH, Psychiatry, Marrakech , Morocco
* Corresponding author.
Keywords: Vitamin B12, Young-onset dementia, pernicious anemia

Conflict of interest:

No

Article ID: EPV0385

Psychiatric manifestations revealing limbic encephalitis: case report

Gharmoul M. 1
Bouzaabia Z. 2 *
Amamou B. 3
Ben Haouala A. 3
Ben Mohamed B. 3
Mhalla A. 3
Gaha L. 4
1 fattouma bourguiba hospital, Monastir, Psychiatry Department, monastir , Tunisia
2 Farhat Hached Hospital, Sousse, Tunisia, Psychiatry Department, SOUSSE , Tunisia
3 Fattouma Bourguiba Hospital, Psychiatric Department, Monastir , Tunisia
4 University Hospital , Psychiatry, Monastir , Tunisia
* Corresponding author.
Keywords: Psychiatric; behavioral, disorders, limbic encephalitis, diagnosis

Conflict of interest:

No

Article ID: EPV0386

Somatic complications in hospitalized patients with psychiatric comorbidity in a general hospital; a descriptive study.

Casanovas F. 1 *
Trabsa A. 2
Dinamarca Cáceres F.N. 1
Arroyo À. 1
Pérez Oms A. 1
Salgado P. 1
Oller Canet S. 1
1 Parc de Salut Mar, Institut De Neuropsiquiatria I Adiccions, Barcelona , Spain
2 Autonomous University of Barcelona , Department of Psychiatry And Legal Medicine, Barcelona , Spain
* Corresponding author.
Keywords: comorbidity, somatic complications, length of stay

Conflict of interest:

No

Article ID: EPV0387

Motivation and perfectionism in middle-age patients with essential hypertension

Pervichko E.
Podstreshnaya A.
Koniukhovskaia J. *
Lomonosov Moscow State University , Faculty of Psychology, Moscow , Russian Federation
* Corresponding author.
Keywords: Power motive, Affiliation motive, Achievement motive, Essential hypertension

Article ID: EPV0388

Are there any differences in emotional control in cardiovascular patients?

Nikolaeva O. 1
Nikolaev E. 2 *
Bogdanov A. 2
Lazareva E. 3
Dulina G. 3
1 Chuvash Republic Cardiology Clinic, Cardiosurgery Unit, Cheboksary , Russian Federation
2 Ulianov Chuvash State University , Faculty of Medicine, Cheboksary , Russian Federation
3 Ulianov Chuvash State University , Social And Clinical Psychology Department, Cheboksary , Russian Federation
* Corresponding author.
Keywords: emotional expression, emotional control, cardiovascular patients, Prevention

Conflict of interest:

No

Article ID: EPV0390
Subjects: Abstract, Cultural Psychiatry

Pharmacological treatment in mental health resources. Are there differences between the united states and spain?

Batalla Monedero M. 1 *
Rodríguez Soria E. 2
Carracedo Sanchidrián D. 3
1 Hospital La Fe, Psychiatry, Valencia , Spain
2 Hospital Universitario de Fuenlabrada , Psychiatry, Fuenlabrada , Spain
3 Hospital Universitario la Paz, Mental Health , Madrid , Spain
* Corresponding author.
Keywords: polipharmacy, psychopharmacology, Cultural Psychatry, Sociodemographic factors

Conflict of interest:

No

Article ID: EPV0391

The me, me, me generation is now the new normal

Bellotti J. 1
Granatto S. 2
Toniolo G. 2
Corti G. 3 *
1 Universidad del Museo Social Argentino , Posgrado: Psquiatría Perinatal, Ciudad Autónoma de Buenos Aires, Argentina
2 Universidad del Museo Social Argentino , Formación Continua, Ciudad Autónoma de Buenos Aires, Argentina
3 Universidad del Museo Social Argentino , Posgrado: Psiquiatría Perinatal, Ciudad Autónoma de Buenos Aires, Argentina
* Corresponding author.
Keywords: Socio-Cultural Paradigm Shift, Novel mental health disorders, Me, Me, Me Generation, Millennial

Conflict of interest:

No

Article ID: EPV0393

Do you speak my language? Exploring the differences between two psychiatrists' intracultural and intercultural countertransference while treating the same patient, a case study

Gjonbalaj I. *
Labib S.
Montefiore Department of Psychiatry and Behavioral Sciences, Psychiatry, Bronx , United States of America
* Corresponding author.
Keywords: cultural psychiatry, countertransference, case study

Conflict of interest:

No

Article ID: EPV0394

Examining the factor structure of the russian translation of the general health questionnaire-12 (GHQ-12) among a sample of russian university students

Chuykova T. 1
Malik J.A. 2
Lewis C.A. 3 *
1 Bashkir State Pedagogical University , Psychology, Ufa , Russian Federation
2 Quaid-i-Azam University , National Institute of Psychology, Islamabad , Pakistan
3 Leeds Trinity University , Social And Behavioural Sciences, Leeds , United Kingdom
* Corresponding author.
Keywords: General Health Questionnaire, GHQ-12, Students, Russian

Conflict of interest:

No

Article ID: EPV0395

Mental health literacy among an online convenience sample of russian adults: examining lay people’s implicit theories of the causes of alcoholism

Lewis C.A. 1 *
Davis S. 2
Khukhrin M. 3
Galyautdinova S. 3
1 Leeds Trinity University , Social And Behavioural Sciences, Leeds , United Kingdom
2 Glyndwr University , Psychology, Wrexham , United Kingdom
3 Bashkir State University , Psychology, Ufa , Russian Federation
* Corresponding author.
Keywords: lay, Russian, alcoholism, implicit

Conflict of interest:

No

Article ID: EPV0396

Stress, resilience, and quality of life among parents of children with physical or intellectual disabilities in pakistan

Akhtar N. 1
Iqbal Malik N. 2
Ahmad Rana S. 3
Lewis C.A. 4 *
1 Islamia University Bahawalpur , Bahawalnagar Campus, Psychology, Bahawalpur , Pakistan
2 University of Sargodha , Psychology, Sargodha , Pakistan
3 MAO College Lahore , Psychology, Lahore , Pakistan
4 Leeds trinity University , Social And Behavioural Sciences, Leeds , United Kingdom
* Corresponding author.
Keywords: stress, Resilience, quality of life, disabled

Conflict of interest:

No

Article ID: EPV0397

‘Kidulthood’ myth: is russian ‘Y’ generation infantile? A self-concept and stereotype research’

Lianguzova V. *
Rikel A.
Lomonosov Moscow State University , Psychology, Moscow , Russian Federation
* Corresponding author.
Keywords: infantility, stereotypes, self-concept, generations

Conflict of interest:

No

Article ID: EPV0398

Koro syndrome: a case report in spain.

Renau Mínguez B. 1 *
Juanes A. 2
Cruz Fourcade J.F. 3
Alvarez-Sesmero S. 3
Losada C. 3
Inca Torres K. 4
1 Complejo Asistencial de Zamora , Psychiatry, Zamora , Spain
2 Hospital Universitario 12 de Octubre , Psychiatry, Madrid , Spain
3 Hospital 12 de Octubre, Liaison Psychiatry, Madrid , Spain
4 Hospital 12 de Octubre, Psychiatry, Madrid , Spain
* Corresponding author.
Keywords: Koro syndrome, culture-bound syndrome, AIDS

Conflict of interest:

No

Article ID: EPV0399

Impact of migratory processes and cultural change on mental health in the USA.

Batalla Monedero M. 1
Rodríguez Soria E. 2 *
Carracedo Sanchidrián D. 3
1 Hospital La Fe , Psychiatry, Valencia , Spain
2 Hospital Universitario de Fuenlabrada , Psychiatry, Fuenlabrada , Spain
3 Hospital Universitario la Paz, Mental Health , Madrid , Spain
* Corresponding author.
Keywords: cultural psychiatry, migration, grieving process, traumatic life events

Conflict of interest:

No

Article ID: EPV0403

Factors of machiavellianism spreading in general population

Sokolova E. 1
Andreyuk K. 1
Ryzhov A. 1 *
Zhuykova E. 2
1 Lomonosov MSU , Faculty of Psychology, Moscow , Russian Federation
2 Psychological Institute of the Russian Academy of Education , Adolescent Psychology Laboratory, Moscow , Russian Federation
* Corresponding author.
Keywords: tolerance to ambiguity, Empathy, Cultural syndromes, Machiavellianism

Conflict of interest:

No

Article ID: EPV0404

Approbation of opinions about mental illness (OMI) questionnaire on Russian speaking sample in azerbaijan

Ryzhov A. 1 *
Mitina O. 1
Tkhostov A. 1
Sokolova E. 1
Gummatova F. 2
1 Lomonosov MSU , Faculty of Psychology, Moscow , Russian Federation
2 Lomonosov MSU , Baku Branch, Baku , Azerbaijan
* Corresponding author.
Keywords: OMI, family burden, Azerbaijan, Stigma

Conflict of interest:

No

Article ID: EPV0405

Cultural psychiatry: psychiatric emergencies in uganda

Bullón-Sáez A.
Complejo Asistencial Universitario de Salamanca , Psychiatry, SALAMANCA , Spain
Keywords: uganda, emergency psychiatry, psychiatric disorders, Cultural Psychiatry

Conflict of interest:

No

Article ID: EPV0406

Decentering ability of mental health professionals working in perinatal mental health in cross-cultural situations.

D'Alessandro A. 1 *
Mestre C. 2
1 University of Poitiers , Psychiatry Department, Poitiers , France
2 Bordeaux University Hospital , Psychiatry Department, BORDEAUX , France
* Corresponding author.
Keywords: Decentering, Mental Health Professionals, Perinatal Period, Migrants

Conflict of interest:

No

Article ID: EPV0407

Plasticity and stability of delusional content in schizophrenia. Religious topics in the 80 years period

Krysta K. *
Dudek A.
Górna A.
Krzystanek M.
Medical University of Silesia , Department of Rehabilitation Psychiatry, Katowice , Poland
* Corresponding author.
Keywords: schizophrenia, delusions, psychopathology

Conflict of interest:

No

Article ID: EPV0408

Acute stress disorder due to work-related stress among oil platform employees in azerbaijan

Mammadzada G. 1 *
Manucheri-Lalen A. 1
Mammadova F. 2
1 Azerbaijan Medical University , Psychiatry, AZ , Azerbaijan
2 Republican Treatment and Diagnostic Hospital, Psychiatry, Baku , Azerbaijan
* Corresponding author.
Keywords: oilworkers, stress, stressdisorder

Article ID: EPV0411

Two-eyed seeing as a philosophy to facilitate communication among indigenous knowledge keepers and mental health professions about indigenous suicide

Mehl-Madrona L. 1
Mainguy B. 1 *
Mcfarlane P. 2
1 University of Maine, Graduate School , Orono , United States of America
2 University of New England/Eastern Maine Medical Center , Family Medicine Residency, Bangor , United States of America
* Corresponding author.
Keywords: indigenous North Americans, two-eyed seeing, narrative understanding, Suicide

Conflict of interest:

No

Article ID: EPV0415
Subjects: Abstract, Depressive Disorders

The theory of mind and alexithymia in patients with recurrent depressive disorder

Culici A. 1 *
Negru I. 2
Negrila V.I. 3
Popovici Z. 4
Bredicean C. 5
1 Timișoara County Emergency Clinical Hospital, Clinic Of Psychiatry ,,eduard Pamfil", Timișoara , Romania
2 ”Victor Babeș” University of Medicine and Pharmacy , Faculty of Medicine, Timișoara , Romania
3 Timișoara County Emergency Hospital, Clinic Of Psychiatry ,,eduard Pamfil”, Timisoara , Romania
4 Arad County Emergency Clinical Hospital, Centre For Mental Health, Arad , Romania
5 "Victor Babeș” University of Medicine and Pharmacy , Neuroscience, Timișoara , Romania
* Corresponding author.
Keywords: Alexithymia, Depressive disorder, theory of mind

Conflict of interest:

No

Article ID: EPV0419

Finasteride and depressive symptoms, case report

Garcia Moreno M. 1 *
De Cós Milas A. 2
Izquierdo De La Puente Á. 1
Del Sol Calderón P. 1
Beatobe Carreño L. 2
1 HOSPITAL UNIVERSITARIO PUERTA DE HIERRO MAJADAHONDA , Psychiatry, MADRID , Spain
2 HOSPITAL UNIVERSITARIO DE MÓSTOLES , Psychiatry, MADRID , Spain
* Corresponding author.
Keywords: depression finasteride

Conflict of interest:

No

Article ID: EPV0420

Dermatomyositis with major depressive disorder and suicidal ideation comorbidity: a case report.

Ghrissi F. *
Fekih-Romdhane F.
Razi Hospital, Psychiatry University Tunis El Manar , Mannouba , Tunisia
* Corresponding author.
Keywords: major depressive disorder, Suicidal ideation, dermatomyositis

Conflict of interest:

No

Article ID: EPV0424

Screening for depressive symptoms in outpatient primary health care

Kola K. *
Agaj H.
Elezi F.
Sotiri E.
UHC Mother Theresa Tirane, Neuroscience, Tirane , Albania
* Corresponding author.
Keywords: primary health care, Screening, Dépression, outpatient

Conflict of interest:

No

Article ID: EPV0426

Additive role of loneliness in depression-induced multimorbidity development in older people

De La Torre-Luque A. 1
Lara E. 2
Rico-Uribe L. 1
De La Fuente J. 3
Caballero F. 4
Sanchez-Niubo A. 5
Haro J.M. 6
Ayuso-Mateos J.L. 7
Lopez-Garcia P. 8 *
1 Center of Biomedical Research in Mental Health (CIBERSAM), Department of Psychiatry. Universidad Autonoma De Madrid , Madrid , Spain
2 Hospital Universitario de La Princesa , Psychiatry, Madrid , Spain
3 Universidad Autonoma de Madrid , Department of Psychiatry, Madrid , Spain
4 Universidad Autonoma de Madrid , Department of Preventive Medicine, Public Health, And Microbiology, Madrid , Spain
5 Parc Sanitari Sant Joan de Déu, Fundació Sant Joan De Déu, Barcelona , Spain
6 Parc Sanitari Sant Joan de Déu, Research, Innovation And Teaching Unit, Barcelona, Spain
7 Universidad Autonoma de Madrid , Psychiatry, Madrid , Spain
8 Faculty of Medicine. Universidad Autonoma of Madrid , Psychiatry, Madrid , Spain
* Corresponding author.
Keywords: Late-life depression, MULTIMORBIDITY, ATHLOS project, Loneliness

Conflict of interest:

No

Article ID: EPV0428

The structure of psychosocial maladaptation and anxiety-depressive symptomatics in women with different genesis of depression

Markov A. 1 *
Markova M. 2
Isakov R. 3
Herasimenko L. 3
1 Kharkiv Medical Academy of Postgraduate Education, Sexology, Medical Psychology Medical And Psychological Rehabilitation, Kharkiv , Ukraine
2 Kharkiv Medical Academy of Postgraduate Education, Sexology, Medical Psychology, Medical Abd Psychological Rehabilitation, Kharkiv , Ukraine
3 Ukrainian Medical Stomatological Academy, Department of Psychiatry, Narcology And Medical Psychology, Poltava , Ukraine
* Corresponding author.
Keywords: psychosocial maladaptation, Dépression

Conflict of interest:

No

Article ID: EPV0430

Maois are no treatment of „last resort“: a review of response after failed tranylcypromine in depression

Ulrich S. 1
Messer T. 2 *
1 Aristo Pharma GmbH, Medical-scientific Department, Berlin , Germany
2 Danuvius Clinics Pfaffenhofen, Clinics Of Psychiatry, Psychotherapy And Psychosomatics, Pfaffenhofen an der Ilm, Germany
* Corresponding author.
Keywords: tranylcypromine, monoamine oxidase inhibitor, Dépression, treatment resistant depression

Article ID: EPV0431

The emotional response to different tastes of food - as possible marker in recognition of depressive symptoms and suicidal ideations

Milasauskiene E. 1 *
Adomaitienė V. 1
Steibliene V. 1
Jarutiene L. 1
Bartkiene E. 2
Lele V. 2
Cernauskas D. 3
Klupsaite D. 3
Zadeike D. 3
Juodeikiene G. 3
1 Lithuanian University of Health Sciences , Psychiatry Department, Kaunas , Lithuania
2 Lithuanian University of Health Sciences , Food Safety And Quality, Kaunas , Lithuania
3 Kaunas University of Technology , Food Science And Technology, Kaunas , Lithuania
* Corresponding author.
Keywords: Suicidality, facereader, Dépression, food tastes

Conflict of interest:

No

Article ID: EPV0432

A prospective study of the association between depression and initiation of insulin therapy in people with type 2 diabetes.

Mohamedali Z. 1 *
Upsher R. 2
Ismail K. 2
Moulton C. 2
Lecher-Lombardi J. 2
1 Kings College London, School of Medicine, LONDON , United Kingdom
2 Institute of Psychiatry and Neuroscience, Department of Psychological Medicine, LONDON , United Kingdom
* Corresponding author.
Keywords: Insulin, Dépression, Diabetes

Conflict of interest:

No

Article ID: EPV0433

Depressive disorders in subjects with metabolic syndrome and cognitive vascular disorders

Piotrovskaya V. 1
Neznanov N. 2 *
1 First Pavlov Medical University , Psychiatry And Narcology, Saint Petersburg, Russian Federation
2 Federal State Budgetary Institution "National Medical Research Centre of psychiatry and neurology named after V.M. Bekhterev" , Psychiatry, Saint Petersburg, Russian Federation
* Corresponding author.
Keywords: metabolic syndrome, cognitive impierment, dementia, Dépression

Conflict of interest:

No

Article ID: EPV0435

Treatment-resistant depression? The importance of the differential diagnosis

Ramos García E. *
Martínez Fernández Á.
Molina Cambra R.
Muñoz Domenjó A.
García-Poggio Fernández-Renau M.
Sagarra Arruego R.
Bianchi Ramos F.L.
Ortega Moreno M.
Hernández Barrera M.
Hospital Universitario de Móstoles , Psiquiatría, Móstoles, Madrid , Spain
* Corresponding author.
Keywords: treatment, resistant, Dépression, diagnosis

Conflict of interest:

No

Article ID: EPV0436

Relationship betwenn childhood trauma and the recurrence of depression

Rebai A. 1 *
Maamri A. 1
Ghazouani N. 2
Maamouri R. 2
Zalila H. 1
1 Razi University Hospital , Adult Outpatient Psychiatry Department, Mannouba , Tunisia
2 Razi University Hospital , Psychiatry, Mannouba , Tunisia
* Corresponding author.
Keywords: Dépression, childhood, trauma, recurrence

Conflict of interest:

No

Article ID: EPV0437

Efficacy of docosahexaenoic acid and eicosapentaenoic acid in major depressive disorder. Report of 200 cases

Romero Guillena S.L.
U.G.C. Salud Mental Virgen Macarena, Psychiatry, Seville , Spain
Keywords: docosahexaenoic acid, Eicosapentaenoic acid, treatment, major depressive disorder

Conflict of interest:

No

Article ID: EPV0439

Clinical severity and physical activity predict cognitive impairment in major depressive disorder

Sánchez Carro Y. 1 *
Portella M. 2
Leal Leturia I. 3
Salvat N. 4
Soria V. 4
Álvarez P. 5
Lopez-Garcia P. 1
1 Faculty of Medicine. Universidad Autonoma of Madrid , Psychiatry, Madrid , Spain
2 Hospital de la Santa Creu I Sant Pau. Biomedical Research Institude Sant Pau , Psychiatry, Barcelona , Spain
3 La Princesa University Hospital , Psychiatry, Madrid , Spain
4 Bellvitge University Hospital -IDIBELL , Psychiatry, Barcelona , Spain
5 Institute of Neuropsychiatry and Addictions, Hospital del Mar, IMIM , Psychiatry, Barcelona , Spain
* Corresponding author.
Keywords: Major Depresive Disorder, Clinical Severity, Physical Activity and Cognition.

Conflict of interest:

No

Article ID: EPV0440

In-patient physical exercise program as an adjuvant therapy for depression

Skuhareuskaya M. 1 *
Yahlouskaya A. 2
Skugarevsky O. 2
Bedulin V. 3
1 The Republican Research and Practice Mental Health Center, Psychiatry, Minsk , Belarus
2 Belarusian State Medical University , Psychiatry, minsk , Belarus
3 Regional Hospital Randers, Psychiatry, Randers , Denmark
* Corresponding author.
Keywords: physical exercise, Dépression, treatment

Conflict of interest:

No

Article ID: EPV0441

A case of sarcoidosis with refractary major depression and fibromyalgia treated with electroconvulsive therapy (ECT)

Tenorio Villegas R. 1 *
De La Mata Hidalgo M. 2
Moleón Ruiz Á. 2
Suárez M. 2
Lineros R. 2
Mateos E. 2
1 Hospital Juan Ramon Jimenez, Psiquiatria, Huelva , Spain
2 Hospital Juan Ramón Jiménez, Psiquiatría, Huelva , Spain
* Corresponding author.
Keywords: depressive disorder, ECT, sarcoidosis

Conflict of interest:

No

Article ID: EPV0444

Immune-modulation therapy for depressive disorders

Vasile D. *
Vasiliu O.
Fainarea A.
Patrascu M.
Morariu E.
Stanescu R.
Manolache R.
Alexandru I.
Ghenoiu I.
Lecu R.
Gionea M.
Vlaicu R.
Amanolesei I.
Gainaru F.
University Emergency Central Military Hospital Dr. Carol Davila , Psychiatry, Bucharest , Romania
* Corresponding author.
Keywords: immune-modulation therapy, anti-cytokine therapy, pro-inflammatory markers, Depressive disorders

Article ID: EPV0445

Evidence-based psychotherapeutic interventions for young people with mood disorders: a systematic review.

Vella Fondacaro D. 1 *
Vousoura E. 2 3
Hochgerner 3
Poulsen S. 4
Torres S. 5
Cosmoiu A. 6
Camilleri N. 1
Saliba A. 1
Sacco R. 1
Saliba E. 1
Ulberg R. 7
Gergov V. 8
Tudor Tulbure B. 9
Löffler-Stastka H. 3
Prevendar T. 10
Markovska-Simoska S. 11
Chiarenza G. 12
Garcia-Lopez L.-J. 13
1 Mental Health Malta, Psychiatry, Attard , Malta
2 University of Athens , Psychology, Athens , Greece
3 Medical University , Psychiatry, Vienna , Austria
4 Department of Psychology, University of Copenhagen , Denmark, Psychology, Copenhagen , Denmark
5 Centro Hospitalar Barreiro Montijo, Psychiatry And Mental Health, Barreiro , Portugal
6 University of Bucharest , Psychology, Bucharest , Romania
7 Institute of Clinical Medicine, University of Oslo , Norway, Psychiatry, Oslo , Norway
8 University of Helsinki , Finland, Psychology, Helsinki , Finland
9 western university of Timsauri , Psychology, Timisauri s, Romania
10 Sigmund Freud University Vienna , Psychology, Vienna , Austria
11 Academy of Sciences and Arts of North Macedonia, Psychology, Skopje , Serbia and Montenegro
12 Centro Internazionale Disturbi di Apprendimento Attenzione Iperattività (CIDAAI), Psychiatry, Milan , Italy
13 UNIVERSITY OF JAEN, DEPARTAMENT OF PSYCHOLOGY, Psychology, Madrid , Spain
* Corresponding author.
Keywords: young people, Systematic Review, psychotherapy, Mood disorders

Conflict of interest:

No

Article ID: EPV0450

Biological and psychosocial predictors of treatment resistant depressive disorders

Markova M. 1 *
Rahman L. 2
Kozhyna H. 3
Zelenska K. 3
Leshchyna I. 4
Koshchii V. 5
Kosenko K. 6
Gaponov K. 7
1 Kharkiv Medical Academy of Postgraduate Education, Sexology, Medical Psychology, Medical Abd Psychological Rehabilitation, Kharkiv , Ukraine
2 Danylo Halytsky Lviv National Medical University , Psychiatry, Psychology And Sexology, Kharkiv , Ukraine
3 KHARKIV NATIONAL MEDICAL UNIVERSITY , Psychiatry, Narcology And Medical Psychology, Kharkiv , Ukraine
4 Kharkiv National Medical University , Psychiatry, Narcology And Medical Psychology, Kharkiv , Ukraine
5 Kharkiv National Medical University , Psychiatry,narcology And Medical Psychology, Kharkiv , Ukraine
6 Odessa National Medical University , Psychiatry, Odessa , Ukraine
7 Kharkiv Medical Academy of Postgraduate Education, Narcology, Kharkiv , Ukraine
* Corresponding author.
Keywords: treatment resistant depressive disorders, psychosocial predictors, biological predictors

Conflict of interest:

No

Article ID: EPV0453

The use of vasopressin type 1b receptor antagonists as psychotropic agents- a literature review

Vasiliu O.
University Emergency Central Military Hospital Dr. Carol Davila , Psychiatry, Bucharest , Romania
Keywords: vasopressin receptors, major depressive disorder, HPA axis, anxiety disorders

Article ID: EPV0461

The role of acupuncture in the treatment of depression in china

He X.
Zhengzhou University , Hospitality Management, Zhengzhou , China
Keywords: acupuncture depression antidepressant China

Conflict of interest:

No

Article ID: EPV0462

1. Paternal postnatal depression: is it an unrecognized disorder by DSM-5 and CIE-11?

Hernandez-Santillan G. 1 *
Bravo-Ortiz M.F. 2
Alcami Pertejo M. 2
Fernandez-Sanchez A. 2
Lahera G. 3
1 Universidad Autónoma de Madrid , Medicine, Madrid , Spain
2 La Paz University Hospital Research Health Institute (IdiPAZ) , Psychiatry, Clinical Psychology And Mental Health, Madrid , Spain
3 University of Alcala , Faculty of Medicine, Madrid , Spain
* Corresponding author.
Keywords: postnatal depression, paternal, father*, postpartum depression

Conflict of interest:

No

Article ID: EPV0463

Impact of comorbid alcohol use disorder on health-related quality of life among clinically depressed patients

Luoto K. 1 2 3 *
Koivukangas A. 2
Lassila A. 2
Sintonen H. 4
Leinonen E. 1 3
Kampman O. 1 3
1 Tampere University , Faculty of Medicine and Health Technology, Tampere , Finland
2 Seinäjoki Central Hospital, Hospital District of South Ostrobothnia, Department of Psychiatry, Seinäjoki , Finland
3 Tampere University Hospital , Pirkanmaa Hospital District, Department of Psychiatry, Tampere , Finland
4 University of Helsinki , Department of Public Health, Helsinki , Finland
* Corresponding author.
Keywords: Depressive disorder, alcohol use disorder, health-related quality of life

Conflict of interest:

No

Article ID: EPV0464

The prevalence and associated factors of depressive disorders in preclinical medical students

Kittipongpaisal G. 1
Cheeptham N. 1
Limsakul N. 1
Phornphiphatphong P. 1
Maneeton B. 2
Kawilapat S. 2
Maneeton N. 2 *
1 Chiang Mai University , Faculty of Medicine, Chiang Mai, Thailand
2 Chiang Mai University , Department of Psychiatry, Faculty of Medicine, Chiang Mai, Thailand
* Corresponding author.
Keywords: Depressive disorder, medical student, Traumatic event

Article ID: EPV0467

Polygenic liability and relative and absolute risk of recurrence in hospital-treated depression patients in denmark

Musliner K. 1 *
Vilhjálmsson B. 1
Albiñana Climente C. 1
Agerbo E. 1
Breen G. 2
Coleman J. 2
Østergaard S. 3
Mortensen P. 1
1 Aarhus University , National Center for Register-based Research, Aarhus , Denmark
2 Kings College London, Institute of Psychiatry , Psychology And Neuroscience, London , United Kingdom
3 Aarhus University Hospital , Psychiatry, Department of Affective Disorders, Aarhus N, Denmark
* Corresponding author.
Keywords: genetics, Dépression, absolute risk, recurrence

Conflict of interest:

No

Article ID: EPV0470

Effects of a single ketamine infusion on working memory-related brain activity in severely depressed patients: a functional magnetic imaging study

Stippl A. 1 *
Gärtner M. 1 2
Herrera A. 1
Bajbouj M. 1
Grimm S. 1 2 3
Aust S. 1
1 Charité Universitätsmedizin , Klinik F. Psychiatrie U. Psychotherapie, Campus Benjamin Franklin, Berlin , Germany
2 MSB Medical Scool Berlin, Hochschule Für Gesundheit Und Medizin, Berlin , Germany
3 Department of Psychiatry, Psychotherapy and Psychosomatics, University Of Zurich , Psychiatric Hospital, Zurich, Switzerland, Zuerich , Switzerland
* Corresponding author.
Keywords: ketamine, major depressive disorder, Cognition–emotion interaction, Fronto-cingulate regions

Conflict of interest:

No

Article ID: EPV0472

Modulation of hippocampal functional connectivity and depressive symptom improvement following ECT

Takamiya A. *
Kishimoto T.
Hirano J.
Nakajima K.
Katayama N.
Kikuchi T.
Yamagata B.
Nakagawa A.
Mimura M.
Keio University School of Medicine , Department of Neuropsychiatry, Tokyo , Japan
* Corresponding author.
Keywords: Hippocampus, functional connectivity, Electroconvulsive Therapy, Depressive disorders

Conflict of interest:

No

Article ID: EPV0473

There are no changes in serum ciliary neurotrophic factor concentration in patients with melancholic depression under antidepressive therapy.

Uzbekov M. *
Shikhov S.
Moscow Research Institute of Psychiatry , Brain Pathology, Moscow , Russian Federation
* Corresponding author.
Keywords: ciliary neurotrophic factor, venlafaxine, blood-brain barrier, melancholic depression

Conflict of interest:

No

Article ID: EPV0477

A rating scale-derived anxious depression subtype does not predict treatment failure, response or remission in patients treated with ssris or placebo

Duicu M. 1 *
Hieronymus F. 2
Østergaard S. 3
1 Randers Regional Hospital, Psychiatry, Randers , Denmark
2 Aarhus University , Department of Clinical Medicine, Aarhus , Denmark
3 Aarhus University Hospital , Psychiatry, Department of Affective Disorders, Aarhus N, Denmark
* Corresponding author.
Keywords: SSRIs Anxious Depression Predictive Scale

Conflict of interest:

No

Article ID: EPV0478

Quality of life in major depressive disorder

Guermazi A. *
Smaoui N.
Omri S.
Lajmi I.
Feki R.
Maalej Bouali M.
Charfi N.
Zouari N.
Ben Thabet J.
Zouari L.
Maalej M.
Hedi Chaker University Hospital , Psychiatry C Department, Sfax , Tunisia
* Corresponding author.
Keywords: major depressive disorder, quality of life

Conflict of interest:

No

Article ID: EPV0479

Depressive disorder, anxiety, somatisation of cardiological impaitients

Kornetov N. 1 *
Molodykh O. 2
Zvereva N. 3 4
Arzhanik A. 3
1 Siberian State Medical University , Department of Psychiatry, Drug Addiction, Psychotherapy, Tomsk , Russian Federation
2 Siberian State Medical University , 4th Year Student Of The Medical Faculty, Tomsk , Russian Federation
3 Siberian States Medical Unifersity , 4th Year Student Of The Medical Faculty, Tomsk , Russian Federation
4 Federal state BUDGETARY INSTITUTION "SIBERIAN FEDERAL SCIENTIFIC and CLINICAL CENTER of the FEDERAL MEDICAL and BIOLOGICAL AGENCY" of Russia, Cardyology, Seversk , Russian Federation
* Corresponding author.
Keywords: Educational Program, Depressive disorder, comorbidity, Cardiologic inpathients

Conflict of interest:

No

Article ID: EPV0483
Subjects: Abstract, Eating Disorders

Life quality of patients with anorexia nervoza and bulimia nervoza.

Belokrylov I. *
Okonishnikova E.
Brukhin A.
Lazukova A.
Peoples' Friendship University of Russia ( RUDN University ), Department of Psychiatry And Medical Psychology, Moscow , Russian Federation
* Corresponding author.
Keywords: Anorexia nervosa and bulimia nervosa, Life quality

Article ID: EPV0484

Clinical manifestations of anorexia nervosa in patients with schizophrenia and schizotypal disorder.

Belokrylov I. *
Lineva T.
Brukhin A.
Kirsanova G.
Karnozov V.
Peoples' Friendship University of Russia ( RUDN University ), Department of Psychiatry And Medical Psychology, Moscow , Russian Federation
* Corresponding author.
Keywords: Anorexia nervosa, schizophrenia, schizotypal disorder, comorbidity

Article ID: EPV0487

Non bulimic shitty meal

Costa A. *
Borges J.
Macedo P.
Santos G.
Leite R.
Alcafache J.
Baixo Vouga´s Hospital Center - Aveiro, Department of Psychiatry And Mental Health, Aveiro , Portugal
* Corresponding author.
Keywords: Coprophagy, Cognitive impairment, pica, schizophrenia

Conflict of interest:

No

Article ID: EPV0492

Psychodiagnostic evaluation of obesity. Rorschach and bariatric surgery.

Galletta D. *
Micanti F.
Mastrola A.M.
Suarato V.
Department of Head-Neck Care Unit of Psychiatry and Psychology “Federico II” University Hospital Naples , Italy, Department of Head-neck Care Unit Of Psychiatry And Psychology “federico Ii” University Hospital Naples, Italy, Naples , Italy
* Corresponding author.

Conflict of interest:

No

Article ID: EPV0493

Treating ultra-orthodox young women with eating disorder in israel: culturally-sensitive interventions, difficulties, and dilemas.

Latzer Y. 1 *
Stein D. 2
Witztum E. 3
1 Haifa university , Social Welfare And Health Sciences, Haifa , Israel
2 Safra Children's Hospital, Chaim Sheba Medical Center, Tel Hashomer, Department of Psychiatry, Tel Hashomer, Israel
3 4Ben Gurion University of the Negev , Department of Psychiatry, Beer Sheva, Israel
* Corresponding author.
Keywords: religiosity, eating disorders, Culture, ultra-Orthodox

Conflict of interest:

No

Article ID: EPV0495

Food addiction in mexican population

Munguía L. 1 *
Valenciano Mendoza E. 1
Granero R. 2
Guzmán Saldaña R.M.E. 3
Jiménez-Murcia S. 4
Fazia G. 5
Mena-Moreno T. 6
Mora B. 6
Garcia F. 3
Gaspar A. 3
De La Cruz Mendez G. 3
Fernandez-Aranda F. 7
1 University of Barcelona , Clinical Sciences Department, School of Medicine, Barcelona , Spain
2 Universitat Autonoma de Barcelona , Departament De Psicobiologia I Metodologia De Les Ciències De La Salut, Barcelona , Spain
3 Autonomus University of the Hidalgo State , Psychology Academic Area, Pachuca , Mexico
4 University Hospital of Bellvitge , Department of Psychiatry, Hospitalet del Llobregat (Barcelona), Spain
5 University "Magna Graecia" of Catanzaro , Department of Medical And Surgical Sciences, Catanzaro , Italy
6 Bellvitge Universitary Hospital and CIBERobn , Psychiatry, Barcelona , Spain
7 University Hospital Bellvitge-IDIBELL and CIBERobn , Psychiatry, Barcelona , Spain
* Corresponding author.
Keywords: obesity, food addiction, Mexican population

Article ID: EPV0497

Motivation index as a prognosis indicator in bariatric surgery.

Nuñez Sevillano S. 1 *
Serrano García A. 2
González Rodríguez I. 3
García Vázquez P. 2
1 Complejo Asistencial Universitario de León , Psychiatry, León , Spain
2 Complejo Asistencial Universitario León , Psiquiatría, Leon , Spain
3 Complejo Asistencial Universitario de León , Psiquiatría, León , Spain
* Corresponding author.
Keywords: Motivation, bariatric surgery, prognosis

Conflict of interest:

No

Article ID: EPV0498

Disordered eating behavior and body image in junior medical students

Petunova S. 1 *
Lazareva E. 1
Zakharova A. 1
Hartfelder D. 1
Dulina G. 1
Petunova Y. 2
Nikolaev E. 3
1 Ulianov Chuvash State University , Social And Clinical Psychology Department, Cheboksary , Russian Federation
2 I.M. Sechenov First Moscow State Medical University , Faculty of Medicine, Moscow , Russian Federation
3 Ulianov Chuvash State University , Faculty of Medicine, Cheboksary , Russian Federation
* Corresponding author.
Keywords: eating behavior, body image, body dissatisfaction, medical students

Conflict of interest:

No

Article ID: EPV0500

The relationship between body mass index and internet problematic use, eating disturbances, sleep difficulties, and psychological distress in portuguese university students

Soares M.J. 1 *
Pereira A.T. 1
Maia B. 2
Gomes A. 3
Macedo A. 1
1 University of Coimbra , Faculty of Medicine, Department of Psychological Medicine, Coimbra , Portugal
2 The Catholic University of Portugal , Faculty of Philosophy And Social Sciences, Braga Regional Centre, Braga , Portugal
3 University of Coimbra , Faculty of Psychology And Educational Sciences, Coimbra , Portugal
* Corresponding author.
Keywords: Body mass index, sleep and eating disturbances, Internet problematic use, Psychological distress

Conflict of interest:

No

Article ID: EPV0501

The influence of family alcoholism on the development of eating disorders

Artemieva M. *
Suleimanov R.
Danilin I.
Arseniev A.
Shumeyko D.
Peoples' Friendship University of Russia ( RUDN University ), Department of Psychiatry And Medical Psychology, Moscow , Russian Federation
* Corresponding author.
Keywords: eating disorders, alcoholism, comorbidity

Conflict of interest:

No

Article ID: EPV0502

Correlation between catecholamine expression and clinical features of eating dissorders

Artemieva M. *
Danilin I.
Suleimanov R.
Lazukova A.
Shumeyko D.
Peoples' Friendship University of Russia ( RUDN University ), Department of Psychiatry And Medical Psychology, Moscow , Russian Federation
* Corresponding author.
Keywords: eating disorders, catecholamine

Conflict of interest:

No

Article ID: EPV0503

Cinematherapy group for patients with eating disorders.

De Frutos Guijarro J.J. 1 *
Chinchurreta N. 2
Martín Aragón R. 3
Moya Rodriguez C. 1
Moreno Menguiano C. 1
Garcia-Consuegra Colado D. 4
Calleja Alonso P. 4
1 Centro de Salud Doctor Luengo Rodriguez, Servicio De Psiquiatría, Mostoles , Spain
2 HOSPITAL PUERTA DE HIERRO, Psychiatry, MADRID , Spain
3 Complejo Hospitalario Mancha Centro., Unidad De Salud Mental Infanto-juvenil., Alcázar de San Juan. , Spain
4 Centro de Salud Coronel de Palma, Unidad De Trastornos De Conducta Alimentaria, Mostoles , Spain
* Corresponding author.
Keywords: cinematherapy, eating disorders, DAY HOSPITAL

Conflict of interest:

No

Article ID: EPV0504

Eating disorders during pregnancy, about 62 cases

Mnif D. 1 *
Sellami R. 2
1 HEDI CHAKER hospital, Psychiatric Service, SFAX , Tunisia
2 HEDI CHAKER hospital, Psychiatric Department, SFAX , Tunisia
* Corresponding author.
Keywords: eating disorder, Anxiety, Pregnancy, Psychiatry

Conflict of interest:

No

Article ID: EPV0508

Therapeutic approaches in night eating syndrome- a case series

Vasiliu O.
University Emergency Central Military Hospital Dr. Carol Davila , Psychiatry, Bucharest , Romania
Keywords: night eating syndrome, sertraline, fluoxetine, Eating Disorders

Article ID: EPV0512

Antidepressant therapeutic drug monitoring by minimally-invasive techniques in eating disorders patients: preliminary results from a pilote study

Mastellari T. 1 2 *
Di Gianni A. 1
Marasca C. 3
Protti M. 3
Mercolini L. 3
Atti A.R. 1
De Ronchi D. 1
1 University of Bologna , Department of Biomedical And Neuromotor Sciences, Bologna , Italy
2 University of Lille , Faculté De Médecine Henri Warembourg, Lille , France
3 University of Bologna , Research Group Of Pharmaco-toxicological Analysis (pta Lab), Department of Pharmacy And Biotechnology (fabit), Bologna , Italy
* Corresponding author.
Keywords: Antidepressant drugs, eating disorders, Therapeutic drug monitoring

Conflict of interest:

No

Article ID: EPV0514

Presence of not detected eating disorder prodromal symptoms in medical services in the year before to treatment start

Ruiz Guerrero F. *
Perez Fernandez M.
Losa Mujika E.
Benito Gonzalez P.
Calcedo Giraldo G.
Gonzalez Gómez J.
Gómez Del Barrio A.
Hospital Universitario Marqués de Valdecilla , Psychiatry, Santander , Spain
* Corresponding author.
Keywords: Prodromal Symptoms, Early Intervention, Time without treatment

Conflict of interest:

No

Article ID: EPV0515

Reduction of duration untreated eating disorder (DUed) in a sample of patients with an eating disorder.

Ruiz Guerrero F. *
Losa Mujika E.
Gándara Gutierrez C.
Benito Gonzalez P.
Calcedo Giraldo G.
Gonzalez Gómez J.
Gómez Del Barrio A.
Hospital Universitario Marqués de Valdecilla , Psychiatry, Santander , Spain
* Corresponding author.
Keywords: Duration Untreated eating disorder, Early protocol

Conflict of interest:

No

Article ID: EPV0517

Forced tube feeding in patients with life-threatening anorexia nervosa

Van Venrooij J. *
Godschalx-Dekker J.
Spaarne Gasthuis, Psychiatry, Haarlem , Netherlands
* Corresponding author.
Keywords: restrictive eating disorders, BMI &lt, 13 kg/m2, forced tube-feeding

Conflict of interest:

No

Article ID: EPV0518

Starving brain: the clinical challenge differentiating anorexia nervosa and psychosis - a case report

Almeida B. *
Almeida H.
Castro L.
Hospital de Magalhães Lemos, General Adult Psychiatry, Porto , Portugal
* Corresponding author.
Keywords: Anorexia nervosa, psychosis

Conflict of interest:

No

Article ID: EPV0519

Using routine outcome monitoring in eating disorders: first-year results of the itamited treatment model

Grau A. 1
Evans C. 2
Medina J. 2
Feixas G. 2 *
1 ITA salud mental, Clinical and Research, Barcelona , Spain
2 Universitat de Barcelona , Clinical Psychology And Psychobiology, Barcelona , Spain
* Corresponding author.
Keywords: anorexia, practice based evidence, bulimia, effectiveness study

Conflict of interest:

No

Article ID: EPV0520

The comorbidity of anorexia nervosa and borderline personality disorder: a case report and literature review.

Kabtni W. 1 *
Baatout A. 1
Ben Cheikh C. 1
Maamouri R. 2
El Kefi H. 1
Oumaya A. 1
1 military hospital of Tunis, Psychiatry Department, Tunis , Tunisia
2 Razi hospital, Psychiatry E, Ben arous , Tunisia
* Corresponding author.
Keywords: Borderline Personality Disorder, Anorexia nervosa, eating disorder

Conflict of interest:

No

Article ID: EPV0522
Subjects: Abstract, E-Mental Health

Can virtual exposure therapy with applied biofeedback help us treat social anxiety disorder? A feasibility study.

Ernst M. 1 *
Lichtenstein M.B. 2
Clemmensen L. 3
1 Mental Health Services in the Southern Region of Denmark, Centre For Telepsychiatry, Odense , Denmark
2 University of Southern Denmark , Department of Clinical Research, Odense , Denmark
3 Mental Health Services in the Southern Region of Denmark, Centre For Telepsychiatry, Odense C , Denmark
* Corresponding author.
Keywords: Social Anxiety Disorder, biofeedback, exposure therapy, virtual reality

Conflict of interest:

No

Article ID: EPV0523

Mindfulness-based program delivered through a smartphone app versus an in-person program in healthcare students: effectiveness in depressive symptoms

Garde González J. 1 *
López V. 2
Orosa-Duarte Á. 1
Mediavilla R. 3
Muñoz-Sanjose A. 4
Palao Á. 4
Bravo-Ortiz M.F. 3
Bayon C. 4
Rodríguez-Vega B. 3
1 La Paz University Hospital , Psychiatry, Clinical Psychology And Mental Health, Madrid , Spain
2 Autonomous University of Madrid (UAM) , School of Medicine, Madrid , Spain
3 La Paz University Hospital Research Health Institute (IdiPAZ) , Psychiatry, Clinical Psychology And Mental Health, Madrid , Spain
4 La Paz University Hospital Institute for Health Research (IdiPAZ) , Psychiatry, Clinical Psychology And Mental Health, Madrid , Spain
* Corresponding author.
Keywords: Mindfulness, Students, smartphone app, Dépression

Conflict of interest:

No

Article ID: EPV0524

Empathic, expressive, advanced virtual coach to improve independent healthy-life-years of the elderly (the empathic project: mid-term achievements)

González-Fraile E. 1 *
González-Pinto A. 2
Tenorio-Laranga J. 3
Fernández-Ruanova B. 3
Olaso J.M. 4
Montenegro C. 4
Santana R. 4
Vázquez A. 4
Justo R. 4
Lozano J. 4
Esposito A. 5
Cordasco G. 5
Troncone A. 5
Escalera S. 6
Palmero Cantariño C. 6
Schlögl S. 7
Petrovska-Delacretaz D. 8
Mtibaa A. 8
Deroo O. 9
Gordeeva O. 9
Chollet G. 10
Dugan N. 10
Irvine M. 10
Glackin N. 10
Pickard C. 10
Korsnes M. 11
Martinussen L. 11
Torres M.I. 4
1 Biomedical Research Networking Centre in Mental Health (CIBERSAM), Spain, Bioaraba Research Institute, Vitoria , Spain
2 University Hospital of Alava-Santiago , Department of Psychiatry, Vitoria , Spain
3 Osakidetza, Osatek, Bilbao , Spain
4 Universidad del País Vasco UPV/EHU , Science And Technology, Leioa , Spain
5 Università degli Studi della Campania , Computer Science And Applied Mathematics, Caserta , Italy
6 Universitat de Barcelona , Computer Vision Center, Barcelona , Spain
7 MCI Management Center Innsbruck, A, Innsbruck , Austria
8 Institut Mines-Telecom Evry, A , Paris , France
9 Acapela Group Mons, *, Belgium, Belgium ,
10 Intelligent Voice Ltd, *, London , United Kingdom
11 Oslo University Hospital , Department of Old Age Psychiatry, Oslo , Norway
* Corresponding author.
Keywords: Elders, Virtual Coach, Artificial Intelligence, Aging

Conflict of interest:

No

Article ID: EPV0527

Sexting practice and sexual orientation in young university students of the colombian caribbean

Muñoz Argel M.N. *
Arcos Guzman M.
Ruiz Gonzalez E.
Perea-Machado M.
Mera-Jimenez K.
Universidad Pontificia Bolivariana , Psicología, Monteria , Colombia
* Corresponding author.

Conflict of interest:

No

Article ID: EPV0532

Development and testing of a mobile application for identifying individuals at high risk for depression in the general population

Skugarevsky O. 1 *
Skuhareuskaya T. 2
Sokol A. 3
1 Belarusian State Medical University , Psychiatry And Medical Psychology, minsk , Belarus
2 Belarusian State Medical University , Psychiatry And Medical Psychology, Minsk , Belarus
3 Belarusian State Medical University , Human Anatomy, Minsk , Belarus
* Corresponding author.
Keywords: Dépression, mobile application, Screening

Conflict of interest:

No

Article ID: EPV0534

E-mental health in the population-based tromsø study

Wynn R. 1 *
Traver Salcedo V. 2
Bellika G. 1
1 UiT The Arctic University of Norway , Clinical Medicine, Tromso , Norway
2 Universitat Politecnica de Valencia , Itaca , Valencia , Spain
* Corresponding author.
Keywords: e-health, mental health, epidemiology

Conflict of interest:

No

Article ID: EPV0535

Smartphone use and mental health: pro and contra digitalisation

Nikolaev E.
Ulianov Chuvash State University , Department of Social And Clinical Pscyhology, Cheboksary , Russian Federation
Keywords: mental health, e-mental health, digitalisation, Smartphone

Conflict of interest:

No

Article ID: EPV0536

Enhancing the efficacy and adherence rate of a french unguided internet intervention for people struggling with the death of or separation from a loved one.

Debrot A. *
Efinger L.
Kheyar M.
Berthoud L.
Pomini V.
Université de Lausanne , Institute of Psychology, Lausanne , Switzerland
* Corresponding author.
Keywords: complicated grief, internet interventions, guidance

Conflict of interest:

No

Article ID: EPV0537

Testing the feasibility and efficacy of an unguided internet intervention for people who struggle to overcome the loss of a significant one.

Efinger L. *
Debrot A.
Pomini V.
Université de Lausanne , Institute of Psychology, Lausanne , Switzerland
* Corresponding author.
Keywords: Efficacy, bereavement, divorce, internet intervention

Conflict of interest:

No

Article ID: EPV0540

Psychosocial online counselling project in ukraine on ipso-care platform: final report

Korostiy V. 1 2 *
Missmail I. 3
Oleksandr Polishchuk O. 4
Penderetska O. 4
Krapivnyk G. 5
1 Kharkiv National Medical University , Department of Psychiatry, Narcology And Medical Psychology, Kharkiv , Ukraine
2 Kharkiv National Medical University , University Clinic, Kharkiv , Ukraine
3 IPSO, Main Office, Berlin , Germany
4 Bukovina State Medical University , Medical Psychology Center, Chernivtsi , Ukraine
5 Kharkiv National Pedagogical University , Department of English Philology, Kharkiv , Ukraine
* Corresponding author.
Keywords: IDPs, psychosocial online counseling, war affected population peoples in Ukraine, PTSD

Conflict of interest:

No

Article ID: EPV0541

How to successfully incorporate telepsychiatry into public mental health care system (scandinavian model)

Mucic D.
Psykiatrien Syd, Psychiatry, Vordingborg , Denmark
Keywords: Public Mental Health, attitudes, telepsychiatry service, quality of care

Conflict of interest:

No

Article ID: EPV0542

Psychotherapy using electronic media

Mucic D.
Little Prince Treatment Centre, E-mental Health Dpt., Copenhagen v , Denmark
Keywords: remote psychotherapy, e-mental health, online therapy, advantages vs disadvantages

Conflict of interest:

No

Article ID: EPV0550
Subjects: Abstract, Emergency Psychiatry

Epidemiological exploration of involuntary admissions during the last 10-year-period of economic crisis in greece

Bakola M. 1
Kitsou K.S. 1 *
Hyphantis T. 2
Gourzis P. 3
Jelastopulu E. 4
1 School of Medicine, University of Patras , Greece, Postgraduate Program Of Public Health, Patras , Greece
2 University Hospital of Ioannina , Department of Psychiatry, IOANNINA , Greece
3 School of Medicine, University of Patras , Greece, Psychiatry, Patras , Greece
4 School of Medicine, University of Patras , Greece, Public Health, Patras , Greece
* Corresponding author.
Keywords: Involuntary admissions, Compulsory admissions, Economic crisis, Public Health

Conflict of interest:

No

Article ID: EPV0553

Liver damage linked to quetiapine. Clinical case report.

Parra Gonzalez A. 1 *
Rentero Martin D. 1
Zamora Banegas B. 2
Santana Florido V. 2
1 Hospital 12 de Octubre, Psiquiatría, Madrid , Spain
2 Hospital 12 de Octubre, Psiquiatría, madrid , Spain
* Corresponding author.
Keywords: Liver injury, Quetiapine, acute liver failure, older population

Conflict of interest:

No

Article ID: EPV0554

The use of restraint and separation in aggressive and non-cooperative patients

Pavešić Radonja A. 1 *
Blazevic Zelic S. 2
Skarpa I. 2
1 University Hospital Rijeka , Psychiatry, Rijeka , Croatia
2 Clinical hospital Rijeka, Psychiatry Department, Rijeka , Croatia
* Corresponding author.
Keywords: restriction and separation, aggressive patient, Emergency Psychiatry

Conflict of interest:

No

Article ID: EPV0556

Physical restraint in psychiatric emergencies

Santana Florido V. *
Eguía Barbarin J.L.
Rentero Martin D.
Parra Gonzalez A.
Zamora Banegas B.
Hospital 12 de Octubre, Psiquiatría, Madrid , Spain
* Corresponding author.
Keywords: physicalretraints, psychiatricemergencies

Conflict of interest:

No

Article ID: EPV0558

First-episode of cannabis-induced psychosis in a young adult

Solis M.O. *
Valverde Barea M.
Soldado Rodriguez L.
COMPLEJO HOSPITALARIO DE JAEN, Psiquiatria, JAEN , Spain
* Corresponding author.
Keywords: First-Episode, cannabis, psychosis

Conflict of interest:

No

Article ID: EPV0559

Psychotic symptoms in steroid-induced mania. Clinical features and treatment. Case report

Del Sol Calderón P. 1 *
Izquierdo De La Puente Á. 1
Garcia Moreno M. 1
Rodriguez De Lorenzo M. 2
Carrajo Garcia C. 2
1 HOSPITAL UNIVERSITARIO PUERTA DE HIERRO MAJADAHONDA , Psychiatry, MADRID , Spain
2 Hospital Universitario Ramon y Cajal , Psychiatry, Madrid , Spain ,
3 HOSPITAL UNIVERSITARIO RAMON Y CAJAL , Psychiatry, MADRID , Spain
* Corresponding author.
Keywords: steroid, mania, Psychotic Symptoms

Conflict of interest:

No

Article ID: EPV0562

Medical-psychological and neuropsychiatric maintenance system to the anti-terrorist operation / joint force operation combatants in multidisciplinary clinic

Bazyka D. 1
Loganovsky K. 2 *
Sushko V. 3
Chumak A. 1
Yaroshenko Z. 4
Zaitseva A. 4
Zdorenko L. 2
Zdanevich N. 2
Gresko M. 2
Vasilenko Z. 2
Kravchenko V. 2
Drozdova N. 2
1 State Institution “National Scientific Center for Radiation Medicine of the National Academy of Medical Sciences of Ukraine”, Clinical Immunology, Kyiv , Ukraine
2 State Institution "National Research Centre for Radiation Medicine of National academy of Medical Sciences of Ukraine, Department of Radiation Psychoneurology, Institute of Clinical Radiology, Kyiv , Ukraine
3 State Institution “National Scientific Center for Radiation Medicine of the National Academy of Medical Sciences of Ukraine”, Division For Medical Expertise And Treatment Of Ionizing Radiation Consequences, Kyiv , Ukraine
4 State Institution “National Scientific Center for Radiation Medicine of the National Academy of Medical Sciences of Ukraine”, Clinic, Kyiv , Ukraine
* Corresponding author.
Keywords: PTSD, Anti-Terrorist Operation, biopsychosocial paradigm, complex psychosocial, medical, and neuropsychiatric interventions

Conflict of interest:

No

Article ID: EPV0563

Personality patterns of anti-terrorist operation / joint force operation combatants in comparison with clean-up workers of the chornobyl catastrophe, ukraine

Gresko M.
Loganovsky K. *
State Institution "National Research Centre for Radiation Medicine of National academy of Medical Sciences of Ukraine, Department of Radiation Psychoneurology, Institute of Clinical Radiology, Kyiv , Ukraine
* Corresponding author.
Keywords: Anti-Terrorist Operation, Chornobyl catastrophe, Personality pattern, Psychophysiology

Conflict of interest:

No

Article ID: EPV0564

Dealing with fire: a portuguese model of intervention for victims of traumatic experiences

Martins Correia J. *
Jesus B.
Caetano S.
Hospital Sousa Martins - Unidade Local de Saúde da Guarda , Departamento De Psiquiatria E Saúde Mental, Guarda , Portugal
* Corresponding author.
Keywords: traumatic experiences, Psychological Trauma, prevention of mental health, Natural disasters

Conflict of interest:

No

Article ID: EPV0567
Subjects: Abstract, Epidemiology and Social Psychiatry

Low cognitive reserve is associated with excess mortality: evidence from a spanish nationally representative study

Lara E. 1
Miret M. 2
Moreno D. 2
Martín-María N. 2
Olaya B. 3
Haro J.M. 3
Ayuso Mateos J.L. 1 *
1 Hospital Universitario de La Princesa , Psychiatry, Madrid , Spain
2 Universidad Autónoma de Madrid , Psychiatry, Madrid , Spain
3 Parc Sanitari Sant Joan de Déu , Research, Innovation And Teaching Unit, Barcelona , Spain
* Corresponding author.
Keywords: cognitive reserve, mortality, population-based study

Conflict of interest:

No

Article ID: EPV0568

The association between loneliness and suicidal ideation in a spanish representative sample

Castelletti C. 1
Lara E. 2
Miret M. 1
Olaya B. 3
Haro J.M. 3
Ayuso Mateos J.L. 2 *
1 Universidad Autónoma de Madrid , Psychiatry, Madrid , Spain
2 Hospital Universitario de La Princesa , Psychiatry, Madrid , Spain
3 Parc Sanitari Sant Joan de Déu, Research, Innovation And Teaching Unit, Barcelona , Spain
* Corresponding author.
Keywords: Suicidal ideation, Dépression, Population based study, Loneliness

Conflict of interest:

No

Article ID: EPV0570

Patients profile of a tunisian child psychiatry department

Behi F.
Bourgou S. *
Hamza M.
Charfi F.
Belhadj A.
Mongi Slim Hospital , Child Psychiatry Department, Tunis , Tunisie , Tunisia
* Corresponding author.
Keywords: Children, Psychiatry, adolescent, epidemiology

Conflict of interest:

No

Article ID: EPV0572

Clinical characteristics and implication of the patient in the choice of treatment with monthly or quarterly paliperidone palmitate

Esparrago Llorca G. 1 *
Jerez Barroso F.J. 2
Juncosa Montes P. 1
Bordes Giménez M.D. 2
Díaz Fernández F. 2
Andrés Pereira G. 2
Leones Gil E.M. 3
Delgado Rastrollo E. 4
García Navarro M. 5
1 Servicio Extremeño de Salud , Equipo De Salud Mental , Cáceres , Spain
2 Servicio Extremeño de Salud, Hospital San Pedro De Alcántara , Cáceres , Spain
3 Servicio Vasco de Salud , Hospital Universitario De Basurto , Bilbao , Spain
4 Servicio Vasco de Salud , Red De Salud Mental De Bizcaia, Bilbao , Spain
5 Servicio Extremeño de Salud , Hospital De Mérida , Mérida , Spain
* Corresponding author.
Keywords: choice of treatment, characteristics, paliperidone palmitate

Conflict of interest:

No

Article ID: EPV0574

Prevalence of pervasive developmental disorders in medico-administrative databases, france 2010-2017

Ha C. 1 *
Chin F. 2
Chan-Chee C. 3
1 Santé publique France , Non-communicable Diseases And Trauma Division , Saint-Maurice Cedex , France
2 Santé publique France , Direction Appui, Traitement Et Analyse De Données, Saint-Maurice , France
3 Santé publique France , Non-communicable Diseases And Trauma Division , Saint-Maurice , France
* Corresponding author.
Keywords: Autism, Pervasive developmental disorders, prevalence, France

Conflict of interest:

No

Article ID: EPV0578

Changes in attitudes toward mental illness in health care professionals and students

Lien Y.-J. 1 *
Lin H.-S. 1 2
Lien Y.-Y. 1
Tsai C.-H. 1
Wu T.-T. 1
1 National Taiwan Normal University , Department of Health Promotion And Health Education, Taipei , Taiwan
2 National Taiwan Normal University , Department of Health Promotion And Health Education, Taipei City , Taiwan
* Corresponding author.
Keywords: Stigma, Healthcare providers, mental illness, Attitude

Conflict of interest:

No

Article ID: EPV0579

Structural competency in psychiatry: going beyond the basics. A case report

Maini R. *
Montefiore Medical Center, Psychiatry, Bronx , United States of America
* Corresponding author.
Keywords: Structural Competency, Culture, Politics

Conflict of interest:

No

Article ID: EPV0580

Increased hospitalization for all psychiatric disorders, mood disorders and alcohol use disorders in italy following the great recession

Mattei G. *
Pistoresi B.
Addabbo T.
University of Modena and Reggio Emilia , Department of Economics, Modena , Italy
* Corresponding author.
Keywords: economic crisis, epidemiology, Great Recession, hospital admissions

Conflict of interest:

No

Article ID: EPV0581

On social psychiatry: resident houses, Part I – theoretical approaches

Neu E. 1 *
Michailov M. 2
Welscher U. 2
Birkenbihl P. 2
Neu R. 2
Foltin V. 3
Holler M. 4
Weber G. 5
1 Inst. Umweltmedizin (IUM) c/o ICSD/IAS e.V. POB 340316 , 80100 Muenchen, Germany (Int.Council Sci.Develop./Int. Acad.Sci. Berlin-Innsbruck-Muenchen-NewDelhi-Paris-Sofia-Vienna) , Pharmacophysiology, München, Germany
2 Inst. Umweltmedizin (IUM) c/o ICSD/IAS e.V. POB 340316 , 80100 Muenchen, Germany (Int.Council Sci.Develop./Int. Acad.Sci. Berlin-Innsbruck-Muenchen-NewDelhi-Paris-Sofia-Vienna) , Pharmacophysiology, Muenchen, Germany
3 St. Elizabeth Univ ., Of Health & Social Work, Bratislava , Slovak Republic
4 Univ. Hamburg , Fac. Economics (ex-dean), Hamburg , Germany
5 Univ. Lxbg. & Vienna , Fac. Psychology (ex-dean), Vienna , Austria
* Corresponding author.
Keywords: resident houses, social physiology, social psychiatry, UNO-Agenda 21

Conflict of interest:

No

Article ID: EPV0584

Burnout in the albanian population.

Skorovoti Z.
Mother Theresa Hospital , Neuroscence, Tirane , Albania
Keywords: work-related stress, exhaustion in the workplace, mentally-challenged employers., Burnout

Conflict of interest:

No

Article ID: EPV0585

Analysis of the causes of frequent and super-frequent hospitalizations in the department of the first psychotic episode

Usova A.
Omsk State Medical University , Psychiatry And Medical Psychology, Оmsk , Russian Federation
Keywords: schizophrenia, frequent hospitalizations, first-episode psychosis

Conflict of interest:

No

Article ID: EPV0586

Syndromes that causes the level of schizophrenia external stigma in students

Vasilchenko K. *
Ismagilova V.
Shnitsar N.
Omsk State Medical University , Psychiatry And Medical Psychology, Omsk , Russian Federation
* Corresponding author.
Keywords: Students, external stigma, Stigma, schizophrenia

Conflict of interest:

No

Article ID: EPV0587

A descriptive comparison between population served by united states of america and spain mental health centers.

Carracedo Sanchidrián D. 1 *
Batalla Monedero M. 2
Rodríguez Soria E. 3
1 Hospital Universitario la Paz, Mental Health , Madrid , Spain
2 Hospital La Fe , Psychiatry, Valencia , Spain
3 Hospital Universitario de Fuenlabrada, Mental Health , Fuenlabrada , Spain
* Corresponding author.
Keywords: mental health, Health system, public health, Cultural impact

Conflict of interest:

No

Article ID: EPV0588

Relationship between safety perception and community participation in victims of the armed conflict in a municipality of the colombian caribbean

Muñoz Argel M.N. 1 *
Arcos Guzman M. 1
Vélez Carvajal J. 2
Forero Ospina P. 3
Quintana Fernandez M. 2
1 Universidad Pontificia Bolivariana , Psicología , Monteria , Colombia
2 Universidad Pontificia Bolivariana , Social Communication , Monteria , Colombia
3 Universidad Pontificia Bolivariana , Formación Humanista, Monteria , Colombia
* Corresponding author.
Keywords: Safety perception, Community participation, Victims, Armed conflict

Conflict of interest:

No

Article ID: EPV0593

Dementia and alzheimer’s disease in the czech republic – what can be expected in the coming years?

Benešová K. *
Jarkovsky J.
Pokorná A.
Soukupova J.
Institute of Health Information and Statistics of the Czech Republic , -, Prague , Czech Republic
* Corresponding author.
Keywords: dementia, health insurance data, Alzheimer’s disease, national registry

Conflict of interest:

No

Article ID: EPV0595

Incidence of disability for mental illness and behavior disorders

Čeledová L. *
Čevela R.
Odlozilik R.
Pastircakova T.
Charles University Faculty of Medicine in Pilsen, Social And Assessment Medicine, Pilsen , Czech Republic
* Corresponding author.
Keywords: Medical Assessment Service, disability, Long-term adverse medical condition

Conflict of interest:

No

Article ID: EPV0596

A novel approach to assessing the size and duration of the impact of life events on affective and cognitive wellbeing

Glozier N. 1 *
Morris R. 2
Kettlewell N. 3
Cobb-Clarke D. 3
Cripps S. 2
1 Prof, Brain And Mind Centre, Camperdown, Australia
2 University of Sydney , Brain And Mind Centre, Camperdwon , Australia
3 University Of Sydney , Economics, Sydney , Australia
* Corresponding author.
Keywords: Cohort, Life events, epidemiology, Wellbeing

Conflict of interest:

No

Article ID: EPV0598

Social exclusion risk factors associated with psychiatric disorders among the young population

Ladea M. 1 2 *
Patrascu M.R. 1
Sofia A. 1 2
Bran M. 3
Ionescu T.C. 1 2
1 Alexandru Obregia Clinical Hospital , Department 3, Bucharest , Romania
2 University of Medicine and Pharmacy’’ Carol Davila’’ , Psychiatry, Bucharest , Romania
3 Coltea Hospital Bucharest , Psychiatry, Bucharest , Romania
* Corresponding author.
Keywords: social exclusion, young population, occupational status, medically insurance

Conflict of interest:

No

Article ID: EPV0601

Recover-e project in bulgaria. past, present and future of the mobile psychiatric teams.

Nakov V. 1 *
Hinkov H. 1
Zarkov Z. 1
Dimitrov P. 1
Dinolova R. 1
Popova A. 2
Dzhisova A. 2
1 National Center of Public Health and Analyses, Mental Health , Sofia , Bulgaria
2 Mental Health Center .Shipkovensky, Mental Health , Sofia , Bulgaria
* Corresponding author.
Keywords: RECOVER-E, Mobile, FACT

Article ID: EPV0604

The importance of treating early emotional trauma for the recovery of persons with experience of psychosis

Strkalj Ivezic S. 1 *
Britvic D. 2
Radic K. 1
1 University psychiatric hospital Vrapce , Social Psychiatry , Zagreb , Croatia
2 University Department of Psychiatry, Social Psychiatry , Split , Croatia
* Corresponding author.
Keywords: psychosis, trauma, psychosocial interventions

Conflict of interest:

No

Article ID: EPV0610
Subjects: Abstract, Ethics and Psychiatry

The level of suicide behavior among transgender people within ukrainian population

Lahutina S. 1 *
Subbota S. 2
1 Bogomolets National Medical University , Institute of Cognitive Modeling, Medical Psychology, Psychosomatic Medicine and Psychotherapy Department, Kyiv , Ukraine
2 Institute of Cognitive Modeling, Scientific Department, Kyiv , Ukraine
* Corresponding author.
Keywords: stigmatization, suicide behavior, transgender, preventive interventions

Conflict of interest:

No

Article ID: EPV0611

Foundations of bioethics and mental health model assistance in the city of piauí

Luz L. 1 *
Rutherford V. 2
Marques Filho E. 3
1 UFPI, Medicine , Teresina , Brazil
2 FMUSP, Medicine, Sao Paulo , Brazil
3 Facid, Medicine, Teresina , Brazil
* Corresponding author.
Keywords: mental health, Bioethics, ethics, Social Responsability

Conflict of interest:

No

Article ID: EPV0613

Coercive measures in our context: a narrative review on its uses

Nocete Navarro L. 1 *
López De Loma Osorio V. 2
Borrero Granell L. 3
Bravo-Ortiz M.F. 4
Fernández Liria A. 2
1 Universidad Autónoma de Madrid , Psychiatry, Madrid , Spain
2 Hospital Universitario Príncipe de Asturias , Psychiatry, Alcalá de Henares , Spain
3 Hospital Universitario Nuestra Señora de Candelaria , Psychiatry, Santa Cruz de Tenerife , Spain
4 La Paz University Hospital Research Health Institute (IdiPAZ) , Psychiatry, Clinical Psychology And Mental Health , Madrid , Spain
* Corresponding author.
Keywords: coercive measures, western countries, mechanical restraint, involuntary hospitalisation

Conflict of interest:

No

Article ID: EPV0614

A review of the sociodemographic, clinical and other contextual factors associated with coercive measures

Nocete Navarro L. 1 *
Borrero Granell L. 2
López De Loma Osorio V. 3
Fernández Liria A. 3
Bravo-Ortiz M.F. 4
1 Universidad Autónoma de Madrid , Psychiatry, Madrid , Spain
2 Hospital Universitario Nuestra Señora de Candelaria , Psychiatry, Santa Cruz de Tenerife , Spain
3 Hospital Universitario Príncipe de Asturias , Psychiatry, Alcalá de Henares , Spain
4 La Paz University Hospital Research Health Institute (IdiPAZ) , Psychiatry, Clinical Psychology And Mental Health , Madrid , Spain
* Corresponding author.
Keywords: coercive measures, Sociodemographic factors, clinical factors

Conflict of interest:

No

Article ID: EPV0615

The impact of coercive measures: are we blind to the obvious?

Nocete Navarro L. 1 *
Borrero Granell L. 2
López De Loma Osorio V. 3
Fernández Liria A. 3
Bravo-Ortiz M.F. 4
1 Universidad Autónoma de Madrid , Psychiatry, Madrid , Spain
2 Hospital Universitario Nuestra Señora de Candelaria , Psychiatry, Santa Cruz de Tenerife , Spain
3 Hospital Universitario Príncipe de Asturias , Psychiatry, Alcalá de Henares , Spain
4 La Paz University Hospital Research Health Institute (IdiPAZ) , Psychiatry, Clinical Psychology And Mental Health , Madrid , Spain
* Corresponding author.
Keywords: trauma informed care, coercive measures, ethical challenges

Conflict of interest:

No

Article ID: EPV0617

Cross-cultural differences in attitudes toward euthanasia among russian and uzbek medical students

Nikolaev E. 1 *
Aleksandrov A. 2
Poverinov I. 3
Niyazov L. 4
Safarova N. 4
Gulamova M. 4
1 Ulianov Chuvash State University , Department of Social And Clinical Pscyhology, Cheboksary , Russian Federation
2 Ulianov Chuvash State University , Department of Public Law, Cheboksary , Russian Federation
3 Ulianov Chuvash State University , Department of Philosophy, Sociology And Pedagogy, Cheboksary , Russian Federation
4 Bukhara State Medical Institute , Medical Chemistry Department, Bukhara , Uzbekistan
* Corresponding author.
Keywords: ethics, attitudes toward euthanasia, cross-cultural issues, medical students

Conflict of interest:

No

Article ID: EPV0618

Beliefs and attitudes toward supervision in CBT and competencies of the trainees in cognitive behavioral training

Prasko J. 1 *
Ociskova M. 1
Slepecky M. 2
Kotianova A. 2
1 Faculty of Medicine and Dentistry, University Palacky Olomouc , Department of Psychiatry, Olomouc , Czech Republic
2 Faculty of Social Science and Health Care - Constantine the Philosopher University in Nitra- Slovak Republic ,, Department of Psychology Sciences, Nitra , Slovak Republic
* Corresponding author.
Keywords: supervision, cognitive behavioral therapy, competencies, beliefs and attitudes

Conflict of interest:

No

Article ID: EPV0622

Un convention on the rights of persons with disabilities (CRPD): relevance for training and practice of psychiatry

StrkaljIvezic S. *
University psychiatric hospital Vrapcetal Vrapce , Social Psychiatry , Zagreb , Croatia
* Corresponding author.
Keywords: Human rights, UN convention, training

Conflict of interest:

No

Article ID: EPV0625
Subjects: Abstract, Forensic Psychiatry

New approaches to the problem of domestic violence against women in russia

Kachaeva M. *
Skibina N.
Nazarova L.
Kharitonova N.
Sechenov First Moscow State Medical University , Chair Of Forensic Psychiatry, Moscow , Russian Federation
* Corresponding author.
Keywords: women, violence, homicide, Dépression

Conflict of interest:

No

Article ID: EPV0627

The use of psychometric scales in assessing the effectiveness of the prevention of recurrent dangerous actions of persons with mental disorders

Makushkina O. *
Sharabidze N.
Federal State Budgetary Institution “V.P. Serbsky National Medical Research Centre for Psychiatry and Narcology” of the Ministry of Health of the Russian Federation , Department of Forensic Psychiatric Prevention, Moscow , Russian Federation
* Corresponding author.
Keywords: psychometric methods, assessing the effectiveness, recurrent dangerous acts, Prevention

Conflict of interest:

No

Article ID: EPV0628

Neuropsychological assessment and forensic evaluation of patients with different dementia types

Novotni G. 1
Novotni A. 2 *
Bajraktarov S. 2
Milutinović M. 3
Novotni L. 2
1 University Clinic of Neurology , Neurology , Skopje , North Macedonia
2 University Clinic of Psychiatry , Psychiatry, Skopje , North Macedonia
3 University Clinic of Psychiatry Skopje , Department For Affective Disorders, Skopje , North Macedonia
* Corresponding author.
Keywords: dementia, testamentary capacity, neuropsychological assessment

Conflict of interest:

No

Article ID: EPV0630

Capgras delusion in a forensic psychiatric setting - case report

Sendula Jengic V. *
Rab Psychiatric Hospital , Department For Forensic Psychiatry, Rab , Croatia
* Corresponding author.
Keywords: Capgras syndrome, forensic psychiatry, matricide, Mental Health Act

Conflict of interest:

No

Article ID: EPV0636

Hesitation wounds and sharp force injuries in forensic psychiatry

Karakasi M.V. 1 *
Pavlidis P. 2
Vlachaki A. 3
1 AHEPA University General Hospital , 3rd Psychiatry Department, Thessaloniki , Greece
2 Democritus University of Thrace , School of Medicine, Laboratory of Forensic Sciences , Alexandroupolis , Greece
3 G. Papanikolaou General Hospital of Thessaloniki , Adult Psychiatry, Psychiatric Department, Thessaloniki , Greece
* Corresponding author.
Keywords: Suicide, hesitation wounds, Self-harm, Forensic Psychiatry

Conflict of interest:

No

Article ID: EPV0637

Underlying motives for perpetrators and categories of victims of sexual homicide

Karakasi M.V. 1 *
Pavlidis P. 1
Vlachaki A. 2
1 Democritus University of Thrace , School of Medicine, Laboratory of Forensic Sciences , Alexandroupolis , Greece
2 G. Papanikolaou General Hospital of Thessaloniki , Adult Psychiatry, Psychiatric Department, Thessaloniki , Greece
* Corresponding author.
Keywords: Motives, violence, victims, homicide

Conflict of interest:

No

Article ID: EPV0638

The evaluation of a weight reduction programme for forensic psychiatric in-patients

Remfeldt D.
Sen P. *
Kings College London , Institute of Psychology, Psychiatry And Neuroscience, Denmark Hill, United Kingdom
* Corresponding author.
Keywords: Exercise, Psychological health, inpatient, physical health

Conflict of interest:

No

Article ID: EPV0639

Title: sociodemographic, clinical and employment profile of patients who sought fitness to rejoin job: a retrospective study

Sr P. 1 *
Gowda G. 2
Math S. 2
Kumar C. 2
1 MANDYA INSTITUTE OF MEDICAL SCIENCES , Psychiatry, MANDYA , India
2 NATIONAL INSTITUTE OF MENTAL HEALTH AND NEURO-SCIENCES , Psychiatry, BANGALORE , India
* Corresponding author.
Keywords: Fitness for Job, Neuro-psychiatry, Absenteeism, Job Profile

Conflict of interest:

No

Article ID: EPV0640

Implementing peer support work into a forensic hospital

Walde P. *
Tomlin J.
Völlm B.
Universitätsmedizin Rostock , Klinik Für Forensische Psychiatrie, Rostock , Germany
* Corresponding author.
Keywords: Forensic Mental Health, forensic psychiatry, Peer Suppor Work

Conflict of interest:

No

Article ID: EPV0641

Clinical profile of homicide and homicide attempt offenders with schizophrenia

Chaari I. *
Omri S.
Abid W.
Smaoui N.
Feki R.
Maalej Bouali M.
Ben Thabet J.
Charfi N.
Zouari L.
Maalej M.
Hedi Chaker University Hospital , Psychiatry C Department, Sfax , Tunisia
* Corresponding author.
Keywords: forensic psychiatric assessment, homicide, homicide attempt, schizophrenia

Conflict of interest:

No

Article ID: EPV0642

Characteristics of sex offenders examined in a forensic psychiatric assessment

Chaari I. *
Omri S.
Abid W.
Smaoui N.
Feki R.
Charfi N.
Maalej Bouali M.
Ben Thabet J.
Zouari L.
Maalej M.
Hedi Chaker University Hospital , Psychiatry C Department, Sfax , Tunisia
* Corresponding author.
Keywords: sex offenders, forensic psychiatric assessment

Conflict of interest:

No

Article ID: EPV0643

Marital homicide: epidemiological and clinical issues

Guermazi A. *
Omri S.
Chaari I.
Feki R.
Smaoui N.
Maalej Bouali M.
Zouari L.
Charfi N.
Ben Thabet J.
Maalej M.
Hedi Chaker University Hospital , Psychiatry C Department, Sfax , Tunisia
* Corresponding author.
Keywords: Marital homicide, sociodemographic and clinical profile, criminal psychiatric expertise

Conflict of interest:

No

Article ID: EPV0644

Legal involvement in a case of hoarding disorder

Martins Correia J. *
Jesus B.
Caetano S.
Hospital Sousa Martins - Unidade Local de Saúde da Guarda , Departamento De Psiquiatria E Saúde Mental, Guarda , Portugal
* Corresponding author.
Keywords: legislation, legal problems, public health, hoarding disorder

Conflict of interest:

No

Article ID: EPV0648
Subjects: Abstract, Genetics & Molecular Neurobiology

The importance of presence or absence of the cag duplication

Kocijancic Azzaoui S. 1 *
Rus Prelog P. 2
1 Splošna bolnišnica Novo mesto , Psihiatrija , Novo mesto , Slovenia
2 University Psychiatric Clinic Ljubljana , Psychiatry, Ljubljana , Slovenia
* Corresponding author.
Keywords: tardive dystonia, risperidone, Huntington’s disease, court ordered hospitalisation

Conflict of interest:

No

Article ID: EPV0651

Epigenome editing of the potential enhancers of the schizophrenia risk genes

Abashkin D.
Kondratyev N.
Golov A.
Karpov D.
Smirnova S.
Lezheiko T. *
Golimbet V.
Mental Health Research Center , Clinical Genetics, Moscow , Russian Federation
* Corresponding author.
Keywords: enhancers, CRISPR/SpyCas9 repressor system, schizophrenia, genome editing

Conflict of interest:

No

Article ID: EPV0652

Exploring the relationship of DNA sequence and methylation within the NDUFA13/ YJEFN3 genes with cognitive deficits in schizophrenia

Alfimova M.
Kondratyev N.
Golov A.
Lezheiko T. *
Golimbet V.
Mental Health Research Center , Clinical Genetics, Moscow , Russian Federation
* Corresponding author.
Keywords: NDUFA13/ YJEFN3 genes, schizophrenia, cognitive deficit, methylation

Conflict of interest:

No

Article ID: EPV0653

A SEARCH FOR GENETIC VARIATIONS ASSOCIATED WITH NEGATIVE SYMPTOMS OF SCHIZOPHRENIA

Golimbet V. 1
Lezheiko T. 2 *
Romanov D. 3
Alfimova M. 1
Korovaitseva G. 1
1 Mental Health Research Center , Clinical Genetics, Moscow , Russian Federation
2 Mental Health Research Center , I.M. Sechenov First Moscow State Medical University , Clinical Genetics, Moscow , Russian Federation
3 I.M. Sechenov First Moscow State Medical University , Mental health research center, Department of Psychiatry And Psychosomatics, Moscow, Russian Federation
* Corresponding author.
Keywords: negative symptoms, factor structure, genetic factors, schizophrenia

Conflict of interest:

No

Article ID: EPV0655

Attitudes among south african university employees and students towards disclosing secondary genetic findings

Spies G. 1 *
Mokaya J. 2
Steadman J. 3
Schuitmaker N. 4
Kidd M. 5
Hemmings S. 3
Carr J. 6
Kuivaniemi H. 7
Seedat S. 3
1 Stellenbosch University , Department of Psychiatry, Cape Town , South Africa
2 Hertford College , University of Oxford , Nuffield Department of Medicine, Oxford , United Kingdom
3 Stellenbosch University , Psychiatry, Cape Town , South Africa
4 Stellenbosch University , Pychiatry, Cape Town , South Africa
5 Stellenbosch University , Centre For Statistical Consultation, Department of Statistics And Actuarial Sciences, Stellenbosch , South Africa
6 Stellenbosch University , 5division Of Neurology, Faculty of Medicine and Health Sciences, Cape Town , South Africa
7 Stellenbosch University , 3division Of Molecular Biology And Human Genetics, Cape Town , South Africa
* Corresponding author.
Keywords: Secondary findings, genetics, Return of results, South Africa

Conflict of interest:

No

Article ID: EPV0656

Physiological effect of glutathione from oxidative stress induced by cadmium in rats

Jovanovic Mirkovic J. 1
Alexopoulos C. 1 *
Kocic G. 2
Miljkovic N. 2
Jurinjak Z. 1
1 The Academy of Applied Preschool Teaching and Health Studies , Medicine, Cuprija , Serbia
2 Faculty of Medicine, Biochemistry, Nis, Serbia, 3 General Hospital , Medicine, Cuprija, Serbia
* Corresponding author.
Keywords: glutathione, brain, oxidative stress, Cadmium

Conflict of interest:

No

Article ID: EPV0657

Monitoring the protective physiological role of α-lipoic acid via malondialdehyde concentrations in the brain of rats

Jovanovic Mirkovic J. 1
Alexopoulos C. 1 *
Kocic G. 2
Miljkovic N. 3
1 The Academy of Applied Preschool Teaching and Health Studies , Medicine, Cuprija , Serbia
2 Faculty of Medicine, Biochemistry , Nis , Serbia
3 General Hospital , Medicine, Cuprija , Serbia
* Corresponding author.
Keywords: Copper, α-lipoic acid, brain, malondialdehyde

Conflict of interest:

No

Article ID: EPV0660

Linking cell cycle to mitochondria in schizophrenia: a case-control study using olfactory neuroepithelium-derived neural progenitor cells

Idotta C. 1 *
Tibaldi E. 2
Pagano M. 2
Favaretto N. 1
Cazzador D. 1
Peruzzo R. 3
Solmi M. 1
Martini A. 1
Leanza L. 3
Favaro A. 1
Brunati A.M. 2
Toffanin T. 4
1 Padova Neuroscience Center, University of Padova , Department of Neurosciences, Padova , Italy
2 University of Padova , Molecular Medicine, Padova , Italy
3 Univeristy of Padova , Department of Biology, Padova , Italy
4 Azienda Ospedaliera di Padova , Psychiatry Clinic , Padova , Italy
* Corresponding author.
Keywords: schizophrenia, translational psychiatry, mitochondria, Wnt/Beta-catenin

Conflict of interest:

No

Article ID: EPV0661

Cnv in silver nanoparticles-primed hyperactive rats

Ishido M.
National Institute for Environmnetal Studies , Center for Environ Risk/health Res, Tsukuba , Japan
Keywords: hyperactivity, silver nanoparticles, copy number variants

Conflict of interest:

No

Article ID: EPV0663
Subjects: Abstract, Guidelines - Guidance

Thromboembolic events in patients with mental disorders. An overlooked and potentially life-threatening treatment related complication.

Bouras P. 1
Argitis P. 2 *
Koukouras T. 2
Karavia S. 2
Garnelis D. 2
Soueref E. 1
Chaviaras Z. 2
1 General Hospital of Corfu , Intrnal Merdicine , Corfu , Greece
2 General Hospital of Corfu , Psychiatric, Corfu , Greece
* Corresponding author.
Keywords: thromboembolitic, antipsychotic

Conflict of interest:

No

Article ID: EPV0667
Subjects: Abstract, Intellectual Disability

Unusual suicide attempt in intellectual deficiency: a case report

Kabtni W. 1 *
Baatout A. 2
Ben Cheikh C. 2
Rebai A. 3
Elkefi H. 2
Oumaya A. 4
1 military hospital of Tunis , Psychiatry Department, Tunis , Tunisia
2 Military hospital of Tunis , Psychiatry Department, Tunis , Tunisia
3 Razi University Hospital , Adult Outpatient Psychiatry Department, Mannouba , Tunisia
4 military hospital of tunis , Psycihiatric Unit , tunis , Tunisia
* Corresponding author.
Keywords: Suicide, intellectual deficiency, Dépression, metals ingestion

Conflict of interest:

No

Article ID: EPV0668

Prevalence and patterns of psychotropic medication treatment in adults with intellectual disabilities in germany

Schützwohl M. *
Koch A.
Dobrindt J.
TU Dresden, Psychiatry And Psychotherapy, Dresden, Germany
* Corresponding author.

Conflict of interest:

No

Article ID: EPV0669

Features of the integrated support of children with neurotrauma at a remote stage of rehabilitation

Bratkova M. 1 2 *
Sidneva Y. 3
1 Moscow City University , Department of Clinical Psychology And The Basics Of Defectology, Moscow , Russian Federation
2 Clinical and Research Institute of Emergency Pediatric Surgery and Trauma (CRIEPST), Moscow, Russia, Rehabilitation Department, Moscow, Russian Federation
3 N.N.Burdenko National Medical Research Center of Neurosurgery; Clinical and Research Institute of Emergency Pediatric Surgery and Trauma, Neurosurgery; Rehabilitation , Moscow , Russian Federation
* Corresponding author.
Keywords: Integration, rehabilitation of children, educational route, neurotrauma

Conflict of interest:

No

Article ID: EPV0673
Subjects: Abstract, Mental Health Care

Examining burnout, depression, and attitudes regarding drug use among lebanese medical students during the 4 years of medical school

Talih F. 1
Daher M. 1
Ajaltouni J. 2 *
1 American University of Beirut Medical Center , Psychiatry, beirut , Lebanon
2 Lebanese American University Medical Center , Psychiatry, Beirut , Lebanon
* Corresponding author.
Keywords: Burnout, Healthcare, Dépression

Conflict of interest:

No

Article ID: EPV0675

Differences in compulsory admissions in southern denmark

BruunBech S. *
Hansen J.P.
Region of Southern Denmark , Mental Health Services Esbjerg , Esbjerg N , Denmark
* Corresponding author.
Keywords: coercion psychiatry outpatient, coercion, Psychiatry, outpatient

Conflict of interest:

No

Article ID: EPV0676

Perceived burden, depression and anxiety among partners of people living with scleroderma: an analysis of the moderation effects of social support and illness severity.

Juneau S.
Rizkallah E.
Leduc H.
El-Baalbaki G. *
Université du Québec À Montreal , Psychology, Montreal , Canada
* Corresponding author.
Keywords: Scleroderma, mental health, caregiver’s burden, Social support

Conflict of interest:

No

Article ID: EPV0680

Unmasking dissociative symptoms

Jambrošić Sakoman A. 1 *
Zegura I. 1
Todorić Laidlaw I. 2
1 University Psychiatric Hospital Vrapče , Department of Psychotic Disorders, Zagreb , Croatia
2 University Hospital Vrapče , Outpatient Clinic , Zagreb , Croatia
* Corresponding author.

Conflict of interest:

No

Article ID: EPV0681

Blades and scabies “desperate times call for desperate measures”

Maricalva E. *
Hospital Universitario Río Hortega , Psychiatry, Valladolid , Spain
* Corresponding author.
Keywords: mental health paradigm, integrative approach, quality of life

Conflict of interest:

No

Article ID: EPV0684

Indicators of the incidence of mental disorders and disability of the child population of the republic of belarus (2017)

Piatnitskaya I. 1 *
Litvinova O. 2
1 Belarusian State Medical University , Psychiatry, Minsk , Belarus
2 City Clinical Children’s Psychiatric Clinic of Minsk , Psychiatry, Minsk , Belarus
* Corresponding author.
Keywords: child population, mental disorders, disability

Conflict of interest:

No

Article ID: EPV0685

Development of first episode psychosis outpatient services at the department of psychiatry and psychotherapy, semmelweis university, budapest

Simon V. *
Hermán L.
Csukly G.
Zsigmond R.
Vass E.
Réthelyi J.
Semmelweis University , Psychiatry And Psychotherapy, Budapest , Hungary
* Corresponding author.
Keywords: early intervention, first psychosis, new service, Hungary

Conflict of interest:

No

Article ID: EPV0688

Mental health in residents. A descriptive analysis of the mental health care needs of training personnel.

Carracedo Sanchidrián D. 1 *
Hernández-Calle D. 2
Cano Arenas A. 3
1 Hospital Universitario la Paz, Mental Health , Madrid , Spain
2 Hospital Universitario La Paz , Psychiatry, Madrid , Spain
3 Universidad Pontificia Comillas , Psychosocial Intervention (unit Uninpsi) , Madrid , Spain
* Corresponding author.
Keywords: mental health, Residents, Public Health Care

Conflict of interest:

No

Article ID: EPV0690

Sociodemographical and clinical characteristics of the paime program

Esparrago Llorca G. 1 *
Corbacho Simón F. 2
Carrión Expósito L. 3
Jerez Barroso F.J. 4
Rejas Marcos C. 5
Robles Agüero E. 6
Blanco González Á.L. 7
Daniel Vega E. 2
1 Servicio Extremeño de Salud , Equipo De Salud Mental , Cáceres , Spain
2 Servicio Extremeño de Salud , Hospital Universitario De Cáceres , Cáceres , Spain
3 Servicio Andaluz de Salud , Ugc-salud Mental Hospital Infanta Margarita , Córdoba , Spain
4 Servicio Extremeño de Salud, Hospital San Pedro De Alcántara , Cáceres , Spain
5 Centro Vía de la Plata, Salud Mental , Cáceres , Spain
6 Servicio Extremeño de Salud , Centro De Salud Nuevo Cáceres , Cáceres , Spain
7 Servicio Extremeño de Salud , Hospital Virgen Del Puerto , Plasencia , Spain
* Corresponding author.
Keywords: health care, doctors, program

Conflict of interest:

No

Article ID: EPV0691

Patient and caregivers satisfaction associated with monthly or quarterly paliperodone palmitate treatment

Esparrago Llorca G. 1 *
Andrés Pereira G. 2
Juncosa Montes P. 2
Bordes Giménez M.D. 2
Díaz Fernández F. 2
Ríos Muñoz M.P. 1
Carreiras Gómez M.Á. 1
Rubio Merino M.I. 1
Gómez Del Castillo M.J. 1
1 Servicio Extremeño de Salud , Equipo De Salud Mental , Cáceres , Spain
2 Servicio Extremeño de Salud, Hospital San Pedro De Alcántara , Cáceres , Spain
* Corresponding author.
Keywords: caregivers, health care, treatment, satisfaction

Conflict of interest:

No

Article ID: EPV0692

Unhealthy behavior in junior medical students: is there a reason for concern?

Petunova S. 1 *
Zakharova A. 1
Dulina G. 1
Petunova Y. 2
Nikolaev E. 3
1 Ulianov Chuvash State University , Social And Clinical Psychology Department, Cheboksary , Russian Federation
2 I.M. Sechenov First Moscow State Medical University , Faculty of Medicine, Moscow , Russian Federation
3 Ulianov Chuvash State University , Faculty of Medicine, Cheboksary , Russian Federation
* Corresponding author.
Keywords: Unhealthy behavior, healthy lifestyles, medical students, Mental Health Care

Conflict of interest:

No

Article ID: EPV0693

Psychiatric disorders and stigma

Soares M.J. 1 *
Araújo A. 2
Pereira A.T. 1
Madeira N. 2
Rosendo I. 3
Miranda A.F. 3
Morais S. 2
Moura D. 2
Coroa M. 2
Macedo A. 1
1 Faculty of Medicine, University of Coimbra , Institute of Psychological Medicine, Coimbra , Portugal
2 Centro Hospitalar e Universitário de Coimbra , Department of Psychiatry, Coimbra , Portugal
3 University of Coimbra , Faculty of Medicine, Coimbra , Portugal
* Corresponding author.
Keywords: Stigma, psychiatric disorders, mental health

Conflict of interest:

No

Article ID: EPV0695

Socio-demographic factors predicting of emotional exhaustion among mental health professionals in a psychiatric hospital

Maalej R.
Zgueb Y.
Ouali U.
Ayedi S. *
Jelidi A.
Jomli R.
Nacef F.
Razi Hospital, A "avicenna" Department , Tunis , Tunisia
* Corresponding author.
Keywords: Demographic Factor, emotional exhaustion, mental health

Conflict of interest:

No

Article ID: EPV0696

The relation between inpatient violence and burnout among mental health professionals in a psychiatric hospital

Maalej R.
Zgueb Y.
Ouali U.
Ayedi S. *
Jelidi A.
Jomli R.
Nacef F.
Razi Hospital, A "avicenna" Department , Tunis , Tunisia
* Corresponding author.
Keywords: workplace violence, mental health, Hospital, Inpatients

Conflict of interest:

No

Article ID: EPV0697

Personal accomplishment among mental health workers in a psychiatric hospital in tunisia

Maalej R.
Zgueb Y.
Ouali U.
Ayedi S. *
Jelidi A.
Jomli R.
Nacef F.
Razi Hospital, A "avicenna" Department , Tunis , Tunisia
* Corresponding author.
Keywords: personal accomplishment, mental health, Hospital

Conflict of interest:

No

Article ID: EPV0698

Experts, patients and community mental health teams: a situation analysis

Bjedov S. 1 *
Medved S. 1
Bistrović P. 2
Ištvanović A. 3
Shields-Zeeman L. 4
Nijsen M. 5
Roth C. 6
Rojnic Kuzman M. 1 7
1 University Clinical Hospital Zagreb , Clinic Of Psychiatry And Psychological Medicine, Zagreb , Croatia
2 Health Center Zagreb-Centar , Family Medicine Office, Zagreb , Croatia
3 Croatian Institute of Public Health , Department For School Medicine, Mental Health And Addiction Prevention , Zagreb , Croatia
4 Dutch Institute for Mental Health and Addiction/Trimbos Institute , Department of Public Mental Health And Prevention, Utrecht , Netherlands
5 Dutch Institute for Mental Health and Addiction/Trimbos Institute , Department of Re-integration, Utrecht , Netherlands
6 University Hospital Heidelberg , Department of General Practice And Health Services Research , Heidelberg , Germany
7 University of Zagreb , School of Medicine, Zagreb , Croatia
* Corresponding author.
Keywords: Severe mental illness, community mental health care team, outpatient care, mobile team

Conflict of interest:

No

Article ID: EPV0701

Prenatal malnutrition predisposes to addictive behaviour and antisocial personality traits

Franzek E. *
Yes We Can Clinics, Department of Research And Development, Eindhoven , Netherlands
* Corresponding author.
Keywords: Prenatal malnutrion, Antisocial personality, addictive behaviour, sex ratio men to woman

Conflict of interest:

No

Article ID: EPV0706

The community group of mental health, a strategy of care in the health field

Hormanez M. *
Cardoso C.
Universidade de São Paulo - USP , Psicologia , Ribeirão Preto. São Paulo ., Brazil
* Corresponding author.
Keywords: mental health, health promotion, Psychotherapy, Group

Conflict of interest:

No

Article ID: EPV0707

Initial experiences of recover-e CMH mobile teams in N. Macedonia / patients homes as a therapeutic setting

Bajraktarov S.
Naumovska A.
Kalpak G. *
Milutinović M.
Novotni L.
Novotni A.
University Clinic of Psychiatry , Psychiatry, Skopje , North Macedonia
* Corresponding author.
Keywords: community health, mobile team, public health, mental health

Conflict of interest:

No

Article ID: EPV0708

The future of rural mental health in greece: dilemmas of a young psychiatrist.

Kourtesis I. *
"DAFNI" Psychiatric Hospital , 10th, HAIDARI , Greece
* Corresponding author.
Keywords: mental health care, professional isolation, ethics, rural psychiatry

Conflict of interest:

No

Article ID: EPV0709

Diagnostic communication in psychiatry - perspectives of patients and caregivers

Lopes L. *
Pereira S.
Centro Hospitalar de Vila Nova de Gaia/Espinho , Serviço De Psiquiatria, Vila Nova de Gaia , Portugal
* Corresponding author.
Keywords: Psychiatry, Caregivers, Patients, Communication

Conflict of interest:

No

Article ID: EPV0713

The influence of negative and positive manifestations of reflection on cognitive states in solving creative problems

Shaiakhmetova L. *
Yusupov M.
Prokhorov A.
Chernov A.
Kazan Federal University , General Psychology, Kazan , Russian Federation
* Corresponding author.

Conflict of interest:

No

Article ID: EPV0714

National health information system as an efficient tool for mapping of the mental health care reform in the czech republic

Soukupova J. *
Melicharová H.
Šanca O.
Jarkovsky J.
Institute of Health Information and Statistics of the Czech Republic , -, Prague , Czech Republic
* Corresponding author.
Keywords: epidemiolodgy, healthcare mapping and planning, mental healthcare reform

Conflict of interest:

No

Article ID: EPV0715

Ensure healthy parent-child bonds in the first year of life as a measure to contribute to greater resilience in people and society

Hernandez-Santillan G. 1 *
Bravo-Ortiz M.F. 2
Alcami Pertejo M. 2
Fernandez-Sanchez A. 2
Lahera G. 2
1 Universidad Autónoma de Madrid , Medicine, Madrid , Spain
2 La Paz University Hospital Research Health Institute (IdiPAZ) , Psychiatry, Clinical Psychology And Mental Health , Madrid, Spain, 3 University of Alcala, Faculty of Medicine, Madrid, Spain
* Corresponding author.
Keywords: Prevention, attachment, parent-child bond, Resilience

Conflict of interest:

No

Article ID: EPV0717

Religious beliefs and practices in college students: their association with stress, affect, cognitive processes and emotion regulation strategies

Soares M.J. 1 *
Amaral A.P. 2
Pereira A.T. 1
Bos S. 1
Macedo A. 1
1 University of Coimbra , Faculty of Medicine, Department of Psychological Medicine, Coimbra , Portugal
2 Polytechnic Institute of Coimbra , Coimbra Health School , Coimbra , Portugal
* Corresponding author.
Keywords: affect, Religious beliefs and practices, cognitive processes and emotion regulation strategies, stress

Conflict of interest:

No

Article ID: EPV0719
Subjects: Abstract, Mental Health Policies

Divergencies in the organization of the comunity-based mental health services of leganés and río de janeiro: a tale of two cities.

Costa Ferrera Da Silva M.L. 1 *
Cerame Del Campo Á. 2
Coucheiro Limeres P. 2
De Hita Santillana R. 2
1 Hospital Universitario Severo Ochoa , Psyhciatrist, leganes , Spain
2 Instituto psiquiatrico Jose Germain , Psyhciatric Trainee , leganes , Spain
* Corresponding author.
Keywords: mental health care, community-based psychiatry, desinstitutionalization

Conflict of interest:

No

Article ID: EPV0721

Ethical, legal and clinical aspects of mechanical restraint: a narrative review

Nocete Navarro L. 1 *
López De Loma Osorio V. 2
Borrero Granell L. 3
Bravo-Ortiz M.F. 4
Fernández Liria A. 2
1 Universidad Autónoma de Madrid , Psychiatry, Madrid , Spain
2 Hospital Universitario Príncipe de Asturias , Psychiatry, Alcalá de Henares , Spain
3 Hospital Universitario Nuestra Señora de Candelaria , Psychiatry, Santa Cruz de Tenerife , Spain
4 La Paz University Hospital Research Health Institute (IdiPAZ) , Psychiatry, Clinical Psychology And Mental Health , Madrid , Spain
* Corresponding author.
Keywords: ethical challenges, mechanical restraint, Mental Health Policies

Conflict of interest:

No

Article ID: EPV0723

On track program for first episode psychosis services in chile: stakeholders perspective in the adaptation phase

Burrone M.S. 1 *
Reginatto G. 1
Arriata T. 1
Velasco P. 1
Susser E. 2 3
Dixon L. 3 4
Alvarado R. 1 5
Yang L. 6
Bello I. 3 4
Cabassa L. 7
1 Universidad de O´Higgins , Instituto De Ciencias De La Salud, Rancagua , O’Higgins , Chile
2 Columbia University , Mailman School of Public Health, NYC , United States of America
3 Columbia University , New York State Psychiatric Institute , NYC , United States of America
4 Columbia University , Vagelos College Of Physicians And Surgeons, NYC , United States of America
5 Universidad de Chile , Escuela De Salud Pública - Facultad De Medicina, Santiago , Chile
6 New York University , New York University , NYC , United States of America
7 Washington University in St. Louis , The Brown School, St Louis , United States of America
* Corresponding author.
Keywords: Early Intervention Services, Chile, Public policies, First Episode Psychosis

Article ID: EPV0724

Cost-effectiveness analysis of interventions to achieve universal health coverage for schizophrenia in méxico

Cabello-Rangel H. 1 *
Díaz-Castro L. 2
Pineda-Antúnez C. 3
1 Psychiatric Hospital “Fray Bernardino Álvarez" ., Division Of Diagnostic Assistants, Mexico City , Mexico
2 National Institute of Psychiatry Ramon de la Fuente Muñiz , Direction Of Epidemiological And Psychosocial Research, Mexico City , Mexico
3 National Public Health Institute , Department of Health Services Research, Cuernavaca City , Mexico
* Corresponding author.
Keywords: Universal health coverage, schizophrenia, cost-effectiveness

Conflict of interest:

No

Article ID: EPV0726

From 19th century asylum buildings to a modern 21th century psychiatric hospital - visions and experiences

Harpøth A. 1 *
Sørensen L. 1 2
1 Aarhus University Hospital , Department of Forensic Psychiatry, Aarhus N, Denmark
2 Aarhus University , Clinical Medicine, Aarhus N, Denmark
* Corresponding author.
Keywords: Structural safety, Organization, physical environment, coercion

Conflict of interest:

No

Article ID: EPV0728

The medicalization model for cannabis based treatments, the israeli experience

Roitman P.
Clalit Health Services , Psychiatry, Bet Shemesh , Israel
Keywords: Medicinal Cannabis, public health, PTSD

Conflict of interest:

No

Article ID: EPV0729

Quality in mental health services: assessment and improvement guide

Samartzis L. 1 2 *
Talias M. 1
1 Open University of Cyprus , Faculty of Economics And Management, Nicosia , Cyprus
2 Cyprus Mental Health Services , Department of Psychiatry, Nicosia , Cyprus
* Corresponding author.
Keywords: mental health, policy, quality, indicators

Conflict of interest:

No

Article ID: EPV0730

Pre-discharge factors associated with early readmission to psychiatric inpatient services within 90 days

Wright J. *
Thomas R.
Barnet Enfield and Haringey NHS Mental Health Trust , St Ann’s Hospital , London , United Kingdom
* Corresponding author.
Keywords: readmission, risk factors, inpatient services, predischarge factors

Conflict of interest:

No

Article ID: EPV0732
Subjects: Abstract, Migration and Mental Health of Immigrants

Cultural and migration influence on psychiatric syndromes.

Álvarez González V. 1 *
Suarez Pérez C. 2
Ballesteros López J. 2
1 Hospital Universitario Infanta Cristina , Psychiatry, Madrid , Spain
2 Hospital Universitario Infanta Cristina , Psiquiatría, Parla, Madrid , Spain
* Corresponding author.
Keywords: inmigration, Transcultural Psychiatry, psychopathology, cross-cultural psychiatry

Conflict of interest:

No

Article ID: EPV0733

Mental health risks in immigrants confined in cie.

Borrero Granell L. 1 *
Nocete Navarro L. 2
Chami Mateo T. 1
Quintana Monzón A. 1
Palanca Estévez V. 1
1 Hospital Universitario Nuestra Señora de Candelaria , Psychiatry, Santa Cruz de Tenerife , Spain
2 Universidad Autónoma de Madrid , Psychiatry, Madrid , Spain
* Corresponding author.
Keywords: immigration CIE Human rights

Conflict of interest:

No

Article ID: EPV0734

Postpartum psychosis in migrant women: analysis of related risk factors

Coucheiro Limeres P. *
Cerame Del Campo Á.
Franco Soler A.V.
De La Mata Ruiz I.
Instituto Psiquiatrico José Germain , Psiquiatría, Leganés , Spain
* Corresponding author.
Keywords: migration, postpartum, psychosis

Conflict of interest:

No

Article ID: EPV0735

Prevalence of major depressive disorder among immigrants of the metropolitan region of santiago, chile

Errazuriz A. *
Pontificia Universidad Católica de Chile , Psychiatry, Santiago , Chile
* Corresponding author.
Keywords: Migrants, prevalence, global mental health, Dépression

Conflict of interest:

No

Article ID: EPV0737

The italian project start-er: work in group with migrants to face post-traumatic vulnerability

Pacetti M. *
Centro Salute Mentale Forlì , Italy , Dsm-dp, Forlì, Italy
* Corresponding author.
Keywords: Migrants, vulnerability, asylum seekers, group

Conflict of interest:

No

Article ID: EPV0739

Differences between adopted children and non-adopted children related to attention-deficit disorder with hyperactivity. The importance of the effect of deprivation.

Martín Villarroel C. *
Carpio Garcia L.
Dominguez Cutanda J.
Belmonte Garcia G.
Matsuura J.
Sánchez Revuelta M.
Garcia E.
HOSPITAL PROVINCIAL DE LA MISERICORDIA , PsiquiatrÍa (consultas Externas), TOLEDO , Spain
* Corresponding author.
Keywords: attention-deficit hyperactivity disorder, adopted children, institutionalized, deprivation

Conflict of interest:

No

Article ID: EPV0740

Healthy lifestyle choices and self-esteem in russian and central asian university students

Nikolaev E. *
Ulianov Chuvash State University , Department of Social And Clinical Pscyhology, Cheboksary , Russian Federation
* Corresponding author.
Keywords: self-esteem, healthy lifestyle choices, University students, migration

Conflict of interest:

No

Article ID: EPV0742

Migration trauma, substance use and psychiatric features associated to suicide attempts and self-harm behaviours of detained migrants

Artoni C. 1
Marchi M. 1 *
Magarini F. 1
Longo F. 1
Reggianini C. 2
Florio D. 3
Galeazzi G. 1
Ferrari S. 1
1 University of Modena and Reggio Emilia , Department of Biomedical, Metabolic And Neural Sciences, Modena , Italy
2 Ausl Reggio Emilia , Mental Health And Drug Abuse , Scandiano , Italy
3 Ausl Modena , Mental Health And Drug Abuse , Modena , Italy
* Corresponding author.
Keywords: trauma, migration, Self-harm, Suicide

Conflict of interest:

No

Article ID: EPV0744

Clinical models in mental health care for multicultural societies and the specificities of french transcultural consultations. a narrative review.

Carballeira Carrera L. 1
Moro M.R. 2
López Álvarez I. 3
Curto Ramos J. 4 *
Lachal J. 5
1 Niño Jesús University Children’s Hospital , Psychiatry And Clinical Psychology Department, Madrid , Spain
2 INSERM, Methods And Cultures , Aiep, Paris , France
3 HOSPITAL UNIVERSITARIO LA PAZ , Psychiatry, Clinical Psychology And Mental Health , Madrid , Spain
4 La Paz University Hospital , Psychiatry, Clinical Psychology And Mental Health , Madrid , Spain
5 APHP , Hôpital Cochin , Maison De Solenn, Paris , France
* Corresponding author.
Keywords: migrant families, mental healthcare, cultural diversity, Trans-cultural Psychiatry

Conflict of interest:

No

Article ID: EPV0746

Psychopathology in central american transmigrants as they pass through the southern border of mexico.

Lopez Salinas A. 1
Ramos Ruiz F. 2
Oscoz Irurozqui M. 3 *
1 TECNOLOGICO DE MONTERREY , Psychiatry, SALTILLO , Mexico
2 TECNOLOGICO DE MONTERREY , Psychiatry, MONTERREY , Mexico
3 Benito Menni CASM , Psychiatry, Sant Boi de Llobregat , Spain
* Corresponding author.
Keywords: Migrants, mental health, Mexico, psychopathology

Conflict of interest:

No

Article ID: EPV0747
Subjects: Abstract, Neuroimaging

Brain activation patterns in FRMI with conventional and unconventional film narratives

Caballero L. (Sr) 1 *
Fernández R. 1
García M. 1
Fernández C. 2 3
Caballero L. (Jr) 3
1 HM CINAC , Servicio De Psiquiatría Y Psicología Clínica Hm Hospitales , Móstoles , Spain
2 HM Hospitales , Servicio De Radiodiagnóstico, Móstoles , Spain
3 Independent, -, Móstoles , Spain
* Corresponding author.
Keywords: neuroimaging, neurocinematics, fMRI, inter-subject correlation

Conflict of interest:

No

Article ID: EPV0751

Psychotic disorder concurrent with dandy-walker malformation: case report

Martinez M. *
Barba Z.
Hospital Marina Baixa , Department of Mental Health-psychology, Villajoyosa , Spain
* Corresponding author.
Keywords: Dandy Walker malformation, Psychotic Disorder, Neurodevelopment, Cerebellum

Conflict of interest:

No

Article ID: EPV0754
Subjects: Abstract, Neuroscience in Psychiatry

Examining vagal tone as a mechanism in gut-brain communication in major depressive disorder

Forth E. 1 *
Milev R. 2
Chinna Meyyappan A. 1
Hawken E. 3
Wallace C. 3
1 Providence Care Hospital , Psychiatry, Kingston , Canada
2 Queen’s University , Psychiatry, Kingston , Canada
3 Providence Care Hospital , Queen’s University , Psychiatry, Kingston , Canada
* Corresponding author.
Keywords: major depressive disorder, Electrophysiology, gut microbiome, Vagus Nerve

Conflict of interest:

No

Article ID: EPV0755

Delusion and depression in huntington disease as a neuropsychiatry paradigm.

Galvañ J. 1 *
Noguero Alegre A. 2
1 Hospital Universitari Son Espases , Psychiatry, Palma , Illes Balears , Spain
2 Hospital Son Espases, Psychiatry, palma , Spain
* Corresponding author.
Keywords: Neuropsychiatry, delusion, psychosis, Dépression

Conflict of interest:

No

Article ID: EPV0756

Poststroke psychosis: a case report

Miranda C. 1 *
Fernandes Santos C. 1
Medeiros A.B. 1
Conde E. 1
Senos Moutinho F. 2
1 Hospital Garcia de Orta , Department of Psychiatry And Mental Health, Almada , Portugal
2 Garcia de Orta Hospital, Psychiatry And Mental Health , Almada , Portugal
* Corresponding author.
Keywords: Neuropsychiatry, poststroke psychosis, stroke, delusions

Conflict of interest:

No

Article ID: EPV0759

Anti-NMDA (n-methyl-d-aspartate) receptor encephalitis presenting with unusual psychiatric symptoms

Tan H.T. *
Ravichandran N.
Institute of Mental Health, General Psychiatry , Singapore , Singapore
* Corresponding author.
Keywords: outcome, symptoms, NDMA receptor encephalitis, Neuropsychiatry

Conflict of interest:

No

Article ID: EPV0761

Neurocognitive deficits in mental pathology in children and adolescents. Luria’s approach.

Zvereva N. 1 *
Sergienko A. 1
Balakireva E. 2
1 Mental Health Research Center ; Moscow State University of Psychology and Education (MSUPE) , Clinical Psychology; Neuro- And Pathopsychology Of Development , Moscow , Russian Federation
2 Mental Health Research Center , Department of Child Psychiatry, Moscow , Russian Federation
* Corresponding author.
Keywords: endogenous mental disordeds, A.R.Luria's approach, neuropsychological assessment, Children

Conflict of interest:

No

Article ID: EPV0762

The complex use of anticonvulsant therapy and a machine-learning-based software package for patients with various forms of seizures.

Moroz S. 1 *
Makarova I. 1
Semenikhina V. 2
Khaitov R. 1
Turishcheva N. 1
1 Dnipropetrovsk Regional Clinic Hospital named after I.I.Mechnikov , Psychosomatic Center, Dnepr , Ukraine
2 Dnipropetrovsk Regional Clinic Hospital named after I.I.Mechnikov , Psychosomatic Center, Днепр , Ukraine
* Corresponding author.

Conflict of interest:

No

Article ID: EPV0763

Screen use and neuro-psychological functions in 10-12 year-old children in monteria city colombia

Muñoz Argel M.N. 1 *
Ruiz Gonzalez E. 1
Romero Otalvaro A. 1
Uribe Urzola A. 1
Vélez Carvajal J. 2
1 Universidad Pontificia Bolivariana , Psicología , Monteria , Colombia
2 Universidad Pontificia Bolivariana, Social Communication , Monteria , Colombia
* Corresponding author.
Keywords: Screens, neuropsychological functions, boys and girls.

Conflict of interest:

No

Article ID: EPV0765

Neuropsychiatric symptoms of huntington’s disease

Santos N. 1 *
Alho A. 1
Santos C. 2
Gasparinho R. 1
Ferreira L. 1
Martins M. 1
Fernandes N. 1
Sêco E. 1
1 Hospital Distrital de Santarém, Psychiatry And Mental Health , Santarém , Portugal
2 Centro Hospitalar Lisboa Ocidental, Psychiatry And Mental Health , Lisboa , Portugal
* Corresponding author.
Keywords: Neuropsychiatric symptoms, Huntington’s Disease

Conflict of interest:

No

Article ID: EPV0766

Hashimoto’s encephalitis with psychotic symptoms – a case report

Santos N. *
Alho A.
Ferreira L.
Fernandes N.
Gasparinho R.
Martins M.
Sêco E.
Hospital Distrital de Santarém, Psychiatry And Mental Health , Santarém , Portugal
* Corresponding author.
Keywords: Hashimoto’s encephalitis, encephalitis, neuropsychiatric disorders

Conflict of interest:

No

Article ID: EPV0767

The effects of camelina sativa seed extract in a complex irritable bowel syndrome mice model, focussing on preliminary correlations between lipid peroxidation and behavioral parameters

Balmus I.M. 1 *
Cojocariu R. 2
Lefter R. 3
Hritcu L. 4
Ababei D. 5
Ciobica A. 3 6 7
Copolovici D. 8
Copolovici L. 8
Copaci S. 9
Jurcoane S. 7 9
1 Alexandru Ioan Cuza University of Iasi , Department of Interdisciplinary Research In Science, Iasi , Romania
2 Alexandru Ioan Cuza University of Iasi , Biology, Iasi , Romania
3 Romanian Academy , Center Of Biomedical Research, Iasi , Romania
4 ”Ion Ionescu de la Bra” University of Agricultural Science and Veterinary Medicine, Veterinary Medicine , Iasi , Romania
5 Facultatea de Medicina si Farmacie Grigore T. Popa, Pharmacology, Iasi , Romania
6 “ Alexandru Ioan Cuza” University of Iasi , Research, Iasi , Romania
7 Academy of Romanian Scientists , Science, Bucharest , Romania
8 Aurel Vlaicu" University , Natural Sciences, Arad , Romania
9 Faculty of Biotechnology, University of Agronomic Sciences and Veterinary Medicine , Biotechnology, Bucharest , Romania
* Corresponding author.
Keywords: oxidative stress, functional gastrointestinal disorder, animal behavior

Article ID: EPV0768

Microbiome-dependent antioxidant, gastrointestinal and neurological modulation of irritable bowel syndrome symptomatology

Balmus I.M. 1 *
Cojocariu R. 2
Ilie O. 2
Lefter R. 3
Ciobica A. 3 4 5
Trifan A. 6
Stanciu C. 3
1 Alexandru Ioan Cuza University of Iasi , Department of Interdisciplinary Research In Science, Iasi , Romania
2 Alexandru Ioan Cuza University of Iasi , Biology, Iasi , Romania
3 Romanian Academy , Center Of Biomedical Research, Iasi , Romania
4 “ Alexandru Ioan Cuza” University of Iasi , Department of Research, Iasi , Romania
5 Academy of Romanian Scientists , Science, Bucharest , Romania
6 Gr. T. Popa University of Medicine and Pharmacy , Medicine, Iasi , Romania
* Corresponding author.
Keywords: Microbiome, oxidative stress, neuropsychiatric comorbidies, Irritable bowel syndrome

Article ID: EPV0770

Preventing the progression of cognitive impairments in epilepsy

Blazhina I. 1 *
Korostiy V. 2
1 Bucovinian State Medical University , Department of Nervous Diseases Psychiatry And Medical Psychology, Chernivtsi , Ukraine
2 Kharkiv National Medical University , Department of Psychiatry, Narcology And Medical Psychology, Kharkiv , Ukraine
* Corresponding author.
Keywords: Cognitive disorders, Epilepsy

Conflict of interest:

No

Article ID: EPV0771

Comparison of visual p300 amplitude and latency in people with schizophrenia and bipolar disorder

Cavieres A. 1 *
Koscina V. 2
Zepeda L. 2
Maldonado R. 2
Prado P. 2
El-Deredy W. 2
1 Universidad de Valparaiso , Psiquiatria , Viña del Mar, Chile
2 Universidad de Valparaiso , Psiquiatria, Valparaíso , Chile
* Corresponding author.
Keywords: visual P3, schizophrenia, Bipolar disorder

Conflict of interest:

No

Article ID: EPV0777

Brain connectivity changes after long-term alcohol abstinence

Martínez Maldonado A. 1 *
Jurado Barba R. 1
Sion A. 2
Domínguez Centeno I. 1
Rubio Valladolid G. 2
1 Camilo José Cela University , Psychology Department, Villanueva de la Cañada , Spain
2 12 de Octubre Hospital , Psychiatry, Madrid , Spain
* Corresponding author.
Keywords: Alcohol, abstinence, EEG, Recovery

Conflict of interest:

No

Article ID: EPV0778

Teaching psychopharmacology to residents using research domain criteria: an ongoing exploration

Mehl-Madrona L. 1 *
Mainguy B. 1
Mcfarlane P. 2
1 University of Maine, Graduate School , Orono , United States of America
2 University of New England/Eastern Maine Medical Center , Family Medicine Residency, Bangor , United States of America
* Corresponding author.
Keywords: Psychiatric Prescribing, Neural Circuitry, Training of Residents, Research Domain Criteria

Conflict of interest:

No

Article ID: EPV0779

Childhood maltreatment, social cognition and functionality in healthy adults

Fares-Otero N. 1
Fernandez Cancer P. 1
Garcia Lopez A. 1
Rodriguez Toscano E. 2
Rodriguez-Jimenez R. 1 2 *
1 Department of Psychiatry, Instituto De Investigación Sanitaria Hospital 12 De Octubre (imas12), Madrid , Spain
2 CIBERSAM, Biomedical Research Networking CentreIn Mental Health , Madrid , Spain
* Corresponding author.
Keywords: Child abuse, social cognition, functionality, adulthood

Conflict of interest:

No

Article ID: EPV0781

Comorbid neuropsychiatric and endocrine disorders upon prenatal exposure to ionizing radiation

Kaminskyi O. 1
Loganovsky K. 2 *
Talko V. 3
Afanasyev D. 1
Loganovska T. 2
Lavrenchuk G. 3
Kopylova O. 1
Chikalova I. 1
Muraviova I. 1
Tepla O. 1
Kiselyova I. 4
1 State Institution "National Research Centre for Radiation Medicine of National Academy of Medical Sciences of Ukraine , Department of Radiation Endocrinology, Institute of Clinical Radiology, Kyiv , Ukraine
2 State Institution "National Research Centre for Radiation Medicine of National Academy of Medical Sciences of Ukraine , Department of Radiation Psychoneurology, Institute of Clinical Radiology, Kyiv , Ukraine
3 State Institution «National Research Center for radiation Medicine of the National Academy of Medical Sciences of Ukraine» , Department Od Radiobiology, Institute of Experimental Radiology,, Kyiv , Ukraine
4 Kyiv City Clinical Endocrinological Center, Advisory Outpatient Clinic , Kyiv , Ukraine
* Corresponding author.
Keywords: brain, parathyroids, endocrine system, ionizing radiation

Conflict of interest:

No

Article ID: EPV0784

Cerebro-ophtalmic effects of ionizing radiation: a follow-up study

Loganovsky K. 1 *
Fedirko P. 2
Marazziti D. 3
Antypchuk K. 1
Babenko T. 4
Masiuk S. 4
Garkava N. 5
Loganovska T. 1
Dorichevska R. 4
Kuts K. 1
Petrchuk I. 1
Kreinis G. 1
1 State Institution "National Research Centre for Radiation Medicine of National academy of Medical Sciences of Ukraine, Department of Radiation Psychoneurology, Institute of Clinical Radiology, Kyiv , Ukraine
2 State Institution "National Research Centre for Radiation Medicine of National academy of Medical Sciences of Ukraine, Institute For Health Physics And Dosimetry , Kyiv , Ukraine
3 University of Pisa , Clinical and Experimental Medicine, Pisa , Italy
4 State Institution "National Research Centre for Radiation Medicine of National academy of Medical Sciences of Ukraine, Institute For Health Physics And Epidemiology , Kyiv , Ukraine
5 State Instition "Dnipropetrovsk Medical Academy of the Ministry of Health of Ukraine" , Neurology And Ophtalmology, Dnipro , Ukraine
* Corresponding author.
Keywords: Chornobyl catastrophe, ionizing radiation, interventional radiologists, vascular ophtalmological dis

Conflict of interest:

No

Article ID: EPV0789
Subjects: Abstract, Obsessive-Compulsive Disorder

Clinical management in posnatal obsessive compulsive disorder. A case report.

Ximenez De Embun I.
Mantilla Reyes M.F. *
Roca Lecumberri A.
Fernandez Gomis N.
Roda Guillen E.
Pinzon Espinosa J.
Sole Roige E.
García – Esteve L.
Hospital Clínic de Barcelona, Institut Clínic De Neurociencies (icn). Unidad De Salud Mental Perinatal , Barcelona , Spain
* Corresponding author.
Keywords: ocd, postpartum, Intervention, bond

Conflict of interest:

No

Article ID: EPV0792

Development of a family psychoeducational intervention for patients with obsessive-compulsive disorder according to falloon’s model

Palummo C. *
Sampogna G.
Giallonardo V.
Solimene N.
Gravagnone M.
Zinno F.
Luciano M.
Del Vecchio V.
Fiorillo A.
University of Campania Luigi Vanvitelli , Psychiatry, naples , Italy
* Corresponding author.
Keywords: family psychoeducational intervention, obsessive-compulsive disorder, Falloon’s model, family burden

Conflict of interest:

No

Article ID: EPV0793
Subjects: Abstract, Old Age Psychiatry

Efficacy of physical exercise in the treatment of depressed older adults

Arts M.H.L. 1 *
Petrykiv S. 2
De Jonge L. 3
1 GGZ Westelijk Noord-Brabant, Geriatric Psychiatry And Neuropsychiatry , Halsteren , Netherlands
2 GGZ WNB , Psychiatry, Halsteren , Netherlands
3 Leonardo Scientific Research Institute , Neuropsychiatry, Groningen , Netherlands
* Corresponding author.
Keywords: physical exercise, Late-life depression

Conflict of interest:

No

Article ID: EPV0794

Polypharmacy as a risk factor for insomnia in older age. A cross-sectional study in greece.

Argyropoulos K. 1
Damka P. 2
Argyropoulou A. 3
Avramidis D. 4 *
Jelastopulu E. 1
1 School of Medicine, University of Patras , Public Health, Patra , Greece
2 Health Center of Distomo, General Practice , Distomo , Greece
3 Health Center of Andravida, General Practice, Patra , Greece
4 University of Patras , School of Medicine, Patra , Greece
* Corresponding author.
Keywords: İnsomnia, Older Age, Polypharmacy, Athens Insomnia Scale

Conflict of interest:

No

Article ID: EPV0795

Differential impact of loneliness patterns on health status in old age. a longitudinal study

Martín-María N. 1
Caballero F. 2
Lara E. 3
Olaya B. 4
Haro J.M. 4
Ayuso-Mateos J.L. 5 *
Miret M. 1
1 Universidad Autónoma de Madrid , Psychiatry, Madrid , Spain
2 Universidad Autonoma de Madrid , Department of Preventive Medicine, Public Health, And Microbiology , Madrid , Spain
3 Hospital Universitario de La Princesa , Psychiatry, Madrid , Spain
4 Parc Sanitari Sant Joan de Déu , Research, Innovation And Teaching Unit, Barcelona, Spain
5 Universidad Autonoma de Madrid , Psychiatry, Madrid , Spain
* Corresponding author.
Keywords: transient loneliness, chronic loneliness, health status, longitudinal study

Conflict of interest:

No

Article ID: EPV0796

Psychiatric symptoms in the elderly as features of degenerative neurologic disorders.

Ballesteros López J. 1 *
Álvarez González V. 2
Suarez Pérez C. 1
1 Hospital Universitario Infanta Cristina , Psiquiatría, Parla, Madrid , Spain
2 Hospital Universitario Infanta Cristina , Psychiatry, Madrid , Spain
* Corresponding author.
Keywords: first psychiatric symptoms, Elderly, neurologic disorders, prodrome

Conflict of interest:

No

Article ID: EPV0797

Is dementia associated with psychotic symptoms in the geriatric population? A review

Banik D. 1 *
Chowdhury M. 2
Rahman M. 2
1 SUNY Downstate Medical Center, Geriatric Psychiatry , Brooklyn , United States of America
2 Jamaica Hospital Medical Center , Psychiatry, Richmond Hill , United States of America
* Corresponding author.
Keywords: Psychosis, Dementia, Geriatric Population

Conflict of interest:

No

Article ID: EPV0800

Sleep quality of elderly living in a retirement home: a tunisian study

Charfi N.
Chaari I. *
Lajmi I.
Omri S.
Feki R.
Maalej Bouali M.
Zouari L.
Ben Thabet J.
Maalej M.
Hedi Chaker University Hospital , Psychiatry C Department, Sfax , Tunisia
* Corresponding author.
Keywords: PSQI, Retirement Home, Psychogeriatrics, Sleep quality

Conflict of interest:

No

Article ID: EPV0801

Acute psychosis in elderly patients: evaluation and intervention

Duarte D. 1 *
Ramos L. 1
Mendonça M.N. 2
1 Centro Hospitalar Universitário do Algarve , Department of Psychiatry And Mental Health, Faro , Portugal
2 Centro de Saúde de Faro, Usf Ria Formosa , Faro , Portugal
* Corresponding author.
Keywords: late onset schizophrenia, Elderly, psychosis

Conflict of interest:

No

Article ID: EPV0802

What your eyes don’t see: a case report of charles bonnet syndrome

Duarte D. 1 *
Mendonça M.N. 2
1 Centro Hospitalar Universitário do Algarve , Department of Psychiatry And Mental Health, Faro , Portugal
2 Centro de Saúde de Faro, Usf Ria Formosa , Faro , Portugal
* Corresponding author.
Keywords: Charles Bonnet syndrome, visually impairment, diagnostic criteria, visual hallucinations

Conflict of interest:

No

Article ID: EPV0804

Behavioural changes in an elderly woman- challenges in diagnosis

Eng S.L.
Changi General Hospital , Psychological Medicine, Singapore , Singapore
Keywords: delirium, dementia, Dépression

Conflict of interest:

No

Article ID: EPV0805

Spiritual and religious beliefs and sense of coherence in greek older adults

Giannouli V. 1 *
Giannoulis K. 2
1 Bulgarian Academy of Sciences , Institute of Neurobiology, Sofia , Bulgaria
2 Aristotle University of Thessaloniki , School of Theology, Thessaloniki , Greece
* Corresponding author.
Keywords: sense of coherence, old age, spiritual and religious beliefs, Greek Orthodox

Conflict of interest:

No

Article ID: EPV0809

- Bipolar disorder due to cerebral infarction -

Montero Quer L. *
De Benito Chafer A.
Fuenlabrada University Hospital , Psychiatry, Madrid , Spain
* Corresponding author.
Keywords: Bipolar disorder, Silent cerebral infarction, old age psychiatry, Manic symptoms

Conflict of interest:

No

Article ID: EPV0813

Depressive symptoms as clinical presentation of gliobastoma multiforme located on frontal lobe in elderly: report of two cases

Regueira P. *
Cerejeira J.
Centro Hospitalar Universitário de Coimbra , Psychiatry, Coimbra , Portugal
* Corresponding author.
Keywords: glioblastoma multiforme, depressive symptoms, elderly

Conflict of interest:

No

Article ID: EPV0816

Dementia vs. depression, the importance of early diagnosis of neurocognitive disorders.

Valverde Barea M. 1 *
Cartas Moreno F. 2
Solis M. 1
1 COMPLEJO HOSPITALARIO DE JAEN , Psiquiatria , JAEN , Spain
2 HOSPITAL SAN JUAN DE LA CRUZ, UBEDA , Psiquiatria, JAEN , Spain
* Corresponding author.
Keywords: dementia, Dépression, neurocognitive disorders, diagnosis

Conflict of interest:

No

Article ID: EPV0817

Sexual unwellness: a qualitative study with older adults from portugal and romania

Von Humboldt S. 1 *
Niculescu G. 2
Low G. 3
Leal I. 1
1 Instituto Superior de Psicologia Aplicada (ISPA) , Psychology, Almada , Portugal
2 Romanian Association for Person Centred Psychotherapy (ARPCP) , Psychology, Bucareste , Romania
3 Faculty of Nursing, University of Alberta , Psychology, Edmonton , Canada
* Corresponding author.
Keywords: cross-national, older adults, qualitative study, sexual unwellness

Conflict of interest:

No

Article ID: EPV0819

Evaluation of the vagus nerve stimulation in neurocognitive disorders

Vasile D. *
Vasiliu O.
University Emergency Central Military Hospital Dr. Carol Davila , Psychiatry, Bucharest , Romania
* Corresponding author.
Keywords: vagus nerve stimulation, neurocognitive disorders, cognitive enhancers

Article ID: EPV0820

Case management of vascular depression

Vasiliu O.
University Emergency Central Military Hospital Dr. Carol Davila , Psychiatry, Bucharest , Romania
Keywords: good practices, retrospective analysis, vascular depression, neurodegenerative processes

Article ID: EPV0824

Adaptation of an evidence-based intervention for disability prevention, implemented by community health workers serving ethnic minority elders

Falgas-Bague I. 1 *
Del Cueto P. 1
Kim E. 2
Fuentes L. 1
Ramos Z. 1
Alegria M. 3
1 50 Staniford Street , Disparities Research Unit. Department of Medicine, Boston , United States of America
2 Tuffts University , Community Health, Medford , United States of America
3 Harvard University , Harvard Medical School, Boston , United States of America
* Corresponding author.
Keywords: ethnic minorities, community health workers, elder population, psycho-social interventions

Conflict of interest:

No

Article ID: EPV0825

Correlation of behavioral and psychological symptoms and the therapeutical effects with the degree of cognitive impairment in dementia

Hrnjica A. *
Bise S.
Lokmic-Pekic I.
Psychiatric Hospital Sarajevo , Women, Sarajevo , Bosnia and Herzegovina
* Corresponding author.
Keywords: dementia, bihavioral and psychological symptoms of dementia, Agitation

Conflict of interest:

No

Article ID: EPV0827

Piloting a specialist mental health and pharmacist clinic for older adults in one scottish health region – preliminary findings

Stevenson G. *
Bate J.
Stratheden Hospital NHS Fife, Older Adult Mental Health , Fife , United Kingdom
* Corresponding author.
Keywords: Realistic Medicine, Polypharmacy, Older Adult Mental Health, Pharmacist

Conflict of interest:

No

Article ID: EPV0828

The impact of built environment on management of frailty in patients with neurocognitive disorders

Zamfir M.V. 1 *
Zamfir M.M. 2
1 Carol Davila University of Medicine and Pharmacy , Physiology - Neurosciences Division, Bucharest , Romania
2 “Ion Mincu” University of Architecture and Urbanism , Synthesis Of Architectural Design Department, Faculty of Architecture,, Bucharest , Romania
* Corresponding author.
Keywords: frailty, management, Architecture, neurocognitive disorders

Conflict of interest:

No

Article ID: EPV0829

The impact of architecture on mental health in nursing homes_3 case studies

Zamfir M.V. 1 *
Zamfir M.M. 2
1 Carol Davila University of Medicine and Pharmacy , Physiology - Neurosciences Division, Bucharest , Romania
2 “Ion Mincu” University of Architecture and Urbanism , Synthesis Of Architectural Design Department, Faculty of Architecture,, Bucharest , Romania
* Corresponding author.
Keywords: age-friendly architecture, mental health, senior, interdisciplinarity

Conflict of interest:

No

Article ID: EPV0832

Visual hallucinations in lewy bodies dementia: a case report and literature review.

Kabtni W. *
Baatout A.
Bencheikh Brahim C.
El Kefi H.
Oumaya A.
military hospital of Tunis , Psychiatry Department, Tunis , Tunisia
* Corresponding author.
Keywords: visual hallucinations, dementia with Lewy bodies

Conflict of interest:

No

Article ID: EPV0833

Maintenance electroconvulsive therapy in the spanish national health system: cost-effectiveness in elderly patients with affective and schizophrenia-spectrum disorders

Caballero-Gonzalez M. 1 2
Rentero Martin D. 1 2
Sánchez-Morla E. 1 2
Torio I. 1 2
Garcia Lopez A. 1
Fares-Otero N. 1
Rodriguez-Jimenez R. 1 2 *
1 Department of Psychiatry, Instituto De Investigación Sanitaria Hospital 12 De Octubre (imas12), Madrid , Spain
2 CIBERSAM, (biomedical Research Networking CentreIn Mental Health) , Madrid , Spain
* Corresponding author.
Keywords: Electroconvulsive Therapy, Schizophrenia, Bipolar disorder, elderly patients

Conflict of interest:

No

Article ID: EPV0838
Subjects: Abstract, Oncology and Psychiatry

Evaluation of the postoperative quality of life and dysfunctions in patients after combined total gastrectomy and esophagectomy.

Saito S. 1 *
Nakamura M. 2
Hosoya Y. 1
Yamaguchi H. 3
Kitayama J. 1
Lefor A. 1
Sata N. 1
1 Jichi Medical University , Surgery, Shimotsuke , Japan
2 Jikei University , School of Nursing, Adult Nursing, Tokyo , Japan
3 Jichi Medical University , Clinical Oncology, Shimotsuke , Japan
* Corresponding author.
Keywords: total gastrectomy, esophagectomy, postoperative QOL, postoperative dysfunction

Conflict of interest:

No

Article ID: EPV0841

Risk factors of interrupted treatment, recurrence and survival on patients with head and neck cancer in southern taiwan.

Wang S.H. 1 *
Chuang C.H. 2
1 Chi Mei Medical Center , Oncology, Tainan City , Taiwan
2 Chang Jung Christian University , Department of Nursing, Tainan City , Taiwan
* Corresponding author.
Keywords: head and neck cancer, interrupted treatment, risk factor, recurrence

Conflict of interest:

No

Article ID: EPV0842

Impact of psychiatric disorders on the course of oncological treatment

Darie C. 1 *
Paslaru A.-M. 2
Rebegea L. 3
Ciubara A. 4
1 Resident Psychiatrist at Psychiatric Hospital ’’Elisabeta Doamna’’ , Psychiatry, GALATI , Romania
2 PhD Student "Dunarea de Jos" University , Oncology, Galati , Romania
3 PhD, Assos. Professor "Dunarea de Jos" University , Oncology, Galati , Romania
4 Professor at "Dunarea de Jos" University, Senior Psychiatrist at Hospital of Psychiatry "Elisabeta Doamna" , Psychiatry, Galati , Romania
* Corresponding author.
Keywords: Dépression, Psychiatry, cancer, Oncology

Conflict of interest:

No

Article ID: EPV0843

Assessment of quality-of-life and functional outcomes in elderly cancer patients undergoing radiotherapy using the european organization for research and treatment of cancer quality-of-life questionnaire

Kitsou K.S. 1 *
Bakola M. 2
Kardamakis D. 3
Gourzis P. 4
Jelastopulu E. 5
1 School of Medicine, University of Patras , Postgraduate Program Of Public Health, Patras , Greece
2 School of Medicine, University of Patras , Greece , Postgraduate Program Of Public Health, Patras, Greece
3 School of Medicine, University of Patras , Greece , Radiation-oncology, Patras, Greece
4 School of Medicine, University of Patras , Greece , Psychiatry , Patras, Greece
5 School of Medicine, University of Patras , Greece , Public Health, Patras, Greece
* Corresponding author.
Keywords: EORTC-QLQ-C30; emotional functioning, cognitive functioning; cancer

Conflict of interest:

No

Article ID: EPV0844

Prescription of opioid drugs in a patient sample with head and neck tumors

Arrieta A. 1
Castelo B. 2
Mediavilla R. 3
Palao Á. 4 *
1 HOPSITAL LA PAZ , Psychiatry, Madrid , Spain
2 HOSPITAL UNIVERSITARIO LA PAZ , Oncologia , Madrid , Spain
3 La Paz University Hospital Research Health Institute (IdiPAZ) , Psychiatry, Clinical Psychology And Mental Health , Madrid , Spain
4 HOSPITAL UNIVERSITARIO LA PAZ , Psiquiatria , MADRID , Spain
* Corresponding author.
Keywords: opioids, Dependence, risk factors, head and neck cancer

Conflict of interest:

No

Article ID: EPV0845

Dignity therapy and quality of life in oncologic patients

Vasile D. *
Vasiliu O.
Mangalagiu A.
Petrescu B.
Candea C.
Tudor C.
University Emergency Central Military Hospital Dr. Carol Davila , Psychiatry, Bucharest , Romania
* Corresponding author.
Keywords: dignity therapy, quality of life, oncological patients

Article ID: EPV0847

Comorbid PTSD in treated with transplantation leucosis patients personality features, internal construct of the cancer illness image (ICii) and resources.

Vasileva A. *
Neznanov N.
Lukoshkina E.
V. M. Bekhterev Research Medical Centre for psychiatry and neurology , Psychiatry, saint petersburg, Russian Federation
* Corresponding author.
Keywords: personality profile, PTSD, Oncology, Anxiety

Conflict of interest:

No

Article ID: EPV0848

Emotional support and emotion regulation in patients undergoing hematopoietic stem cell transplantation (HSCT)

Torrijos M. 1
Torrea I. 1
Castellanos T. 1
López Álvarez I. 2
Rocamora González C. 2
Rodríguez-Vega B. 3
Palao Á. 3 *
1 Hospital Universitario La Paz, Psychiatry, Clinical Psychology And Mental Health Service , Madrid , Spain
2 HOSPITAL UNIVERSITARIO LA PAZ, Psychiatry, Clinical Psychology And Mental Health , Madrid , Spain
3 La Paz University Hospital Research Health Institute (IdiPAZ), Psychiatry, Clinical Psychology And Mental Health , Madrid , Spain
* Corresponding author.
Keywords: psychooncology, Hematopoietic Stem Cell Transplantation, Global Quality of Life, Emotional Support

Conflict of interest:

No

Article ID: EPV0855
Subjects: Abstract, Others

The relationship between cognitive functioning and psychopathology in patients with psychiatric disorders: a transdiagnostic network analysis

Chavez-Baldini U. 1 *
Nieman D. 1
Keestra A. 1
Verweij K. 1
Vulink N. 1
Wigman J. 2
Denys D. 1
1 Amsterdam University Medical Center , Psychiatry, Amsterdam , Netherlands
2 University Medical Center Groningen, University of Groningen , Interdisciplinary Center Psychopathology And Emotion Regulation (icpe), Groningen , Netherlands
* Corresponding author.
Keywords: transdiagnostic, network analysis, cognitive functioning, psychopathology

Conflict of interest:

No

Article ID: EPV0858

Mobbing-psychological violence at workplace

Idrizaj K. 1 *
Tepshi L. 2
Saliu A. 2
1 UHC “MOTHER THERESA” Tirana , Albania, Psychiatry , Tirana , Albania
2 University Hospital Center "Mother Teresa" , Tirana, Psychiatry , Tirana, Albania
* Corresponding author.
Keywords: Mobbing, psychiatric disorders, somatic disorders, social effects

Conflict of interest:

No

Article ID: EPV0859

Man and delusion - believing fast and slow

Khan F. 1 *
Breen E. 2
1 Galway Roscommon Mental Health , Psychiatry, Galway , Ireland
2 Mater Misericordiae University hospital DUblin , Adult Psychiatry, Dallas , Ireland
* Corresponding author.
Keywords: Believing fast and slow, Book review, Ethics and Psychiatry, Culture and Psychiatry

Conflict of interest:

No

Article ID: EPV0862

Burnout among nurses in emergency departments

El Kefi H.
Bouguerra N.E.H.
Bouali I.
Kefi K.
Bencheikh Brahim C.
Krir W.
Oumaya A. *
military hospital of tunis, Psycihiatric Unit , tunis , Tunisia
* Corresponding author.
Keywords: workload, burn out, nurses

Conflict of interest:

No

Article ID: EPV0865

Pedagogical criterions of parenting competence in assessment of good-enough parenting of persons under long-term psychiatrical observation

Zhuravleva E.
Rusakovskaya O. *
Moscow State University of Psychology and Education , Legal And Clinical Psychology, Moscow , Russian Federation
* Corresponding author.
Keywords: good-enough parenting, parenting

Article ID: EPV0866

Triguna, coping strategies and anxiety in early adulthood

S M. 1 *
Parameshwari M. 2
1 Sri Bhagavan Mahaveer Jain University , Psychology, Bangalore , India
2 Jain University , Psychology, Bangalore , India
* Corresponding author.
Keywords: coping strategies, Triguna, Anxiety, Early Adulthood

Conflict of interest:

No

Article ID: EPV0869

The impact of ADHD symptomatology on everyday life

Vňuková M. *
Ptáček R.
Raboch J.
Stehlíková M.
Tkáčová S.
First Faculty of Medicine, Psychiatry, Prague , Czech Republic
* Corresponding author.
Keywords: time management, ADHD, lifestyle

Article ID: EPV0870

The meaning of ethnic violence in terms of dissociative identity disorder in transitional societies

Yıldız H.
özel cihan hastanesi yenişehir mah.özden sok.no:35, Psikiyatri, izmit/kocaeli , Turkey
Keywords: transitional societies, dissociative identity, ethnic violence, defense mechanisms

Conflict of interest:

No

Article ID: EPV0871

Cognitive and emotional correlates of perceived stress and negative affect in college students: preliminary results of a longitudinal study

Amaral A.P. 1 *
Soares M.J. 2
Pereira A.T. 2
Bos S. 2
Nogueira V. 2
Madeira N. 2
Cabaços C. 2
Macedo A. 2
1 Polytechnic of Coimbra, Coimbra Health School , Coimbra , Portugal
2 University of Coimbra , Faculty of Medicine, Department of Psychological Medicine, Coimbra , Portugal
* Corresponding author.
Keywords: stress, negative affect, Rumination, self-blame

Conflict of interest:

No

Article ID: EPV0872

A factitious disorder case report: all is a lie?

Bravo Arráez M. 1 *
Mainar De Paz V. 2
Martín Alvarez C. 3
1 Hospital Universitario Severo Ochoa , Psiquiatría, Madrid , Spain
2 Centro de Salud Mental Maresme Nord , Psiquiatría, Barcelona , Spain
3 Hospital Universitario de Fuenlabrada , Psiquiatría, Madrid , Spain
* Corresponding author.
Keywords: factitious disorder

Conflict of interest:

No

Article ID: EPV0879

Styles of upbringing of children with different somatic health status

Goryacheva T. *
Komolov D.
Pirogov Russian National Research Medical University , Psychological-social Faculty, Moscow , Russian Federation
* Corresponding author.
Keywords: somatic health status, family upbrining

Conflict of interest:

No

Article ID: EPV0881

Success rates of smoking cessation therapies to patients with mental illness by video consultants or by treatment in the community: a randomized controlled trial

Hjorth P. 1 *
Sørensen M. 2
1 Psychiatry, Region of Southern Denmark , Vejle, Vejle, Denmark
2 Psychiatry, Region of Southern Denmark , Outpatients Clinics, Vejle, Denmark
* Corresponding author.
Keywords: randomized controlled trial, smoking cessation, video

Conflict of interest:

No

Article ID: EPV0882

Hysteria or frontotemporal dementia: the importance of clinical evolution in psychiatric diagnosis

Izquierdo De La Puente Á. 1 *
Del Sol Calderón P. 1
Garcia Moreno M. 1
Vizcaíno Da Silva M. 1
Mendez Gonzalez O. 1
Rodríguez Rodríguez A. 1
Fernández Fernández R. 2
Blanco R. 1
González-Villalobos Rincón I. 1
Martín M. 1
1 HOSPITAL UNIVERSITARIO PUERTA DE HIERRO MAJADAHONDA , Psychiatry, MADRID , Spain
2 Hospital Universitario HM Puerta del Sur , Psychiatry, Mostoles , Spain
* Corresponding author.
Keywords: Hysteria, frontotemporal dementia, diagnosis

Conflict of interest:

No

Article ID: EPV0883

Supporting obese patients through mobile health approaches: aren’t we forgetting mental health?

Silva S. 1
Cunha F. 1
Melo D. 2
Santos T. 3
Soares S. 4
Madeira N. 5 *
Oliveira I. 1
1 University of Aveiro , Department of Electronics, Telecommunication And Informatics (deti)/Institute of Electronics And Informatics Engineering (ieeta), Aveiro , Portugal
2 University of Aveiro , Department of Education And Psychology, Aveiro , Portugal
3 Baixo Vouga Hospital Centre, Psychiatry And Mental Health Department , Aveiro , Portugal
4 William James Center for Research, University of Aveiro , Department of Education And Psychology, Aveiro , Portugal
5 University of Coimbra , Faculty of Medicine, Department of Psychological Medicine, Coimbra , Portugal
* Corresponding author.
Keywords: obesity, eating disorders, mobile health

Conflict of interest:

No

Article ID: EPV0885

Resilience and social cognition in entrepreneurs within the vulnerable context of the base of the pyramid in the cristo rey neighborhood santa marta, colombia

Guardiola Esmeral A. 1
Pérez Correa K. 2 *
Pedraza Álvarez L. 3
Miranda L.F. 4
1 Universidad Cooperativa de Colombia, Facultad Ciencias Contables Administrativas Y De Comercio Internacional , Santa Marta, Colombia
2 Universidad Cooperativa de Colombia , Facultad De Psicología, Santa Marta, Colombia
3 Universidad del Magdalena , Facultad De Ciencias De La Salud, Santa Marta, Colombia
4 Universidad del Magdalena , Facultad De Educación, Santa Marta, Colombia
* Corresponding author.
Keywords: Social Cognition, entrepreneurs, Resilience, vulnerable

Conflict of interest:

No

Article ID: EPV0887

Dissociative phenomena in psychiatric patients

Ramos García E. *
Martínez Fernández Á.
Muñoz Domenjó A.
Molina Cambra R.
García-Poggio Fernández-Renau M.
Bianchi Ramos F.L.
Sagarra Arruego R.
Hernández Barrera M.
Ortega Moreno M.
Hospital Universitario de Móstoles , Psiquiatría, Móstoles, Madrid , Spain
* Corresponding author.
Keywords: dissociation

Conflict of interest:

No

Article ID: EPV0889

Connection between the cognitive style and copping strategies of the HIV-positive young patients

Sedova E. *
Gardanova Z.
Shashkin I.
Ilgov V.
Pirogov Russian National Research Medical University , Psychological-social Faculty, Moscow , Russian Federation
* Corresponding author.
Keywords: HIV, coping strategy, cognitive style

Conflict of interest:

No

Article ID: EPV0890

The difference of the psychological characteristics of the patients with hypochondriac and anxiety-depressive disorders

Sedova E. *
Gardanova Z.
Sobol P.
Burma A.
Pirogov Russian National Research Medical University , Psychological-social Faculty, Moscow , Russian Federation
* Corresponding author.
Keywords: hypochondriacal disorder, anxiety-depressive disorder

Conflict of interest:

No

Article ID: EPV0892

Nine premises of why physicians have little interest in scientific theories on human sciences: reflections and proposals for medical educators

Turato E.
University of Campinas , Medical Psychology And Psychiatry, Campinas , Brazil
Keywords: Qualitative Research, Medical Sociology, medical education

Conflict of interest:

No

Article ID: EPV0894

Capgras syndrome

Del Hoyo Mitjans R. *
Ezquerra B.
Carpintero Solano A.
Esteban Gavilán M.
Marquez Martín P.
López Vicente C.
Hospital Universitario de Guadalajara , Psychiatry, Guadalajara , Spain
* Corresponding author.
Keywords: Capgras syndrome, capgras, Delusional Misidentification

Conflict of interest:

No

Article ID: EPV0897

Frontotemporal dementia - diagnostic problems

Skiba A.
Filip M. *
Galecki P.
Medical University of Lodz , Department of Adult Psychiatry, Lodz , Poland
* Corresponding author.
Keywords: Frontotemporal, dementia, neurodegenerative disorders

Conflict of interest:

No

Article ID: EPV0898

Evaluation of physical health monitoring in a high secure forensic setting

Freeman R. *
Daud A.
West London NHS Trust, Broadmoor Hospital , Crowthorne , United Kingdom
* Corresponding author.
Keywords: physical health, forensic, CPA

Conflict of interest:

No

Article ID: EPV0899

Unlocking the mind: understanding the impact of mindfulness meditation on the cognitive functioning of children with ADHD

Gottlieb M. *
Bigelow H.
Fenesi B.
Western University , Faculty of Education, London , Canada
* Corresponding author.
Keywords: ADHD, Youth, Meditation, Neuroimaging

Conflict of interest:

No

Article ID: EPV0901

Algoneurodystrophy: the missing diagnosis

Henriques V. 1 *
Henriques S. 2
1 Hospital Garcia de Orta EPE , Psychiatry, Almada , Portugal
2 Hospital Central do Funchal , Psychiatry, Funchal , Portugal
* Corresponding author.
Keywords: algoneurodystrophy, functional syndrome, pseudoparalysis, conversion disorder

Conflict of interest:

No

Article ID: EPV0902

Epilepsy and non-aggressive criminal acts: a case report and a review of the literature

Ilzarbe L. 1 *
Anmella G. 2
Carreño M. 3
Freixa N. 4
Arbelo N. 1
Llach C. 1
Pintor L. 5
Balcells-Oliveró M. 4
1 Institute of Neuroscience/Hospital Clinic de Barcelona , Psychiatry And Psychology, Barcelona , Spain
2 Hospital Clínic de Barcelona , Bipolar And Depressive Disorders Unit, Department of Psychiatry And Psychology, Barcelona , Spain
3 Hospital Clínic de Barcelona, Epilepsy Unit, Department of Neurology, Neuroscience Institute , Barcelona , Spain
4 Hospital Clínic de Barcelona, Addictive Behaviours Unit, Department of Psychiatry And Psychology, Neuroscience Institute , Barcelona , Spain
5 Hospital Clínic de Barcelona, Psychosomatic And Liason Psychiatry Unit, Department of Psychiatry And Psychology, Neuroscience Institute , Barcelona , Spain
* Corresponding author.
Keywords: non-aggressive, Epilepsy, criminal responsibility, postictal psychosis

Conflict of interest:

No

Article ID: EPV0908

Impact of patient’s treatment (psychopharmacological vs ECT) on caregiver’s functionality.

Salvat N. 1 2 *
Labad J. 1
Urretavizcaya M. 2
De Arriba-Arnau A. 2
Crespo J.M. 2
Monreal J.A. 1
Palao Vidal D. 1
Menchon Magrina J. 2
Soria V. 2
1 Parc Taulí Hospital Universitario,I3PT, UAB, CIBERSAM , Salut Mental, Sabadell , Spain
2 Bellvitge University Hospital -IDIBELL-CIBERSAM , Psychiatry, Barcelona , Spain
* Corresponding author.
Keywords: ECT, functionality, Caregiver burden, Sheehan Disability Scale

Conflict of interest:

No

Article ID: EPV0909

Online time among adolescents and young adults: the relation between psychotic-like experiences and problematic internet use

Santesteban-Echarri O. 1 *
Goreis A. 2
Kafka J. 3
Scharinger C. 4
Addington J. 1
Felnhofer A. 3
Mossaheb N. 5
Plener P. 6
Kothgassner O. 4
1 University of Calgary , Psychiatry Department, Calgary , Canada
2 University of Vienna , Department of Applied Psychology: Health, Development, Enhancement And Intervention, Faculty of Psychology, Vienna, Austria
3 Medical University of Vienna , Department of Pediatrics And Adolescent Medicine, Vienna , Austria
4 Medical University of Vienna , Department of Child And Adolescent Psychiatry, Vienna , Austria
5 Medical University of Vienna , Department of Psychiatry And Psychotherapy, Clinical Division Of Social Psychiatry, Vienna , Austria
6 Medical University of Vienna , Department of Child And Adolescence Psychiatry, Vienna , Austria
* Corresponding author.
Keywords: Problematic Internet Use, adolescents, Psychotic-Like Experiences, Compulsive Internet Use Scale

Conflict of interest:

No

Article ID: EPV0911

Can the legislation affect the use of coercion in psychiatry?

Kacalak H.
Wolna D. *
Kiejna A.
Lower Silesian Center for Mental Health , Psychiatry, Wrocław , Poland
* Corresponding author.
Keywords: legislation, coercive measures, Psychiatric hospital, Polish law on Mental Health Protection

Conflict of interest:

No

Article ID: EPV0915

A retrospective study on a screening program for physical health problems in community mental health service users

Saraceni S. 1
Mattei G. 2 *
Baccari F. 3
Tedeschini E. 3
Starace F. 3
Galeazzi G. 4
1 University of Modena and Reggio Emilia, Department of Biomedical Science, Metabolic Science And Neuroscience , Modena , Italy
2 University of Modena and Reggio Emilia , Department of Economics, Modena , Italy
3 AUSL Modena , Department of Mental Health And Drug Abuse, Modena , Italy
4 University of Modena and Reggio Emilia , Department of Biomedical, Metabolic And Neural Sciences, Modena , Italy
* Corresponding author.
Keywords: physical health, antipsychotics, metabolic syndrome, screening practice

Conflict of interest:

No

Article ID: EPV0917
Subjects: Abstract, Pain

Portuguese version of the short-fear of dental pain questionnaire – preliminary psychometric study

Pereira A.T. 1
Xavier S. 1
Soares M.J. 1
Araújo A. 2 *
Cabaços C. 1
Marques C. 2
Macedo A. 1
1 University of Coimbra , Faculty of Medicine, Department of Psychological Medicine, Coimbra , Portugal
2 Faculty of Medicine, University of Coimbra , Institute of Psychological Medicine, Coimbra , Portugal
* Corresponding author.
Keywords: EFA & CFA, Dental Anxiety, fear of pain, short-Fear of Dental Pain Questionnaire

Conflict of interest:

No

Article ID: EPV0919

Autolytic attempt in a patient with phantom limb syndrome after orchiectomy. Case report

Del Sol Calderón P. 1 *
Izquierdo De La Puente Á. 1
Garcia Moreno M. 1
Carrajo Garcia C. 2
Rodriguez De Lorenzo M. 3
1 HOSPITAL UNIVERSITARIO PUERTA DE HIERRO MAJADAHONDA , Psychiatry, MADRID , Spain
2 HOSPITAL UNIVERSITARIO RAMON Y CAJAL , Psychiatry, MADRID , Spain
3 Hospital Universitario Ramon y Cajal , Psychiatry, Madrid , Spain
* Corresponding author.
Keywords: phantom limb, suicide attemp, pain

Conflict of interest:

No

Article ID: EPV0922

The efectiveness of a mindful self-compassion integrated with art therapy intervention to improve quality of life in chronic pain patients

Del Rio M. 1
Mellado L. 2
Torrijos M. 3
Palao Á. 3 *
1 AUTONOMA UNIVERSITY OF MADRID , Art Education And Visual Arts, Madrid , Spain
2 LA PAZ HOSPITAL ., Psychiatry, MADRID , Spain
3 Psychiatry and Mental Health Department La Paz Hospital ., Psychiatry, MADRID , Spain
* Corresponding author.
Keywords: MINDFUL SELF-COMPASSION, chronic pain, Art Therapy Intervention, quality of life

Conflict of interest:

No

Article ID: EPV0926

Multidisciplinary pain treatment in patients with somatic diseases and comorbid mental disorders, and chronic pain syndrome

Korostiy V. 1 2
1 Kharkiv National Medical University , Department of Psychiatry, Narcology And Medical Psychology, Kharkiv , Ukraine
2 Kharkiv National Medical University, University Clinic , Kharkiv , Ukraine
Keywords: chroniс pain, somatic diseases, pain catastrophising

Conflict of interest:

No

Article ID: EPV0937
Subjects: Abstract, Personality and Personality Disorders

Does the immaturity level of adventurous temperament type in asia differ from western culture?

Lee S.J. *
Kim M.J.
Kyungsung University , Department of Psychology, Busan , Korea , Republic of
* Corresponding author.
Keywords: temperament type, temperament and character inventory, adventurous temperament type, immauturity

Conflict of interest:

No

Article ID: EPV0938

Borderline and schizotaxia - on the moving boundaries of schizophrenia

Santos F. *
Gomes A.
Nascimento S.
Gonçalves E.
Centro Hospitalar Universitário do Algarve , Departamento De Psiquiatria E Saúde Mental, Faro , Portugal
* Corresponding author.
Keywords: borderline personality, Personality disorders, schizophrenia, borderline schizophrenia

Conflict of interest:

No

Article ID: EPV0940

Malignant narcissism: from “once upon a time” stories to stark family realities

Bellotti J. 1
Granatto S. 2
Toniolo G. 2
Corti G. 3 *
1 Universidad del Museo Social Argentino , Posgrado: Psquiatría Perinatal, Ciudad Autónoma de Buenos Aires, Argentina
2 Universidad del Museo Social Argentino, Formación Continua , Ciudad Autónoma de Buenos Aires, Argentina
3 Universidad del Museo Social Argentino , Posgrado: Psiquiatría Perinatal, Ciudad Autónoma de Buenos Aires, Argentina
* Corresponding author.
Keywords: Malignant Narcissism, Core Narcissistic Personality Disorder, Antisocial Behavior, Ego-syntonic Sadism

Conflict of interest:

No

Article ID: EPV0941

The cognitive therapy of adolescents with obsessive compulsive personality characteristics: focusing on self-criticisim

Gungor M. 1 *
Calli S. 1
Arslandogdu S. 1
Ozen E. 1
Yavuz M. 2
1 Istanbul Aydin University , Psychology Department, Küçükçekmece , Turkey
2 ISTANBUL AYDIN UNİVERSİTY, FRENCH LAPE HOSPİTAL , Psychology, Child And Adolescent Psychiatry, Küçükçekmece , Turkey
* Corresponding author.
Keywords: Obsessive, Compulsive, Personality, Therapy

Conflict of interest:

No

Article ID: EPV0942

Recurrence of suicidal behavior in patients with borderline personality disorder: about 30 cases

Kabtni W. 1 *
Baatout A. 2
Maamouri R. 3
Ben Cheikh C. 2
El Kefi H. 4
Oumaya A. 4
1 military hospital of Tunis , Psychiatry Department, Tunis , Tunisia
2 Military hospital of Tunis , Psychiatry Department, Tunis , Tunisia
3 Razi hospital , Psychiatry E, Ben arous, Tunisia
4 military hospital of tunis, Psycihiatric Unit , tunis , Tunisia
* Corresponding author.
Keywords: Suicide, predictive factor, Borderline Personality Disorder, prevalence

Conflict of interest:

No

Article ID: EPV0943

Features of downshifters’ self-concept

Lianguzova V.
Lomonosov Moscow State University , Psychology, Moscow , Russian Federation
Keywords: self-concept, downshifters, career, Personality

Conflict of interest:

No

Article ID: EPV0944

Validation of the arabic version of the 42-item version of Ryff’s psychological well-being scales (PWB) among undergraduates in kuwait

Alansari B.
Kuwait University , Psychology, Shuwaikh , Kuwait
Keywords: PWB / 42, Psychometric Properties

Conflict of interest:

No

Article ID: EPV0946

The driver’s chronic stress and chronic fatigue in traffic jams

Barabanshchikova V. 1
Sultanova F. 1
Boyarinov D. 1 *
Gubaidulina L. 1
Hatoyama K. 2
1 Lomonosov Moscow State University , Department of Psychology, Moscow , Russian Federation
2 Nagaoka University of Technology , Center for Academia–industry Fusion, Nagaoka, Japan
* Corresponding author.
Keywords: Traffic Jam, chronic fatigue, chronic stress

Conflict of interest:

No

Article ID: EPV0948

Chronic stress and anxiety among lawyers from civil service

Barabanshchikova V.
Sultanova F.
Ivanova S.
Boyarinov D.
Gubaidulina L. *
Lomonosov Moscow State University , Department of Psychology, Moscow , Russian Federation
* Corresponding author.
Keywords: lawyers, Anxiety, chronic stress

Conflict of interest:

No

Article ID: EPV0950

Affective neuroscience emotional system: differences between school refusing and non-school refusing help-seeking adolescents

Carpentieri R. 1
Lannoni M.E. 2
Listanti G. 2 *
Farulla C. 2
Mantovani B. 2
Guidobaldi L. 1
Sarlatto C. 2
1 " La Sapienza" University of Rome , Dynamic And Clinical Psychology, Rome , Italy
2 Sant'Andrea Hospital , Nesmos, Rome , Italy
* Corresponding author.
Keywords: Personality, social functioning, School Refusal, adolescence

Conflict of interest:

No

Article ID: EPV0953

Features of self-injurious behaviour in patients with bpd comorbid with alcohol dependence

Kuzminov V. 1 *
Linskiy I. 2
1 SI , Deparment Of Emergency Psychiatry And Addiction, Kharkiv , Ukraine
2 SI Institute of Neurology Psychiatry and Narcology NAMS of Ukraine , Deparment Of Emergency Psychiatry And Addiction, Kharkiv , Ukraine
* Corresponding author.
Keywords: Borderline personality disorder, alcohol dependence, self-harm

Conflict of interest:

No

Article ID: EPV0954

Personalized treatment for disturbed personality: an experience of group psychotherapy fo severe personality disorders in emilia romagna region

Pacetti M.
Centro Salute Mentale Forlì , Italy , Dsm-dp, Forlì, Italy
Keywords: personality disorders, group psychotherapy

Conflict of interest:

No

Article ID: EPV0955

Associations of childhood trauma with personality traits in healthy adults

Fares-Otero N. 1
Garcia Lopez A. 1
Fernandez Cancer P. 1
Rodrigo Hildalgo I. 1
Rodriguez Toscano E. 2
Rodriguez-Jimenez R. 1 2 *
1 Department of Psychiatry, Instituto De Investigación Sanitaria Hospital 12 De Octubre (imas12), Madrid , Spain
2 CIBERSAM, Biomedical Research Networking Centre In Mental Health , Madrid , Spain
* Corresponding author.
Keywords: Child Abuse, Bullying, Parental Violence, Personality, Adulthood

Conflict of interest:

No

Article ID: EPV0956
Subjects: Abstract, Philosophy and Psychiatry

Suitability of narrative approach to studying lived experiences of persons with mental illnesses

Laranjeira C.
Piaget Institute - RECI I&D, Higher School of Health Sciences , Viseu , Portugal
Keywords: narrative, lived experience, recovery, mental illness

Conflict of interest:

No

Article ID: EPV0957

The specifics of the formation of religious feelings in people with mental disorders who have committed socially dangerous acts

Makushkina O. *
Panchenko E.
Federal State Budgetary Institution “V.P. Serbsky National Medical Research Centre for Psychiatry and Narcology” of the Ministry of Health of the Russian Federation , Department of Forensic Psychiatric Prevention, Moscow , Russian Federation
* Corresponding author.
Keywords: religious feelings, protective factors, socially dangerous, mental disorders

Conflict of interest:

No

Article ID: EPV0958

Diffusion of identity in the context of sociocultural uncertainty: a clinical or a sociocultural phenomenon?

Sokolova E.
Pechnikova L.
Ryzhov A. *
Andreyuk K.
Lomonosov MSU , Faculty of Psychology, Moscow , Russian Federation
* Corresponding author.
Keywords: identity diffusion, manipulation, ambiguity, destructivity

Conflict of interest:

No

Article ID: EPV0960

Labyrinth, mannerism and schizophrenia

Doerr Zegers O.
Universidad Diego Portales , Center for Studies On Phenomenology And Psychiatry, Santiago , Chile
Keywords: labyrinth, mannerism, PHENOMENOLOGY, schizophrenia

Conflict of interest:

No

Article ID: EPV0964
Subjects: Abstract, Posttraumatic Stress Disorder

Key issues in the treatment of people traumatised by economic, psychological or physical abuse in buddhist groups

Anders A.I.M.
Ludwig-Maximilians-University Munich , Institute For Social And Cultural Anthropology, Munich , Germany
Keywords: slandering people with ‘rlung disease’, silencing and stigmatising the traumatised, economical, psychological and physical abuse in Buddhist groups, neologisms ‘karma purification’, ‘pure view’ and ‘guru yoga’ for silencing trauma, current developments in Buddhist groups and Tibetan medicine

Article ID: EPV0965

Great parents are traumatized by the unexpected death of one of their twins little sons!

Loucar S. 1 *
Ba F. 2
Sylla A. 3
1 régional hospital louga , Louga , LOUGA , Senegal
2 UFR santé Saint LOUIS , Neuropsychiatry, dakar , Senegal
3 CHNU Fann , Psychiatry, dakar , Senegal
* Corresponding author.
Keywords: grandparents, twins, traumatic death

Conflict of interest:

No

Article ID: EPV0966

The ability to use computer brain interfaces as an additional diagnostic procedure for post-traumatic stress disorder

Kuzminov V.
Minko O. *
SI Institute of Neurology Psychiatry and Narcology NAMS of Ukraine , Deparment Of Emergency Psychiatry And Addiction, Kharkiv , Ukraine
* Corresponding author.
Keywords: comorbid conditions, post-traumatic stress disorder, diagnosis

Conflict of interest:

No

Article ID: EPV0967

“You have to suffer”: traumatic stress as a consequence of institutional violence in obstetrics.

Molchanova E. 1 *
Kim E. 1
Karakai F. 1
Chirkina G. 2
1 American University in Central Asia , Psychology, Bishkek , Kyrgyzstan
2 Alliance of Reproductive Health, Obstetrics And Gynecology, Bishkek , Kyrgyzstan
* Corresponding author.
Keywords: violence, Traumatic stress, physical abuse, women in labor

Conflict of interest:

No

Article ID: EPV0968

I am not stressed, i am just ill: pathomorphosis of PTSD symptoms in Osh survivors.

Molchanova E.
American University in Central Asia , Psychology, Bishkek , Kyrgyzstan
Keywords: Traumatic stress, conversion symptoms, pathomorphosis

Conflict of interest:

No

Article ID: EPV0970

Cannabinoids effects in anxiety symptoms of ptsd: literature review and case report.

Costa Ferrera Da Silva M.L. 1
Coucheiro Limeres P. 2 *
Cerame Del Campo Á. 2
De Hita Santillana R. 2
1 Hospital Universitario Severo Ochoa , Psyhciatrist, leganes , Spain
2 Instituto psiquiatrico Jose Germain, Psyhciatric Trainee , leganes , Spain
* Corresponding author.
Keywords: New treatments, Cannabinoids, PTSD, Anxiety

Conflict of interest:

No

Article ID: EPV0971

Correlations of family health deterioration and poststress psychological maladaptation in combatants

Markov A. 1 *
Markova M. 2
Rosinsky G. 1
Chernyaev M. 1
Driuchenko M. 3
1 Kharkiv Medical Academy of Postgraduate Education , Sexology, Medical Psychology Medical And Psychological Rehabilitation, Kharkiv, Ukraine
2 Kharkiv Medical Academy of Postgraduate Education , Sexology, Medical Psychology, Medical Abd Psychological Rehabilitation, Kharkiv , Ukraine
3 Uzhhorod national university , Department of Neuro Rehabilitation With Courses Of Medical Psychology, Pulmonology And Physiology, Uzhhorod , Ukraine
* Corresponding author.
Keywords: poststress psychological maladaptation, family health deterioration, participants in military actions

Conflict of interest:

No

Article ID: EPV0972

Phenomenology of psychopathological disorders in combatants with eyes injury and varying severity of post-stress psychological maladaptation

Markova M. 1 *
Abdryahymov R. 1
Abdriakhimova T. 2
Kleban K. 2
Sapon D. 2
Markov A. 3
1 Kharkiv Medical Academy of Postgraduate Education , Sexology, Medical Psychology, Medical Abd Psychological Rehabilitation, Kharkiv , Ukraine
2 Bogomolets National Medical University , Medical Psychology, Psychosomatic Medicine and Psychotherapy, Киев , Ukraine
3 Kharkiv Medical Academy of Postgraduate Education , Sexology, Medical Psychology Medical And Psychological Rehabilitation, Kharkiv , Ukraine
* Corresponding author.
Keywords: post-stress psychological maladaptation, eyes injury, combatants

Conflict of interest:

No

Article ID: EPV0973

The relationship between trauma, HIV infection and neurocognitive impairment: a systematic review of observational epidemiological studies

Spies G. 1 *
Wieler H. 2
Masilela L. 3
Konkiewitz E. 4
Mall S. 3
Seedat S. 5
1 Stellenbosch University , Department of Psychiatry, Cape Town , South Africa
2 Berlin, Psychology, Berlin , Germany
3 University of the Witwatersrand , Division Of Epidemiology And Biostatistics, Johannesburg , South Africa
4 Universidade Federal da Grande Dourados , Psychiatry, Dourados , Brazil
5 Stellenbosch University , Psychiatry, Cape Town , South Africa
* Corresponding author.
Keywords: HIV/AIDS, trauma, Neurocognitive impairment, Systematic Review

Conflict of interest:

No

Article ID: EPV0979

Clinical incidence of psycho traumas in outpatient consultation at the ar-razi hospital salé

Yamoul M. 1 *
Benzahra S. 2
Karara A. 3
Laboudi F. 1
Ouanass A. 1
1 CHU IBN SINA , Ar-razi Hospital, SALE, Morocco
2 CHU IBN SINA , Hospital Arrazi Sale, SALE, Morocco
3 CHU Ibn Sina / Arrazi -SALE hospital , Psychiatry, SALE, Morocco
* Corresponding author.
Keywords: incidence, PTSD, trauma, psychotraumatisme

Conflict of interest:

No

Article ID: EPV0980

Psychotrauma: clinical polymorphism and misdiagnosis

Zaki A. *
El Omari F.
ar-razi hospital , Psychiatry, salé, Morocco
* Corresponding author.
Keywords: Post traumatic stress disorder, delirium, psychotrauma, acute stress reaction

Conflict of interest:

No

Article ID: EPV0983
Subjects: Abstract, Prevention of Mental Disorders

Evaluation of the impact of a psychoeducational intervention in suicidal ideation and depressive symptoms in adolescents

Amaral A.P. *
Sampaio J.
Pocinho M.
Polytechnic of Coimbra, Coimbra Health School , Coimbra , Portugal
* Corresponding author.
Keywords: Prevention, Suicidal ideation, adolescents, Depressive symptoms

Conflict of interest:

No

Article ID: EPV0984

Promoting an adaptive relationship with eating behavior in young adults – a preliminary case series study

Amaral A.P. 1 *
Nogueira P. 1
Duarte C. 2
Castilho P. 3
1 Polytechnic of Coimbra, Coimbra Health School , Coimbra , Portugal
2 University of Leeds , Faculty of Medicine & Health, Leeds , United Kingdom
3 University of Coimbra , Faculty of Psychology And Educational Sciences, Coimbra , Portugal
* Corresponding author.
Keywords: eating behavior, intuitive eating, Intervention, shame

Conflict of interest:

No

Article ID: EPV0985

Impact of a nutritional psychology intervention on quality of life of the elderly: a preliminary study

Simões P.
Amaral A.P. *
Rocha C.
Polytechnic of Coimbra, Coimbra Health School , Coimbra , Portugal
* Corresponding author.
Keywords: quality of life, health promotion, social isolation, Elderly

Conflict of interest:

No

Article ID: EPV0986

Mindfulness and burnout prevention among mental health professionals.

Matyushin V.
Blinnikova I. *
Moscow State University , Psychology, Moscow , Russian Federation
* Corresponding author.
Keywords: Mindfulness, Burnout

Conflict of interest:

No

Article ID: EPV0987

Sagacity college: a complementary approach and a prevention model offered in the community to promote good mental health for seniors

Lord M.-M. 1 *
Briand C. 2
Hand C. 3
1 Université du Québec à Trois-Rivières , Ergotherapie, Trois-Rivières , Canada
2 Université du Québec à Trois-Rivières , Department of Educational Sciences, Trois-Rivières , Canada
3 Western University , Occupational Therapy, London , Canada
* Corresponding author.
Keywords: Prevention, Seniors, learning

Conflict of interest:

No

Article ID: EPV0989

“Progetto mary”: work with patients, thinking about children.

Pesce L. *
Imoli M.
Cristofori S.
Dell'Eva A.M.
Agostini C.
APSS , Pychiatry - Unit 1, trento, Italy
* Corresponding author.
Keywords: prevention+children+parental mental illness+family intervention

Conflict of interest:

No

Article ID: EPV0990

Supporting research: the RANZCP foundation

Peters A.
Royal Australian and New Zealand College of Psychiatrists , Chief Executive Officer, Melbourne , Australia
Keywords: research, grants

Article ID: EPV0991

Reducing stress in mental health nurses: the development of a comprehensive approach to build resilience in the working place

Semenova N. 1 *
Burygina L. 2
Kryukova E. 3
Palin A. 3
1 Moscow Research Institute of Psychiatry – a branch of V.Serbsky National Medical Research Centre for Psychiatry and Narcology , Department of Clinical, Social And Biological Studies Of Psychotic Spectrum Disorders, Moscow , Russian Federation
2 Psychiatric hospital No . 4 named after P.B.Gannushkin, Administration, Moscow , Russian Federation
3 Psychiatric hospital No. 4 named after P.B.Gannushkin, Rehabilitation, Moscow , Russian Federation
* Corresponding author.
Keywords: mental health nurses, Resilience, Burnout

Conflict of interest:

No

Article ID: EPV0993

Hope and psychological resilience: cross cultural study

Alali T.
Kuwait University , Psychology Department, Kuwait , Kuwait
Keywords: hope, trauma, psychopathology, psychological resilience

Conflict of interest:

No

Article ID: EPV0994

Factorial invariance of the adult hope scale among undergraduates in kuwait

Alali T.
Kuwait University , Psychology Department, Kuwait , Kuwait
Keywords: hope, Adult Hope Scale

Conflict of interest:

No

Article ID: EPV0996

Prevention of tobacco use among youth: systematic review of the literature and meta-analysis

Nosari G. 1 *
Calorio F. 1
Salazar De Pablo G. 2
Di Marco B. 1
Di Maggio L. 1
Famularo I. 1
Ruzzi F. 1
Politi P. 1
Fusar-Poli P. 3
1 Università Degli Studi di Pavia , Psychiatry, Pavia , Italy
2 King’s College London , Institute of Psychiatry, Psychology, And Neuroscience, London , United Kingdom
3 King’s College London , Department of Psychosis Studies, London , United Kingdom
* Corresponding author.
Keywords: Systematic Review, Prevention, Smoke, meta-analysis

Conflict of interest:

No

Article ID: EPV0997

2. A glance to perinatal mental health: screening and early intervention of bonding disorders as a strategy for preventing complex trauma.

Hernandez-Santillan G. 1 *
Bravo-Ortiz M.F. 2
Alcami Pertejo M. 2
Fernandez-Sanchez A. 2
Lahera G. 3
1 Universidad Autónoma de Madrid , Medicine, Madrid , Spain
2 La Paz University Hospital Research Health Institute (IdiPAZ) , Psychiatry, Clinical Psychology And Mental Health, Madrid , Spain
3 University of Alcala , School of Medicine, Alcalá de Henares , Spain
* Corresponding author.
Keywords: bonding disorders, complex trauma, perinatal mental health, Prevention

Conflict of interest:

No

Article ID: EPV0999
Subjects: Abstract, Promotion of Mental Health

Future perspectives of the institute of cognitive modeling within mental health care delivery in ukraine

Lahutina S. 1 *
Subbota S. 2
1 Bogomolets National Medical University , Institute of Cognitive Modeling, Medical Psychology, Psychosomatic Medicine and Psychotherapy Department, Kyiv , Ukraine
2 Institute of Cognitive Modeling, Scientific Department, Kyiv , Ukraine
* Corresponding author.
Keywords: mental health awareness, psychoeducation, Institute of Cognitive Modeling, mental health

Conflict of interest:

No

Article ID: EPV1000

Re-enrolling young people who had dropped out of high school. A follow up study describing experiences with a public intervention program

Ramsdal G. 1
Wynn R. 2 *
1 UiT The Arctic University of Norway , Social Education, Harstad , Norway
2 UiT The Arctic University of Norway , Clinical Medicine, Tromso , Norway
* Corresponding author.
Keywords: School drop-out, Motivation, Relationships, Coping

Conflict of interest:

No

Article ID: EPV1001

Effect of stress on self-reported psychopathic experience

Joo J. 1 2 *
Goo A.J. 3
1 Seoul National University Hospital , Department of Public Health Medical Service, daehak-ro, Jongno-gu, Seoul , Korea , Republic of
2 National Center for Mental Health , Department of Health Promotion, Yongmasan-ro, Gwanjin-gu, Seoul , Korea , Republic of
3 National Center for Mental Health , Department of Health Promotion, Yongmasan-ro, Gwanjin-gu, Seoul , Korea , Republic of
* Corresponding author.
Keywords: stress-vulnerability model, stress, psychopathology, Psychotic Symptoms

Conflict of interest:

No

Article ID: EPV1007
Subjects: Abstract, Psychoneuroimmunology

Gut microbiota and mental health

Hervías Higueras P. *
Benítez Alonso M.
Correas Lauffer J.
HOSPITAL UNIVERSITARIO DEL HENARES , PsiquiatrÍa , COSLADA, MADRID, Spain
* Corresponding author.
Keywords: gut microbiota, mental health, First Episode Psychosis, dysbiosis

Conflict of interest:

No

Article ID: EPV1009

Anxiety and dissociation: the debut of multiple sclerosis

Ortega G. 1 *
Mantilla Reyes M.F. 1
Martinez M.C. 2
Vendrell J. 1
Vargas S. 1
1 Hospital Universitari Vall d'Hebron , Psychiatry, Barcelona , Spain
2 Hospital Sant Rafael , Psychiatry, Barcelona , Spain
* Corresponding author.
Keywords: multiple sclerosis, Anxiety, dissociation

Conflict of interest:

No

Article ID: EPV1012

Immune types of asthenic disorders in schizophrenia

Zozulya S. 1 *
Yakimets A. 2
Oleichik I. 2
Klyushnik T. 1
1 Federal State Budgetary Scientific Institution "Mental Health Research Centre" , Laboratory of Neuroimmunology, Moscow , Russian Federation
2 Federal State Budgetary Scientific Institution "Mental Health Research Centre" , Department of Endogenous Mental Disorders And Affective Conditions, Moscow , Russian Federation
* Corresponding author.
Keywords: schizophrenia, endogenous asthenia, immune types, inflammation markers

Conflict of interest:

No

Article ID: EPV1013

Type 17 immune response facilitates progression of inflammation and correlates with cognition in stable schizophrenia

Borovcanin M. 1 *
Minic Janicijevic S. 2
Jovanovic I. 3
Gajovic N. 3
Jurisevic M. 4
Arsenijevic N. 3
1 Faculty of Medical Sciences, University of Kragujevac , Department of Psychiatry, Kragujevac , Serbia
2 Faculty of Medical Sciences, University of Kragujevac , Phd Student At Department of Psychiatry, Kragujevac , Serbia
3 Faculty of Medical Sciences, University of Kragujevac , Center for Molecular Medicine and Stem Cell Research, Kragujevac , Serbia
4 Faculty of Medical Sciences, University of Kragujevac , Department of Clinical Pharmacy, Kragujevac , Serbia
* Corresponding author.
Keywords: IL-17, schizophrenia, Inflammation, Cognition

Conflict of interest:

No

Article ID: EPV1019
Subjects: Abstract, Psychopathology

Mythomania: a review and a case report

Banzo-Arguis C. *
Matas Ochoa A.M.
Nava P.
Rodríguez-Quiroga A.
Martinez De Velasco-Soriano R.
Mora F.
INFANTA LEONOR HOSPITAL , Psychiatry, Madrid (Vallecas), Spain
* Corresponding author.
Keywords: Mythomania, Pseudologia Fantastica, treatment, Pathological lying

Conflict of interest:

No

Article ID: EPV1020

Habit formation in attention deficit hyperactivity disorder (ADHD): protocol for a scoping review

Musullulu H.
Bernacer J. *
Murillo J.
Arrondo G.
University of Navarra , Mind-brain Group; Institute For Culture And Society (ics), Pamplona, Spain
* Corresponding author.
Keywords: ADHD, habits, Systematic Review, protocol

Conflict of interest:

No

Article ID: EPV1021

Does an association between social functioning and mentally-ill terrorists exist? A psychological perspective on gap in mental health rehabilitation

Curtolo C.
University , Department of Law, Macerata , Italy
Keywords: agentivity, rehabilitation, mood, agentivity, rehabilitation, mood

Conflict of interest:

No

Article ID: EPV1023

Chiari malformation and attention deficit hyperactivity disorder

Dubow A. 1 *
Mourot A. 1
Tourjman S. 2
1 University of Montreal , Psychiatry, Montreal , Canada
2 IUSMM, Psychiatry, Montreal , Canada
* Corresponding author.
Keywords: Cognition, Attention deficit disorder, Arnold Chiari Malformation, Cerebellum

Article ID: EPV1025

Dissociative symptoms in neurosis and psychosis: a reflection regarding a clinical case

Fragoeiro C. 1 *
Ribeiro N. 2
Frias P. 1
Palha Fernandes E. 3
Valente P. 1
Milheiro C. 1
Brito L. 1
Mota I. 1
Patrício L. 1
1 Hospital de Magalhães Lemos , Psychiatry, Porto , Portugal
2 Centro Hospitalar de Setúbal , Psychiatry, Setubal , Portugal
3 Unidade Local de Saude Alto Minho , Psychiatry, Viana do Castelo , Portugal
* Corresponding author.
Keywords: neurosis, psychosis, dissociation, psychodynamic

Conflict of interest:

No

Article ID: EPV1026

“I got myself a family": delusions of having a spouse and children

Marinho G. 1 *
Ganhao I. 2
1 CHPL , Ccsmo, Lisbon , Portugal
2 Centro Hospitalar Psiquiátrico de Lisboa , Clínica 6, Lisbon , Portugal
* Corresponding author.
Keywords: delusions of having a spouse, paternity/maternity delusions, delusional procreation syndrome

Conflict of interest:

No

Article ID: EPV1030

A mexican werewolf in barcelona: clinical lycanthropy due to cerebellar lesion and hydrocephalus

Arbelo N. 1 *
Gómez-Ramiro M. 1
Ferrés A. 2
Oliveras C. 1
Ilzarbe L. 1
Llach C. 1
Cárdenas R. 3
Parellada E. 1 4 5 6
1 Institute of Neuroscience , Hospital Clínic de Barcelona, Barcelona Clínic Schizophrenia Unit. Department of Psychiatry And Psychology, Barcelona , Spain
2 Institute of Neuroscience , Hospital Clínic de Barcelona, Department of Neurosurgery, Barcelona , Spain
3 Hospital Humberto Elorza de Illapel, Department of Psychiatry, Illapel , Chile
4 University of Barcelona , Department of Medicine, Barcelona , Spain
5 Institut d’Investigacions Biomèdiques August Pi I Sunyer , Idibaps, Barcelona , Spain
6 Centro de Investigación Biomédica en Red en Salud Mental, Cibersam, Madrid , Spain
* Corresponding author.
Keywords: lycanthropy, cerebellum, hydrocephalus, psychosis

Conflict of interest:

No

Article ID: EPV1032

Analysis of mental disorder rates among cardiovascular patients after successful surgical treatment

Chernus N. 1 *
Gorenkov R. 1
Sivkova S. 2
Sivkov A. 3
Zolotovickaja A. 2
Sivkov S. 4
Savina T. 2
Krutofal A. 2
1 I.M. Sechenov First Moscow State Medical University , The Higher Healthcare Organization Management, Moscow , Russian Federation
2 I.M. Sechenov First Moscow State Medical University , The Outpatient Care Department, Moscow , Russian Federation
3 I.M. Sechenov First Moscow State Medical University , The Department of Clinical Pharmacology And Internal Diseases Propaedeutics Of The Medical Faculty, Moscow , Russian Federation
4 I.M. Sechenov First Moscow State Medical University , Department of Clinical Pharmacology And Internal Diseases Propaedeutics, moscow , Russian Federation
* Corresponding author.
Keywords: somatoform disorders, panic attacks, adaptation disorders

Conflict of interest:

No

Article ID: EPV1033

Peculiarities of pain syndrome in patients with cardiac syndrome X

Chernus N. 1 *
Gorenkov R. 2
Sivkova S. 1
Sivkov A. 3
Sivkov S. 4
Zolotovickaja A. 1
Savina T. 1
Krutofal A. 1
1 I.M. Sechenov First Moscow State Medical University , The Outpatient Care Department, Moscow , Russian Federation
2 I.M. Sechenov First Moscow State Medical University , The Higher Healthcare Organization Management, Moscow , Russian Federation
3 I.M. Sechenov First Moscow State Medical University , The Department of Clinical Pharmacology And Internal Diseases Propaedeutics Of The Medical Faculty, Moscow , Russian Federation
4 I.M. Sechenov First Moscow State Medical University , The Department of Clinical Pharmacology And Internal Diseases Propaedeutics, moscow , Russian Federation
* Corresponding author.
Keywords: Organic anxiety disorders, depressive disorders, emotionally labile mental disorders

Conflict of interest:

No

Article ID: EPV1034

Assessment of hostility / aggression in esophagitis patent

Chernus N. 1 *
Gorenkov R. 2
Sivkov S. 3
Sivkov A. 1
Sivkova S. 1
Savina T. 1
Zolotovickaja A. 1
Krutofal A. 1
1 I.M. Sechenov First Moscow State Medical University , The Outpatient Care Department, Moscow , Russian Federation
2 I.M. Sechenov First Moscow State Medical University , The Higher Healthcare Organization Management, Moscow , Russian Federation
3 I.M. Sechenov First Moscow State Medical University , The Department of Clinical Pharmacology And Internal Diseases Propaedeutics, moscow , Russian Federation
* Corresponding author.
Keywords: I-concept, Anxiety, Dépression, hostility

Conflict of interest:

No

Article ID: EPV1035

Gender- and age-related quality of life differences in rheumatoid arthritis patients

Chernus N. 1 *
Gorenkov R. 2
Sivkov S. 3
Sivkova S. 1
Sivkov A. 4
Savina T. 1
Zolotovickaja A. 1
Krutofal A. 1
1 I.M. Sechenov First Moscow State Medical University , The Outpatient Care Department, Moscow , Russian Federation
2 I.M. Sechenov First Moscow State Medical University , The Higher Healthcare Organization Management, Moscow , Russian Federation
3 I.M. Sechenov First Moscow State Medical University , The Department of Clinical Pharmacology And Internal Diseases Propaedeutics, moscow , Russian Federation
4 I.M. Sechenov First Moscow State Medical University , The Department of Clinical Pharmacology And Internal Diseases Propaedeutics Of The Medical Faculty, Moscow , Russian Federation
* Corresponding author.
Keywords: quality of life, rheumatoid arthritis

Conflict of interest:

No

Article ID: EPV1036

Sleep disorders in psychosomatic conditions

Chernus N. 1 *
Gorenkov R. 2
Sivkov S. 3
Sivkova S. 1
Savina T. 1
Sivkov A. 4
Zolotovickaja A. 1
Krutofal A. 1
1 I.M. Sechenov First Moscow State Medical University , The Outpatient Care Department, Moscow , Russian Federation
2 I.M. Sechenov First Moscow State Medical University , The Higher Healthcare Organization Management, Moscow , Russian Federation
3 I.M. Sechenov First Moscow State Medical University , The Department of Clinical Pharmacology And Internal Diseases Propaedeutics, moscow , Russian Federation
4 I.M. Sechenov First Moscow State Medical University , The Department of Clinical Pharmacology And Internal Diseases Propaedeutics Of The Medical Faculty, Moscow , Russian Federation
* Corresponding author.
Keywords: Anxiety, sleep disorders, Psychosomatic Conditions, depression symptoms

Conflict of interest:

No

Article ID: EPV1044
Subjects: Abstract, Psychopharmacology and Pharmacoeconomics

Determinants of medication adherence in patients with psychiatric disorders

Ben Hamadi A. 1 *
Hamdoun J. 1
Ben Ammar H. 1
Aissa A. 2
Khelifa E. 3
Zouhaier E.H. 2
1 Razi hospital , Psychiatry F, Manouba , Tunisia
2 Razi Hospital , F Department, Tunis , Tunisia
3 University of Tunis El Manar- Faculty of medecine of Tunis , Psychiatry - Razi Hospital, Tunis , Tunisia
* Corresponding author.
Keywords: Adherence, psychiatric disorders

Conflict of interest:

No

Article ID: EPV1045

Association between the use of an antipsychotic drug and changes in lipid profile: a meta-analysis

Brierley B. 1 *
Bak M. 2
Drukker M. 2
1 Maastricht University , Faculty of Health Medicine and Life Sciences, Maastricht , Netherlands
2 Maastricht University , Department of Psychiatry & Neuropsychology, Maastricht , Netherlands
* Corresponding author.
Keywords: lipid profile, cholesterol, Antipsychotic medication, adverse effects

Conflict of interest:

No

Article ID: EPV1048

Drug induced liver injury in the course of a treatmen with clozapine: a case report.

Cerame Del Campo Á. 1 *
De Hita Santillana R. 1
Franco Soler A.V. 2
Coucheiro Limeres P. 1
De La Mata Ruiz I. 3
Aguilar Romero M.D.C. 4
Costa Ferrera Da Silva M.L. 5
1 Instituto psiquiatrico Jose Germain , Psyhciatric Trainee, leganes , Spain
2 Instituto psiquiatrico Jose Germain , Clinical Psychology Trainee, leganes , Spain
3 Instituto Psiquiatrico José Germain , Psiquiatría, Leganés , Spain
4 Hospital Severo Ochoa , Psychiatry, Leganés , Spain
5 Hospital Universitario Severo Ochoa , Psyhciatrist, leganes , Spain
* Corresponding author.
Keywords: clozapine, hepatotoxicity, adverse effect, Drug induced liver injury

Conflict of interest:

No

Article ID: EPV1050

Management of antidepressants drugs during breastfeeding

Rodríguez Mercado C.M. 1
Fernández Ortiz S.L. 2 *
1 SESPA , Centro De Salud Mental La Calzada, Gijón , Spain
2 SESPA , Centro De Salud La Calzada, Gijón , Spain
* Corresponding author.
Keywords: Side Effects, breastfeeding, Antidepressants, mental disorders

Conflict of interest:

No

Article ID: EPV1051

Mirtazapine interactions with monoamine oxidase: a systematic review

Guinovart M. 1 *
Guinovart A. 2
Cardoner Álvarez N. 1
Monreal J.A. 1
Palao Vidal D. 1
1 Parc Taulí University Hospital . Autonomous University of Barcelona (UAB). I3PT. CIBERSAM, Department of Mental Health, Sabadell , Spain
2 Miguel Servet University Hospital , Department of Clinical Neurophysiology, Zaragoza , Spain
* Corresponding author.
Keywords: Monoamine oxidase, Mirtazapine, psychopharmacology, interactions

Conflict of interest:

No

Article ID: EPV1052

Neuroleptic malignant syndrome as a consequence of the use of low dose olanzapine: a matter to consider

Inca Torres K. 1 *
Renau Mínguez B. 2
Juanes A. 3
Cruz Fourcade J.F. 4
Alvarez-Sesmero S. 4
Losada C. 4
1 Hospital 12 de Octubre, Psychiatry, Madrid , Spain
2 Complejo Asistencial de Zamora , Psiquiatria, Zamora , Spain
3 Hospital Universitario 12 de Octubre, Psychiatry, Madrid , Spain
4 Hospital 12 de Octubre, Liaison Psychiatry, Madrid , Spain
* Corresponding author.
Keywords: adverse reaction, antipsychotics, Neuroleptic malignant syndrome, olanzapine

Conflict of interest:

No

Article ID: EPV1053

Importance of prolonged release antipsychotic treatment. A case report.

López Sánchez E.J. *
Sánchez Gayango A.
Hospital Nuestra Señora de Valme , Psiquiatría, Sevilla , Spain
* Corresponding author.
Keywords: schizophrenia, antipsychotic, treatment, aripiprazole

Conflict of interest:

No

Article ID: EPV1055

When risperidone has a psychotic effect

Rodrigues S. 1
Martins D. 2 *
Marinho M. 2
1 Centro Hospitalar e Universitário do Porto , Child And Adolescent Psychiatry, Porto , Portugal
2 Hospital de Magalhães Lemos , Hospital De Magalhães Lemos, Porto , Portugal
* Corresponding author.
Keywords: psychosis, schizofrenia, paradoxal effect, risperidone

Conflict of interest:

No

Article ID: EPV1058

A cohort study of serotonin–norepinephrine re-uptake inhibitors and risk of hyponatremia

Nilsson E. 1 *
Evald E. 1
Carrero J. 2
1 Örebro University , School of Medical Sciences, Örebro , Sweden
2 Karolinska Institutet , Medical Epidemiology And Biostatistics, Stockholm , Sweden
* Corresponding author.
Keywords: hyponatremia, pharmacopeidemiology, Antidepressants

Conflict of interest:

No

Article ID: EPV1059

Lithium and thyroid: are we paying enough attention?

Melo M.
Nogueira V. *
Fernandes R.
Centro Hospitalar Psiquiátrico de Lisboa , Psiquiatria, Lisboa , Portugal
* Corresponding author.
Keywords: lithium treatment, lithium toxicity, thyroid dysfunction, thyroid autoimmunity

Conflict of interest:

No

Article ID: EPV1061

Review of psychotropic drugs approved for adhd in spain

Romero Martin C. 1 *
Hernandez Romero M.D.C. 2
Padilla Romero P. 3
1 Hospital General Nuestra Señora del Prado, Hospital Pharmacy , Talavera, Toledo , Spain
2 Servicio Andaluz de Salud , Primary Care Pharmacy, Sevilla , Spain
3 Hospital General Nuestra Señora del Prado , Psychiatry, Talavera, Toledo , Spain
* Corresponding author.
Keywords: PHARMACOLOGICAL TREATMENT, Psychostimulant psychotropic, ADHD, Non-psychostimulant psychotropic

Conflict of interest:

No

Article ID: EPV1062

Fighting nihilism: can we do more for our long term chronic patients?

Salcedo Jarabo D. 1 *
García Escudero A. 2
Marquez Martín P. 3
Cejas Méndez M.D.R. 4
1 HOSPITAL UNIVERSITARIO DE CANARIAS , Usm Icod De Los Vinos, SANTA CRUZ DE TENERIFE, Spain
2 HOSPITAL UNIVERSITARIO NUESTRA SEÑORA DE LA CANDELARIA , Usm Adeje, SANTA CRUZ DE TENERIFE, Spain
3 HOSPITAL GENERAL UNIVERSITARIO DE GUADALAJARA , Centro De Salud Mental El Ferial, GUADALAJARA, Spain
4 HOSPITAL UNIVERSITARIO DE CANARIAS , PsiquiatrÍa, SANTA CRUZ DE TENERIFE, Spain
* Corresponding author.
Keywords: paliperidone, satisfaction, DEPOT, schizophrenia

Conflict of interest:

No

Article ID: EPV1064

Methylphenidate, a companion in young age and a foe in old age?

Arts M.H.L. 1 *
Petrykiv S. 2
De Jonge L. 3
1 GGZ Westelijk Noord-Brabant , Geriatric Psychiatry And Neuropsychiatry, Halsteren , Netherlands
2 GGZ WNB , Psychiatry, Halsteren , Netherlands
3 Leonardo Scientific Research Institute , Neuropsychiatry, Groningen , Netherlands
* Corresponding author.
Keywords: neurodegeneration, Methylphenidate, Inflammation, dementia

Conflict of interest:

No

Article ID: EPV1068

Aripiprazole and hypogalactorrhea in postpartum

Mantilla Reyes M.F. *
Ximenez De Embun Ferrer I.
Roca Lecumberri A.
Naranjo Díaz C.
Sole Roige E.
Torres Gimenez A.
Andrés Perpiñá S.
García – Esteve L.
Hospital Clínic de Barcelona , Institut Clínic De Neurociencies (icn). Unidad De Salud Mental Perinatal, Barcelona , Spain
* Corresponding author.
Keywords: breastfeeding, lactation, aripiprazole

Conflict of interest:

No

Article ID: EPV1070

Raynaud secondary to aripiprazole. An unexpected adverse reaction.

Ruiz Martínez G.M. 1 *
Soldado Rodriguez L. 2
Coca Cruz C. 2
1 Neurotraumatological Hospital , Mental Health Hospitalization Unit, Jaen , Spain
2 Neurotraumatological Hospital , Mir, Mental Health Unit, Jaen , Spain
* Corresponding author.
Keywords: psychosis, aripiprazole, raynaud, adverse reaction

Conflict of interest:

No

Article ID: EPV1073

A prevalence study of clozapine-induced sialorrhea (CIS): preliminary findings.

Fuentes J.J. *
Roldán M.
Sanagustín D.
Valiente A.
INAD (Instituto de Neuropsiquiatría y Adicciones) , Psychiatry, Barcelona , Spain
* Corresponding author.
Keywords: sialorrhea, clozapine

Conflict of interest:

No

Article ID: EPV1074

Evaluation of use long acting antipsychotic in an assertive community treatment.

Garcia Gonzalez L. 1 *
Rodríguez Mercado C. 2
Willems Aguado A. 3
González Fernández A. 4
Díaz M. 5
Perez Gomez L. 6
1 Sespa, Centro De Salud Mental Eria, Oviedo, Spain
2 Sespa, Centro De Salud Mental La Calzada, Gijon, Spain
3 sespa, Centro De Salud Mental Cangas Del Narcea , Cangas del Narcea , Spain
4 sespa, Centro De Salud Mental Corredoria , oviedo, Spain
5 sespa, Hospital Valle Del Nalon , Langreo , Spain
6 Hospital San Agustin, Mental Health , Avilés , Spain
* Corresponding author.
Keywords: schizophrenia, treatment, ACT, LAIs

Conflict of interest:

No

Article ID: EPV1075

Descriptive study of clozapine in a team of severe mental disorders

Garcia Gonzalez L. 1 *
Rodríguez Mercado C.M. 2
González Fernández A. 3
Willems Aguado A. 4
Perez Gomez L. 5
1 Sespa, Centro De Salud Mental Eria, Oviedo , Spain
2 SESPA, Centro De Salud Mental La Calzada , Gijón , Spain
3 sespa, Centro De Salud Mental Corredoria , oviedo , Spain
4 sespa, Centro De Salud Mental Cangas Del Narcea , Cangas del Narcea , Spain
5 Hospital San Agustin, Mental Health , Avilés , Spain
* Corresponding author.
Keywords: ACT, severe mental disorder, clozapine, schizophrenia

Conflict of interest:

No

Article ID: EPV1077

Patterns of use and dosage of psychiatric medications in major psychiatric illnesses in taiwan

Pan Y.-J. 1 *
Yeh L.-L. 2
1 Far Eastern Memorial Hospital , Department of Psychiatry, New Taipei City , Taiwan
2 Graduate School of Humanities and Social Sciences , Dharma Drum Institute of Liberal Arts, New Taipei City , Taiwan
* Corresponding author.
Keywords: defined daily dose (DDD), antipsychotic, sedative/hypnotic agent, antidepressant

Conflict of interest:

No

Article ID: EPV1078

Long-acting injectable paliperidone palmitate plus clozapine in resistant schizophrenia patients. Series of cases study in guadalajara area.

Rodríguez López I.T. 1 *
Largo Gomez R. 1
López Vicente C. 1
Taillefer Aguanell V. 2
Pedroviejo A. 2
1 Hospital Universitario de Guadalajara , Unidad De Salud Mental, Guadalajara , Spain
2 Hospital Universitario Guadalajara , Psiquiatría, Guadalajara , Spain
* Corresponding author.
Keywords: psychopharmacology, paliperidone, treatment-resistant patients, clozapine

Conflict of interest:

No

Article ID: EPV1080

Long-term side effects of anti-depressants (a review of the literature)

Slimani G. 1 *
Gourani M.E. 2
Kisra H. 3
1 Arrazi Psychiatric University Hospital /Faculty of Medicine and Pharmacy. Mohammed V University of Rabat, Psychiatric Emergency Department, Sale, Morocco
2 Psychiatry Department,, Psychiatric Emergency Department, Ouarzazate , Morocco
3 Arrazi Psychiatric University Hospital /Faculty of Medicine and Pharmacy. Mohammed V University of Rabat, Child Psychiatric Department, Sale, Morocco
* Corresponding author.
Keywords: Antidepressive Agents, Long Term Adverse Effects, Tricyclic, Second-Generation Antidepressive Agents

Conflict of interest:

No

Article ID: EPV1081

Review of the treatment of affective symptoms in huntington disease

Willems Aguado A.I. 1 *
Garcia Gonzalez L. 2
Rodríguez Mercado C.M. 3
González Fernández A. 2
Perez Gomez L. 4
1 CSM Cangas del Narcea, Mental Health , Cangas del Narcea , Spain
2 Sespa, Centro De Salud Mental Eria , Oviedo , Spain
3 SESPA, Centro De Salud Mental La Calzada , Gijón , Spain
4 Hospital San Agustin, Mental Health , Avilés , Spain
* Corresponding author.
Keywords: treatment, affective symptoms, Huntington Disease, major depression

Conflict of interest:

No

Article ID: EPV1083

Phenothiazine-induced systemic lupus erythematosus: a case report

Affes H. 1
Chaari I. 2 *
Feki I. 2
Hammami S. 1
Masmoudi J. 2
Zeghal K. 1
Ksouda K. 1
1 Faculty of Medicine, University of Sfax , Pharmacology Department, Sfax , Tunisia
2 Hedi Chaker University Hospital , Psychiatry A Department, Sfax , Tunisia
* Corresponding author.
Keywords: lupus, Phenothiazine, drug induced injury

Conflict of interest:

No

Article ID: EPV1084

Neuropsychiatric drugs-induced liver injury: a retrospective study

Chaari I. 1 *
Affes H. 2
Omri S. 1
Smaoui N. 1
Hammami S. 2
Zeghal K. 2
Ben Thabet J. 1
Ksouda K. 2
1 Hedi Chaker University Hospital , Psychiatry C Department, Sfax , Tunisia
2 Faculty of Medicine, University of Sfax , Pharmacology Department, Sfax , Tunisia
* Corresponding author.
Keywords: Neuropsychiatric drugs, psychopharmacology, Drug-induced liver injury

Conflict of interest:

No

Article ID: EPV1086

Risperidone vs quetiapine in the treatment of behavioral and psychological symptoms of dementia

Hrnjica A. 1 *
Bise S. 1
Lokmic-Pekic I. 2
1 Psychiatric Hospital Sarajevo , Women, Sarajevo , Bosnia and Herzegovina
2 Psychiatric Hospital Sarajevo , Intensive Care, Sarajevo , Bosnia and Herzegovina
* Corresponding author.
Keywords: behavioral and psychological symptoms of dementia, Quetiapine, risperidone, aggression

Conflict of interest:

No

Article ID: EPV1087

Lorazepam-induced urinary urgency

Kaufman K. *
Chaguturu V.
Trenton A.
Aziz R.
Babalola R.
Coluccio M.
Rutgers Robert Wood Johnson Medical School , Psychiatry, New Brunswick, United States of America
* Corresponding author.
Keywords: Adverse Drug Reaction, Lorazepam, Urinary Urgency, Treatment Non-Adherence

Conflict of interest:

No

Article ID: EPV1091
Subjects: Abstract, Psychophysiology

The impact of trait anxiety and autism traits on olfactory abilities of the general population

Barros F. 1
Figueiredo C. 2
Costa A. 3
Morais S. 4
Madeira N. 4 *
Soares S. 1
1 William James Center for Research, University of Aveiro , Department of Education And Psychology, Aveiro , Portugal
2 University of Aveiro , Research Unit On Governance, Competitiveness And Public Policies, Aveiro , Portugal
3 Universidade Católica Portuguesa , Institute of Health Sciences, Lisboa , Portugal
4 Centro Hospitalar e Universitário de Coimbra , Department of Psychiatry, Coimbra , Portugal
* Corresponding author.
Keywords: Autism, autism spectrum disorders, anxiety, olfaction

Conflict of interest:

No

Article ID: EPV1095

Neurobiological effects of two physiotherapy programmes on somatic and neurophysiological manifestations show the relevance of physiotherapy in the management of children with attention-deficit/hyperactivity disorder.

Bayo-Tallón V. 1 *
Esquirol-Caussa J. 1
Pàmias Massana M. 2
Planells-Keller K. 2
Palao Vidal D. 3
1 Escoles Universitàries Gimbernat , Autonomous University of Barcelona, Cerdanyola del Vallès, Spain, Universitary Research Service Of Physical Therapy (servei Universitari De Recerca En Fisioteràpia -s.u.r.f.), Sant Cugat del Vallès, Barcelona , Spain
2 Hospital Parc Taulí , Csmij, Sabadell (Barcelona), Spain
3 Parc Tauli-University Hospital , Mental Health, Sabadell (Barcelona), Spain
* Corresponding author.
Keywords: Autonomic Nervous System, Joint Hypermobility, ADHD, Manual Therapy

Conflict of interest:

No

Article ID: EPV1096

The role of the ectorhinal cortex (area 36) in emotion regulation of patients with essential hypertension

Kovtoniuk S. 1 *
Pervichko E. 2
Vartanov A. 2
Kozlovskiy S. 2
Korsakova N. 2
Enikolopova E. 2
1 Center for Speech Pathology and Neurorehabilitation , Neurorehabilitation, Moscow , Russian Federation
2 Lomonosov Moscow State University , Faculty of Psychology, Moscow , Russian Federation
* Corresponding author.
Keywords: ectorhinal cortex, emotion regulation, area 36, Essential hypertension

Article ID: EPV1097

Hostility in coronary heart disease: relations to myocardial contractility and coronary artery patency

Nikolaeva O. 1
Nikolaev E. 2 *
Bogdanov A. 2
Hartfelder D. 3
Litvinova E. 3
1 Chuvash Republic Cardiology Clinic , Cardiosurgery Unit, Cheboksary , Russian Federation
2 Ulianov Chuvash State University , Faculty of Medicine, Cheboksary , Russian Federation
3 Ulianov Chuvash State University , Social And Clinical Psychology Department, Cheboksary , Russian Federation
* Corresponding author.
Keywords: coronary heart disease, postmyocardial infarction patients, hostility, Psychophysiology

Conflict of interest:

No

Article ID: EPV1098
Subjects: Abstract; Psychosurgery & stimulation methods (ECT, TMS, VNS, DBS)

Risks of electroconvulsive therapy in depressed patients with intracranial aneurysm

Arts M.H.L. 1 *
Petrykiv S. 2
De Jonge L. 3
1 GGZ Westelijk Noord-Brabant , Geriatric Psychiatry And Neuropsychiatry, Halsteren , Netherlands
2 GGZ WNB , Psychiatry, Halsteren , Netherlands
3 Leonardo Scientific Research Institute , Neuropsychiatry, Groningen , Netherlands
* Corresponding author.
Keywords: Dépression, ECT, aneurysm

Conflict of interest:

No

Article ID: EPV1099

The role of electroconvulsive therapy in the treatment of dementia with lewy bodies

Arts M.H.L. 1 *
Petrykiv S. 2
Michielsen P. 3
De Jonge L. 4
1 GGZ Westelijk Noord-Brabant , Geriatric Psychiatry And Neuropsychiatry, Halsteren , Netherlands
2 GGZ WNB , Psychiatry, Halsteren , Netherlands
3 GGZ-WNB, High And Intensive Care (hic) , Halsteren , Netherlands
4 Leonardo Scientific Research Institute , Neuropsychiatry, Groningen , Netherlands
* Corresponding author.
Keywords: Lewy bodies, ECT, dementia

Conflict of interest:

No

Article ID: EPV1100

Is it necessary to dismiss the ventriculo-peritoneal bypass valve malfunction before undergoing electroconvulsive therapy in patients with normal pressure hydrocephalus (NPH) neurologically asymptomatic?

Baenas I. 1 *
De Arriba-Arnau A. 2
Soria V. 2
Urretavizcaya M. 2
1 Bellvitge University Hospital , Department of Psychiatry, L'Hospitalet de Llobregat, Spain
2 Bellvitge University Hospital -IDIBELL , Psychiatry, Barcelona , Spain
* Corresponding author.
Keywords: Electroconvulsive Therapy (ECT), Normal Pressure Hydrocephalus (NPH), Ventriculo-peritoneal bypass valve

Conflict of interest:

No

Article ID: EPV1101

Use of etomidate in patients undergoing electroconvulsive therapy (ECT): effects of hypnotic change on seizure quality. A case series report.

Baenas I. 1 *
De Arriba-Arnau A. 2
González-Águila M. 1
Soria V. 2
Urretavizcaya M. 2
1 Bellvitge University Hospital , Department of Psychiatry, L'Hospitalet de Llobregat, Spain
2 Bellvitge University Hospital -IDIBELL , Psychiatry, Barcelona , Spain
* Corresponding author.
Keywords: Electroconvulsive Therapy (ECT), Etomidate

Conflict of interest:

No

Article ID: EPV1105

Cerebellar transcranial magnetic stimulation for anhedonia in depression

Kocijancic Azzaoui S. 1 *
Bon J. 2
1 Splošna bolnišnica Novo mesto , Psihiatrija , Novo mesto , Slovenia
2 University Psychiatric clinic Ljubljana , Psychiatry, Ljubljana , Slovenia
* Corresponding author.
Keywords: TMS, Cerebellum, Dépression, Anhedonia

Conflict of interest:

No

Article ID: EPV1106

Particularities of psychotic depression and its response to electroconvulsive therapy (ECT), in comparison with nonpsychotic depression

De Arriba Arnau A. 1 2 3 *
Massaneda C. 1
Martínez Fernández L. 1
Soria V. 4
Salvat N. 5
González-Águila M. 1
Menchon Magrina J. 4
Urretavizcaya M. 4
1 Hospital Universitari de Bellvitge, Psychiatry, L'Hospitalet de Llobregat , Barcelona , Spain
2 Carlos III Health Institute ,, Centro De Investigación Biomédica En Red De Salud Mental (cibersam), Barcelona , Spain
3 Bellvitge Biomedical Research Institute (IDIBELL) , Neurosciences Group - Psychiatry And Mental Health, L'Hospitalet de Llobregat, Spain
4 Bellvitge University Hospital -IDIBELL-CIBERSAM - UB, Psychiatry, Department of Clinical Sciences, School of Medicine, Barcelona, Spain
5 Bellvitge University Hospital -IDIBELL-CIBERSAM - Corporació Sanitària Parc Taulí , Psychiatry, Barcelona , Spain
* Corresponding author.
Keywords: ECT, Psychotic Depression, Dépression, Electroconvulsive Therapy

Conflict of interest:

No

Article ID: EPV1108

Bispectral index (BIS) as tool to optimize electroconvulsive therapy (ETC)

Mármol A. 1 *
Rosero Á.S. 1
Ballesteros A. 2
Uriarte Rosquil E. 1
Yoldi Murillo J. 1
López Ilundain J.M. 1
1 Red de Salud Mental - Servicio Navarro de Salud - Osasunbidea , Psychiatric Hospitalization Unit, Pamplona , Spain
2 RiojaSalud, Psychiatry, Berrioplano , Spain
* Corresponding author.
Keywords: Electroconvulsive Therapy, Bispectral EEG index, ECT, BIS

Conflict of interest:

No

Article ID: EPV1109

Benefit of bispectral index (BIS) in electroconvulsive therapy (ETC)

Mármol A. 1 *
Rosero Enríquez Á.S. 1
Ballesteros A. 2
Uriarte Rosquil E. 1
Yoldi Murillo J. 1
López Ilundain J.M. 1
1 Red de Salud Mental - Servicio Navarro de Salud - Osasunbidea , Psychiatric Hospitalization Unit, Pamplona , Spain
2 RiojaSalud, Psychiatry, Berrioplano , Spain
* Corresponding author.
Keywords: Bispectral EEG index, BIS, Electroconvulsive Therapy, ECT

Conflict of interest:

No

Article ID: EPV1111

Efficacy of bilateral continuous theta burst stimulation in treatment resistant auditory hallucinations: a single-blind, randomized, sham-controlled trial.

Purohith A. 1 *
Kumar C. 2
Mehta U. 1
1 Kasturba Medical College , Manipal , Manipal Academy of Higher Education(MAHE), Psychiatry, Manipal , India
2 NATIONAL INSTITUTE OF MENTAL HEALTH AND NEURO-SCIENCES , Psychiatry, BANGALORE , India
* Corresponding author.
Keywords: rTMS, auditory verbal hallucinations, cTBS, RCT

Conflict of interest:

No

Article ID: EPV1112

Preliminary findings on efficacy of repetitive transcranial magnetic stimulation in improving cognitive functions in patients with mild cognitive impairments

Senczyszyn A. *
Marta M.
Szcześniak D.
Kobyłko A.
Fila-Witecka K.
Beszłej J.A.
Piotrowski P.
Zimny A.
Maciaszek J.
Siwicki D.
Wieczorek T.
Rymaszewska J.
Wroclaw Medical University , Wroclaw, Poland, Department of Psychiatry, Wrocław, Poland
* Corresponding author.
Keywords: cognitive stimulation, MCI, rTMS

Conflict of interest:

No

Article ID: EPV1114
Subjects: Abstract, Psychotherapy

Effectively treating depression: study design and methodology of a naturalistic study of group cognitive behavioural therapy as ECT continuation treatment

Bönke L. *
Hartling C.
Aust S.
Bajbouj M.
Grimm S.
Charite, Psychiatry, Berlin , Germany
* Corresponding author.
Keywords: Electroconvulsive Therapy, CBASP, Dépression, Cognitive Behavioral Group Therapy

Conflict of interest:

No

Article ID: EPV1116

Psychotherapeutic group intervention on psychological risk factors for suicide. Efficacy to improve hopelessness in a pilot study.

De Santiago-Díaz A.I. *
Gómez-Ruiz E.
Artal-Simón J.
Hospital Universitario Marqués de Valdecilla , Servicio De Psiquiatría, SANTANDER, Spain
* Corresponding author.
Keywords: Suicide, suicide risk, hopelessness, Psychotherapy

Conflict of interest:

No

Article ID: EPV1118

On cognitive flexibility. Acceptance and commitment therapy versus a mindfulness-based emotional regulation intervention in anxiety disorders. A randomized controlled trial: the rem-act study

Fernández-Jiménez E. 1 *
Vidal-Bermejo E. 1
Hospital-Moreno A. 2
1 La Paz University Hospital . IdiPAZ, Department of Psychiatry, Clinical Psychology And Mental Health, Madrid , Spain
2 La Paz University Hospital , Department of Psychiatry, Clinical Psychology And Mental Health, Madrid , Spain
* Corresponding author.
Keywords: Acceptance and Commitment Therapy, Mindfulness-based Emotional Regulation, randomized controlled trial, Cognitive Flexibility

Conflict of interest:

No

Article ID: EPV1119

On the stroop effect. Acceptance and commitment therapy versus a mindfulness-based emotional regulation intervention in anxiety disorders. A randomized controlled trial: the rem-act study

Fernández-Jiménez E. 1 *
Vidal-Bermejo E. 1
Hospital-Moreno A. 2
1 La Paz University Hospital. IdiPAZ , Department of Psychiatry, Clinical Psychology And Mental Health, Madrid , Spain
2 La Paz University Hospital , Department of Psychiatry, Clinical Psychology And Mental Health, Madrid , Spain
* Corresponding author.
Keywords: Stroop effect, Acceptance and Commitment Therapy, Mindfulness-based Emotional Regulation, randomized controlled trial

Conflict of interest:

No

Article ID: EPV1120

On attentional function. Acceptance and commitment therapy versus a mindfulness-based emotional regulation intervention in anxiety disorders. A randomized controlled trial: the rem-act study

Fernández-Jiménez E. 1 *
Vidal-Bermejo E. 1
Hospital-Moreno A. 2
1 La Paz University Hospital. IdiPAZ , Department of Psychiatry, Clinical Psychology And Mental Health, Madrid , Spain
2 La Paz University Hospital , Department of Psychiatry, Clinical Psychology And Mental Health, Madrid , Spain
* Corresponding author.
Keywords: randomized controlled trial, attention, Acceptance and Commitment Therapy, Mindfulness-based Emotional Regulation

Conflict of interest:

No

Article ID: EPV1121

On auditive working memory. Acceptance and commitment therapy versus a mindfulness-based emotional regulation intervention in anxiety disorders. A randomized controlled trial: the rem-act study

Fernández-Jiménez E. 1 *
Vidal-Bermejo E. 1
Hospital-Moreno A. 2
1 La Paz University Hospital. IdiPAZ , Department of Psychiatry, Clinical Psychology And Mental Health, Madrid , Spain
2 La Paz University Hospital , Department of Psychiatry, Clinical Psychology And Mental Health, Madrid , Spain
* Corresponding author.
Keywords: auditive working memory, Acceptance and Commitment Therapy, Mindfulness-based Emotional Regulation, randomized controlled trial

Conflict of interest:

No

Article ID: EPV1122

On anxiety sensitivity. Acceptance and commitment therapy versus a mindfulness-based emotional regulation intervention in anxiety disorders. A randomized controlled trial: the rem-act study

Vidal-Bermejo E. 1
Fernández-Jiménez E. 1 *
Hospital-Moreno A. 2
1 La Paz University Hospital. IdiPAZ , Department of Psychiatry, Clinical Psychology And Mental Health, Madrid , Spain
2 La Paz University Hospital , Department of Psychiatry, Clinical Psychology And Mental Health, Madrid , Spain
* Corresponding author.
Keywords: Acceptance and Commitment Therapy, Mindfulness-based Emotional Regulation, randomized controlled trial, anxiety sensitivity

Conflict of interest:

No

Article ID: EPV1125

Psychotherapeutic approaches in emergencies during the acute period

Kuzmina T. *
Zakharova N.
Baeva A.
Tsvetkova M.
National Medical Research Centre of Psychiatry and Addiction n.a. V.P. Serbsky Moscow , Russia, Department of Psychiatry And Psychotherapy Assistance To Victims Of Emergency Situations, Moscow , Russian Federation
* Corresponding author.
Keywords: MEDICAL AND PSYCHOLOGICAL ASSISTANCE, VICTIMS OF EMERGENCIES, EMERGENCY PSYCHOTHERAPY, EMERGENCY SITUATION

Conflict of interest:

No

Article ID: EPV1126

On mindfulness trait. Acceptance and commitment therapy versus a mindfulness-based emotional regulation intervention in anxiety disorders. A randomized controlled trial: the rem-act study

Vidal-Bermejo E. 1
Fernández-Jiménez E. 1
Louzao-Rojas I.I. 1 *
Hospital-Moreno A. 2
1 La Paz University Hospital. IdiPAZ , Department of Psychiatry, Clinical Psychology And Mental Health, Madrid , Spain
2 La Paz University Hospital , Department of Psychiatry, Clinical Psychology And Mental Health, Madrid , Spain
* Corresponding author.
Keywords: Acceptance and Commitment Therapy, Mindfulness-based Emotional Regulation, randomized controlled trial, mindfulness trait

Conflict of interest:

No

Article ID: EPV1127

On experiential avoidance. Acceptance and commitment therapy versus a mindfulness-based emotional regulation intervention in anxiety disorders. A randomized controlled trial: the rem-act study

Vidal-Bermejo E. 1
Fernández-Jiménez E. 1
Louzao-Rojas I.I. 1 *
Hospital-Moreno A. 2
1 La Paz University Hospital. IdiPAZ , Department of Psychiatry, Clinical Psychology And Mental Health, Madrid , Spain
2 La Paz University Hospital , Department of Psychiatry, Clinical Psychology And Mental Health, Madrid , Spain
* Corresponding author.
Keywords: Acceptance and Commitment Therapy, Mindfulness-based Emotional Regulation, randomized controlled trial, experiential avoidance

Conflict of interest:

No

Article ID: EPV1129

Gender, oedipus, and the psychotic rejection of lack and femininity

Mitropoulos G. 1 *
Gorgoli D. 2
1 Psychiatric Hospital of Attica , 5th Department, Athens , Greece
2 Constantopouleio General Hospital of Nea Ionia , Psychiatric Department, Athens , Greece
* Corresponding author.
Keywords: gender, oedipus, psychosis, lack

Conflict of interest:

No

Article ID: EPV1131

Modalities of play in children’s interventive psychodiagnosis registered through an electronic form in a university clinical practice in brazil

Varanda C. *
Campos M.
Poppe A.
Universidade Paulista , Instituto De Ciências Humanas, Santos , Brazil
* Corresponding author.
Keywords: assessment, psychotherapy, intervention, children

Conflict of interest:

No

Article ID: EPV1132

Dynamics of attitudes to concomitant pharmacotherapy in patients with neurotic disorders in the process of group-analytical psychotherapy

Belokrylov I. *
Munin A.
Brukhin A.
Lineva T.
Semikov S.
Peoples' Friendship University of Russia (RUDN University), Department of Psychiatry And Medical Psychology, Moscow , Russian Federation
* Corresponding author.
Keywords: concomitant psychopharmacotherapy, dynamics of pharmacological effects, group analysis

Article ID: EPV1133

Psychotherapy of somatoform disorders developing in patients with schizotypal personality disorder.

Belokrylov I. *
Semikov S.
Brukhin A.
Munin A.
Bayarsaikhan B.
Peoples' Friendship University of Russia (RUDN University), Department of Psychiatry And Medical Psychology, Moscow , Russian Federation
* Corresponding author.
Keywords: schizotypal personality disorder., psychotherapy, somatoform disorders

Article ID: EPV1134

Implementation of low-intense group psychotherapy approach within psychiatric ward in ukraine

Lahutina S.
Bogomolets National Medical University , Institute of Cognitive Modeling, Medical Psychology, Psychosomatic Medicine and Psychotherapy Department, Kyiv , Ukraine
Keywords: group psychotherapy, approach, Effectiveness, psychiatric ward

Conflict of interest:

No

Article ID: EPV1135

Applications of acceptance and commitment therapy for group treatment

León-Quismondo L. 1 *
López Ríos F. 2
Fernández Liria A. 3
Lahera G. 4
1 Ramon y Cajal University Hospital , Psychiatry, Madrid , Spain
2 University of Almería , Psychology, Almería , Spain
3 Hospital Universitario Príncipe de Asturias , Psychiatry, Alcalá de Henares , Spain
4 University of Alcala , School of Medicine, Alcalá de Henares , Spain
* Corresponding author.
Keywords: Acceptance and Commitment Therapy, Group treatment, Applications

Conflict of interest:

No

Article ID: EPV1136

Reducing the risk of socially dangerous behavior in people with mental disorders based on comprehensive psychotherapeutic correction and rehabilitation

Makushkina O.
Federal State Budgetary Institution “V.P. Serbsky National Medical Research Centre for Psychiatry and Narcology” of the Ministry of Health of the Russian Federation , Department of Forensic Psychiatric Prevention, Moscow , Russian Federation
Keywords: psychotherapeutic correction, Prevention, socially dangerous behavior, reducing risk

Conflict of interest:

No

Article ID: EPV1137

Severe hypertensive crisis in adolescents treated with atomoxetine and nasal decongestants. clinical case.

Pérez Sánchez S. 1 *
Martín Herrero I. 1
Crespo Portero A. 2
Güimil Raya D. 1
Cassinello Marco M. 1
1 Morales Meseguer Public University Hospital , Psychiatry, Murcia , Spain
2 Lorca Mental health Center , Psychiatry, Lorca , Spain
* Corresponding author.
Keywords: Atomoxetine, adolescence, pseudoephedrine, decongestants.

Conflict of interest:

No

Article ID: EPV1138

Adverse effects related to prolactin in adolescents with antipsychotics.

Pérez Sánchez S. 1 *
Martín Herrero I. 1
Crespo Portero A. 2
Güimil Raya D. 1
Cassinello Marco M. 1
1 Morales Meseguer Public University Hospital , Psychiatry, Murcia , Spain
2 Lorca Mental health Center , Psychiatry, Lorca , Spain
* Corresponding author.
Keywords: adolescents, antipsychotics, hyperprolactinemia

Conflict of interest:

No

Article ID: EPV1139

Psychosocial adjustment in breast cancer patients: adaptivity, coping and self-regulation

Petunova S. 1 *
Lazareva E. 1
Maksimova N. 1
Litvinova E. 1
Petunova Y. 2
1 Ulianov Chuvash State University , Social And Clinical Psychology Department, Cheboksary , Russian Federation
2 I.M. Sechenov First Moscow State Medical University , Faculty of Medicine, Moscow , Russian Federation
* Corresponding author.
Keywords: self-regulation, breast cancer patients, psychosocial adjustment, Coping

Conflict of interest:

No

Article ID: EPV1140

Cognitive behavior therapy for trichotillomania: case report for a “secret” disorder

Lahmar S.
Qatar University , Student Counseling Center, doha , Qatar
Keywords: cognitive behavior Therapy, Culture, trichotillomania, social life

Conflict of interest:

No

Article ID: EPV1141

Auditive working memory 6 months after two mindfulness-based group interventions: acceptance and commitment therapy and a mindfulness-based emotional regulation intervention in anxiety disorders. preliminary results

Fernández-Jiménez E. 1
Vidal-Bermejo E. 1
Louzao-Rojas I.I. 1 *
Hospital-Moreno A. 2
1 La Paz University Hospital. IdiPAZ , Department of Psychiatry, Clinical Psychology And Mental Health , Madrid , Spain
2 La Paz University Hospital , Department of Psychiatry, Clinical Psychology And Mental Health , Madrid , Spain
* Corresponding author.
Keywords: mindfulness-based interventions, randomized controlled trial, follow-up, auditive working memory

Conflict of interest:

No

Article ID: EPV1142

Relationship between attention self-reported and objectively measured in adult patients with anxiety disorders referred to mindfulness-based group interventions

Vidal-Bermejo E. 1
Fernández-Jiménez E. 1
Louzao-Rojas I.I. 1 *
Hospital-Moreno A. 2
1 La Paz University Hospital. IdiPAZ , Department of Psychiatry, Clinical Psychology And Mental Health , Madrid , Spain
2 La Paz University Hospital , Department of Psychiatry, Clinical Psychology And Mental Health , Madrid , Spain
* Corresponding author.
Keywords: mindfulness-based interventions, self-reported cognition, objective neuropsychological test, attention

Conflict of interest:

No

Article ID: EPV1143

Cognitive behavioral therapy approach in affective disorders patients

Mykhaylov B. *
Kudinova O.
Kharkiv Medical Academy Postgraduate Education , Psychotherapy, Kharkiv , Ukraine
* Corresponding author.
Keywords: Cognitive behavioral therapy, affective disorders, panic disorder

Conflict of interest:

No

Article ID: EPV1144

On alexithymia. acceptance and commitment therapy versus a mindfulness-based emotional regulation intervention in anxiety disorders. a randomized controlled trial: the rem-act study

Fernández-Jiménez E. 1
Vidal-Bermejo E. 1
Peñalver-Alonso M. 1 *
Hospital-Moreno A. 2
1 La Paz University Hospital. IdiPAZ , Department of Psychiatry, Clinical Psychology And Mental Health , Madrid , Spain
2 La Paz University Hospital , Department of Psychiatry, Clinical Psychology And Mental Health , Madrid , Spain
* Corresponding author.
Keywords: Acceptance and Commitment Therapy, Mindfulness-based Emotional Regulation, randomized controlled trial, Alexithymia

Conflict of interest:

No

Article ID: EPV1145

Cognitive flexibility 6 months after two mindfulness-based group interventions: acceptance and commitment therapy and a mindfulness-based emotional regulation intervention in anxiety disorders. preliminary results

Fernández-Jiménez E.
Vidal-Bermejo E.
Peñalver-Alonso M. *
Hospital-Moreno A.
La Paz University Hospital. IdiPAZ , Department of Psychiatry, Clinical Psychology And Mental Health , Madrid , Spain
* Corresponding author.
Keywords: mindfulness-based interventions, follow-up, Cognitive Flexibility, randomized controlled trial

Conflict of interest:

No

Article ID: EPV1146

Stroop effect 6 months after two mindfulness-based group interventions: acceptance and commitment therapy and a mindfulness-based emotional regulation intervention in anxiety disorders. preliminary results

Fernández-Jiménez E. 1
Vidal-Bermejo E. 1
Peñalver-Alonso M. 1 *
Hospital-Moreno A. 2
1 La Paz University Hospital. IdiPAZ , Department of Psychiatry, Clinical Psychology And Mental Health , Madrid , Spain
2 La Paz University Hospital , Department of Psychiatry, Clinical Psychology And Mental Health , Madrid , Spain
* Corresponding author.
Keywords: mindfulness-based interventions, randomized controlled trial, follow-up, Stroop effect

Conflict of interest:

No

Article ID: EPV1147

Attentional function 6 months after two mindfulness-based group interventions: acceptance and commitment therapy and a mindfulness-based emotional regulation intervention in anxiety disorders. preliminary results

Fernández-Jiménez E. 1
Vidal-Bermejo E. 1
Peñalver-Alonso M. 1 *
Hospital-Moreno A. 2
1 La Paz University Hospital. IdiPAZ , Department of Psychiatry, Clinical Psychology And Mental Health , Madrid , Spain
2 La Paz University Hospital , Department of Psychiatry, Clinical Psychology And Mental Health , Madrid , Spain
* Corresponding author.
Keywords: mindfulness-based interventions, randomized controlled trial, follow-up, attention

Conflict of interest:

No

Article ID: EPV1148

Psychological therapies targeting negative symptoms in schizophrenia: a systematic review

Petrescu M. *
Tudor C.
Vasile D.
"Dr. Carol Davila" Central Military Emergency University Hospital , Department of Psychiatry, Bucharest , Romania
* Corresponding author.
Keywords: negative symptoms, psychotherapy, psychological intervention, schizophrenia

Conflict of interest:

No

Article ID: EPV1150

An introduction to the concept of sensory and genetic memory codes in mental health

Sluiter S.
Susan Sluiter Clinical Psychologist , Psychology, South , South Africa
Keywords: who is susan sluiter, Trauma Looking Through the Trauma Lens

Conflict of interest:

No

Article ID: EPV1152

Gratification disorder – a case report in the light of transgenerational family therapy

Almeida B. 1 *
Magalhães P. 2
Pereira M.J. 2
1 Hospital de Magalhães Lemos , Psychiatry, Porto , Portugal
2 Centro Materno-Infantil do Norte, Centro Hospitalar e Universitário do Porto , Child And Adolescent Psychiatry, Porto , Portugal
* Corresponding author.
Keywords: family therapy, sexual abuse, Transgenerational, Gratification Disorder

Conflict of interest:

No

Article ID: EPV1153

Experience in the psychotherapy unit (ufip) of la paz university hospital.

Navarro Morejón L. 1
Piña Baena A. 2
Martinez Parreño E. 3
Torrijos M. 4
Rocamora González C. 5
Palao Á. 6 *
1 Hospital Universitario de Canarias , Psychiatry, La Laguna , Spain
2 Hospital Universitario Virgen Macarena , Psychiatry, Sevilla , Spain
3 Complejo Hospitalario de Navarra , Psychiatry, Pamplona , Spain
4 Psychiatry and Mental Health Department La Paz Hospital ., Psychiatry, MADRID , Spain
5 Hospital Universitario La Paz , Psiquiatria, Madrid , Spain
6 HOSPITAL UNIVERSITARIO LA PAZ , Psiquiatria , MADRID , Spain
* Corresponding author.
Keywords: Psychotherapy, Oncology, Psychooncology, UFIP

Conflict of interest:

No

Article ID: EPV1155
Subjects: Abstract, Quality Management

Analysis of a hospital discharge questionnaire in a short psychiatric hospitalization.

Herrera Imbroda J. 1 *
Santiago González S. 1
Ruiz Rodríguez F.I. 2
1 Hospital Regional Universitario Málaga, Mental Health, Plaza del Hospital Civil , s/n , Spain
2 Hospital Universitario Virgen de la Victoria , Pharmacy, Málaga , Spain
* Corresponding author.
Keywords: quality satisfaction

Conflict of interest:

No

Article ID: EPV1157

Compliance with renal function monitoring in patients on lithium and its influence on the clinical decision-making process at an adult community mental health team

Sacristan Alonso E. 1 *
Dabup E. 2
Corr E. 2
Sheridan R. 2
Mcenterggart T. 2
Cullivan R. 2
1 HAMAD MEDICAL CORPORATION , Psychiatry, DOHA , Qatar
2 HSE, Cavan-monaghan Mental Health Services , Dublin , Ireland
* Corresponding author.
Keywords: lithium, Guideline, eGFR, monitoring

Conflict of interest:

No

Article ID: EPV1158

Changes in patient satisfaction questionnaire responses following the modification of the program delivery to group-based didactic treatment approach.

Samokhvalov A. 1 *
Csuzdi N. 2
Costello J. 3
Furlong B. 4
Sousa S. 3
Mackillop J. 5
1 Homewood Health Centre, Comprehensive Psychiatric Care , Guelph , Canada
2 Homewood Health Centre , Quality Management And Outcomes, Guelph , Canada
3 Homewood Research Institute, Evaluation General , Guelph , Canada
4 Homewood Health Centre, Chief Of Staff , Guelph , Canada
5 McMaster University , Department of Psychiatry, Hamilton , Canada
* Corresponding author.
Keywords: Patient Satisfaction Questionnaire, Quality Improvement, Program Change

Conflict of interest:

No

Article ID: EPV1159

Interest of the biological assessment of entry into the screening of comorbidities

Ben Hamadi A. 1 *
Ben Ammar H. 1
Hamdoun J. 1
Khelifa E. 2
Aissa A. 3
Zouhaier E.H. 3
1 Razi hospital , Psychiatry F, Manouba , Tunisia
2 University of Tunis El Manar- Faculty of medecine of Tunis, Psychiatry - Razi Hospital , Tunis , Tunisia
3 Razi Hospital , F Department, Tunis , Tunisia
* Corresponding author.
Keywords: biological assessment, comorbidities

Conflict of interest:

No

Article ID: EPV1160
Subjects: Abstract, Rehabilitation and Psychoeducation

Multidisciplinary approach for patient with aphasia: combined speech- language therapy, psychological, and sociological treatments forward progression of self- management, self-efficacy and motivation. a case study

Ahmed T. *
Private, Speech-language Pathology& Psychology, Salmiya, Kuwait
* Corresponding Author:
Keywords: Speech, Language therapy, Psychology, Self-management & Efficacy, Motivation

Conflict of interest:

No

Article ID: EPV1166

The model of psychosocial and psychotherapeutic care to relatives of the mentally ill

Solokhina T.
FSBSI Mental Health Research Center , Moscow, Russia, Department of Mental Health Services, Moscow, Russian Federation
Keywords: schizophrenia, Effectiveness, relatives, care

Conflict of interest:

No

Article ID: EPV1167

Pilot study about the efficacy of the ensemble (together) program: a tailored intervention for informal caregivers of people with severe psychiatric disorders

Wilquin H. 1 *
Plessis L. 1
Rexhaj S. 2
1 Aix-Marseille Université - Maison de la Recherche , Laboratoire LPCPP EA 3278 , Bureau 1.43, Clinical Psychology, Aix-en-Provence , France
2 Institut et Haute Ecole de la Santé , La Source, Nursing School, Lausanne, Vaud , Switzerland
* Corresponding author.
Keywords: Informal caregivers, Tailored intervention, Randomized controlled trials, psychiatric disorders

Conflict of interest:

No

Article ID: EPV1168

Severe neurotrauma in children: a typology of mental disorders in minimal consciousness

Zakrepina A. 1 *
Sidneva Y. 2
1 Clinical and Research Institute of Emergency Pediatric Surgery and Trauma (CRIEPST), Institute of Special Education of the Russian Academy of Education , Moscow, Russia, Department of Recovery Treatment And Rehabilitation, Moscow, Russian Federation
2 Clinical and Research Institute of Emergency Pediatric Surgery and Trauma (CRIEPST), Department of Recovery Treatment And Rehabilitation, Moscow , Russian Federation
* Corresponding author.
Keywords: mental activity, children rehabilitation, severe brain injuries, minimal consciousness

Conflict of interest:

No

Article ID: EPV1169

Educational approach for children with intellectual disorders

Strebeleva E.
Zakrepina A. *
Kinach E.
Butusova T.
Institute of Special Education of the Russian Academy of Education , Laboratory For The Education And Training Of Children With Mental Retardation, Moscow , Russian Federation
* Corresponding author.
Keywords: remedial education, intellectual impairment, cognitive activity, mentally disabled children

Conflict of interest:

No

Article ID: EPV1170

Self autonomy in preschool children with intellectual deficiency

Butusova T.
Zakrepina A. *
Institute of Special Education of the Russian Academy of Education , Laboratory For The Education And Training Of Children With Mental Retardation, Moscow , Russian Federation
* Corresponding author.
Keywords: preschool children with intellectual disabilities, children self autonomy, preschool education, playing skills

Conflict of interest:

No

Article ID: EPV1172

Patient satisfaction in a mental health psychosocial rehabilitation center. a descriptive analysis.

Carracedo Sanchidrián D. *
Hospital Universitario la Paz, Mental Health , Madrid , Spain
* Corresponding Author:
Keywords: mental health, rehabilitation, Chronic Mental Disorder

Conflict of interest:

No

Article ID: EPV1175

Graphic skills of children with cognitive dysfunction

Kinach E. 1
Sidneva Y. 2 *
1 Institute of Special Education of the Russian Academy of Education , Laboratory For The Education And Training Of Children With Mental Retardation, Moscow , Russian Federation
2 Clinical and Research Institute of Emergency Pediatric Surgery and Trauma (CRIEPST), Department of Recovery Treatment And Rehabilitation, Moscow , Russian Federation
* Corresponding author.
Keywords: graphic skills, graphics of writing, children with cognitive dysfunction, cognitive dysfunctions

Conflict of interest:

No

Article ID: EPV1176

Knowledge of disorders by counseling patients in psychiatry

Falfel D.
Mongi Slim hospital, Mental Health , Tunis , Tunisia
Keywords: psychoeducation, disorder, Psychiatry, Stigma

Conflict of interest:

No

Article ID: EPV1177

Add escape: an immersive experience for add treatment

Figueroa P. 1 *
Cabas-Hoyos K. 2
Gomez-Cubillos V. 3
1 Universidad de los Andes , Systems And Computing Engineering, Bogotá , Colombia
2 Universidad del Magdalena , Magdalena, Santa Marta , Colombia
3 Universidad de los Andes , Systems And Computing Engineering, Bogota , Colombia
* Corresponding author.
Keywords: virtual reality, ADD, ADD Intervention, serious games

Conflict of interest:

No

Article ID: EPV1178

Assessment of lithium adherence in bipolar patients: the experience in an italian psychiatric unit

Franchini L. 1
Smail D. 2 *
Tonet L. 2
Seghi F. 2
Causarano B. 1
Varacalli C. 1
Barbini B. 1
Colombo C. 2
1 San Raffaele Hospital, Mood Disorder Unit , Milan , Italy
2 Università Vita-Salute, San Raffaele Hospital , Milan , Italy
* Corresponding author.
Keywords: Bipolar, medication adherence, lithium knowledge, lithium attitude

Conflict of interest:

No

Article ID: EPV1179

The choice of rehabilitation and educational route for children with neurotrauma.

Bratkova M. 1 2 *
Zakrepina A. 3 4
1 Moscow City University , Department of Clinical Psychology And The Basics Of Defectology, Moscow , Russian Federation
2 Clinical and Research Institute of Emergency Pediatric Surgery and Trauma (CRIEPST), Moscow, Russia, Department of Recovery Treatment And Rehabilitation, Moscow, Russian Federation
3 Institute of Special Education of the Russian Academy of Education / ISE RAO , Laboratory of Psychological And Pedagogical Research And Technologies For Special Education Of Persons With Intellectual Disabilities, Moscow, Russia, Moscow, Russian Federation
4 Clinical and Research Institute of Emergency Pediatric Surgery and Trauma (CRIEPST), Department of Recovery Treatment And Rehabilitation, Moscow , Russian Federation
* Corresponding author.
Keywords: educational route, Integration, neurotrauma, rehabilitation of children

Conflict of interest:

No

Article ID: EPV1181

Dynamic characteristics of higher mental functions in children 7-9 years old following traumatic brain injury of mild severity

Kovtoniuk S. 1 *
Goriatcheva T. 2
1 Center for Speech Pathology and Neurorehabilitation, Neurorehabilitation, Moscow , Russian Federation
2 Russian national research medical University N. I. Pirogov , Psychology, Moscow , Russian Federation
* Corresponding author.
Keywords: higher mental functions, Children, mTBI, dynamic characteristics

Conflict of interest:

No

Article ID: EPV1182

Speech pathology research in adolescents following traumatic brain injury of mild severity.

Kovtoniuk S. 1 *
Goriatcheva T. 2
1 Center for Speech Pathology and Neurorehabilitation, Neurorehabilitation, Moscow , Russian Federation
2 Russian national research medical University N. I. Pirogov , Psychology, Moscow , Russian Federation
* Corresponding author.
Keywords: adolescents, Speech pathology, traumatic brain injury, mTBI

Conflict of interest:

No

Article ID: EPV1184
Subjects: Abstract, Research Methodology

Internal consistency and confirmatory factor analysis of the scale of attitude towards christianity (francis-5) in colombian psychiatric outpatients

Campo-Arias A. 1 *
Herazo E. 2
Ceballos-Ospino G. 1
1 University of Magdalena , Faculty of Health Sciences, Santa Marta, Colombia
2 Human Behavioral Research Institute , Direction, Bogota , Colombia
* Corresponding author.
Keywords: Validation studies, religiosity, mental disorders, Outpatients

Conflict of interest:

No

Article ID: EPV1186

Content overlap analysis of 71 symptoms among seven most commonly used neurological soft signs scales.

Krupa A. 1 *
Chrobak A. 2
Dudek D. 2
Siwek M. 3
1 Jagiellonian University Medical College , Doctoral School of Medical And Health Sciences, Cracow , Poland
2 Jagiellonian University Medical College , Department of Adult Psychiatry, Cracow , Poland
3 Jagiellonian University Medical College , Department of Affective Disorders, Cracow , Poland
* Corresponding author.
Keywords: Content analysis, neurological soft signs

Conflict of interest:

No

Article ID: EPV1189

Interest for the innovative clinical-qualitative method grows among brazilian health researchers

Turato E.
University of Campinas , Medical Psychology And Psychiatry, Campinas , Brazil
Keywords: Qualitative Research, clinical-qualitative research in Brazil, psychological meanings

Conflict of interest:

No

Article ID: EPV1190

Placebo response in clinical trials- how to decrease its negative impact?

Vasile D. *
Vasiliu O.
Fainarea A.
Patrascu M.
Morariu E.
Stanescu R.
Manolache R.
Alexandru I.
Ghenoiu I.
Gionea M.
Gainaru F.
Amanolesei I.
Vlaicu R.
University Emergency Central Military Hospital Dr. Carol Davila , Psychiatry, Bucharest , Romania
* Corresponding author.
Keywords: placebo response, placebo effect, major depression, Antidepressants

Article ID: EPV1191

Video ethnography: introducing a new tool for psychiatric research ?

Abrahamyan Empson L. *
Conus P.
CHUV , Psychiatry Department, Prilly , Switzerland
* Corresponding author.
Keywords: videoethnography, Mixed methods, urbanicity, psychosis

Conflict of interest:

No

Article ID: EPV1192

Psychometric evaluation of the caffeine expectancy questionnaire (caffeq) in spanish speakers

Cebrián J. 1 *
Gonzalez-Cuevas G. 1 2
1 European University of Madrid , Department of Psychology, Madrid , Spain
2 Idaho State University , Department of Biomedical And Pharmaceutical Sciences, Meridian , United States of America
* Corresponding author.
Keywords: Caffeine, CAffEQ, reliability, validity

Conflict of interest:

No

Article ID: EPV1193

Optical manipulation of prefrontal parvalbumin neurons disrupts attention in mice

Dexter T.
Western University , Schulich Medicine and Dentistry, London , Canada
Keywords: Prefrontal Cortex, attention, Parvalbumin Neurons

Conflict of interest:

No

Article ID: EPV1194

Contributions of projective techniques as potent tools of therapeutic intervention in the psychodiagnosis process.

Poppe A.
Universidade Paulista, Instituto De Ciências Humanas , Santos , Brazil
Keywords: Projective techniques, Psychodiagnosis, Evaluation, Therapeutic intervention

Conflict of interest:

No

Article ID: EPV1195

The polish short form of the purpose in life test (pil-6): psychometric properties for patients with mental disorders

Zycinska J. 1 *
Januszek M. 1
Dobaczewska P. 2
1 SWPS University of Social Sciences and Humanities , Katowice Faculty of Psychology, Katowice, Poland
2 SWPS University of Social Sciences and Humanities , Interdisciplinary Doctoral School, Warsaw , Poland
* Corresponding author.
Keywords: purpose in life, Confirmatory Factor Analysis, serious mental illness

Conflict of interest:

No

Article ID: EPV1199
Subjects: Abstract, Schizophrenia and Other Psychotic Disorders

A comparative study regarding emotional awareness and interpersonal relating abilities in the schizophrenia versus the affective spectra

Adam I.R. 1 *
Oprea C.V. 1
Culici A. 1
Bredicean C. 2
Giurgi Oncu C. 2
1 Timișoara County Emergency Clinical Hospital, Clinic Of Psychiatry , ,eduard Pamfil" , Timișoara , Romania
2 "Victor Babeș” University of Medicine and Pharmacy , Neuroscience , Timișoara , Romania
* Corresponding author.
Keywords: Social Cognition, recurrent depressive disorder, Alexithymia, Schizophrenia Spectrum Disorder

Conflict of interest:

No

Article ID: EPV1204

Relationships between suicide risk and neuropsychological domains in schizophrenia patients

Bargagna P. *
Corigliano V.
Forcina F.
Nardella A.
Mancinelli I.
Falcone G.
Pompili M.
Comparelli A.
Sapienza University - Faculty of Medicine and Psychology, Neurosciences, Mental Health And Sensory Organs ., Rome , Italy
* Corresponding author.
Keywords: Suicide, schizophrenia, neurocognitive deficit, Social Cognition

Conflict of interest:

No

Article ID: EPV1206

Depression in remitted schizophrenia patients

Bhagyalakshmi Nanjayya S. *
Grover S.
PGIMER , Department of Psychiatry, Chandigarh , India
* Corresponding author.
Keywords: Dépression; schizophrenia,; remission; chronic

Conflict of interest:

No

Article ID: EPV1207

First episode psychosis in cannabis users: primary psychotic disorder or cannabis induced psychotic disorder? case-control study: preliminary results about cognitive impairment in first-episode psychosis.

Boi S. 1 *
Sanz-Aranguez Avila B. 1
González-Salvador T. 1
Suarez Del Rio E.M. 1
Ponte-López T. 2
Lobato M.J. 2
De Arce Cordón R. 1
1 Hospital Universitario Puerta de Hierro de Majadhonda , Psychiatry, Majadahonda , Spain
2 Hospital Universitario Puerta de Hierro , Majadahonda, Madrid , Spain
* Corresponding author.
Keywords: first-episode psychosis, SCIP-S First Episode Psychosis, COGNITIVE IMPAIRMENT IN FIRST-EPISODE PSYCHOSIS, cannabis users

Conflict of interest:

No

Article ID: EPV1208

Comparison of polytherapy versus monotherapy in patients with treatment-resistant schizophrenia: a review.

Bravo Arráez M. 1 *
Martín Alvarez C. 2
Mainar De Paz V. 3
1 Hospital Severo Ochoa , Psiquiatría, Madrid , Spain
2 Hospital Universitario de Fuenlabrada , Psiquiatría, Madrid , Spain
3 Centro de Salud Mental Maresme Nord , Psiquiatría, Barcelona , Spain
* Corresponding author.
Keywords: schizophrenia, psychopharmacology, Polypharmacy

Conflict of interest:

No

Article ID: EPV1209

The connection between the theory of mind and alexithymia in schizoaffective disorder

Bredicean C. 1 *
Papava I. 2
Moldovan R.T. 3
Giurgi Oncu C.I. 1
Popovici Z. 4
Rivis I. 5
Grujic D. 6
1 "Victor Babeș” University of Medicine and Pharmacy , Neuroscience , Timișoara , Romania
2 Timisoara Univeristy of Medicine and Pharmacy, Psychiatric Clinic , Neurosciences, Timisoara , Romania
3 Timișoara County Emergency Hospital , Clinic Of Psychiatry , ,eduard Pamfil” , Timisoara , Romania
4 Arad County Emergency Clinical Hospital, Centre For Mental Health , Arad , Romania
5 “Carol Davila” University of Medicine and Pharmacy Bucharest , Psychiatry, bucuresti, Romania
6 “ Victor Babeș” University of Medicine and Pharmacy Timișoara , Plastic And Reconstructive Surgery, timisoara , Romania
* Corresponding author.

Conflict of interest:

No

Article ID: EPV1210

Victimization in schizophrenia

Budak M. 1 *
Gulec H. 2
Ustundag M. 2
1 Erenkoy Mental Health Training and Education Hospital, Male Inpatient Clinic , Istanbul , Turkey
2 Erenkoy Mental Health Training and Education Hospital , Psychiatry, Istanbul , Turkey
* Corresponding author.
Keywords: schizophrenia, parry fracture, violence, victimization

Conflict of interest:

No

Article ID: EPV1212

Paranoid, steroids – a case report of an organic delusional disorder secondary to systemic lupus erythematosus

Carneiro M. 1 *
Pereira D. 1
Neves S. 2
1 Centro Hospitalar e Universitário de Coimbra , Psychiatry, Coimbra , Portugal
2 Centro Hospitalar e Universitário de Coimbra , Psychiatry, Coimbra , Portugal
* Corresponding author.
Keywords: organic delusional disorder, Systemic Lupus Erythematosus, corticosteroids

Conflict of interest:

No

Article ID: EPV1215

Psychotic symptoms of onset in childhood. a clinical case

Cozar Ortiz J. *
Rubio Zavala I.
Suarez Guinea R.
Hernández Caro C.M.
Plaza Yuste M.
Torrente Seoane C.
Rodríguez Delgado C.
Torres Varona F.J.
Ainslie Mata Y.
Faber D.
Benavente López S.
Juárez Calvo V.
Rodríguez Villarino M.C.
Iglesias García C.
Pérez-Iñigo Gancedo J.L.
Presa García M.
Hospital Central de la Defensa Gómez Ulla, Psychiatry And Mental Health Department , Madrid , Spain
* Corresponding author.
Keywords: Psychotic, childhood onset, aripiprazole, olfactory hallucinations

Conflict of interest:

No

Article ID: EPV1216

A 40-years old man with first schizophrenic episode: a case report

Curk Fišer N. *
Tavčar R.
University psychiatric clinic Ljubljana, Unit For Rehabilitation , Ljubljana , Slovenia
* Corresponding author.
Keywords: symptomatology, schizophrenia, shizoaffective disorder, misdiagnosis

Conflict of interest:

No

Article ID: EPV1217

A 40-years old man with first schizophrenic episode: a case report

Curk Fišer N. *
Tavčar R.
University psychiatric clinic Ljubljana, Unit For Rehabilitation , Ljubljana , Slovenia
* Corresponding author.
Keywords: symptomatology, schizophrenia, shizoaffective disorder, misdiagnosis

Conflict of interest:

No

Article ID: EPV1218

Management of late onset schizophrenia and delusions of influence: a patient with no awareness of disease

De Benito Cháfer A.O. *
Ibáñez Martínez E.
Hospital Universitario de Fuenlabrada , Psiquiatría, Fuenlabrada , Spain
* Corresponding author.
Keywords: paranoid schizophrenia, late onset schizophrenia, delusions of influence, no awareness of disease

Conflict of interest:

No

Article ID: EPV1219

First approach to delusional parasitosis: how a delayed and unproper diagnosis can influence a person’s wellbeing

De Benito Cháfer A.O. *
Martín Álvarez C.
Martín Galeote S.
Ventura Tejerina B.
Rodríguez Soria E.
Nasarre Grasa P.
De Antonio Pastor L.
Hospital Universitario de Fuenlabrada, Salud Mental , Fuenlabrada , Spain
* Corresponding author.
Keywords: Ekbom’s syndrome, Tactile hallucinations, Delusional disorder, Delusional parasitosis

Conflict of interest:

No

Article ID: EPV1220

An attachment perspective on high risk for psychosis: clinical correlates and psychosis’ predictive value of attachment patterns and mentalization

Di Cicilia G. 1 *
Boldrini T. 2
Tanzilli A. 1
Pontillo M. 3
Salcuni S. 2
Vicari S. 3
Lingiardi V. 1
1 Sapienza University of Rome , Department of Dynamic And Clinical Psychology, Rome , Italy
2 University of Padova , Department of Developmental Psychology And Socialization, Padua , Italy
3 Children Hospital Bambino Gesù , Department of Neuroscience, Child And Adolescence Neuropsychiatry Unit, Rome , Italy
* Corresponding author.
Keywords: reflective functioning, clinical high risk for psychosis, Attachment Patterns, mentalization

Conflict of interest:

No

Article ID: EPV1223

Baseline sociodemographic and clinical characteristics in the first episode psychosis program of navarra (pepsna).

García De Jalón E. 1 *
Martinez Moneo M. 2
Ariz M.C. 1
Azcarate L. 1
Fernandez Falces A. 1
1 Servicio Navarro de Salud, Servicio De Psiquiatría. Primeros Episodios ., Pamplona , Spain
2 SNS-O , Servicio De Psiquiatría. Primeros Episodios., Pamplona , Spain
* Corresponding author.
Keywords: First Episode Psychosis Program, premorbid functioning, Duration of Untreated Psychosis, Early treatment psychosis

Conflict of interest:

No

Article ID: EPV1224

Childhood trauma, schizophrenia and suicidality

García Eslava J.S. 1 *
Alvarez M.-J. 2
Roura-Poch P. 3
Tasa-Vinyals E. 2
Masramon H. 4
González-Vázquez A. 5
Foguet-Boreu Q. 2
1 Hospital Universitario de Vic, Psychiatry And Mental Health , Vic , Spain
2 Consorci Hospitalari Vic, Mental Health Depatment , Vic , Spain
3 Consorci Hospitalari Vic , Epidemilogy Department, Vic , Spain
4 Clínica Sant Josep, Mental Health Depatment , Vic , Spain
5 Complejo Hospitalario Universitario de A Coruña, Mental Health Depatment, A Coruña , Spain
* Corresponding author.
Keywords: schizophrenia, Suicide, Choldhood trauma, psychosis

Conflict of interest:

No

Article ID: EPV1227

Using moca for cognitive impairment screening in long-term psychosis patients

Gil Berrozpe G. 1 *
Sánchez-Torres A. 1
García De Jalón E. 1
Moreno-Izco L. 1
Fananas L. 2
Peralta V. 1
Cuesta M. 1
1 Complejo Hospitalario de Navarra , Servicio De Psiquiatría, Pamplona , Spain
2 University of Barcelona , Evolutionary Biology, Barcelona , Spain
* Corresponding author.
Keywords: schizophrenia, psychosis, Cognitive impairment, Cognitive screening

Conflict of interest:

No

Article ID: EPV1228

Cognitive profile of long-term outpatients with psychotic disorders in the mccb.

Sánchez-Torres A. *
Gil Berrozpe G.
Moreno-Izco L.
García De Jalón E.
Peralta V.
Cuesta M.
Group S.
Complejo Hospitalario de Navarra , Servicio De Psiquiatría, Pamplona , Spain
* Corresponding author.

Conflict of interest:

No

Article ID: EPV1229

Cognitive network analysis in psychosis

Sánchez-Torres A. 1
Gil Berrozpe G. 1 *
Moreno-Izco L. 1
Mezquida G. 2
Parellada M. 3
Corripio I. 4
Bernardo M. 5
Cuesta M. 1
Peps G. 1
1 Complejo Hospitalario de Navarra , Servicio De Psiquiatría, Pamplona , Spain
2 Hospital Clínic de Barcelona, Barcelona Clinic Schizophrenia Unit , Barcelona , Spain
3 University Hospital Gregorio Marañón , Child And Adolescent Psychiatry, Madrid , Spain
4 Hospital de la Santa Creu i Sant Pau , Psychiatry, Barcelona , Spain
5 Hospital Clinic, University of Barcelona, IDIBAPS, CIBERSAM , Schizophrenia Unit , Institute of Neuroscience, Barcelona , Spain
* Corresponding author.

Conflict of interest:

No

Article ID: EPV1230

The cains scale in schizophrenia spectrum psychosis. factorial and concurrent validity and its relationship with psychosocial functioning

Gil Berrozpe G. *
Sánchez-Torres A.
Moreno-Izco L.
Peralta V.
Cuesta M.
Complejo Hospitalario de Navarra , Servicio De Psiquiatría, Pamplona , Spain
* Corresponding author.
Keywords: CAINS, SANS, psychosis, psychosocial functioning

Conflict of interest:

No

Article ID: EPV1231

The impact of sex differences in negative symptoms and real-life functioning predictors in subjects with schizophrenia

Giordano G.M. 1 *
Galderisi S. 1
Mucci A. 1
Bucci P. 1
Vignapiano A. 1
Rossi A. 2
Rocca P. 3
Bertolino A. 4
Maj M. 1
1 University of Campania "Luigi Vanvitelli" , Department of Psychiatry, Naples , Italy
2 University of L’Aquila , Department of Biotechnological And Applied Clinical Sciences, Section Of Psychiatry , L’Aquila, Italy
3 University of Turin , Department of Neuroscience, Section Of Psychiatry , Turin , Italy
4 University of Bari , Department of Neurological And Psychiatric Sciences, Bari , Italy
* Corresponding author.
Keywords: negative symptoms, functioning, schizophrenia, Gender

Conflict of interest:

No

Article ID: EPV1233

Can the attitude of the patiens change to injectable medication when they know the existence of quarterly formulation?

Min Kim H.-B.J. 1
Velilla Diez L. 2
González Rodríguez I. 3 *
1 COMPLEJO ASISTENCIAL UNIVERSITARIO DE LEON , Psiquiatria , LEON , Spain
2 CAULE, Psychiatry, LEON , Spain
3 Complejo Asistencial Universitario de León , Psiquiatría, León , Spain
* Corresponding author.
Keywords: Attitude, schizophrenia, quarterly formulation

Conflict of interest:

No

Article ID: EPV1236

The prevalence of hyperprolactinemia among acute admitted women with recurrent schizophrenia

Herceg M. 1 *
Puljic K. 2
Herceg D. 3
1 School of Medicine, University of Zagreb , Croatia 2University Psychiatric Hospital Vrapče , Zagreb, Croatia, Department For Psychotic Disorders, Zagreb, Croatia
2 University Psychiatric Hospital Vrapče , Department For Psychotic Disorders, Zagreb , Croatia
3 School of Medicine, University of Zagreb , School Pf Medicine, Zagreb , Croatia
* Corresponding author.
Keywords: women, prolactin, acute admission, schizophrenia

Conflict of interest:

No

Article ID: EPV1237

Narrative therapy in psychosis recovery

Herranz-Herrer J. 1 *
Estevez-Peña B. 2
Gil-Benito E. 2
Corres-Fuentes Y. 1
Ponte-López T. 2
Boi S. 1
González-Salvador T. 1
Sánchez-Rivero I. 3
1 Hospital Universitario Puerta de Hierro de Majadhonda , Psychiatry, Majadahonda , Spain
2 Hospital Universitario Puerta de Hierro , Majadahonda, Madrid , Spain
3 Hospital Puerta de Hierro de Majadahonda , Majadahonda, Madrid , Spain
* Corresponding author.
Keywords: Recovery, psychosis, narrative, psychoeducation

Conflict of interest:

No

Article ID: EPV1238

Hero’s journey in psychosis recovery

Herranz-Herrer J. 1 *
Castelao Almodovar S. 1
Maguilla Franco P. 1
Solari-Heresmann L.M. 1
Ponte-López T. 2
Gil-Benito E. 2
Blasco-Fontecilla H. 1
1 Hospital Puerta de Hierro de Majadahonda , Majadahonda, Madrid , Spain
2 Hospital Universitario Puerta de Hierro , Majadahonda, Madrid , Spain
* Corresponding author.
Keywords: Hero’s Journey, psychosis, Recovery, narrative therapy

Conflict of interest:

No

Article ID: EPV1239

Auditory vocal hallucination treatment in outpatient’s clinic

Hjorth P. 1 *
Juel H. 2
1 Psychiatry, Region of Southern Denmark , Vejle, Vejle, Denmark
2 Psychiatry, Region of Southern Denmark , Outpatients Clinics, Vejle, Denmark
* Corresponding author.
Keywords: auditory verbal hallucinations, schizophrenia, group therapy

Conflict of interest:

No

Article ID: EPV1240

The outset of schizophrenia through suicide attempt

Iliuta F.P. 1 *
Buduru A. 1
Manea M. 1
Manea O. 2
1 Clinical Hospital of Psychiatry “Prof. Dr. Alexandru Obregia”, 7, Bucharest , Romania
2 Clinical Hospital CF2 , Psychiatry, Bucharest , Romania
* Corresponding author.
Keywords: Suicide, schizophrenia, rash act, risk factors

Conflict of interest:

No

Article ID: EPV1241

Tobacco intensive motivational and estimate risk (timer). study protocol for a randomized controlled trial

Jaen-Moreno M.J. 1 *
Ruiz Rull C. 2
Valdivia F. 3
Valverde M. 4
Osuna M.I. 5
Caro I. 5
Martin D. 5
Montiel F.J. 6
Sarramea Crespo F. 3
1 Instituto de Investigación Biomédica de Córdoba. Universidad de Córdoba , Psiquiatría, Córdoba , Spain
2 Instituto de Investigación Biomédica de Córdoba., Hospital Universitario Reina Sofía , Cordoba , Spain
3 Instituto de Investigación Biomédica de Córdoba., Hospital Universitario Reina Sofía. Psiquiatría , Cordoba , Spain
4 Hospital Ciudad de Jaen, Unidad De Salud Mental Andujar , Andujar , Spain
5 Hospital Virgen de la Victoria , Psiquiatría, malaga, Spain
6 Hospital Ciudad de Jaen, Unidad De Salud Mental , Jaen , Spain
* Corresponding author.
Keywords: chronic obstructive pulmonary disease, schizophrenia, Bipolar disorder, tobacco cessation

Conflict of interest:

No

Article ID: EPV1242

Readiness to change versus stage of change to predict the efficacy of tobacco reduction in severe mental illness

Gomez Moreno C. 1
Carrión Expósito L. 2
Del Pozo Seseña G.I. 3
Chauca Chauca G.M. 2
Alcalá Partera J.A. 3
Sanchez M.D. 1
Jaen-Moreno M.J. 4 *
Sarramea Crespo F. 5
1 Instituto de Investigación Biomédica de Córdoba. Universidad de Córdoba, Usmc Andujar , Córdoba , Spain
2 Servicio Andaluz de Salud, Ugc-salud Mental Hospital Infanta Margarita , Córdoba , Spain
3 Instituto de Investigación Biomédica de Córdoba., Hospital Universitario Reina Sofía. Psiquiatría , Córdoba , Spain
4 Instituto de Investigación Biomédica de Córdoba. Universidad de Córdoba , Psiquiatría, Córdoba , Spain
5 Instituto de Investigación Biomédica de Córdoba., Hospital Universitario Reina Sofía. Psiquiatría , Cordoba , Spain
* Corresponding author.
Keywords: tobacco cessation, Readiness to change, schizophrenia, Bipolar disorder

Conflict of interest:

No

Article ID: EPV1246

Cognitive remediation using virtual reality in inpatient acute setting: pilot study

Lipskaya-Velikovsky L. 1 *
Reut Cohen R. 2
Taubenblat H. 2
Welly E. 2
Harel E. 2
1 Tel Aviv University , Occupational Therapy, Tel Aviv , Israel
2 Beer-Ya’akov-Ness-Ziona-Maban Mental Health Center , Occupational Therapy , Beer Yaakov, Israel
* Corresponding author.
Keywords: Everyday functioning, schizophrenia, cognitive remediation, neurocognition

Conflict of interest:

No

Article ID: EPV1247

Negative symptoms in psychosis: approach and treatment

Mainar De Paz V. 1 *
Bravo Arráez M. 2
Martín Alvarez C. 3
1 Centro de Salud Mental Maresme Nord , Psiquiatría, Barcelona , Spain
2 Hospital Universitario Severo Ochoa , Psiquiatria, Madrid , Spain
3 Hospital Universitario de Fuenlabrada, Centro De Salud Mental , Madrid , Spain
* Corresponding author.

Conflict of interest:

No

Article ID: EPV1248

Clozapine-resistant schizophrenia

Martínez Fernández Á. *
Ramos García E.
Molina Cambra R.
Muñoz Domenjó A.
Sagarra Arruego R.
Bianchi Ramos F.L.
Ortega Moreno M.
Hernández Barrera M.
Hospital Universitario de Móstoles , Psiquiatría, Móstoles, Madrid , Spain
* Corresponding author.
Keywords: schizophrenia, clozapine, clozapine-resistant, clozapine-resistant schizophrenia

Conflict of interest:

No

Article ID: EPV1250

Erotomania in schizoaffective disorder - a treatment resistent case report

Mauricio J. *
Marino M.
Amorim M.
Pina P.
ULSAM , Psychiatry, Viana do Castelo , Portugal
* Corresponding author.
Keywords: treatment resistent, clerambault, erotomania, Schizoaffective disorder

Conflict of interest:

No

Article ID: EPV1251

Impact of schizophrenia and schizotypal disorders at the unified health system of brazil

Mieli G. 1 *
Mieli M. 2
1 Universidade Municipal de São Caetano do Sul , Psychiatry, São Paulo , Brazil
2 Universidade Municipal de São Caetano do Sul , Clinical Abilities, São Paulo , Brazil
* Corresponding author.
Keywords: schizophrenia, public healthcare, Schizotypal, brazil

Conflict of interest:

No

Article ID: EPV1252

Psychosis in noonan syndrome

Molina Lietor M.D.C. *
Santiago Moreno A.G.
Hospital Universitario Príncipe de Asturias , Psychiatry, Alcalá de Henares , Spain
* Corresponding author.
Keywords: Noonan, psychosis, Psychotic Symptoms, Social Cognition

Conflict of interest:

No

Article ID: EPV1255

Functionality improvement in patients suffering from schizophrenia in a real life clinical setting

Myta I. *
Aspradaki M.
Chioti V.
Theodoropoulou P.
Sismanogleio General Hospital , Psychiatric, Athens , Greece
* Corresponding author.
Keywords: schizophrenia, GAF, functionality

Conflict of interest:

No

Article ID: EPV1256

Psychotic disorder resulting from mephedrone use: a case repot

Nava P. 1 *
Nadales M. 1
Rodríguez-Quiroga A. 2
Martinez De Velasco R. 1
Mora F. 1
Matas A. 1
Banzo C. 1
1 Hospital Infanta Leonor , Psychiatry, Madrid , Spain
2 Hospital Universitario Infanta Leonor , Psychiatry, Madrid , Spain
* Corresponding author.
Keywords: chemsex, mephedrone, psychosis, drug

Conflict of interest:

No

Article ID: EPV1257

Psychotic disorder presenting in the context of a anti–n-methyl-d-aspartate receptor encephalitis

Nava P. 1 *
Banzo C. 1
Martinez De Velasco R. 1
Nieves M. 1
Matas Ochoa A.M. 2
Rodríguez-Quiroga A. 2
Mora F. 1
1 Hospital Infanta Leonor , Psychiatry, Madrid , Spain
2 Hospital Universitario Infanta Leonor , Psychiatry, Madrid , Spain
* Corresponding author.
Keywords: encephalitis, psychosis, anti-NMDAR

Conflict of interest:

No

Article ID: EPV1259

Comparison of schizofrenic and delusional patients hospitalized for first time in their life

Palau A. *
Khatib M.
Nieto E.
Isern C.
Puig M.
Xarxa Assitencial Althaia , Psychiatry, Manresa , Spain
* Corresponding author.
Keywords: Delusional disorder, schizofrenia, comparison, fist-time hospitalized

Conflict of interest:

No

Article ID: EPV1260

Is there social cognitive decline in psychosis? differences in performance in early and chronic ssd patients

Pastor Haro J. 1 *
De Diego Á. 1
Sánchez P. 1
Román Mazuecos E.M. 1
Cebolla S. 1
Vidal-Villegas M.P. 2
Mediavilla R. 2
Pinto García A. 2
Fernandez Gomez N. 1
Muñoz-Sanjose A. 1
Palao Á. 1
Lahera G. 3
Bayon C. 1
Rodríguez-Vega B. 1
Bravo-Ortiz M.F. 1
1 Hospital Universitario La Paz, Psiquiatría Y Salud Mental , Madrid , Spain
2 La Paz University Hospital Research Health Institute (IdiPAZ) , Psychiatry, Clinical Psychology And Mental Health , Madrid , Spain
3 University of Alcala , Faculty of Medicine, Madrid , Spain
* Corresponding author.
Keywords: Social Cognition, psychosis

Conflict of interest:

No

Article ID: EPV1265

Farmer with sturge weber syndrme to the leak

Rodriguez Calzada R. 1 *
Royo-Villanova B. 1
Delgado Alonso L. 2
1 HOSPITAL UNIVERSITARIO RIO HORTEGA DE VALLADOLID (SACYL) , Servicio De PsiquiatrÍa, VALLADOLID , Spain
2 HOSPITAL UNIVERSITARIO RIO HORTEGA DE VALLADOLID (SACYL) , Servicio De Urgencias, VALLADOLID , Spain
* Corresponding author.
Keywords: STURGE WEBER, psychosis, delusions

Conflict of interest:

No

Article ID: EPV1267

Chemotherapy induced brief psychotic disorder

Sánchez-Rivero I. *
Herranz-Herrer J.
Hospital Universitario Puerta de Hierro , Majadahonda, Madrid , Spain
* Corresponding author.
Keywords: chemotherapy, psychosis, Psychotic, induced

Conflict of interest:

No

Article ID: EPV1268

Teenage spiking humour – a clinical case

Santos M. 1 *
López P. 2
1 Hospital Prof. Doutor Fernando Fonseca , E.P.E., Department of Psychiatry, Amadora , Portugal
2 Bizkaia Mental Health Network , Lehenak Program, Bilbao , Spain
* Corresponding author.
Keywords: psychosis, mania, Epilepsy, First episode

Conflict of interest:

No

Article ID: EPV1269

The role of artistic language as a mean of expression/communication for schizophrenic patients

Sanz-Aranguez Avila B. 1 *
Del Rio M. 2
Pérez-Balaguer A. 3
Boi S. 1
1 Hospital Universitario Puerta de Hierro de Majadhonda , Psychiatry, Majadahonda , Spain
2 AUTONOMA UNIVERSITY OF MADRID , Art Education And Visual Arts, Madrid , Spain
3 Hospital Puerta de Hierro de Majadahonda , Majadahonda, Madrid , Spain
* Corresponding author.
Keywords: verbal language, artistic language, art therapy, schizophrenia

Conflict of interest:

No

Article ID: EPV1270

Low-dose corticosteroid-induced psychosis: a case report

Saykal S.G. *
Ileri T.
Kaya H.
Göka E.
Health Sciences University- Ankara City Hospital , Psychiatry, Ankara , Turkey
* Corresponding author.
Keywords: Corticosteroid, Delusional disorder, Low-dose, psychosis

Conflict of interest:

No

Article ID: EPV1272

First psychotic episode and incidental meningioma

Soldado Rodriguez L. 1 *
Valverde Barea M. 2
Ruiz Martínez G.M. 3
1 Neurotraumatological Hospital , Mir , Mental Health Unit , Jaen , Spain
2 COMPLEJO HOSPITALARIO DE JAEN , Psiquiatria , JAEN , Spain
3 Neurotraumatological Hospital, Mental Health Hospitalization Unit , Jaen , Spain
* Corresponding author.
Keywords: urgencies, First psychotic episode, meningioma, Involuntary hospitalization

Conflict of interest:

No

Article ID: EPV1274

Kretschmer’s sensitive delusion of reference: a psychiatry archaism? a case report.

Sousa R. *
Brás J.
Costa A.
Guedes B.
Cunha N.
Centro Hospitalar Tondela-Viseu , Department of Psychiatry And Mental Health, VISEU , Portugal
* Corresponding author.
Keywords: delusion, kretschmer, sensitive, paranoia

Conflict of interest:

No

Article ID: EPV1275

Late onset psychosis: a challenging diagnosis. case report.

Sousa R. *
Brás J.
Costa A.
Guedes B.
Cunha N.
Centro Hospitalar Tondela-Viseu , Department of Psychiatry And Mental Health, VISEU , Portugal
* Corresponding author.
Keywords: schizophrenia, lateonset, psychosis, paraphrenia

Conflict of interest:

No

Article ID: EPV1277

Paliperidone palmitate long-acting injection in schizophrenic patients

Suarez Gisbert E.
HOSPITAL UNIVERSITARIO INFANTA SOFIA , Psychiatry, SAN SEBASTIAN DE LOS REYES, Spain
Keywords: paliperidone palmitate, SCHIZOPHRENIC PATIENTS, Negative symtons, Metabolic rates

Conflict of interest:

No

Article ID: EPV1278

Comparative of the effectiveness of long-acting injections of second generation antipsychotic

Suarez Gisbert E.
HOSPITAL UNIVERSITARIO INFANTA SOFIA , Psychiatry, SAN SEBASTIAN DE LOS REYES, Spain
Keywords: Effectiveness of antipsychotic, second generation antipsychotic

Conflict of interest:

No

Article ID: EPV1279

A case of hysteric psychosis symptomed with childish behaviors

Akıncı Ş. 1
Taner M.E. 1
Teksin Bakir M.G. 2 *
1 Gazi University , Psychiatry, ANKARA , Turkey
2 Dr. AY Ankara Oncology Training and Research Hospital, Psychiatry Department, ANKARA , Turkey
* Corresponding author.
Keywords: hysteric psychosis, trauma

Conflict of interest:

No

Article ID: EPV1281

Compliance and cognitive functioning in patientswith first episode of schizophrenia

Tsyrenova K.
FSBEI St. Petersburg State University , Department of Psychiatry And Narcology, Saint-Petersburg , Russian Federation
Keywords: cognitive functions, compliance, schizophrenia, first episode schizophrenia, cognitive functions, compliance

Conflict of interest:

No

Article ID: EPV1282

Analysis of diffusion parameters in the cst of patients with early stage of schizophrenia. dti study.

Ublinskiy M. *
Semenova N.
Akhadov T.
Manzhurtsev A.
Yakovlev A.
Clinical and Research Institute of Emergency Pediatric Surgery and Trauma, Radiology, Radiology , Moscow , Russian Federation
* Corresponding author.
Keywords: schizophrenia, corticospinal tract, DTI, MRI

Conflict of interest:

No

Article ID: EPV1283

Naa dynamics in motor cortex of normal individuals and schizophrenia patients in the period of event related bold response.

Ublinskiy M. *
Semenova N.
Akhadov T.
Manzhurtsev A.
Yakovlev A.
Clinical and Research Institute of Emergency Pediatric Surgery and Trauma, Radiology, Radiology , Moscow , Russian Federation
* Corresponding author.
Keywords: schizophrenia, Functional MRI, magnetic resonance spectroscopy, N-acetyleaspartate

Conflict of interest:

No

Article ID: EPV1286

Feasibility of 6 months outpatient cognitive remediation in schizophrenia: experience from the randomized controlled isst-study

Wölwer W. *
Lowe A.
Weide K.
Isst-Study Group F.
University of Düsseldorf, LVR-Klinikum Düsseldorf , Department of Psychiatry And Psychotherapy, Düsseldorf , Germany
* Corresponding author.
Keywords: schizophrenia, Feasibility, cognitive remediation, safety

Conflict of interest:

No

Article ID: EPV1287

Influence of age on the subjective well-being in endogenous psychosis

Yamada T. *
Sakamoto H.
Okutani K.
Hyogo University of Health Sciences, Occupational Therapy School of Rehabilitaion , Kobe , Japan
* Corresponding author.
Keywords: Subjective Well-being, Endogenous Psychosis, Age, SWNS-J

Conflict of interest:

No

Article ID: EPV1289

Emotional facial recognition deficits in patients with alzheimer’s disease and schizophrenia: trans-diagnostic effects of social functioning factors

Vieira-Campos A. 1
Ayuso-Mateos J.L. 2 *
Carreras-Rodriguez M.T. 1
Vivancos J. 1
Bilderbeck A. 3
Aghajani M. 4
Lopez-Montoya G. 5
Martinez C. 5
Arango C. 6
De La Torre-Luque A. 7
1 La Princesa University Hospital , Neurology, Madrid , Spain
2 Universidad Autonoma de Madrid , Psychiatry, Madrid , Spain
3 P1 Vital Ltd , Psychopharmacology And Emotion Research Laboratory, Wallingford , United Kingdom
4 Amsterdam UMC/VUmc , Department of Psychiatry, Amsterdam , Netherlands
5 Gregorio Marañon University Hospital , Psychiatry, Madrid , Spain
6 Hospital General Universitario Gregorio Marañón , Child And Adolescent Department of Psychiatry, Madrid , Spain
7 Center of Biomedical Research in Mental Health (CIBERSAM)., Department of Psychiatry. Universidad Autonoma De Madrid , Madrid , Spain
* Corresponding author.
Keywords: social functioning, schizophrenia, dementia, emotion recognition

Article ID: EPV1290

Do negative symptoms worsen functionality in early psychosis?

Becerra Darriba H.
Osasunbidea - Servicio Navarro de Salud, Centro De Salud Mental De Tudela , Tudela , Spain
Keywords: negative symptoms, functionality, Cognition, early psychosis.

Conflict of interest:

No

Article ID: EPV1291

Features of the syndrome of dysmorfofobia in patients with schizophrenia

Belokrylov I. *
Brukhin A.
Lineva T.
Karnozov V.
Kirsanova G.
Muzychenko G.
Peoples’ Friendship University of Russia ( RUDN University ), Department of Psychiatry And Medical Psychology, Moscow , Russian Federation
* Corresponding author.
Keywords: dysmorfofobia, schizophrenia, clinical prognosis

Article ID: EPV1294

Secondary psychosis due to a systemic infection? a case report

Coucheiro Limeres P. 1 *
Aguilar Romero M.D.C. 2
González Juárez C. 2
1 Instituto Psiquiátrico José Germain , Psychiatry, Leganés , Spain
2 Hospital Severo Ochoa , Psychiatry, Leganés , Spain
* Corresponding author.
Keywords: Secondary psychosis, Neurological functional disorder, Systemic infection

Conflict of interest:

No

Article ID: EPV1296

Treatment of delusional ideas of poisoning with paliperidone prolonged release. diagnostic discussion. case report

Del Sol Calderón P. *
Izquierdo De La Puente Á.
Garcia Moreno M.
HOSPITAL UNIVERSITARIO PUERTA DE HIERRO MAJADAHONDA , Psychiatry, MADRID , Spain
* Corresponding author.
Keywords: tactil hallucinosis, delusional ideas, antipsychotic

Conflict of interest:

No

Article ID: EPV1297

Cognitive deficit in schizophrenia and epilepsy: common aspects

Esparrago Llorca G. 1 *
Rodriguez Parrón M. 1
Carrión Expósito L. 2
Bordes Giménez M.D. 3
Díaz Fernández F. 3
Andrés Pereira G. 3
Juncosa Montes P. 3
1 Servicio Extremeño de Salud, Equipo De Salud Mental , Cáceres , Spain
2 Servicio Andaluz de Salud, Ugc-salud Mental Hospital Infanta Margarita , Córdoba , Spain
3 Servicio Extremeño de Salud, Hospital San Pedro De Alcántara , Cáceres , Spain
* Corresponding author.
Keywords: schizophrenia, epilepsy, cognitive deficit

Conflict of interest:

No

Article ID: EPV1298

Benign paranoia, sensitive delusion of reference case report

Garcia Moreno M. 1 *
De Cós Milas A. 2
Beatobe Carreño L. 2
Del Sol Calderón P. 1
Izquierdo De La Puente Á. 1
Vizcaíno Da Silva M. 3
Poza Cano M.B. 3
1 CENTRO DE SALUD MENTAL MAJADAHONDA.HOSPITAL UNIVERSITARIO PUERTA DE HIERRO MAJADAHONDA , Psychiatry, MADRID , Spain
2 CENTRO DE SALUD MENTAL MÓSTOLES. HOSPITAL UNIVERSITARIO DE MÓSTOLES , Psychiatry, MADRID , Spain
3 CENTRO ESPECIALIDADES SAN CARLOS. HOSPITAL UNIVERSITARIO EL ESCORIAL , Psychiatry, MADRID , Spain
* Corresponding author.

Conflict of interest:

No

Article ID: EPV1300

Impulsivity in subjects at ultra high risk for psychosis.

Ghrissi F. *
Fekih F.
Razi Hospital, Psychiatry University Tunis El Manar , Mannouba , Tunisia
* Corresponding author.
Keywords: Impulsivity, ultra-high risk for psychosis UHR, early phases of psychosis, Behavioral disorders

Conflict of interest:

No

Article ID: EPV1301

Clozapine and myocarditis: a case report

Izquierdo De La Puente Á. 1 *
Del Sol Calderón P. 1
Garcia Moreno M. 1
Mendez Gonzalez O. 1
Vizcaíno Da Silva M. 1
Fernández Fernández R. 2
Rodríguez Rodríguez A. 1
1 HOSPITAL UNIVERSITARIO PUERTA DE HIERRO MAJADAHONDA , Psychiatry, MADRID , Spain
2 Hospital Universitario HM Puerta del Sur , Psychiatry, Mostoles , Spain
* Corresponding author.
Keywords: clozapine, Myocarditis

Conflict of interest:

No

Article ID: EPV1302

Endocannabinoid system and dual dissorder: a case report

Izquierdo De La Puente Á. *
Del Sol Calderón P.
Garcia Moreno M.
HOSPITAL UNIVERSITARIO PUERTA DE HIERRO MAJADAHONDA , Psychiatry, MADRID , Spain
* Corresponding author.
Keywords: schizophrenia, dual dissorder, Endocannabinoid system

Conflict of interest:

No

Article ID: EPV1306

Cortico-induced psychotic episode: a case report

Kabtni W. 1 *
Baatout A. 2
Rebai A. 3
Ben Cheikh C. 2
El Kefi H. 4
Oumaya A. 4
1 military hospital of Tunis , Psychiatry Department, Tunis , Tunisia
2 Military hospital of Tunis , Psychiatry Department, Tunis , Tunisia
3 Razi University Hospital , Adult Outpatient Psychiatry Department, Mannouba , Tunisia
4 military hospital of tunis, Psycihiatric Unit , tunis , Tunisia
* Corresponding author.
Keywords: psychosis, corticoide, side effect

Conflict of interest:

No

Article ID: EPV1308

Medication compliance in schizophrenia: a qualitative study of patients’ perspectives

Laranjeira C.
Piaget Institute - RECI I&D, Higher School of Health Sciences , Viseu , Portugal

Conflict of interest:

No

Article ID: EPV1310

Mother-child bond and schizophrenia in treatment with paliperidone palmitate. clinical case.

Pérez Sánchez S. 1 *
Martín Herrero I. 1
Crespo Portero A. 2
Güimil Raya D. 1
Cassinello Marco M. 1
1 Morales Meseguer Public University Hospital , Psychiatry, Murcia , Spain
2 Lorca Mental health Center , Psychiatry, Lorca , Spain
* Corresponding author.
Keywords: Mother-child attachment, schizophrenia, parenting

Conflict of interest:

No

Article ID: EPV1312

Aripiprazole long-acting injectable in schizophrenia. A 33 months follow-up

Romero Guillena S.L. 1 *
Plasencia Garcia De Diego B.O. 2
1 U.G.C. Salud Mental Virgen Macarena , Psychiatry, Seville , Spain
2 U.G.C. Salud Mental Virgen del Rocio , Psychiatry, Sevilla , Spain
* Corresponding author.
Keywords: long-term antipsychotic, Aripiprazole long-acting injectable, schizophrenia

Conflict of interest:

No

Article ID: EPV1313

Paliperidone palmitate 3 month formulation for the treatment of schizophrenia: a 36 months follow-up study

Romero Guillena S.L. 1 *
Plasencia Garcia De Diego B.O. 2
1 U.G.C. Salud Mental Virgen Macarena , Psychiatry, Seville , Spain
2 U.G.C. Salud Mental Virgen del Rocio , Psychiatry, Sevilla , Spain
* Corresponding author.
Keywords: long-term antipsychotic, Paliperidone palmitate 3-month formulation, schizophrenia

Conflict of interest:

No

Article ID: EPV1315

Machiavellianism factors in patients with various forms of schizophrenia

Sokolova E. 1
Oleichik I. 2
Andreyuk K. 1
Ryzhov A. 1 *
1 Lomonosov MSU , Faculty of Psychology, Moscow , Russian Federation
2 Mental Health Research Centre , Department of Endogenous Mental Disorders And Affective Conditions, Moscow , Russian Federation
* Corresponding author.
Keywords: schizophrenia, schizotypal disorder, Machiavellianism, values

Conflict of interest:

No

Article ID: EPV1316

Smooth pursuit eye movements in different schizophrenia dimensions

Skuhareuskaya M. 1 *
Khamenka N. 2
Skugarevsky O. 3
Skuhareuskaya T. 2
Obyedkov I. 4
1 The Republican Research and Practice Mental Health Center , Psychiatry, Minsk , Belarus
2 Belarusian State Medical University , Psychiatry And Medical Psychology, Minsk , Belarus
3 Belarusian State Medical University , Psychiatry, minsk , Belarus
4 The Republican Research and Practice Mental Health Center , Narcology , Minsk , Belarus
* Corresponding author.
Keywords: schizophrenia, smooth pursuit eye movements, dimensions

Conflict of interest:

No

Article ID: EPV1317

Controversy and current relevance of the diagnosis of simple schizophrenia: a case report.

Torres Varona F.J. *
Benavente López S.
Plaza Yuste M.
Hernández Caro C.M.
Cozar Ortiz J.
Torrente Seoane C.
Rodríguez Delgado C.
Ainslie Mata Y.
Iglesias García C.
Presa García M.
Hospital Central de la Defensa Gómez Ulla, Psychiatry And Mental Health Department , Madrid , Spain
* Corresponding author.
Keywords: clozapine, simple schizophrenia, attenuated psychosis

Conflict of interest:

No

Article ID: EPV1318

Prevalence of physical illness in relatives of patients diagnosed with schizophrenia

Torres Varona F.J. 1 *
García Ramos P. 2
Benavente López S. 1
Gutiérrez Ortega C. 1
Losantos Pascual R. 1
Torrente Seoane C. 1
Plaza Yuste M. 1
Cozar Ortiz J. 1
Hernández Caro C.M. 1
Rodríguez Delgado C. 1
Ainslie Mata Y. 1
Presa García M. 1
1 Hospital Central de la Defensa Gómez Ulla, Psychiatry And Mental Health Department , Madrid , Spain
2 Private Practice, Private Practice, Madrid , Spain
* Corresponding author.
Keywords: physical illness, schizophrenia, relatives, medical comorbidity

Conflict of interest:

No

Article ID: EPV1319

Multiple sclerosis or extrapyramidal symptoms secondary to treatment with antipsychotics in psychosis. the importance of differential diagnosis. about a case

Valverde Barea M. 1 *
Alvarado Dafonte A. 1
Cartas Moreno F. 2
1 COMPLEJO HOSPITALARIO DE JAEN , Psiquiatria , JAEN , Spain
2 HOSPITAL SAN JUAN DE LA CRUZ, UBEDA , Psiquiatria, JAEN , Spain
* Corresponding author.
Keywords: multiple sclerosis, extrapyramidal symptoms, antipsychotics, psychosis

Conflict of interest:

No

Article ID: EPV1320

Efficacy of the new formulations of atypical antipsychotics in patients with chronic schizophrenia

Vasile D. *
Vasiliu O.
Vasiliu D.
Vasile F.
University Emergency Central Military Hospital Dr. Carol Davila , Psychiatry, Bucharest , Romania
* Corresponding author.
Keywords: olanzapine, paliperidone, atypical antipsychotics, long acting injectable antipsychotics

Article ID: EPV1322

Uses of clozapine and depot treatment in patients diagnosed with schizophrenia in calatayud’s region

Ortigosa Bea C.
Pérez Ginés B.
Abad Bouzán C. *
HELL, Mental Salud , calatayud , Spain
* Corresponding author.
Keywords: clozapine, schizophrenia, Depot injections, treatment

Conflict of interest:

No

Article ID: EPV1327

Relevance of butyrylcholinesterase assay in the investigation of schizophrenia

Jemal K. 1 2
Maalej Bouali M. 3
Naifar M. 1 2
Guidara W. 1 2
Mseddi M. 1
Omri S. 3
Smaoui N. 3
Maalej M. 3
Charfi N. 3
Ayedi F. 1 2 *
1 Faculty of Medicine, Ur :12es17 « Molecular Bases Of Human Pathology », Sfax , Tunisia
2 Habib Bourguiba University Hospital , Laboratory of Biochemistry, Sfax , Tunisia
3 Hedi Chaker University Hospital , Psychiatry C Department, Sfax , Tunisia
* Corresponding author.
Keywords: schizophrenia, Butyrylcholinesterase

Conflict of interest:

No

Article ID: EPV1330

Psychometric properties and functional correlates of the italian version of the “cognitive assessment interview” (cai) in a large sample of subjects with schizophrenia

Brando F. 1 *
Palumbo D. 1
Giordano G.M. 1
Aiello C. 1
Mucci A. 1
Bucci P. 1
Rocca P. 2
Rossi A. 3
Bertolino A. 4
Galderisi S. 1
Maj M. 1
1 Università degli Studi della Campania "Luigi Vanvitelli" , Department of Psychiatry, napoli , Italy
2 University of Turin , Department of Neuroscience, Section Of Psychiatry , Turin , Italy
3 University of L'Aquila , Department of Applied Clinical Sciences And Biotechnologies, L'Aquila, Italy
4 University of Bari , Department of Neurological And Psychiatric Sciences, Bari , Italy
* Corresponding author.

Conflict of interest:

No

Article ID: EPV1333

Persistent negative symptoms in individuals with first-episode of psychosis: six-month follow-up.

Cuñat O. 1 *
Del Hoyo B. 1
Butjosa A. 1 2 3
Vila-Badia R. 1 3
Serra-Arumi C. 1 3
Del Cacho N. 1 3
Usall J. 1
1 Parc Sanitari Sant Joan de Déu, Research Unit , Sant Boi de Llobregat (Barcelona), Spain
2 Maternity and Child Hospital Sant Joan de Déu, Research Unit , Esplugues de Llobregat (Barcelona), Spain
3 Sant Joan de Déu Foundation, Research Unit , Esplugues de Llobregat (Barcelona), Spain
* Corresponding author.
Keywords: first-episode psychosis, drug-naive, Persistent negative symptoms, negative symptoms

Conflict of interest:

No

Article ID: EPV1335

Negative symptoms in drug-naive patients with a first-episode psychosis

Del Hoyo B. 1 *
Cuñat O. 1
Butjosa A. 1 2 3
Vila-Badia R. 1 3
Serra-Arumi C. 1 3
Del Cacho N. 1 3
Usall J. 1
1 Parc Sanitari Sant Joan de Déu, Research Unit , Sant Boi de Llobregat (Barcelona), Spain
2 Maternity and Child Hospital Sant Joan de Déu, Research Unit , Esplugues de Llobregat (Barcelona), Spain
3 Sant Joan de Déu Foundation, Research Unit , Esplugues de Llobregat (Barcelona), Spain
* Corresponding author.
Keywords: first-episode psychosis, negative symptoms, drug naive

Conflict of interest:

No

Article ID: EPV1336

Cognitive impairment in schizophrenia and its association with personality types

Dzagania N. 1 *
Makhatadze N. 2
1 European University , Faculty of Medicine, Tbilisi , Georgia
2 Center of mental health and drug addiction , Psychiatry, Tbilisi , Georgia
* Corresponding author.
Keywords: Cognitive impairment, severity scale, personality profile, schizophrenia

Conflict of interest:

No

Article ID: EPV1339

“Geneva’s syndrome: an emerging clinical entity?”

Giulia C. *
Othman S.
Michel S.
HUG , Adult Psychiatrie, thonex , Switzerland
* Corresponding author.
Keywords: pathological journey, psychotic disorder, Geneva syndrome, retrospective study

Conflict of interest:

No

Article ID: EPV1342

What specific metacognitive deficits underlie lack of insight in schizophrenia?

Lopez-Morinigo J.-D. 1 *
González Ruiz-Ruano V. 1
Sánchez Escribano-Martínez A. 2
Sánchez-Alonso S. 2
Mata-Iturralde L. 2
Muñoz Lorenzo L. 2
Baca-García E. 3
1 Universidad Autónoma de Madrid , Psychiatry, Madrid , Spain
2 Hospital Universitario Fundación Jiménez Díaz , Psychiatry, Madrid , Spain
3 Fundación Jiménez Díaz University Hospital , Psychiatry, Madrid , Spain
* Corresponding author.

Conflict of interest:

No

Article ID: EPV1343

Influence of aging in cognitive functioning of patients diagnosed with schizophrenia spectrum disorders

Malliaris P. 1 *
Bonotis K. 2
1 General Hospital of Karditsa , Psychiatry, Karditsa , Greece
2 University of Thessaly , Psychiatry, Larisa , Greece
* Corresponding author.
Keywords: Cognitive assessment, Clinician-Rated Dimension of Psychosis Symptom Severity, Aging, schizophrenia spectrum disorders

Conflict of interest:

No

Article ID: EPV1345

Early access to clozapine in early intervention in psychosis services: hope versus reality. A mixed method service analysis.

Nikolic N. 1 2 *
Whale R. 3 4
Hill K. 4 5
Campbell E. 6
Wickramasinghe V. 5
1 NHS England , Early Intervention In Psychosis Programme, Horley , United Kingdom
2 Sussex Partnership NHS Foundation Trust , Early Intervention In Psychosis Service, Worthing , United Kingdom
3 Sussex Partnership NHS Foundation Trust , Early Intervention In Psychosis Service, Hove , United Kingdom
4 Brighton and Sussex Medical School, 2. Department of Medical Education, Brighton , United Kingdom
5 Sussex Partnership NHS Foundation Trust , Early Intervention In Psychosis Service, Hailsham , United Kingdom
6 Sussex Partnership NHS Foundation Trust, Research And Development , Hove , United Kingdom
* Corresponding author.
Keywords: early intervention in psychosis, clozapine, First Episode Psychosis, Treatment Resistant Schizophrenia

Conflict of interest:

No

Article ID: EPV1351

The efficacy and safety of cariprazine in schizophrenia patients with insufficient effectiveness of previous antipsychotic therapy: a latvian observational study

Rancans E. 1 *
Barabássy Á. 2
Sebe B. 2
Skrivele I. 3
Dombi Z. 2
Németh G. 2
1 Riga Stradins University , Psychiatry And Addiction Disorders, Riga , Latvia
2 Richter Gedeon Plc ., Medical Division, Budapest , Hungary
3 Richter Gedeon Plc ., Representative Office Of Latvia , Marupe , Latvia
* Corresponding author.
Keywords: observational study, schizophrenia, Cariprazine

Article ID: EPV1354

Personal and social recovery of patients with schizophrenia in the treatment of long-acting antipsychotics

Serazetdinova V. *
Petrova N.
Saint Petersburg State University , Psychiatry, St. Petersburg , Russian Federation
* Corresponding author.
Keywords: Neurophysiology, schizophrenia, Recovery

Conflict of interest:

No

Article ID: EPV1356

Reviewing process of guidelines for severe psychiatric outpatient care in japan

Tanoue M. 1 *
Hirabayashi M. 2
Okubo S. 3
1 Totho University , Makuhari Human Care Department, Chiba City , Japan
2 International Agency for Research on Cancer, Infection And Cancer Epidemiology Group, Lyon , France
3 BMS Yokohama Inc., And Ritsumeikan University , Yokohama, Japan
* Corresponding author.
Keywords: care guidelines, community living, severe psychiatric outpatient care, family care

Conflict of interest:

No

Article ID: EPV1359

Urban living and psychosis: state of the art and challenges for therapy

Abrahamyan Empson L. *
Conus P.
CHUV , Psychiatry Department, Prilly , Switzerland
* Corresponding author.
Keywords: urbanicity, experience-based studies, psychosis, Early intervention

Conflict of interest:

No

Article ID: EPV1366

Risperidone plasma levels in patients with delusional disorder: the relevance of therapeutic drug monitoring.

González-Rodríguez A. 1 *
Guàrdia Delgado A. 2
Álvarez Pedrero A. 1
Betriu M. 2
Sanz N. 1
Acebillo S. 1
Parra I. 3
Monreal J.A. 4
Palao Vidal D. 3
Labad J. 3
1 Parc Taulí University Hospital . Autonomous University of Barcelona (UAB) . I3PT, Mental Health , Sabadell , Spain
2 Parc Taulí University Hospital, Mental Health , Sabadell , Spain
3 Parc Taulí University Hospital . Autonomous University of Barcelona (UAB) . I3PT. CIBERSAM, Mental Health , Sabadell , Spain
4 Parc Taulí University Hospital . Autonomous University of Barcelona (UAB) . I3PT , CIBERSAM, Mental Health , Sabadell , Spain
* Corresponding author.
Keywords: drug monitoring, Delusional disorder, psychosis, Antipsychotic plasma levels

Article ID: EPV1367

Epidemiological approach to the delusional disorder

Guermazi A. *
Omri S.
Smaoui N.
Feki R.
Maalej Bouali M.
Charfi N.
Zouari L.
Ben Thabet J.
Maalej M.
Hedi Chaker University Hospital , Psychiatry C Department, Sfax , Tunisia
* Corresponding author.
Keywords: Delusional disorder, epidemiological approach, evolution

Conflict of interest:

No

Article ID: EPV1368

Schizencephaly associated with psychosis: a tunisian case report

Hamdoun J. *
Ben Hamadi A.
Ben Ammar H.
Khelifa E.
Aissa A.
Zouhaier E.H.
Razi hospital , Psychiatry F, Manouba , Tunisia
* Corresponding author.
Keywords: Schizencephaly, psychosis

Conflict of interest:

No

Article ID: EPV1369

Factors influencing the duration of untreated psychosis in a tunisian sample

Hamdoun J. *
Ben Hamadi A.
Ben Ammar H.
Khelifa E.
Aissa A.
Zouhaier E.H.
Razi hospital , Psychiatry F, Manouba , Tunisia
* Corresponding author.
Keywords: Duration of Untreated Psychosis, predictive factors

Conflict of interest:

No

Article ID: EPV1370

Psychosis or dementia? difficulties in mental health diagnosis

Lopes L. 1 *
Nombora O. 2
1 Centro Hospitalar de Vila Nova de Gaia/Espinho , Serviço De Psiquiatria, Vila Nova de Gaia , Portugal
2 Centro Hospitalar Vila Nova de Gaia/Espinho , Serviço De Psiquiatria, Vila Nova de Gaia , Portugal
* Corresponding author.
Keywords: psychosis, dementia, treatment, very late-onset psychosis

Conflict of interest:

No

Article ID: EPV1371

Development of an early intervention in psychosis program in madrid (spain)

Louzao-Rojas I.I. 1 *
Muñoz-Sanjose A. 2
Bravo-Ortiz M.F. 3
Sánchez P. 1
Mediavilla R. 3
Rojano P. 1
Gotor L. 1
Rivelles V. 1
1 La Paz University Hospital ., Department of Psychiatry, Clinical Psychology And Mental Health , Madrid , Spain
2 La Paz University Hospital Institute for Health Research (IdiPAZ) , Psychiatry, Clinical Psychology And Mental Health , Madrid , Spain
3 La Paz University Hospital Research Health Institute (IdiPAZ) , Psychiatry, Clinical Psychology And Mental Health , Madrid , Spain
* Corresponding author.
Keywords: Early intervention, prodrome, psychosis, schizophrenia

Conflict of interest:

No

Article ID: EPV1372

The relationship between neurocognition, social cognition and functional capacity in patients with first-episode schizophrenia

Rodriguez-Jimenez R. 1 2 *
Martinez-Gras I. 1
Espejo-Saavedra Roca J.M. 1
Sanchez Pastor L. 1
Dompablo M. 1 2
Fernandez Sotos P. 1 2 3
Fernandez Cancer P. 1
Garcia Lopez A. 1
Fares-Otero N. 1
1 Department of Psychiatry, Instituto De Investigación Sanitaria Hospital 12 De Octubre (imas12), Madrid , Spain
2 CIBERSAM, (biomedical Research Networking Centre In Mental Health) , Madrid , Spain
3 Complejo Hospitalario Universitario de Albacete, Servicio De Salud Mental , Albacete , Spain
* Corresponding author.
Keywords: Neurocognition, Social Cognition, Functional Capacity, Schizophrenia

Conflict of interest:

No

Article ID: EPV1373

Social cognition and neurocognition using the matrics consensus cognitive battery in patients with first-episode schizophrenia: relationship with insight

Rodriguez-Jimenez R. 1 2 *
Caballero-Gonzalez M. 1 2
Torio I. 1 2
Sánchez-Morla E. 1 2
Rentero Martin D. 1 2
Dompablo M. 1 2
Garcia Lopez A. 1
Fernandez Sotos P. 1 2 3
Fernandez Cancer P. 1
Fares-Otero N. 1
1 Department of Psychiatry, Instituto De Investigación Sanitaria Hospital 12 De Octubre (imas12), Madrid , Spain
2 CIBERSAM, (biomedical Research Networking Centre In Mental Health) , Madrid , Spain
3 Complejo Hospitalario Universitario de Albacete, Servicio De Salud Mental , Albacete , Spain
* Corresponding author.
Keywords: Insight, Neurocognition, Social Cognition, Schizophrenia

Conflict of interest:

No

Article ID: EPV1377
Subjects: Abstract, Sexual Medicine and Mental Health

The role of sexual behaviour in the assessement of female sexual function among tunisian medical trainees: an exploratory study

Bouzaabia Z. 1 *
Regaya M. 1
Souilem A. 2
Mtiraoui A. 2
Elkissi Y. 2
Ben Nasr S. 1
1 University of Sousse , Faculty of medicine, Farhat Hached Hospital , Psychiatry Department, Lr12es04, SOUSSE , Tunisia
2 University of Sousse , Faculty of medicine, Farhat Hached Hospital. Tunisian society of clinical sexology , Psychiatry Department, Lr12es04, SOUSSE , Tunisia
* Corresponding author.
Keywords: Female sexual dysfunction, sexual health, Female medical trainees, FSFI

Conflict of interest:

No

Article ID: EPV1378

Cannabis use and sexual experience: a paraplegic patient perspective

Fernandes E. 1 *
Castanheira L. 1
Gonçalves M.J. 1
Alves F. 2
Telles-Correia D. 3
1 Centro Hospitalar Universitário Lisboa Norte, Serviço De Psiquiatria E Saúde Mental , Lisboa , Portugal
2 Unidade Local do Alto Minho- EPE , Departamento De Psiquiatria E Saúde Mental De Viana Do Castelo- Portugal , Viana do Castelo , Portugal
3 University of Lisbon, Centro Hospitalar Psiquiátrico De Lisboa , Lisbon , Portugal
* Corresponding author.
Keywords: cannabis, sexuality, sexual function, paraplegia

Conflict of interest:

No

Article ID: EPV1381

Physical health and safety issues in patients with chronic antipsychotic-related hyperprolactinemia.

Montejo Á. 1 *
Acosta J. 2
Prieto N. 3
Martin-Pinto T. 3
Gallego M.T. 3
Sanchez-Iglesias S. 3
Bote B. 3
Notario M. 3
Buch B. 2
1 UNiversity of Salamanca , Psychiatry Euef, Salamanca , Spain
2 IBSAL , Neurosciences, Salamanca , Spain
3 Hospital Universitario , Psychiatry, Salamanca , Spain
* Corresponding author.
Keywords: adverse effects, physical safety, antipsychotic, Hyperprolactinemia

Article ID: EPV1382

Is taking a sexual history still a taboo: a cross sectional study.

Radauš S. 1 *
Kirigin M. 2
Arbanas G. 3
1 General Hospital Karlovac , Department of Psychiatry, Karlovac , Croatia
2 General Hospital Dubrovnik , Department of Psychiatry, Dubrovnik , Croatia
3 University Psychiatric Hospital Vrapče , Department For Forensic Psychiatry, Zagreb , Croatia
* Corresponding author.
Keywords: sexual history, acute inpatient unit, Psychiatry, Sexual Medicine

Conflict of interest:

No

Article ID: EPV1383

Sexual assault related post traumatic stress disorder: a profile overview

Rema J. *
Lemos M.
Jerónimo J.
Cavaco T.
Silva C.
Queirós T.
Croca M.
Centro Hospitalar Universitário Lisboa Norte, Serviço De Psiquiatria E Saúde Mental , Lisboa , Portugal
* Corresponding author.
Keywords: sexual related PTSD, PTSD, sexual assault

Conflict of interest:

No

Article ID: EPV1385

Talking about tourette - a clinical case with fetishistic disorder

Fragoeiro C. *
Almeida B.
Machado C.
Ferreira P.
Hospital de Magalhães Lemos , Psychiatry, Porto , Portugal
* Corresponding author.
Keywords: Neuropsychiatry, Tourette, SexualMedicine, Paraphilia

Conflict of interest:

No

Article ID: EPV1386

Factors having a role in help-seeking behavior among patients with sexual dysfunctions attending the psychiatry outpatient department in a tertiary care hospital of Bangladesh

Acharjee P. 1 *
Mullick M.S.I. 2
1 Chittagong Medical College , Dept. Of Psychiatry, Chittagong , Bangladesh
2 Bangabandhu Sheikh Mujib Medical University , Dept. Of Psychiatry, Dhaka , Bangladesh
* Corresponding author.
Keywords: Help-seeking, Sexual dysfunctions, Bangladesh

Conflict of interest:

No

Article ID: EPV1388

Impact of laparoscopic promontofixation for pelvic organ prolapse on sexuality and quality of life

Mannai J. 1 *
Majdoub R. 1
Romdhani I. 1
Naouar S. 2
Khribi M. 2
1 CHU IBEN EL JAZZAR, Psuchiatry, kairouan , Tunisia
2 CHU Iben EL Jazzar , Urology, Kairouan , Tunisia
* Corresponding author.
Keywords: Prolapse, Promontofixation

Conflict of interest:

No

Article ID: EPV1390

Gender identity of patients with mental disorders

Piskareva T.
Mental Health Research Center , Medical Psychology, Moscow , Russian Federation
Keywords: gender identity, schizotypal disorder, personality disorder

Conflict of interest:

No

Article ID: EPV1395

Hypoactive sexual desire disorder in review: from a gender non-specific disorder to a gender-specific one

Martins Correia J. *
Caetano S.
Hospital Sousa Martins - Unidade Local de Saúde da Guarda , Departamento De Psiquiatria E Saúde Mental, Guarda , Portugal
* Corresponding author.
Keywords: male hypoactive sexual desire disorder, Sexual Medicine, male sexual dysfunction

Conflict of interest:

No

Article ID: EPV1402
Subjects: Abstract, Sleep Disorders & Stress

Neuropsychiatric diseases among people working in call center

Etindele Sosso F.
Hôpital du Sacré-Coeur de Montréal ; Université du Québec à Montréal ;, Center for Advanced Research In Sleep Medicine; Institut Santé Et Société; Quebec Network On Suicide, Mood Disorders And Related Disorders , Montréal , Canada
Keywords: İnsomnia, sleepiness, Anxiety, Dépression

Conflict of interest:

No

Article ID: EPV1403

Assessing cognitive behavioral therapy for insomnia in individuals with cannabis use disorder utilizing actigraphy and serum biomarkers

Geagea L. 1 *
Tamim H. 2
Elbejjani M. 2
Chebaro M. 1
Kobeissy F. 3
Nakhle P. 1
Talih F. 1
1 American University of beirut , Psychiatry, beirut , Lebanon
2 American University of beirut , Internal Medicine, beirut , Lebanon
3 American University of beirut , Biochemistry, beirut , Lebanon
* Corresponding author.
Keywords: cannabis, İnsomnia, Therapy, biomarkers

Conflict of interest:

No

Article ID: EPV1405

Procrastination and stress levels in medical students with different mean mark

Artemieva M. *
Lazukova A.
Peoples' Friendship University of Russia ( RUDN University ), Department of Psychiatry And Medical Psychology, Moscow , Russian Federation
* Corresponding author.
Keywords: procrastination, stress

Conflict of interest:

No

Article ID: EPV1406

Looking beyond excessive daytime somnolence: not everything are mood disorders.

Garcia-Malo C. 1
Boi S. 2 *
1 Sleep Rearch Institute , Neurology, madrid , Spain
2 Hospital Universitario Puerta de Hierro de Majadhonda , Psychiatry, Majadahonda , Spain
* Corresponding author.
Keywords: sleep-disorder, excesive-daytime somnolence, narcolepsy

Conflict of interest:

No

Article ID: EPV1407

A rare sleep disorder, a pharmacological side-effect? “Doctor, I find traces of food in my bed”.

Boi S. 1 *
Garcia-Malo C. 2
1 Hospital Universitario Puerta de Hierro de Majadhonda , Psychiatry, Majadahonda , Spain
2 Sleep Rearch Institute , Neurology, madrid , Spain
* Corresponding author.
Keywords: parasomnia, phamarcological side effect, Zolpidem, eating-disorder

Conflict of interest:

No

Article ID: EPV1408

Analysis of the quality of sleep in adolescents who consult in the emergency department due to suicidal ideation.

Pérez Sánchez S. 1 *
Martín Herrero I. 1
Crespo Portero A. 2
Güimil Raya D. 1
1 Morales Meseguer Public University Hospital , Psychiatry, Murcia , Spain
2 Lorca Mental health Center , Psychiatry, Lorca , Spain
* Corresponding author.
Keywords: sleep, adolescents, suicidal ideation.

Conflict of interest:

No

Article ID: EPV1409

“the early bird catches the worm” – behavioural sleep problems in children and how to treat them

Pinto M. *
Lobato De Sousa M.J.
Almeida M.G.
Maia C.
Centro Hospitalar do Tâmega e Sousa, Child And Adolescent Psychiatry, Guilhufe , Portugal
* Corresponding author.
Keywords: sleep, behavioural insomnia of childhood, Children, behavioural interventions

Conflict of interest:

No

Article ID: EPV1410

Current evidence-based therapeutic recommendations in narcolepsy

Vasiliu O.
University Emergency Central Military Hospital Dr. Carol Davila , Psychiatry, Bucharest , Romania
Keywords: narcolepsy, psychopharmacology, H3 inverse agonists, norepinephrine-dopamine reuptake inhibitor

Article ID: EPV1412

The relationship between functional state self-regulation resources and job satisfaction in contact center operators

Titova M. 1 *
Nematova K. 2
1 Lomonosov Moscow state university , Faculty of Psychology, Moscow , Russian Federation
2 Baku branch of Lomonosov Moscow state university , Faculty of Psychology, Baku , Azerbaijan
* Corresponding author.
Keywords: stress, job satisfaction, Resilience, functional state self-regulation resources

Conflict of interest:

No

Article ID: EPV1414
Subjects: Abstract, Suicidology and Suicide Prevention

Suicide attempts in the psychiatric day hospital: a retrospective study

Borges L. 1 *
Duarte P. 2
Rita Moura A. 2
Maia A. 2
Halpern C. 2
1 Centro Hospitalar Universitário do Algarve , Psychiatry, Portimão , Portugal
2 Centro Hospitalar de Lisboa Ocidental , Psychiatry, Lisbon , Portugal
* Corresponding author.
Keywords: Suicide, DAY HOSPITAL, suicide attempt

Conflict of interest:

No

Article ID: EPV1418

Suicide prevention actions carried out by an academic psychiatry service in rio de janeiro city

Jaber Filho J. 1 *
Verissimo J. Jr 2
Hollanda A. 3
Geraldes P.C. 4
1 Clínica Jorge Jaber, Saúde Mental , Vargem Pequena , Brazil
2 Clínica Jorge Jaber, Saúde Mental , Rio de Janeiro, Rio de Janeiro, Brasil , Brazil
3 Clínica Jorge Jaber, Saúde Mental , Rio de Janeiro , Brazil
4 Clínica Jorge Jaber, Saúde Mental, Vargem Pequenaos Reis , Brazil
* Corresponding author.

Conflict of interest:

No

Article ID: EPV1419

Childhood sexual abuse and suicidal behavior in bipolar disorder patients

Maamouri R. 1 *
Ellini S. 2
Ghazouani N. 3
Cheour M. 4
1 Razi hospital , Psychiatry E, tunis , Tunisia
2 Razi hospital , Psychiatry E, manouba, Tunisia
3 Razi University Hospital , Psychiatry, Mannouba , Tunisia
4 Tunis El Manar University , Faculty of Medicine of Tunis, Department of Psychiatry "e", Razi Hospital , La Manouba, Tunisia
* Corresponding author.
Keywords: sexual abuse, childhood trauma, Bipolar Disorders, suicidal behavior

Conflict of interest:

No

Article ID: EPV1420

Impulsivity and suicidal behavior in bipolar disorder patients

Maamouri R. 1 *
Ellini S. 2
Ghazouani N. 3
Ati C. 4
Cheour M. 5
1 Razi hospital , Psychiatry E, Ben arous, Tunisia
2 Razi hospital , Psychiatry E, manouba, Tunisia
3 Razi University Hospital , Psychiatry, Mannouba , Tunisia
4 Razi hospital , Psychiatry E, tunis , Tunisia
5 Tunis El Manar University , Faculty of Medicine of Tunis, Department of Psychiatry "e", Razi Hospital , La Manouba, Tunisia
* Corresponding author.
Keywords: suicidal behavior, Prevention, Impulsivity, Bipolar Disorders

Conflict of interest:

No

Article ID: EPV1421

Methods of suicide attempts in a tunisian sample of patients with bipolar disorders

Maamouri R. 1 *
Ellini S. 2
Ghazouani N. 3
Rebai A. 4
Cheour M. 5
1 Razi hospital , Psychiatry E, Ben arous, Tunisia
2 Razi hospital , Psychiatry E, manouba, Tunisia
3 Razi University Hospital , Psychiatry, Mannouba , Tunisia
4 Razi University Hospital , Adult Outpatient Psychiatry Department, Mannouba , Tunisia
5 Tunis El Manar University , Faculty of Medicine of Tunis, Department of Psychiatry "e", Razi Hospital , La Manouba, Tunisia
* Corresponding author.
Keywords: Bipolar Disorders, Transcultural, SUICIDE ATTEMPTS, methods

Conflict of interest:

No

Article ID: EPV1422

Self-reported measures vs. clinical scales to assess the effectiveness of dialectical behavioral therapy in adolescents at high risk of suicide

Martin Villalba I.
Hospital Clinic de Barcelona , Departament Of Psychiatry And Clinical Psychology, BARCELONA , Spain
Keywords: dialectical behavior therapy, suicidal behavior, DBT, adolescents

Conflict of interest:

No

Article ID: EPV1423

Suicide risk among multiple suicide attempters

Mastrangelo M. 1 *
Anibaldi G. 1
De Luca G. 1
Imbastaro B. 1
Montalbani B. 1
Rogante E. 2
Sarubbi S. 2
Ronca A. 2
Forte A. 2
Erbuto D. 2
Pompili M. 2
1 Sapienza University of Rome, Psychiatry Residency Training Program , Faculty of Medicine and Psychology, Rome , Italy
2 Sapienza University of Rome , Neurosciences, Mental Health And Sensory Organs , Rome , Italy
* Corresponding author.
Keywords: multiple suicide attempts, lethality, Suicide prevention, suicide risk

Conflict of interest:

No

Article ID: EPV1424

Epidemiological aspects of self-harm hospital cases in panama from 2009-2017

Moreno I. 1 *
Castelpietra G. 2
Higuera G. 1
Castro F. 1
Gómez B. 1
Motta J. 1
Goti R. 3
1 Gorgas Memorial Institute for Health Studies , Diets, Panama , Panama
2 Central Health Directorate Region Friuli, Venezia Giulia , Trieste, Primary Care Services Area, Trieste, Italy
3 Ministry of Health, Mental Health , Panama , Panama
* Corresponding author.
Keywords: Self-harm, Suicide, Panama

Conflict of interest:

No

Article ID: EPV1425

The risk factors of self-directed violence in severe mental illness. A scoping review.

Moreno-Calvete M.C.
Biocruces Bizkaia Health Research Institute, Bizkaia Mental Health Network , Osakidetza, Basque Health Service, Mental Health Service , Bilbao , Spain
Keywords: Severe mental illness, Self-harm, risk factor, Suicide

Conflict of interest:

No

Article ID: EPV1426

Study of revenues in psychiatric unit in relation to suicidal behaviors

Padilla Romero P. 1 *
Dhiver Cantalejo Y. 2
Lopez-Arteaga T. 2
1 Hospital General Nuestra Señora del Prado , Psychiatry, Talavera, Toledo , Spain
2 Hospital General Nuestra Señora del Prado , Psychiatry, Talavera de la Reina , Toledo , Spain
* Corresponding author.
Keywords: suicidal behaviors, psychiatric hospitalization unit, admission, diagnoses

Conflict of interest:

No

Article ID: EPV1429

Intento de suicidio en fuerteventura, observación de noviembre 2008 a mayo 2009; ¿cuáles son los principales factores asociados?

Segura E.
Clínica Privada, Salud Mental , Madrid , Spain
Keywords: asociados, intento, suicidio, factores

Conflict of interest:

No

Article ID: EPV1430

Detection of suicidal risk factors in a sample of patients followed in an intensive intervention program.

Valtueña García M. 1 *
González Suarez A. 2
Lago García L. 1
Ludwig C. 1
López Fernández J. 3
Huergo Lora M. 1
Arias Martino R. 4
Ocio León S. 5
1 Hospital Vital Álvarez Buylla ., Psychiatry, Asturias , Spain
2 Hospital Universitario Central de Asturias , Psychiatry, Asturias , Spain
3 Atención Primaria Área VIII del Principado de Asturias , Centro De Salud, Asturias , Spain
4 Hospital Vital Álvarez Buylla ., Unidad De Tratamiento De Toxicomanías área Vii Del Servicio De Salud Del Principado De Asturias, Asturias , Spain
5 Hospital Vital Álvarez Buylla ., Director Área De Gestión Clínica, Asturias , Spain
* Corresponding author.
Keywords: Prevention of suicidal attempts, predictors of suicide, Intensive Intervention Program, high suicidal risk

Conflict of interest:

No

Article ID: EPV1431

Social-psychological factors of attempted suicide in adolescence

Oleshkevich V. 1
Pechnikova L. 2
Burlakova N. 3 *
Buromskaya N. 4
1 Scientific Practical Children's Mental Health Centre n. a. G. Sukhareva of Moscow City Department of Healthcare, -, Moscow , Russian Federation
2 Lomonosov Moscow State University , Department of Neuro- And Pathopsychology, Moscow , Russian Federation
3 Lomonosov Moscow State University , Faculty of Psychology, Department of Neuro- And Pathopsychology, Moscow , Russian Federation
4 G.E. Sukhareva Research Practical Centre of Children and Adolescents Mental Health, No 1, Moscow , Russian Federation
* Corresponding author.
Keywords: narcissistic crise, adolescent crisis, Suicide, prevention of suicides

Conflict of interest:

No

Article ID: EPV1432

Suicidal ideation in a depressive episode. A clinical case

Cozar Ortiz J. *
Benavente López S.
Ric Benito P.
Hernández Caro C.M.
Plaza Yuste M.
Torrente Seoane C.
Rodríguez Delgado C.
Torres Varona F.J.
Ainslie Mata Y.
Faber D.
Juárez Calvo V.
Rodríguez Villarino M.C.
Suarez Guinea R.
Rubio Zavala I.
Iglesias García C.
Pérez-Iñigo Gancedo J.L.
Presa García M.
Hospital Central de la Defensa Gómez Ulla, Psychiatry And Mental Health Department , Madrid , Spain
* Corresponding author.
Keywords: psychotherapy, venlafaxine, Depressive, Suicide

Conflict of interest:

No

Article ID: EPV1433

Therapeutic interventions for suicide behaviour: 12-months outcomes for the suicidal behaviour management program for suicide prevention (CARS) in cantabria (spain)

De Santiago-Díaz A.I. 1 *
Gómez-Revuelta M. 1
García-Rumayor E. 1
Boada-Antón L. 1
Ibañez-Alario M. 1
Sánchez-Blanco L. 2
Artal-Simón J.Á. 1
1 Hospital Universitario Marqués de Valdecilla , Servicio De Psiquiatría, SANTANDER , Spain
2 Hospital Galdakao-Usansolo , Servicio De Psiquiatría, Galdakao , Bizkaia , Spain
* Corresponding author.
Keywords: Suicide, suicide behaviour, Suicide prevention

Conflict of interest:

No

Article ID: EPV1434

Psychotherapeutic group intervention designed specifically to approach suicidal behavior: a pilot study.

De Santiago-Díaz A.I. *
Carceller-Meseguer T.M.
Artal-Simón J.Á.
Hospital Universitario Marqués de Valdecilla , Servicio De Psiquiatría, SANTANDER , Spain
* Corresponding author.
Keywords: Suicide, suicide behaviour, psychotherapie group intervention, Suicide prevention

Conflict of interest:

No

Article ID: EPV1436

“Attitudinal beliefs on suicide and suicide risk in young people of a private university of monteria”.

Muñoz Argel M.N. *
Uribe Urzola A.
Ruiz Gonzalez E.
Arcos Guzman M.
Ramos Vidal I.
Universidad Pontificia Bolivariana , Psicología , Monteria , Colombia
* Corresponding author.
Keywords: suicide risk, Suicide, attitudinal beliefs, University students

Conflict of interest:

No

Article ID: EPV1437

Intrapersonal anti-suicide barriers in dental students

Nikolaev E.
Ulianov Chuvash State University , Department of Social And Clinical Pscyhology, Cheboksary , Russian Federation
Keywords: anti-suicide barriers, dental students, faith, satisfaction

Conflict of interest:

No

Article ID: EPV1442

Suicide and emotion regulation: which difficulties are involved?

Beomonte Zobel S. *
Velotti P.
Rogier G.
Sapienza Università di Roma , Dynamical And Clinical Psychology, Roma , Italy
* Corresponding author.
Keywords: emotion regulation, Systematic Review, Suicide

Conflict of interest:

No

Article ID: EPV1443

Vulnerable or grandiose? The two faces of suicide in pathological narcissism.

Beomonte Zobel S. *
Rogier G.
Velotti P.
Sapienza Università di Roma , Dynamical And Clinical Psychology, Roma , Italy
* Corresponding author.
Keywords: vulnerability, Suicide, narcissism, grandiosity

Conflict of interest:

No

Article ID: EPV1444

Suicidare behavior and risk factors: observation study in the department of mental health

Carluccio A. 1
Campi S. 2 *
1 ASL Lecce , Department of Mental Health, ITALIA , Italy
2 asl lecce , Department of Mental Healthh, lecce, Italy
* Corresponding author.
Keywords: suicide behavior risk factors, Suicide, risk factors, suicide attemps

Conflict of interest:

No

Article ID: EPV1445

Harm minimisation approaches for management of self-harm: a study describing the prevalence and characteristics of patients who self-harm and use harm minimisation techniques

Cliffe C. *
Rowe S.
Pitman A.
UCL, Psychiatry , London , United Kingdom
* Corresponding author.
Keywords: Self-harm, Suicidality, harm minimisation, emotional dysregulation

Conflict of interest:

No

Article ID: EPV1446

Demographic, social psychological and risk factors in the dynamic of young suicidal patient

Comaniciu A. 1 *
Muntean L. 1
Lemeti L. 1
Nirestean A. 1 2
1 Mures County Hospital , Psychiatric Clinic No. 2, Targu Mures , Romania
2 George Emil Palade University of Medicine , Pharmacy, Science and Technology of Targu Mures, Psychiatry, Targu Mures , Romania
* Corresponding author.
Keywords: Suicide, risk factors, young patients, Social support

Conflict of interest:

No

Article ID: EPV1448

Identification of groups and factors of suicidal risk for the development of suicide prevention measures in the kyrgyz republic

Galako T.
Kyrgyz State Medical Academy , Psychiatry, Medical Psychology And Drug Abuse Department , Bishkek, Kyrgyzstan
Keywords: Suicide prevention, suicide situation in Kyrgyzstan, Groups and Factors of Suicidal Risk, suicidal statistics

Conflict of interest:

No

Article ID: EPV1449

A novel approach to identifying causal factors for risk events: applying the hazard and operability study methodology to mental health services.

Nathan R. *
Nathan K.
University of Manchester, Chemical Engineering And Analytical Science , Manchester , United Kingdom
* Corresponding author.
Keywords: risk, safety, investigation, Heuristic

Conflict of interest:

No

Article ID: EPV1451

Does physical ill-health increase the risk of suicide? A census-based follow-up study of over 1 million people

O'Reilly D. 1 *
Onyeka I. 2
Maguire A. 2
Ross E. 1
1 Queen's University Belfast , Centre For Public Health, Belfast , United Kingdom
2 Queens University Belfast , Centre For Public Health, Belfast , United Kingdom
* Corresponding author.

Conflict of interest:

No

Article ID: EPV1453

Complicated grief and suicidal behavior: barriers, boundaries and access to mental health treatment

Pinto S. 1 *
Soares J. 1
Silva A. 2
Curral R. 2
1 Centro Hospitalar Universitário de São João , Department of Psychiatry, Porto , Portugal
2 Centro Hospitalar Universitário de São Jão , Department of Psychiatry, Porto , Portugal
* Corresponding author.
Keywords: complicated grief, Suicide, suicidal behavior, Stigma

Conflict of interest:

No

Article ID: EPV1454

Suicidal behavior in the inpatient setting: understanding the phenomenon

Pinto S. 1 *
Delgado A.M. 1
Guedes R. 1
Andrade F. 1
Morais J. 1
Silva A. 2
Silveira C. 2
Curral R. 1
1 Centro Hospitalar Universitário de São João , Department of Psychiatry, Porto , Portugal
2 Centro Hospitalar Universitário de São Jão , Department of Psychiatry, Porto , Portugal
* Corresponding author.
Keywords: Suicide, inpatient, psychiatric disorder, Prevention

Conflict of interest:

No

Article ID: EPV1455

The association between COMT RS4818 polymorphism and suicidality in schizophrenia

Madzarac Z. 1
Sagud M. 2 *
Tudor L. 3
Mihaljevic Peles A. 2
Pivac N. 3
1 University Hospital Centre Zagreb , Psychiatry, Zagreb , Croatia
2 School of Medicine, Unversity of Zagreb , Psychiatry, Zagreb , Croatia
3 Rudjer Boskovic Institute , Laboratory For Molecular Neuropsychiatry, Division Of Molecular Medicine, Zagreb , Croatia
* Corresponding author.
Keywords: schizophrenia, Suicidality, COMT

Conflict of interest:

No

Article ID: EPV1456

Suicide statistics: do we believe them?

Snowdon J.
Sydney University , Psychological Medicine, Sydney , Australia
Keywords: suicide rates undetermined cause Europe longitudinal

Conflict of interest:

No

Article ID: EPV1458

Suicide in a HIV-infected adult population cared at a chilean public hospital: an appraisal of the issue

Wolff Levy C. 1 *
Wolff M. 2
1 Universidad Mayor /Universidad de Santiago , Departamento De Psiquiatría, Santiago , Chile
2 Universidad de Chile , Departamento De Medicina Interna, Campus Sur, Santiago , Chile
* Corresponding author.
Keywords: Chile, Suicide, HIV, AIDS

Conflict of interest:

No

Article ID: EPV1460

Suicidality in schizophrenia, delusional disorder, schizoaffective disorder and first-episode of psychosis: a systematic review

Álvarez Pedrero A. *
Guàrdia Delgado A.
González-Rodríguez A.
Betriu M.
Parra I.
Monreal J.A.
Palao Vidal D.
Labad J.
Parc Taulí University Hospital . Autonomous University of Barcelona (UAB) . I3PT, Mental Health , Sabadell , Spain
* Corresponding author.
Keywords: Delusional disorder, schizophrenia, Suicide, psychosis

Conflict of interest:

No

Article ID: EPV1462

Suicide attempt as first manifestation of first-episode psychosis.

Carvalheiro A.M. *
Maia J.
Leiria Hospital Center , Psychiatry And Mental Health Department, Leiria , Portugal
* Corresponding author.
Keywords: prodromal stage, Suicide, first-episode psychosis, psychosis

Conflict of interest:

No

Article ID: EPV1465

Suicide in children and adolescents: a tunisian perspective from 2009 to 2017

Mannai J. 1 *
Mosbahi A. 2
Majdoub W. 2
Turki E. 2
1 CHU IBEN EL JAZZAR, Psychiatry, kairouan , Tunisia
2 CHU Iben EL JAZZAR , Forensic Medicine, kairouan , Tunisia
* Corresponding author.
Keywords: 271181

Conflict of interest:

No

Article ID: EPV1468
Subjects: Abstract, Training in Psychiatry

Difficulties in assessing mental illness stigma in medical students.

Callizo Silvestre A. 1 *
Martín Jimenez A. 2
Moreno Pérez A. 2
1 Área de gestiónclínica de psiquiatría y salud mental del Hospital Universitario Príncipe de Asturias (HUPA) –, Centro De Salud Mental Francisco Díaz . AlcaláDe Henares (spain), Alcalá de Henares , Spain
2 Área de gestión clínica de psiquiatría y salud mental del Hospital Universitario Príncipe de Asturias (HUPA) –, Hospital Universitario Príncipe De Asturias, Alcalá de Henares , Spain
* Corresponding author.
Keywords: Stigma, medical students, mental health, Qualitative Research

Conflict of interest:

No

Article ID: EPV1469

A pre-project survey for use of a social media platform for trainee psychiatrists across 5 mental health trusts in UK

Matheiken S. 1 *
Runciman R. 2
1 EAST LONDON NHS FOUNDATION TRUST, Fountains Court, BEDFORD , United Kingdom
2 Gloucestershire Health and Care NHS Foundation Trust , Weavers Croft, Stroud , United Kingdom
* Corresponding author.
Keywords: psychiatrists, online platform, engagement

Conflict of interest:

No

Article ID: EPV1470

On psychoeducation and psychosomatics

Michailov M. 1 *
Neu E. 2
Bauer H.W. 3
Hofstetter A. 4
Weisenbacher E. 5
Weber G. 6
1 Inst. Umweltmedizin (IUM) c/o ICSD/IAS e.V. POB 340316 , 80100 Muenchen, Germany (Int. Council Sci. Develop./Int . Acad. Sci. Berlin-Innsbruck-Muenchen-NewDelhi-Paris-Sofia-Vienna) , Pharmacophysiology, Muenchen, Germany
2 Inst. Umweltmed. c/o ICSD e.V ., Pharmacophysiology, München , Germany
3 Univ. München & Free Univ. Berlin , Med. Fac., München, Germany
4 Univ. München, Klinikum Großhadern (dir.a.d.) , München , Germany
5 Univ . München Med. Fac., & Premium Med. Clinic (dir.), München , Germany
6 Univ. Lxbg. & Vienna , Fac. Psychology (ex-dean), Vienna , Austria
* Corresponding author.
Keywords: UNO-Agenda 21, psychoeducation, psychosomatics, psychooncology

Conflict of interest:

No

Article ID: EPV1471

Migration momentum in macedonia for early career psychiatrists

Milutinović M. 1 *
Kalpak G. 1
Pinto Da Costa M. 2
1 University Clinic of Psychiatry Skopje , Department For Affective Disorders, Skopje , North Macedonia
2 Queen Mary University of London , Unit Of Social And Community Psychiatry, London , United Kingdom
* Corresponding author.
Keywords: Early Career Psychiatrists, migration, brain drain, Macedonia

Conflict of interest:

No

Article ID: EPV1473

Case reporting as a tool for learning and teaching

Wynn R. *
Nissen T.
UiT The Arctic University of Norway , Clinical Medicine, Tromso , Norway
* Corresponding author.
Keywords: case history, teaching, learning, case reporting

Conflict of interest:

No

Article ID: EPV1479

Ethical reasoning learning sessions among tunisian medical students: a satisfactory survey

Sahli L. 1
Zgueb Y. 2
Ouali U. 2
Ayedi S. 2 *
Jomli R. 2
Nacef F. 2
1 Razi Hospital , Psychiatry Department 'avicenne', Manouba , Tunisia
2 Razi Hospital, A "avicenna" Department , Tunis , Tunisia
* Corresponding author.
Keywords: Reasoning, ethics, medical student, Pedagogy

Conflict of interest:

No

Article ID: EPV1480

How to learn obsessive compulsive disorder by simulation?: A tunisian experience

Sahli L. 1
Zgueb Y. 2
Ouali U. 2
Ayedi S. 2 *
Jlidi A. 2
Jomli R. 2
Nacef F. 2
1 Razi Hospital , Psychiatry Department 'avicenne', Manouba , Tunisia
2 Razi Hospital, A "avicenna" Department , Tunis , Tunisia
* Corresponding author.
Keywords: simulation, obsessive compulsive disorder, medical student, Training

Conflict of interest:

No

Article ID: EPV1481

Motivation in residents. Reasons why physicians, psychologists and nurses choose to train and work in mental health

Carracedo Sanchidrián D. 1 *
Cano Arenas A. 2
Hernández-Calle D. 1
1 Hospital Universitario la Paz, Mental Health , Madrid , Spain
2 Universidad Pontificia de Comillas, Unidad De Intervención Psicosocial , Madrid , Spain
* Corresponding author.
Keywords: Motivations, mental health, Training, Residents

Conflict of interest:

No

Article ID: EPV1484

Differences in burnout prevalence between mental health professionals working in an emergency psychiatric unit and general population: a cross-sectional survey

Ladea M. 1 2 *
Patrascu M.R. 2
Bran M. 3
Sofia A. 1 2
Ionescu T.C. 1 2
1 University of Medicine and Pharmacy''Carol Davila'' , Psychiatry, Bucharest , Romania
2 Alexandru Obregia Clinical Hospital , Department 3, Bucharest , Romania
3 Coltea Hospital Bucharest , Psychiatry, Bucharest , Romania
* Corresponding author.
Keywords: bureaucracy, administrative stuff, Burnout, Mental Health Professionals

Conflict of interest:

No

Article ID: EPV1485
Subjects: Abstract; Women, Gender and Mental Health

Role of female sex hormones on cognitive functions in schizophrenic women

Amer R. 1 *
Ramadan E. 1
Morad H. 2
Shama G. 1
1 Faculty of Medicine/ Tanta University / Egypt, Psychiatry And Neurology Department, Tanta , Egypt
2 Faculty of Medicine/ Tanta University / Egypt, Clinical Pathology Department, Tanta , Egypt
* Corresponding author.
Keywords: schizophrenia, women, sex hormone, cognitive functions

Conflict of interest:

No

Article ID: EPV1488

Factors associated with psychiatric morbidity in female medical doctors in kwara state, north-central, nigeria

Buhari O. 1 *
Ogunmodede A. 2
1 University of Ilorin , Department of Behavioral Sciences, Ilorin , Nigeria
2 university of Ilorin Teaching Hospital , Ilorin, Department of Behavioral Sciences, ilorin, Nigeria
* Corresponding author.
Keywords: female doctor, factors, associated, psychiatric morbidity

Conflict of interest:

No

Article ID: EPV1489

Pseudocyesis: a case of an almost forgotten disorder

Derganc M. *
Pirtovsek Savs A.
University Psychiatric Clinic Ljubljana , Ckp, Ljubljana , Slovenia
* Corresponding author.
Keywords: miscarriage, borderline intellectual functioning, pseudocyesis

Conflict of interest:

No

Article ID: EPV1492

Case report of treatment resistant postpartum psychosis as a possible manifestation of schizophrenia.

Kočāne A.
SLC Hospital Gintermuiza , Psychiatry, Jelgava , Latvia
Keywords: clozapine, postpartum, psychosis

Conflict of interest:

No

Article ID: EPV1493

Egg donation and bonding: a case report

Lakis S. 1 *
Mantilla Reyes M.F. 2
Ximenez De Embun Ferrer I. 2
Roca Lecumberri A. 2
Naranjo Díaz C. 2
Torres Gimenez A. 2
Fernandez Gomis N. 2
Roda Guillen E. 2
García - Esteve L. 2
1 HOSPITAL CLINIC DE BARCELONA , Institut Clínic De Neurociencies (icn). Unidad De Salud Mental Perinatal , Barcelona , Spain
2 Hospital Clínic de Barcelona , Institut Clínic De Neurociencies (icn). Unidad De Salud Mental Perinatal , Barcelona , Spain
* Corresponding author.
Keywords: bonding, egg donation, postpartum

Conflict of interest:

No

Article ID: EPV1495

Characteristics of the emotional sphere of women in the IVF program with natural and donor ova

Sedova E. *
Gardanova Z.
Kalina S.
Pirogov Russian National Research Medical University , Psychological-social Faculty, Moscow , Russian Federation
* Corresponding author.
Keywords: IVF, donor ova, infertility

Conflict of interest:

No

Article ID: EPV1496

Do french health agency’s guidelines have an effect on prescription of valproic acid and derivatives (valpromide, sodium divalproate) in childbearing-aged women?

Yosofi C. *
Communier L.
Bellay R.
Langrée B.
Marie N.
Centre hospitalier Guillaume Regnier , Pharmacie, Rennes , France
* Corresponding author.
Keywords: Childbearing-aged women, Valproic acid and derivatives, moodstabilizer

Conflict of interest:

No

Article ID: EPV1497

Assessment of caregivers’ strain and burden during radiation therapy of cancer patients in western greece using modified caregivers’ strain index scale and zarit burden interview

Grigoratou K. 1
Argyropoulos K. 1
Avramidis D. 2 *
Alexopoulos P. 3
Kardamakis D. 4
Gourzis P. 3
Jelastopulu E. 1
1 School of Medicine, University of Patras , Greece , Public Health, Patras, Greece
2 University of Patras , School of Medicine, Patra , Greece
3 School of Medicine, University of Patras , Greece , Psychiatry , Patras, Greece
4 School of Medicine, University of Patras , Greece , Radiation-oncology, Patras, Greece
* Corresponding author.
Keywords: cancer patients, Caregivers, Burden, Radiation Therapy

Conflict of interest:

No

Article ID: EPV1498

Hermeneutical epistemic injustice in motherhood: guilt, caring and grieving in fatal infant diseases.

Cerame Del Campo Á. 1 *
Franco Soler A.V. 2
Costa Ferrera Da Silva M.L. 3
Coucheiro Limeres P. 1
Aguilar Romero M.D.C. 4
1 Instituto psiquiatrico Jose Germain, Psyhciatric Trainee , leganes , Spain
2 Instituto psiquiatrico Jose Germain , Clinical Psychology Trainee, leganes , Spain
3 Instituto psiquiatrico Jose Germain , Psyhciatrist, leganes , Spain
4 Hospital Severo Ochoa , Psychiatry, Leganés , Spain
* Corresponding author.
Keywords: grieving, motherhood, epistemic injustice, guilt

Conflict of interest:

No

Article ID: EPV1499

Protocol for transsexual, transgender and gender-nonconforming persons care on mental health service of mostoles university hospital.

De FrutosGuijarro J.J. 1 *
Martín Aragón R. 2
Chinchurreta N. 3
Moreno Menguiano C. 1
1 Centro de Salud Doctor Luengo Rodriguez , Servicio De Psiquiatría, Mostoles , Spain
2 Complejo Hospitalario Mancha Centro ., Unidad De Salud Mental Infanto-juvenil ., Alcázar de San Juan., Spain
3 HOSPITAL PUERTA DE HIERRO , Psychiatry, MADRID , Spain
* Corresponding author.
Keywords: transgender, gender identity, gender diversity

Conflict of interest:

No

Article ID: EPV1500

Case report: maternity abroad: the importance of support

Garcia Murillo L. *
Marin Vila M.
Díaz De Neira M.
Forti Buratti A.
Palanca Maresca I.
Hospital Universitario Puerta de Hierro , Child And Adolescent Psychiatry, Majadahonda, Madrid , Spain
* Corresponding author.
Keywords: support, Maternity, Perinatal, Anxiety

Conflict of interest:

No

Article ID: EPV1501

Prescribing antipsychotics in pregnancy: considerations on the treatment in a clinical case

Mainar De Paz V. 1 *
Martín Alvarez C. 2
Bravo Arráez M. 3
1 Centro de Salud Mental Maresme Nord , Psiquiatría, Barcelona , Spain
2 Hospital Universitario de Fuenlabrada, Centro De Salud Mental , Madrid , Spain
3 Hospital Universitario Severo Ochoa , Psiquiatria, Madrid , Spain
* Corresponding author.

Conflict of interest:

No

Article ID: EPV1502

Peculiarities of neurotic anxious-depressive disorders in internally displaced women and combatants’s wives

Markov A. 1
Markova M. 2 *
Rahman L. 3
Shpylovyi I. 3
Kosenko K. 4
1 Kharkiv Medical Academy of Postgraduate Education , Sexology, Medical Psychology Medical And Psychological Rehabilitation, Kharkiv, Ukraine
2 Kharkiv Medical Academy of Postgraduate Education , Sexology, Medical Psychology, Medical Abd Psychological Rehabilitation, Kharkiv, Ukraine
3 Danylo Halytsky Lviv National Medical University , Psychiatry, Psychology And Sexology, Lviv, Ukraine
4 Odessa National Medical University , Psychiatry, Odessa , Ukraine
* Corresponding author.
Keywords: neurotic disorders, internally displaced persons, women, combatants’s wives

Conflict of interest:

No

Article ID: EPV1503

Breastfeeding practice in women displaced by armed violence in a rural area of colombia

Muñoz Argel M.N. 1 *
Romero Otalvaro A. 1
Vélez Carvajal J. 2
Naranjo Quintero V. 3
Gonzalez L. 1
1 Universidad Pontificia Bolivariana , Psicología , Monteria , Colombia
2 Universidad Pontificia Bolivariana, Social Communication , Monteria , Colombia
3 Universidad Pontificia Bolivariana, Humanist Training , Medellin , Colombia
* Corresponding author.

Conflict of interest:

No

Article ID: EPV1508

Improving maternal and infant mental health: an integrated model of care for specialist perinatal mental health services

Miele M. 1
Austin K. 2
Mapepa I. 1
Halai K. 3 *
1 Imperial College London, Perinatal Mental Health Service - St Mary's Hospital , London , United Kingdom
2 Central North West London FT, Perinatal Mental Health Service - St Mary's Hospital , London , United Kingdom
3 CNWL FT, Perinatal Mental Health , London , United Kingdom
* Corresponding author.
Keywords: perinatal services, maternal mental illness, prescribing in pregnancy, infant mental health

Conflict of interest:

No

Article ID: EPV1511

When depression and insomnia tinges the reproductive pathways of a woman in assisted reproduction

Santamaría Rubio E. 1 *
Matas A. 2
Dominguez Ballesteros E. 3
1 Clínica López Ibor , Psychiatry, Maddrid , Spain
2 Hospital Infanta Leonor , Psychiatry, Madrid , Spain
3 Clinica Lopez Ibor , Child Psychiatry, Madrid , Spain
* Corresponding author.
Keywords: Dépression, Perinatal Depression, assisted reproduction

Conflict of interest:

No

Article ID: EPV1512

Clinical and socio-demographic characteristics associated with perinatal depression

Ciampi C. 1
Tarantino G. 1
De Felice G. 1
Palummo C. 1
Zinno F. 1 *
Sampogna G. 1
Del Vecchio V. 1
Giallonardo V. 1
Luciano M. 1
Torella M. 2
Fiorillo A. 1
1 University of Campania "Luigi Vanvitelli" , Department of Psychiatry, Naples , Italy
2 University of Campania "Luigi Vanvitelli" , Department of Woman, Child And General And Specialized Surgery, Naples , Italy
* Corresponding author.
Keywords: Perinatal Depression, women's health, Screening

Conflict of interest:

No

Article ID: EPV1513

Postpartum first-episode psychosis – a case report and literature review

Almeida C. *
Miranda J.
Carvalheiro A.M.
Barbosa M.
Santos Silva L.
Martinho S.
Araújo R.
Silva M.
Centro Hospitalar de Leiria, Psychiatry And Mental Health , Leiria , Portugal
* Corresponding author.
Keywords: postpartum, psychosis, First-Episode

Conflict of interest:

No

Article ID: EPV1516

Psychiatric treatment of pregnant women with severe mental illness in minsk, belarus

Kazakova O. 1 *
Hilko A. 2
Krautsova V. 3
Aizberg O. 4
Buhval O. 5
Reutfors J. 6
1 Pstchiatric Clinic of Minsk City, Inpatient Psychiatric Department, Minsk, Belarus
2 Belarusian State Medical University, Department of Psychiatry And Medical Psychology, Minsk, Belarus
3 Psychiatric Clinic of Minsk City, Deputy Chief Physician For Medical Examination And Rehabilitation, Minsk, Belarus
4 Belarusian Medical Academy of Postgraduate Education, Department of Psychiatry And Addictions, Minsk, Belarus
5 Republican Scientific and Practical Center of Mental Health, Inpatient Psychiatric Department #19, Minsk, Belarus
6 Karolinska University Hospital, Centre For Pharmacoepidemiology, Stockholm, Sweden
* Corresponding author.
Keywords: psychiatric treatment, pregnancies, hospitalisation

Conflict of interest:

No

